# Genetic structure of different ethnic populations at the frontotemporal dementia risk loci

**DOI:** 10.1371/journal.pone.0329809

**Published:** 2025-08-05

**Authors:** Volodymyr Mavrych, Maryam Alamil, Olena Bolgova, Volodymyr Dvornyk

**Affiliations:** 1 College of Medicine, Alfaisal University, Riyadh, Saudi Arabia; 2 College of Science and General Studies, Alfaisal University, Riyadh, Saudi Arabia; IRCCS San Raffaele Scientific Research Institute, ITALY

## Abstract

**Background and purpose:**

Frontotemporal dementia (FTD) is a devastating neurodegenerative disorder affecting behavior, language, and cognition. It has a complex and still poorly understood genetic basis. The prevalence of FTD and other neurodegenerative disorders varies in populations of different ethnicities. This study aimed to analyze the genetic structure of different ethnic populations at FTD risk loci and provide insights into possible genetic factors underlying the above variation.

**Methods:**

The data of single-nucleotide polymorphisms (in total 32) with genome-wide significance were extracted from the GWAS Database. The individual genotype data were retrieved from the 1000 Genomes Phase 3 Project. We analyzed several standard parameters of population genetic structure and computed a composite polygenic risk score. In total, five major ethnic superpopulations and 26 subpopulations were analyzed.

**Results:**

All populations were significantly differentiated (*P* << 10^−5^) at the FTD risk loci. Ethnic populations manifested clear differences in the enrichment/depletion patterns of the risk alleles as evidenced by heatmaps. The population-specific unweighted genetic risk scores were relatively low and averaged at 0.091 ± 0.078. The scores differed significantly at the super- and subpopulation levels.

**Conclusions:**

The results suggest that the major ethnic groups and their subpopulations differ by the allelic and genotypic structure at the FTD risk loci. This may be one of the key factors explaining the different prevalence of FTD across populations. However, currently available data on the epidemiology and genetics of FTD warrant further research.

## Introduction

Frontotemporal dementia (FTD) is a group of neurodegenerative disorders characterized by progressive degeneration of the frontal and temporal lobes of the brain, which leads to significant changes in behavior, personality, and language abilities. FTD is the second most common form of early-onset dementia after Alzheimer’s disease, yet it remains less well-known and understood [1].

FTD covers several clinical syndromes, including behavioral variant FTD (bvFTD), semantic variant primary progressive aphasia (svPPA), and nonfluent variant primary progressive aphasia (nfvPPA) [2–4]. Each variant has distinct clinical presentations, but they all share the common feature of progressive deterioration of frontal and temporal lobe functions. Understanding the epidemiology and genetics of FTD is crucial for improving diagnosis, developing targeted therapies, and providing appropriate support for patients and their families.

FTD can occur in familial and sporadic forms. Familial FTD (fFTD), defined as having at least one first-degree relative with a similar neurodegenerative disorder, accounts for approximately 30–50% of all FTD cases [5–7]. The remaining cases are considered sporadic, with no apparent family history of the disease. Familial FTD has been associated with mutations in several genes, specifically, *MAPT*, *GRN*, and *C9orf72*. However, mutations in these genes account for approximately 60–70% of all fFTD cases and only about 10–20% of sporadic cases [7]. This gap between the high heritability estimates derived from family studies and the proportion explained by known genetic factors represents the “missing heritability.” This phenomenon suggests that additional genetic factors, potentially including common variants with small individual effects, rare variants not captured by current genotyping approaches, gene-gene interactions, or gene-environment interactions, contribute significantly to the risk of FTD [6]. Understanding the complete genetic architecture of FTD, including this missing heritability component, is crucial for developing comprehensive genetic risk models and potential therapeutic approaches [5–7]. The genetic basis of sporadic FTD (sFTD) remains largely unknown. It may include rare genetic variations or a combination of genetic risk factors and environmental influences. The distinction between familial and sporadic cases is not always clear-cut, as limited family information or reduced penetrance of genetic mutations can sometimes obscure familial patterns [8].

FTD is relatively rare compared to other forms of dementia. The estimated point prevalence is 15–22/100,000, and the incidence is 2.7–4.1/100,000 [1]. Studies have shown that FTD accounts for about 5–15% of all dementia cases [9]. The incidence appears to increase with age until around 65 years, after which it plateaus or slightly decreases [2]. However, these figures may underestimate the true prevalence due to challenges in diagnosis and potential misclassification as psychiatric disorders or other types of dementia. It is important to note that the prevalence and incidence of FTD may vary depending on the specific subtype. For instance, the behavioral variant FTD (bvFTD) is generally more common than the language variants. Some studies suggest that bvFTD accounts for about 60% of all FTD cases, while semantic variant PPA and nonfluent variant PPA each account for roughly 20% of cases [3,4].

The prevalence of FTD varies across different geographic regions and ethnic groups, although more research is needed to understand these variations fully [10]. Its prevalence in European Caucasians has been estimated at 10.8 per 100,000 [11–13]. The data from other ethnic populations are limited. In the most recent systematic review, Llibre-Guerra *et al*. [14] reported the FTD prevalence within 1.2 to 1.7 per 1,000 in three Latin American countries. Some studies have suggested that FTD may be more common in Western countries than in Asian countries and Asian patients with FTD have a lower frequency of positive family history compared to data from Western cohorts [15]. For example, the comprehensive analysis of the Japanese population estimated the FTD prevalence at about 4.8 per 100,000 [16]. While these estimates are apparently biased due to the differences in the assessment methods and diagnostics, they nonetheless support the abovementioned interethnic variation in FTD prevalence. Additional evidence comes from the data about the prevalence of other dementia types, specifically, Alzheimer’s and Parkinson’s diseases, which were shown to share a genetic basis with FTD [17]. For example, a large-scale analysis of the data from the multiethnic Health and Retirement Study (HRS) reported the highest dementia prevalence in Blacks, followed by Hispanics and Whites [18].

Apart from socioeconomic and other non-genetic factors, ethnic-specific differences in population genetic structure at candidate loci may underlie the observed interpopulation disparities in the prevalence of multifactorial diseases (e.g., [19–22]).

Several GWAS have been conducted (primarily in European Caucasians) to identify loci that might contribute to FTD [8,23–26]. However, no analysis of the genetic structure of different ethnic populations has been conducted for FTD risk loci. The present study assessed whether interethnic differences in the genetic structure at FTD candidate loci correlate with documented differences in disease prevalence.

## Materials and methods

Thirty-two genome-wide significant candidate SNPs with identified risk alleles for FTD were extracted from the GWAS catalog (https://www.ebi.ac.uk/gwas/) and used for the present study (Table 1). The raw genetic data (allele and genotype information) were retrieved from the 1000 Genomes Phase 3 Project database [27] provided by Ensembl (http://ensembl.org). The analysis was conducted at two levels of population structure. Initially, data from five major ethnic superpopulations were examined: European (EUR), African (AFR), admixed American (AMR), East-Asian (EAS), and South-Asian (SAS). Following this, a more detailed analysis was performed on data from 26 smaller subpopulations (Table 2).

**Table 1:** Summary of the SNPs selected for the present study.

Genomic region	SNP	RA	OA	Location	MAF^*^	OR (95% CI)	Mapped gene(s)	Reference
2p16.3	rs17042852	**C**	T	Intron	0.04	2.82 (1.91-4.18)	*LINC01867*	[8]
2q33.3	rs13393316	A	**G**	Intron	0.10	1.89 (1.47-2.44)	*NDUFS1*	[23]
3p22.1	rs4676496	G	**A**	Intergenic	0.40	1.37 (1.28-1.49)	*RPSA*, *MOBP*	[26]
3q26.2	rs6809184	**T**	C	Intron	0.07	0.02 (0.01-0.02)	*TNIK*	[23]
3q29	rs13072484	**A**	G	Intron	0.21	1.51 (1.28-1.78)	*LINC02012*, *DLG1*-*AS1*	[23]
4q31.23	rs11099660	C	**T**	Intron	0.29	1.57 (1.37-1.77)	*LINC02507*	[8]
5q21.3	rs79095029	C	**G**	Regulatory region	0.05	2.56 (1.69-3.7)	*PJA2*, *KRT18P42*	[23]
5q35.1	rs517339	**C**	T	Intron	0.32	1.75 (1.37-2.23)	*ERGIC1*	[26]
6p21.32	rs9268856	C	**A**	Intron	0.29	1.24 (1.16-1.32)	*HLA*-*DRB9*	[28]
7p14.1	rs62443267	C	**T**	Intergenic	0.16	1.54 (1.28-4.35)	*STARD3NL*, *SFRP4*	[23]
7p21.3	rs7791726	**G**	C	Intergenic	0.39	1.85 (1.59-2.17])	*TMEM106B*, *VWDE*	[23]
7p21.3	rs6962939	**A**	T	Intron	0.06	1.27 (1.23-1.51)	*COL28A1*	[23]
7q21.3	rs2922921	**A**	G	Intergenic	0.01	1.35 (1.18-1.54)	*SEM1*, *MARK2P10*	[23]
8p21.3	rs36196656	A	**C**	Intron	0.45	1.55 (1.32-1.82)	*GFRA2*	[23]
8p23.1	rs10101195	C	**A**	Regulatory region	0.29	1.61 (1.33-1.96)	*GATA4*, *NEIL2*	[23]
8q11.23	rs9792144	**G**	C	Intron	0.16	0.02 (0.01-0.03)	*ST18*	[23]
8q21.13	rs3922636	**A**	C,G	Intergenic	0.003, 0.17	2.98 (1.71-4.25)	*RPL3P9*, *MITA1*	[23]
9p21.2	rs12554036	**T**	G	Intron	0.13	0.09 (0.08-0.10)	*MOB3B*	[26]
9q31.3	rs10816848	T	**A**	Intron	0.34	1.43 (1.22-1.67)	*PALM2*, *AKAP2*	[23]
10q22.3	rs4980079	**T**	C	Intron	0.28	1.46 (1.30-1.62)	*ZCCHC24*	[8]
11q14.2	rs302668	T	**C**	Intron	0.24	1.23 (1.09-1.41)	*RAB38*	[28]
12q23.1	rs10860097	**T**	A	Intron	0.04	3.04 (1.92-4.81)	*CFAP54*	[23]
14q12	rs229243	A	**C**	Intron	0.48	1.55 (1.33-1.82)	*G2E3*	[26]
17q12	rs3110643	**C**	T	Intron	0.08	1.65 (1.43-1.87)	*HNF1B*	[8]
17q25.3	rs906175	**T**	C	Intron	0.42	1.58 (1.33-1.87)	*CEP131*	[8]
18q23	rs7240419	**A**	G	Intron	0.32	1.52 (1.28-1.79)	*ATP9B*	[23]
19p13.11	rs12608932	**C**	A	Intron	0.43	1.37 (1.21-1.56)	*UNC13A*	[23]
19q13.32	rs6857	**T**	C	3’-UTR	0.11	1.67 (1.54-1.81)	*NECTIN2*	[29]
20p11.21	rs6076187	**A**	G	Intron	0.12	2.27 (1.58-3.26)	*LINC01721*	[23]
20p12.1	rs6111609	**A**	C	Intergenic	0.07	2.78 (1.59-3.97)	*RRBP1*, *BANF2*	[23]
20p12.2	rs6108746	**C**	T	Intron	0.13	3.24 (1.96-4.52)	*FAT1P1*, *LINC02871*	[23]
20q13.13	rs4810992	**A**	G	Non-coding transcript exon	0.13	1.2 (1.11-1.30)	*RNF114*	[26]

SNP, single-nucleotide polymorphism; RA, risk allele; OA, other allele; OR, odds ratio.

*Minor allele frequency in the 1000 Genomes Phase 3 combined population.

Minor allele is bold.

**Table 2:** Populations analyzed in the present study.

Code	Size	Description
ALL	2504	All phase 3 individuals
AFR	661	African
ACB	96	African Caribbean in Barbados
ASW	61	African Ancestry in Southwest US
ESN	99	Esan in Nigeria
GWD	113	Gambian in Western Division, The Gambia
LWK	99	Luhya in Webuye, Kenya
MSL	85	Mende in Sierra Leone
YRI	108	Yoruba in Ibadan, Nigeria
AMR	347	American
CLM	94	Colombian in Medellin, Colombia
MXL	64	Mexican Ancestry in Los Angeles, California
PEL	85	Peruvian in Lima, Peru
PUR	104	Puerto Rican in Puerto Rico
EUR	503	European
CEU	99	Utah residents with Northern and Western European ancestry
FIN	99	Finnish in Finland
GBR	91	British in England and Scotland
IBS	107	Iberian populations in Spain
TSI	107	Toscani in Italy
EAS	504	East Asian
CDX	93	Chinese Dai in Xishuangbanna, China
CHB	103	Han Chinese in Bejing, China
CHS	105	Southern Han Chinese, China
JPT	104	Japanese in Tokyo, Japan
KHV	99	Kinh in Ho Chi Minh City, Vietnam
SAS	489	South Asian
BEB	86	Bengali in Bangladesh
GIH	103	Gujarati Indian in Houston, TX
ITU	102	Indian Telugu in the UK
PJL	96	Punjabi in Lahore, Pakistan
STU	102	Sri Lankan Tamil in the UK

All loci were checked for correspondence to the Hardy-Weinberg equilibrium. Population genetic structure was analyzed using the standard parameters: allele frequencies, observed (*H*_o_) and expected (*H*_e_) heterozygosity [30], Wright’s fixation index (*F*, inbreeding coefficient) [31]. Interpopulation differentiation was estimated by *F*_ST_ [32] from AMOVA (analysis of molecular variance), Nei’s genetic distances [33] and the log-likelihood ratio (*G*) based exact test [34]. The above analyses were conducted using GenAlEx 6.5 [35] and GENEPOP v. 4.8.3 [36].

The enrichment/depletion of risk alleles in the populations compared to the global average at a locus was estimated using *P*-values of the hypergeometric distribution. The *P*-values were log_10_ transformed and assigned a positive or negative value depending on whether the allele was enriched or depleted. The results were visualized in a heatmap. The populations and alleles were clustered based on the average correlation.

The individual composite polygenic risk score (PRS) of FTD was calculated as suggested by Choi *et al*. and implemented in the PRSice-2 software [37]:

$PRS=\sum_{i=1}^{N}\left(\beta_{i}\times {SNP}_{i}\right)$,

where *β*_*i*_ is the effect size of the *i-*th SNP from the GWAS dataset, *SNP*_*i*_ is the allele count (0/1/2 for additive model) of the *i*-th SNP in the target sample, and *N* is the number of overlapping SNPs between the base and target datasets. An individual with two copies of a risk allele at each SNP will have a maximum risk score of 1, whereas one with no risk alleles will have a risk score of 0. We did not weight the PRS by effect sizes because they were available only for European samples and were not readily transferable to populations of different ethnicities [38]. On the other hand, we assumed that the genetic associations from most GWAS-identified variants can be replicated in populations of non-European ancestries [39]. The population-specific PRS (psPRS) was computed as the mean of the individual PRSs.

## Results

### Population allele frequencies

Two loci, rs13393316 and rs2922921, were fixed at the non-risk allele in the EAS superpopulation. All other loci were polymorphic in all superpopulations. However, several loci were monomorphic in some subpopulations (S1 Table). Furthermore, 12 loci manifested significant departure from HWE in the superpopulations and 25 – at the level of subpopulations (S1 Table).

**Fig 1:**
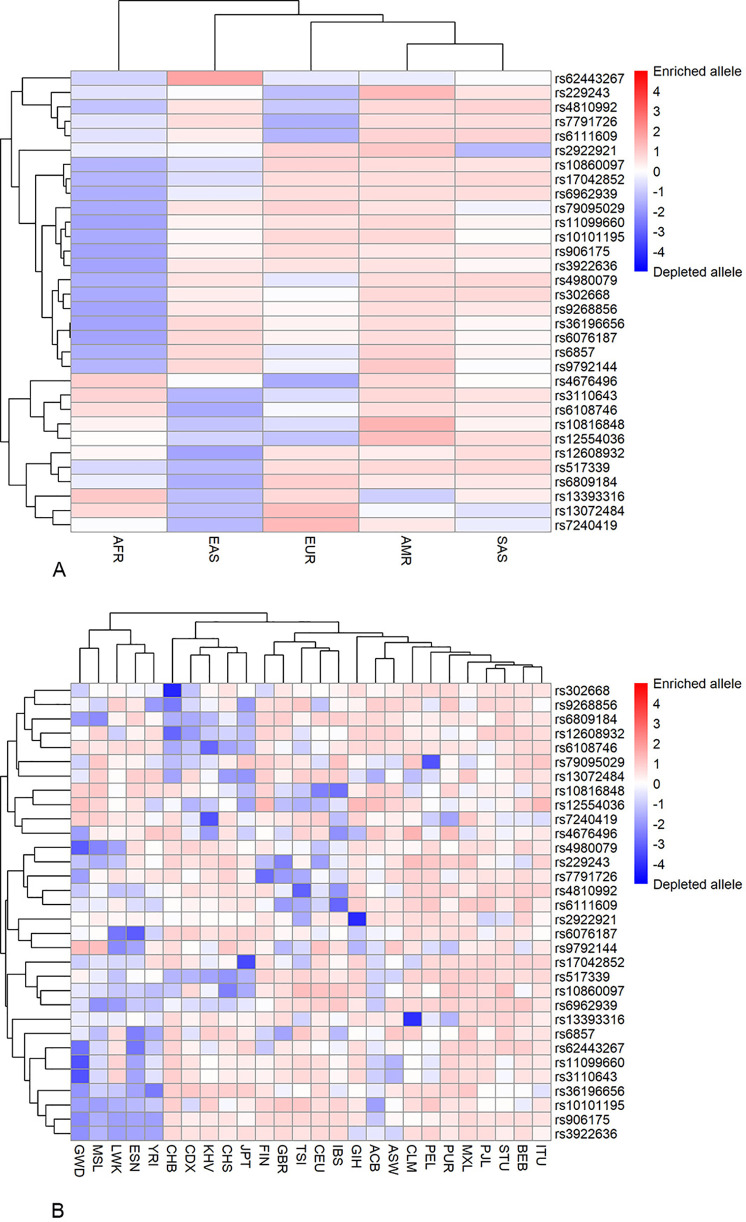
A heatmap of the risk allele frequencies in the studied ethnic populations. The color scale represents the depletion (blue) and enrichment (red) of the allele as compared to the average allele frequency in the whole sample (white). A – ethnic superpopulations; B – ethnic subpopulations. For the population designations, see Table 2.

The heatmaps of allelic frequency enrichment/depletion are shown in Fig 1. Several loci manifested contrasting patterns of allele frequencies in the ethnic superpopulations. Specifically, in the AFR superpopulation, frequencies of 15 loci were significantly depleted while being enriched in all other superpopulations. Likewise, the other 11 loci were depleted in EAS superpopulation and enriched in most other superpopulations (Fig 1A). Interestingly, the allele enrichment/depletion patterns manifested the highest similarity in the two geographically most distant superpopulations, AMR and SAS. The majority of alleles were enriched in these superpopulations. The clustering of subpopulations was generally concordant with the respective superpopulations (Fig 1B). There were several exceptions, though. Specifically, two subpopulations of African ancestry, Africans in Southwest US (ASW) and African Caribbean in Barbados (ACB), and Gujarati Indian in Houston, TX (GIH), manifested the enrichment/depletion patterns more similar to those of the AMR and EUR superpopulations (Fig 1B).

Frequencies of FTD risk alleles at many loci manifested multifold differences in the superpopulations (S2 Table). For example, the frequency of the risk allele T of the locus rs906175 mapped to the intron of the *CEP131* gene is more than 22-fold lower in the AFR superpopulation than in any other. The risk allele rs3110643-C located in the intron of the *HNF1 homeobox B* gene manifests even more extreme differences: its frequency (0.001) in the EAS population is more than 50 times lower than in the SAS superpopulation and 174 times lower than in the EUR superpopulation (S2 Table).

Likewise, significant differences in frequencies of several risk alleles were observed among subpopulations of the same ethnicities. For example, the above-mentioned allele rs906175-T occurred only in two American subpopulations of African ancestry (ASW and ACB) but was absent in the other African subpopulations. More than tenfold differences in allele frequencies were observed for rs17042852-C in the European subpopulations (S2 Table).

### Intra- and interpopulation genetic diversity

The values of the within-population diversity parameters for the five superpopulations are given in Table 3. The highest diversity was observed in EUR, whereas the lowest was in EAS. The subpopulations of the same ethnicity manifested similar levels of diversity (S3 Table).

**Table 3:** The parameters of genetic variation for the ethnic superpopulations at the FTD candidate loci.

Populations	*H*_o_	*H*_e_	*I*	*F*
EUR	0.326 ± 0.028	0.324 ± 0.027	0.488 ± 0.034	0.002 ± 0.009
AFR	0.252 ± 0.031	0.253 ± 0.031	0.393 ± 0.041	0.004 ± 0.006
AMR	0.284 ± 0.027	0.294 ± 0.028	0.451 ± 0.036	0.037 ± 0.009
EAS	0.248 ± 0.030	0.248 ± 0.030	0.386 ± 0.041	−0.002 ± 0.006
SAS	0.282 ± 0.027	0.283 ± 0.027	0.439 ± 0.035	0.003 ± 0.008

*H*_o_, observed heterozygosity; *H*_e_, expected heterozygosity; *I*, Shannon’s information index; *F*, fixation index.

**Fig 2:**
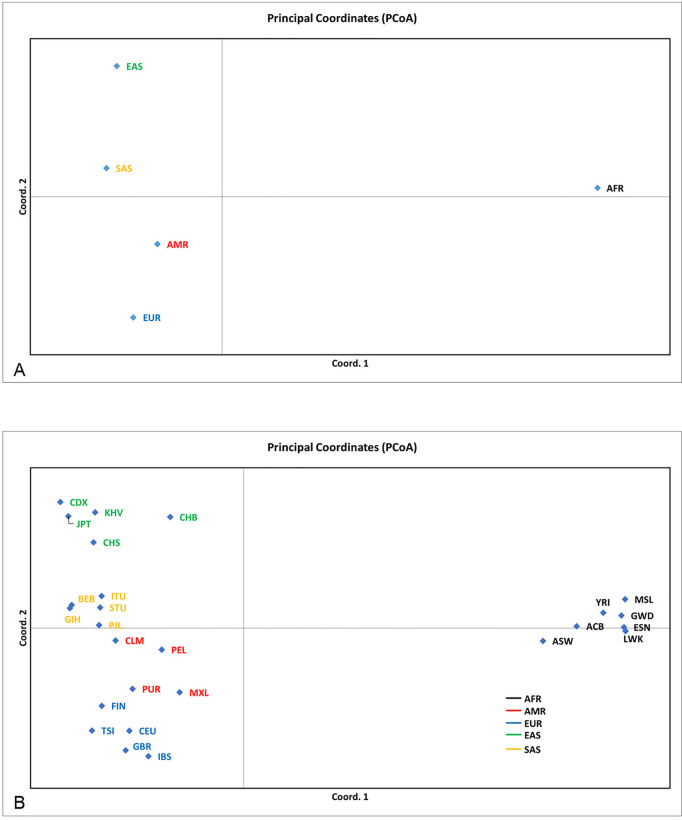
Principal Coordinate Analysis of the ethnic super- and subpopulations. A – ethnic superpopulations; B – ethnic subpopulations. For the population designations, see Table 2.

The studied populations manifested significant differentiation at the analyzed loci. The Principal Coordinate Analysis grouped the ethnic subpopulations into well-defined clusters corresponding to the five superpopulations (Fig 2). Among the latter, EAS and AFR were the most distinct from the others. These two also possessed the lowest genetic diversity (Table 3).

The pairwise *F*_ST_ and Nei’s genetic distances between the superpopulations and subpopulations are shown in Table 4 and S4 Table, respectively. Nearly all subpopulations were significantly differentiated across all loci.

**Table 4:** A matrix of the pairwise Nei’s genetic distances and *F*_ST_ values between the ethnic superpopulations.

	EUR	AFR	AMR	EAS	SAS
EUR		0.052	0.009	0.033	0.016
AFR	0.113		0.043	0.054	0.050
AMR	0.019	0.102		0.020	0.012
EAS	0.077	0.134	0.051		0.012
SAS	0.036	0.118	0.027	0.032	

The *F*_ST_ values are given below the diagonal, the Nei’s genetic distances are given above the diagonal. All *F*_ST_ values are significant at *P* < 0.01.

The global *F*_ST_ values at individual loci varied significantly among the superpopulations, from 0.004 at rs9792144 to 0.297 at rs906175 (S5a Table). The variation at the subpopulation level was lower, ranging from 0.012 (rs4676496) to 0.262 (rs906175) (S5b Table). All *F*_ST_ values were significant at *P* < 0.01.

The pairwise exact G-test for genic and genotypic differentiation across all studied loci yielded significant values (*P* << 10^−6^) for all pairs of the superpopulations. A similar level of significance was observed for the exact *G*-test at individual loci: only a few pairwise superpopulation comparisons were non-significant (S1 Appendix).

Differentiation of most ethnic subpopulations generally corresponded to that between the superpopulations. Furthermore, most subpopulations were significantly differentiated even within the same ethnicity. There were several exceptions, though. In particular, all South-Asian subpopulations, except GIH, did not manifest significant differentiation (S4 Table). Likewise, the G-test yielded non-significant values for the ACW-ACB, CEU-GBR, and CDX-KHV pairs (S2 Appendix).

### Composite genetic scores for the FTD risk loci

**Fig 3:**
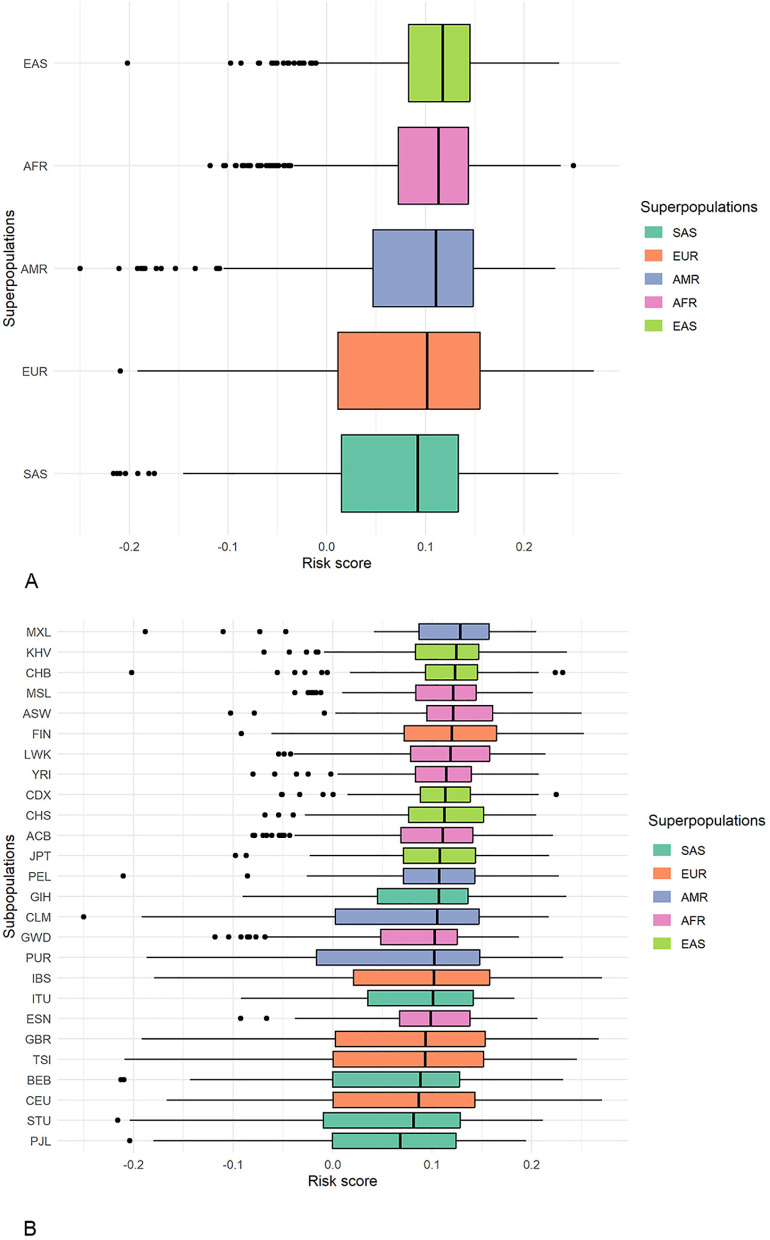
Box plot diagram of population-specific composite genetic risk scores for FTD. A – ethnic superpopulations; B – ethnic subpopulations. The center line of the box plot represents the median, the edges of the box indicate the 25th and 75th percentiles; the whiskers extend 1.5 times the interquartile range from these percentiles, and circles denote outliers. For the population designations, see Table 2.

The mean unweighted composite genetic risk score of FTD across all studied populations was quite low, 0.091 ± 0.078. The lowest was in South Asians (0.072 ± 0.082) and the highest in East Asians (0.109 ± 0.058, Fig 3A). The more pronounced differences were observed between the subpopulations: the lowest and the highest psPRSs differed nearly twofold (Fig 3B).

The unweighted psPRS significantly underestimates the true (weighted) psPRS that accounts for the risk allele effect size. For example, the unweighted psPRS for the EUR superpopulation was 0.083, whereas the weighted psPRS was 0.563.

The pairwise comparison of the unweighted psPRS across the superpopulations using Tukey’s test showed that six out of ten pairs differed significantly (Table 5). The largest differences were observed between the Asian superpopulations, EAS and SAS. Within-ethnic differences of psPRS were much less pronounced: only two pairs of subpopulations of the same ethnicity, FIN–CEU and FIN–TSI, manifested significant differences (S6 Table).

**Table 5:** The pairwise *P* values matrix of the Tukey’s t-test for psPRS differences between the five superpopulations.

	AFR	AMR	EAS	EUR	SAS
AFR					
AMR	0.039				
EAS	0.256	< 0.001			
EUR	0.001	0.980	< 0.001		
SAS	< 0.001	0.075	< 0.001	0.172	

## Discussion

In this study, we analyzed the genetic structure and differentiation of ethnic populations at the FTD risk loci. These findings may have important implications for confirming the genetic factors underlying variations in FTD clinical manifestations within populations and identifying genes contributing to FTD’s different prevalence across major ethnic groups.

FTD has a strong genetic component, particularly significant for fFTD [13]. A family study of the Italian cohort reported heritability from 75.7% to 98.5% for early-onset and late-onset FTD [40]. A significant portion of fFTD is caused by autosomal dominant mutations in a few major genes, primarily *C9orf72*, *MAPT* (*tau*), and *GRN* (*progranulin*). These mutations account for a substantial part of the genetic risk in affected families and are estimated to be responsible for roughly 10–25% of all FTD cases [41].

However, many fFTD cases lack mutations in the known major genes [42]. This phenomenon is known as a problem of “missing heritability”. This suggests that other genetic factors, potentially including numerous common variants with small additive effects (polygenic risk) or rarer variants, contribute to FTD risk and thus to its narrow-sense heritability.

The genetics of sFTD (around 60–70% of cases) is still poorly understood. Heritability of sFTD is likely lower than that of fFTD, but its accurate estimates are not available.

The common disease-common variant (CDCV) hypothesis suggests that the genetic risk for widespread multifactorial diseases often involves variants that are common (i.e., frequency ≥ 5%) across various human populations [43,44]. However, this view contrasts with the well-documented disparities in the prevalence of many complex disorders between ethnic groups (e.g., [26,45,46]). Traditionally, these inequalities have largely been attributed to differences in socioeconomic status and environmental factors among these groups [47,48]. However, there is growing evidence that the above disparities may be due to the differences in genetic structure of ethnicities [19,20,49]. This assumption seems particularly reasonable for FTD, which, as mentioned above, has a very strong genetic component.

The present study showed significant differentiation of ethnic populations at the FTD risk loci. Importantly, all five major ethnic groups manifested such differentiation at the level of subpopulations, too (S4 Table, S2 Appendix). The data about the genetic structure of ethnic populations at FTD candidate loci are limited and concern mainly the variants of the major-effect genes [50,51]. However, even these scarce data suggest that ethnicities likely differ by genetic structure at the FTD loci. For example, the *C9orf72* repeat expansion, a common genetic cause of fFTD, appears to be more prevalent in populations of European descent compared to Asian populations [52].

The unweighted psPRS values obtained in this study are significantly lower than the weighted psPRSs computed with the effect sizes. However, the effect sizes for FTD risk loci are unavailable for all ethnic populations, except Europeans. Based on the comparison of the unweighted and weighted psPRSs for Europeans (0.083 and 0.563), we can assume that the weighted PRS values for non-Europeans probably range within 0.5–0.7. Indeed, most unweighted psPRSs for the subpopulations do not differ significantly (S6 Table). Furthermore, recent studies reported transferability of the Alzheimer’s disease PRS for Europeans to other ethnic populations [53,54]. Given the growing evidence that Alzheimer’s disease may share a genetic basis with FTD [17,55,56], the above assumption seems reasonable.

The population-wise genetic risk scores (Fig 3) may suggest that the predicted prevalence of FTD is lowest in South Asians and highest in East Asians. However, these results should be interpreted with caution. The data about the prevalence of FTD in different populations are quite variable and inconsistent (see, for example, [1,57]). This variability stems from a combination of factors, including the evolving nature of diagnostic criteria, the inherent challenges in clinically recognizing FTD subtypes, methodological differences in epidemiological studies, and varying levels of awareness among healthcare professionals and the general public. Furthermore, PRS may not necessarily correlate with the disease prevalence [54]. Given the limited and inconsistent data on the FTD prevalence, the bias can be quite significant.

There is growing evidence that the genetic structure of populations is one of the significant factors explaining the different prevalence of complex diseases (e.g., [20,21,49,58,59]). While these genetic differences may potentially contribute to variation in disease prevalence, the relationship is likely complex. The discrepancy between our findings and some epidemiological data might result from several factors: the limitations of unweighted PRS without population-specific effect sizes, the scarcity of standardized epidemiological studies of FTD across diverse populations, and the complex interplay between genetic architecture and environmental factors that affect disease diagnosis and reporting.

Understanding the genetics of FTD is crucial for improving diagnosis, developing targeted therapies, and providing appropriate support for patients. The identification of causative genetic variants for FTD is important for both clinical practice and research in the field of neurodegenerative disorders. From a diagnostic perspective, these discoveries may lead to the development of more comprehensive genetic testing panels for FTD, potentially increasing the likelihood of identifying the underlying genetic cause in affected individuals. Moreover, the growing understanding of genotype-phenotype correlations enhances clinicians’ ability to predict disease course and symptoms based on specific genetic mutations, thereby improving patient care and management strategies.

From a molecular biology standpoint, the identified genetic variants provide valuable insights into the key cellular processes involved in neurodegeneration. Furthermore, each newly identified candidate gene represents a potential target for therapeutic development, expanding the range of possible treatment strategies for FTD. Importantly, the involvement of some of these genes in both FTD and amyotrophic lateral sclerosis has highlighted the biological connections between these conditions, suggesting potential similarities in disease mechanisms and treatment approaches [56,60].

This study has several limitations, which should be acknowledged. First, the sample sizes of subpopulations were small, which might result in reduced statistical power. Second, the set of the analyzed FTD risk loci was limited only to those with the identified risk alleles (32 loci out of 55 listed in the GWAS Catalog). Third, only common alleles were analyzed, whereas the major effect genes (e.g., *MAPT*, *GRN*, and *C9orf72*) were not included in the analysis.

## Conclusions

Our analysis of genetic structure across different ethnic populations at FTD risk loci revealed significant differentiation at both super- and subpopulation levels. The distinct patterns of risk allele enrichment/depletion observed among ethnic groups, along with the significant differences in population-specific polygenic risk scores, suggest that population genetic architecture may contribute substantially to the variation in FTD prevalence.

These findings have important implications for understanding ethnic disparities in disease burden and may guide more targeted approaches for screening and intervention in high-risk populations. However, the limited epidemiological data available across diverse populations, challenges in diagnosis, and variable methodologies in prevalence studies necessitate further research.

Future studies should focus on expanding genetic analyses across diverse populations, integrating environmental and socioeconomic factors, and developing population-specific risk assessment tools for efficient FTD diagnosis, control, and management.

## Supporting information

S1 Table Summary of Chi-Square Tests for Hardy-Weinberg Equilibrium in ethnic superpopulations (a) and subpopulations (b). (XLSX)

S2 Table Allele frequencies with graphs by population and locus. (a), superpopulations; (b), subpopulations.
(XLSX)

S3 Table Parameters of genetic diversity of the ethnic subpopulations. (XLSX)

S4 Table A matrix of the pairwise Nei’s genetic distances and *F*_*ST*_ values between ethnic subpopulations. (XLSX)

S5 Table Summary of the analysis of *F*-statistics by locus in ethnic superpopulations (a) and subpopulations (b). (XLSX)

S6 Table The pairwise *P* values matrix of the Tukey’s t-test for psPRS differences between the ethnic subpopulations. (XLSX)

S1 Appendix Exact G-test for genic and genotypic differentiation of the superpopulations. (DOCX)

S2 Appendix Exact G-test for genic and genotypic differentiation of the subpopulations. (DOCX)
