# Supplementary material for: Genetic structure of different ethnic populations at the frontotemporal dementia risk loci
Source: PLoS One. 2025 Aug 5;20(8):e0329809. doi: 10.1371/journal.pone.0329809 (PMC12324132; doi:10.1371/journal.pone.0329809)
Supplement: S1 Appendix — (DOCX) [file pone.0329809.s007.docx]

**Genic differentiation for each ethnic superpopulation pair (exact G test)**

**Allele codes: A – 1, C – 2, G – 3, T - 4**

Markov chain parameters

Dememorisation : 10000

Batches : 100

Iterations per batch : 5000

Locus: rs12608932

=================================

Pop Alleles:

-----------------------------------------------------------

1 2 Total

AFR 886 436 1322

AMR 483 211 694

EAS 306 702 1008

EUR 655 351 1006

SAS 505 473 978

Total: 28352173 5008

Locus Population pair P-Value S.E.

----------- --------------------- ------- -------

rs12608932 AMR & AFR 0.24742 0.00751

rs12608932 EAS & AFR 0.00000 0.00000

rs12608932 EAS & AMR 0.00000 0.00000

rs12608932 EUR & AFR 0.33848 0.00935

rs12608932 EUR & AMR 0.05889 0.00390

rs12608932 EUR & EAS 0.00000 0.00000

rs12608932 SAS & AFR 0.00000 0.00000

rs12608932 SAS & AMR 0.00000 0.00000

rs12608932 SAS & EAS 0.00000 0.00000

rs12608932 SAS & EUR 0.00000 0.00000

Locus: rs302668

=================================

Pop Alleles:

-----------------------------------------------------------

2 4 Total

AFR 135 1187 1322

AMR 190 504 694

EAS 321 687 1008

EUR 351 655 1006

SAS 216 762 978

Total: 12133795 5008

Locus Population pair P-Value S.E.

----------- --------------------- ------- -------

rs302668 AMR & AFR 0.00000 0.00000

rs302668 EAS & AFR 0.00000 0.00000

rs302668 EAS & AMR 0.05245 0.00289

rs302668 EUR & AFR 0.00000 0.00000

rs302668 EUR & AMR 0.00154 0.00038

rs302668 EUR & EAS 0.15191 0.00643

rs302668 SAS & AFR 0.00000 0.00000

rs302668 SAS & AMR 0.01248 0.00137

rs302668 SAS & EAS 0.00001 0.00001

rs302668 SAS & EUR 0.00000 0.00000

Locus: rs9268856

=================================

Pop Alleles:

-----------------------------------------------------------

1 2 Total

AFR 505 817 1322

AMR 192 502 694

EAS 263 745 1008

EUR 247 759 1006

SAS 254 724 978

Total: 14613547 5008

Locus Population pair P-Value S.E.

----------- --------------------- ------- -------

rs9268856 AMR & AFR 0.00007 0.00007

rs9268856 EAS & AFR 0.00000 0.00000

rs9268856 EAS & AMR 0.50160 0.00715

rs9268856 EUR & AFR 0.00000 0.00000

rs9268856 EUR & AMR 0.14913 0.00506

rs9268856 EUR & EAS 0.43640 0.00766

rs9268856 SAS & AFR 0.00000 0.00000

rs9268856 SAS & AMR 0.46966 0.00667

rs9268856 SAS & EAS 0.95945 0.00079

rs9268856 SAS & EUR 0.48936 0.00760

Locus: rs906175

=================================

Pop Alleles:

-----------------------------------------------------------

2 4 Total

AFR 129527 1322

AMR 270 424 694

EAS 373 635 1008

EUR 557 449 1006

SAS 409 569 978

Total: 29042104 5008

Locus Population pair P-Value S.E.

----------- --------------------- ------- -------

rs906175 AMR & AFR 0.00000 0.00000

rs906175 EAS & AFR 0.00000 0.00000

rs906175 EAS & AMR 0.45498 0.00833

rs906175 EUR & AFR 0.00000 0.00000

rs906175 EUR & AMR 0.00000 0.00000

rs906175 EUR & EAS 0.00000 0.00000

rs906175 SAS & AFR 0.00000 0.00000

rs906175 SAS & AMR 0.23919 0.00642

rs906175 SAS & EAS 0.03098 0.00305

rs906175 SAS & EUR 0.00000 0.00000

Locus: rs17042852

=================================

Pop Alleles:

-----------------------------------------------------------

2 4 Total

AFR 5 1317 1322

AMR 16 678 694

EAS 77 931 1008

EUR 46 960 1006

SAS 40 938 978

Total: 184 4824 5008

Locus Population pair P-Value S.E.

----------- --------------------- ------- -------

rs17042852 AMR & AFR 0.00023 0.00004

rs17042852 EAS & AFR 0.00000 0.00000

rs17042852 EAS & AMR 0.00002 0.00002

rs17042852 EUR & AFR 0.00000 0.00000

rs17042852 EUR & AMR 0.01640 0.00077

rs17042852 EUR & EAS 0.00477 0.00052

rs17042852 SAS & AFR 0.00000 0.00000

rs17042852 SAS & AMR 0.05497 0.00147

rs17042852 SAS & EAS 0.00113 0.00025

rs17042852 SAS & EUR 0.65757 0.00289

Locus: rs11099660

=================================

Pop Alleles:

-----------------------------------------------------------

2 4 Total

AFR 705 617 1322

AMR 499 195 694

EAS 794 214 1008

EUR 750 256 1006

SAS 767 211 978

Total: 35151493 5008

Locus Population pair P-Value S.E.

----------- --------------------- ------- -------

rs11099660 AMR & AFR 0.00000 0.00000

rs11099660 EAS & AFR 0.00000 0.00000

rs11099660 EAS & AMR 0.00114 0.00042

rs11099660 EUR & AFR 0.00000 0.00000

rs11099660 EUR & AMR 0.22594 0.00626

rs11099660 EUR & EAS 0.03051 0.00269

rs11099660 SAS & AFR 0.00000 0.00000

rs11099660 SAS & AMR 0.00275 0.00056

rs11099660 SAS & EAS 0.86771 0.00258

rs11099660 SAS & EUR 0.04640 0.00341

Locus: rs4980079

=================================

Pop Alleles:

-----------------------------------------------------------

2 4 Total

AFR 1140182 1322

AMR 462 232 694

EAS 664 344 1008

EUR 586 420 1006

SAS 735 243 978

Total: 35871421 5008

Locus Population pair P-Value S.E.

----------- --------------------- ------- -------

rs4980079 AMR & AFR 0.00000 0.00000

rs4980079 EAS & AFR 0.00000 0.00000

rs4980079 EAS & AMR 0.79189 0.00376

rs4980079 EUR & AFR 0.00000 0.00000

rs4980079 EUR & AMR 0.00046 0.00024

rs4980079 EUR & EAS 0.00055 0.00024

rs4980079 SAS & AFR 0.00000 0.00000

rs4980079 SAS & AMR 0.00026 0.00019

rs4980079 SAS & EAS 0.00003 0.00002

rs4980079 SAS & EUR 0.00000 0.00000

Locus: rs6857

=================================

Pop Alleles:

-----------------------------------------------------------

2 4 Total

AFR 125270 1322

AMR 621 73 694

EAS 907 101 1008

EUR 840 166 1006

SAS 835 143 978

Total: 4455553 5008

Locus Population pair P-Value S.E.

----------- --------------------- ------- -------

rs6857 AMR & AFR 0.00004 0.00003

rs6857 EAS & AFR 0.00000 0.00000

rs6857 EAS & AMR 0.74349 0.00334

rs6857 EUR & AFR 0.00000 0.00000

rs6857 EUR & AMR 0.00048 0.00017

rs6857 EUR & EAS 0.00001 0.00001

rs6857 SAS & AFR 0.00000 0.00000

rs6857 SAS & AMR 0.01371 0.00116

rs6857 SAS & EAS 0.00199 0.00049

rs6857 SAS & EUR 0.26565 0.00613

Locus: rs3110643

=================================

Pop Alleles:

-----------------------------------------------------------

2 4 Total

AFR 112 1210 1322

AMR 74 620 694

EAS 1 1007 1008

EUR 175 831 1006

SAS 52 926 978

Total: 414 4594 5008

Locus Population pair P-Value S.E.

----------- --------------------- ------- -------

rs3110643 AMR & AFR 0.12544 0.00326

rs3110643 EAS & AFR 0.00000 0.00000

rs3110643 EAS & AMR 0.00000 0.00000

rs3110643 EUR & AFR 0.00000 0.00000

rs3110643 EUR & AMR 0.00012 0.00006

rs3110643 EUR & EAS 0.00000 0.00000

rs3110643 SAS & AFR 0.00522 0.00070

rs3110643 SAS & AMR 0.00002 0.00001

rs3110643 SAS & EAS 0.00000 0.00000

rs3110643 SAS & EUR 0.00000 0.00000

Locus: rs10816848

=================================

Pop Alleles:

--------------------------------------------------------------

1 4 Total

AFR 545 777 1322

AMR 238 456 694

EAS 234 774 1008

EUR 455 551 1006

SAS 262 716 978

Total: 17343274 5008

Locus Population pair P-Value S.E.

----------- --------------------- ------- -------

rs10816848 AMR & AFR 0.00331 0.00094

rs10816848 EAS & AFR 0.00000 0.00000

rs10816848 EAS & AMR 0.00010 0.00010

rs10816848 EUR & AFR 0.05086 0.00443

rs10816848 EUR & AMR 0.00000 0.00000

rs10816848 EUR & EAS 0.00000 0.00000

rs10816848 SAS & AFR 0.00000 0.00000

rs10816848 SAS & AMR 0.00118 0.00035

rs10816848 SAS & EAS 0.06140 0.00399

rs10816848 SAS & EUR 0.00000 0.00000

Locus: rs79095029

=================================

Pop Alleles:

--------------------------------------------------------------

2 3 Total

AFR 1196126 1322

AMR 669 25 694

EAS 966 42 1008

EUR 954 52 1006

SAS 954 24 978

Total: 4739269 5008

Locus Population pair P-Value S.E.

----------- --------------------- ------- -------

rs79095029 AMR & AFR 0.00000 0.00000

rs79095029 EAS & AFR 0.00000 0.00000

rs79095029 EAS & AMR 0.61191 0.00231

rs79095029 EUR & AFR 0.00005 0.00005

rs79095029 EUR & AMR 0.12505 0.00232

rs79095029 EUR & EAS 0.29216 0.00396

rs79095029 SAS & AFR 0.00000 0.00000

rs79095029 SAS & AMR 0.19348 0.00255

rs79095029 SAS & EAS 0.03271 0.00134

rs79095029 SAS & EUR 0.00252 0.00031

Locus: rs13393316

=================================

Pop Alleles:

--------------------------------------------------------------

1 3 Total

AFR 1189133 1322

AMR 501 193 694

EAS 10080 1008

EUR 856 150 1006

SAS 941 37 978

Total: 4495513 5008

Locus Population pair P-Value S.E.

----------- --------------------- ------- -------

rs13393316 AMR & AFR 0.00000 0.00000

rs13393316 EAS & AFR 0.00000 0.00000

rs13393316 EAS & AMR 0.00000 0.00000

rs13393316 EUR & AFR 0.00030 0.00009

rs13393316 EUR & AMR 0.00000 0.00000

rs13393316 EUR & EAS 0.00000 0.00000

rs13393316 SAS & AFR 0.00000 0.00000

rs13393316 SAS & AMR 0.00000 0.00000

rs13393316 SAS & EAS 0.00000 0.00000

rs13393316 SAS & EUR 0.00000 0.00000

Locus: rs7791726

=================================

Pop Alleles:

--------------------------------------------------------------

2 3 Total

AFR 977 345 1322

AMR 400 294 694

EAS 655 353 1008

EUR 410 596 1006

SAS 630 348 978

Total: 30721936 5008

Locus Population pair P-Value S.E.

----------- --------------------- ------- -------

rs7791726 AMR & AFR 0.00000 0.00000

rs7791726 EAS & AFR 0.00000 0.00000

rs7791726 EAS & AMR 0.00316 0.00082

rs7791726 EUR & AFR 0.00000 0.00000

rs7791726 EUR & AMR 0.00000 0.00000

rs7791726 EUR & EAS 0.00000 0.00000

rs7791726 SAS & AFR 0.00000 0.00000

rs7791726 SAS & AMR 0.00583 0.00111

rs7791726 SAS & EAS 0.81566 0.00367

rs7791726 SAS & EUR 0.00000 0.00000

Locus: rs10101195

=================================

Pop Alleles:

--------------------------------------------------------------

1 2 Total

AFR 694 628 1322

AMR 187 507 694

EAS 168 840 1008

EUR 272 734 1006

SAS 147 831 978

Total: 14683540 5008

Locus Population pair P-Value S.E.

----------- --------------------- ------- -------

rs10101195 AMR & AFR 0.00000 0.00000

rs10101195 EAS & AFR 0.00000 0.00000

rs10101195 EAS & AMR 0.00000 0.00000

rs10101195 EUR & AFR 0.00000 0.00000

rs10101195 EUR & AMR 1.00000 0.00000

rs10101195 EUR & EAS 0.00000 0.00000

rs10101195 SAS & AFR 0.00000 0.00000

rs10101195 SAS & AMR 0.00000 0.00000

rs10101195 SAS & EAS 0.32152 0.00619

rs10101195 SAS & EUR 0.00000 0.00000

Locus: rs36196656

=================================

Pop Alleles:

--------------------------------------------------------------

1 2 Total

AFR 1083239 1322

AMR 309 385 694

EAS 600 408 1008

EUR 395 611 1006

SAS 367 611 978

Total: 27542254 5008

Locus Population pair P-Value S.E.

----------- --------------------- ------- -------

rs36196656 AMR & AFR 0.00000 0.00000

rs36196656 EAS & AFR 0.00000 0.00000

rs36196656 EAS & AMR 0.00000 0.00000

rs36196656 EUR & AFR 0.00000 0.00000

rs36196656 EUR & AMR 0.03236 0.00326

rs36196656 EUR & EAS 0.00000 0.00000

rs36196656 SAS & AFR 0.00000 0.00000

rs36196656 SAS & AMR 0.00463 0.00094

rs36196656 SAS & EAS 0.00000 0.00000

rs36196656 SAS & EUR 0.42754 0.00888

Locus: rs13072484

=================================

Pop Alleles:

----------------------------------------

1 3 Total

AFR 330 992 1322

AMR 92 602 694

EAS 129 879 1008

EUR 232 774 1006

SAS 287 691 978

Total: 10703938 5008

Locus Population pair P-Value S.E.

----------- --------------------- ------- -------

rs13072484 AMR & AFR 0.00000 0.00000

rs13072484 EAS & AFR 0.00000 0.00000

rs13072484 EAS & AMR 0.82698 0.00247

rs13072484 EUR & AFR 0.31429 0.00783

rs13072484 EUR & AMR 0.00000 0.00000

rs13072484 EUR & EAS 0.00000 0.00000

rs13072484 SAS & AFR 0.02009 0.00238

rs13072484 SAS & AMR 0.00000 0.00000

rs13072484 SAS & EAS 0.00000 0.00000

rs13072484 SAS & EUR 0.00102 0.00033

Locus: rs62443267

=================================

Pop Alleles:

-----------------------------------------------------------

2 4 Total

AFR 1187135 1322

AMR 517 177 694

EAS 839 169 1008

EUR 767 239 1006

SAS 878 100 978

Total: 4188820 5008

Locus Population pair P-Value S.E.

----------- --------------------- ------- -------

rs62443267 AMR & AFR 0.00000 0.00000

rs62443267 EAS & AFR 0.00000 0.00000

rs62443267 EAS & AMR 0.00000 0.00000

rs62443267 EUR & AFR 0.00000 0.00000

rs62443267 EUR & AMR 0.43216 0.00792

rs62443267 EUR & EAS 0.00007 0.00005

rs62443267 SAS & AFR 1.00000 0.00000

rs62443267 SAS & AMR 0.00000 0.00000

rs62443267 SAS & EAS 0.00001 0.00001

rs62443267 SAS & EUR 0.00000 0.00000

Locus: rs10860097

=================================

Pop Alleles:

-----------------------------------------------------------

1 4 Total

AFR 13211 1322

AMR 683 11 694

EAS 925 83 1008

EUR 979 27 1006

SAS 922 56 978

Total: 4830178 5008

Locus Population pair P-Value S.E.

----------- --------------------- ------- -------

rs10860097 AMR & AFR 0.00006 0.00002

rs10860097 EAS & AFR 0.00000 0.00000

rs10860097 EAS & AMR 0.00000 0.00000

rs10860097 EUR & AFR 0.00000 0.00000

rs10860097 EUR & AMR 0.13874 0.00210

rs10860097 EUR & EAS 0.00000 0.00000

rs10860097 SAS & AFR 0.00000 0.00000

rs10860097 SAS & AMR 0.00001 0.00001

rs10860097 SAS & EAS 0.03317 0.00189

rs10860097 SAS & EUR 0.00090 0.00019

Locus: rs7240419

=================================

Pop Alleles:

-----------------------------------------------------------

1 3 Total

AFR 304 1018 1322

AMR 142 552 694

EAS 453 555 1008

EUR 258 748 1006

SAS 421 557 978

Total: 15783430 5008

Locus Population pair P-Value S.E.

----------- --------------------- ------- -------

rs7240419 AMR & AFR 0.18672 0.00562

rs7240419 EAS & AFR 0.00000 0.00000

rs7240419 EAS & AMR 0.00000 0.00000

rs7240419 EUR & AFR 0.13975 0.00678

rs7240419 EUR & AMR 0.01585 0.00153

rs7240419 EUR & EAS 0.00000 0.00000

rs7240419 SAS & AFR 0.00000 0.00000

rs7240419 SAS & AMR 0.00000 0.00000

rs7240419 SAS & EAS 0.43044 0.00845

rs7240419 SAS & EUR 0.00000 0.00000

Locus: rs6076187

=================================

Pop Alleles:

-----------------------------------------------------------

1 3 Total

AFR 376 946 1322

AMR 47 647 694

EAS 107 901 1008

EUR 45 961 1006

SAS 39 939 978

Total: 614 4394 5008

Locus Population pair P-Value S.E.

----------- --------------------- ------- -------

rs6076187 AMR & AFR 0.00000 0.00000

rs6076187 EAS & AFR 0.00000 0.00000

rs6076187 EAS & AMR 0.00784 0.00067

rs6076187 EUR & AFR 0.00000 0.00000

rs6076187 EUR & AMR 0.04780 0.00167

rs6076187 EUR & EAS 0.00000 0.00000

rs6076187 SAS & AFR 0.00000 0.00000

rs6076187 SAS & AMR 0.01198 0.00081

rs6076187 SAS & EAS 0.00000 0.00000

rs6076187 SAS & EUR 0.65617 0.00297

Locus: rs4676496

=================================

Pop Alleles:

-----------------------------------------------------------

1 3 Total

AFR 806 516 1322

AMR 400 294 694

EAS 659 349 1008

EUR 523 483 1006

SAS 639 339 978

Total: 30271981 5008

Locus Population pair P-Value S.E.

----------- --------------------- ------- -------

rs4676496 AMR & AFR 0.14615 0.00632

rs4676496 EAS & AFR 0.03099 0.00280

rs4676496 EAS & AMR 0.00097 0.00027

rs4676496 EUR & AFR 0.00002 0.00002

rs4676496 EUR & AMR 0.02364 0.00272

rs4676496 EUR & EAS 0.00000 0.00000

rs4676496 SAS & AFR 0.03739 0.00385

rs4676496 SAS & AMR 0.00099 0.00034

rs4676496 SAS & EAS 1.00000 0.00000

rs4676496 SAS & EUR 0.00000 0.00000

Locus: rs517339

=================================

Pop Alleles:

-----------------------------------------------------------

2 4 Total

AFR 640 682 1322

AMR 201 493 694

EAS 115 893 1008

EUR 389 617 1006

SAS 274 704 978

Total: 16193389 5008

Locus Population pair P-Value S.E.

----------- --------------------- ------- -------

rs517339 AMR & AFR 0.00000 0.00000

rs517339 EAS & AFR 0.00000 0.00000

rs517339 EAS & AMR 0.00000 0.00000

rs517339 EUR & AFR 0.00000 0.00000

rs517339 EUR & AMR 0.00013 0.00011

rs517339 EUR & EAS 0.00000 0.00000

rs517339 SAS & AFR 0.00000 0.00000

rs517339 SAS & AMR 0.69547 0.00503

rs517339 SAS & EAS 0.00000 0.00000

rs517339 SAS & EUR 0.00000 0.00000

Locus: rs12554036

=================================

Pop Alleles:

----------

3 4 Total

AFR 1212110 1322

AMR 598 96 694

EAS 953 55 1008

EUR 775 231 1006

SAS 802 176 978

Total: 4340668 5008

Locus Population pair P-Value S.E.

----------- --------------------- ------- -------

rs12554036 AMR & AFR 0.00020 0.00008

rs12554036 EAS & AFR 0.00689 0.00077

rs12554036 EAS & AMR 0.00000 0.00000

rs12554036 EUR & AFR 0.00000 0.00000

rs12554036 EUR & AMR 0.00000 0.00000

rs12554036 EUR & EAS 0.00000 0.00000

rs12554036 SAS & AFR 0.00000 0.00000

rs12554036 SAS & AMR 0.02803 0.00187

rs12554036 SAS & EAS 0.00000 0.00000

rs12554036 SAS & EUR 0.00530 0.00073

Locus: rs4810992

=================================

Pop Alleles:

----------

1 3 Total

AFR 15 1307 1322

AMR 115 579 694

EAS 76 932 1008

EUR 322 684 1006

SAS 139 839 978

Total: 667 4341 5008

Locus Population pair P-Value S.E.

----------- --------------------- ------- -------

rs4810992 AMR & AFR 0.00000 0.00000

rs4810992 EAS & AFR 0.00000 0.00000

rs4810992 EAS & AMR 0.00000 0.00000

rs4810992 EUR & AFR 0.00000 0.00000

rs4810992 EUR & AMR 0.00000 0.00000

rs4810992 EUR & EAS 0.00000 0.00000

rs4810992 SAS & AFR 0.00000 0.00000

rs4810992 SAS & AMR 0.19565 0.00481

rs4810992 SAS & EAS 0.00000 0.00000

rs4810992 SAS & EUR 0.00000 0.00000

Locus: rs229243

=================================

Pop Alleles:

----------------------------------------

1 2 Total

AFR 854 468 1322

AMR 342 352 694

EAS 656 352 1008

EUR 351 655 1006

SAS 415 563 978

Total: 26182390 5008

Locus Population pair P-Value S.E.

----------- --------------------- ------- -------

rs229243 AMR & AFR 0.00000 0.00000

rs229243 EAS & AFR 0.82764 0.00406

rs229243 EAS & AMR 0.00000 0.00000

rs229243 EUR & AFR 0.00000 0.00000

rs229243 EUR & AMR 0.00000 0.00000

rs229243 EUR & EAS 0.00000 0.00000

rs229243 SAS & AFR 0.00000 0.00000

rs229243 SAS & AMR 0.00494 0.00088

rs229243 SAS & EAS 0.00000 0.00000

rs229243 SAS & EUR 0.00055 0.00023

Locus: rs6809184

=================================

Pop Alleles:

----------------------------------------

2 4 Total

AFR 1177145 1322

AMR 674 20 694

EAS 10017 1008

EUR 949 57 1006

SAS 881 97 978

Total: 4682326 5008

Locus Population pair P-Value S.E.

----------- --------------------- ------- -------

rs6809184 AMR & AFR 0.00000 0.00000

rs6809184 EAS & AFR 0.00000 0.00000

rs6809184 EAS & AMR 0.00042 0.00007

rs6809184 EUR & AFR 0.00000 0.00000

rs6809184 EUR & AMR 0.00636 0.00048

rs6809184 EUR & EAS 0.00000 0.00000

rs6809184 SAS & AFR 0.44486 0.00613

rs6809184 SAS & AMR 0.00000 0.00000

rs6809184 SAS & EAS 0.00000 0.00000

rs6809184 SAS & EUR 0.00059 0.00021

Locus: rs6962939

=================================

Pop Alleles:

----------------------------------------

1 4 Total

AFR 188 1134 1322

AMR 23 671 694

EAS 3 1005 1008

EUR 34 972 1006

SAS 36 942 978

Total: 284 4724 5008

Locus Population pair P-Value S.E.

----------- --------------------- ------- -------

rs6962939 AMR & AFR 0.00000 0.00000

rs6962939 EAS & AFR 0.00000 0.00000

rs6962939 EAS & AMR 0.00000 0.00000

rs6962939 EUR & AFR 0.00000 0.00000

rs6962939 EUR & AMR 1.00000 0.00000

rs6962939 EUR & EAS 0.00000 0.00000

rs6962939 SAS & AFR 0.00000 0.00000

rs6962939 SAS & AMR 0.78626 0.00148

rs6962939 SAS & EAS 0.00000 0.00000

rs6962939 SAS & EUR 0.80575 0.00164

Locus: rs9792144

=================================

Pop Alleles:

----------------------------------------

2 3 Total

AFR 1064258 1322

AMR 586 108 694

EAS 829 179 1008

EUR 867 139 1006

SAS 842 136 978

Total: 4188820 5008

Locus Population pair P-Value S.E.

----------- --------------------- ------- -------

rs9792144 AMR & AFR 0.02600 0.00190

rs9792144 EAS & AFR 0.27367 0.00621

rs9792144 EAS & AMR 0.23607 0.00564

rs9792144 EUR & AFR 0.00008 0.00006

rs9792144 EUR & AMR 0.32911 0.00603

rs9792144 EUR & EAS 0.01789 0.00146

rs9792144 SAS & AFR 0.00039 0.00016

rs9792144 SAS & AMR 0.35692 0.00589

rs9792144 SAS & EAS 0.02232 0.00194

rs9792144 SAS & EUR 1.00000 0.00000

Locus: rs6108746

=================================

Pop Alleles:

----------------------------------------

2 4 Total

AFR 166 1156 1322

AMR 98 596 694

EAS 38 970 1008

EUR 197 809 1006

SAS 157 821 978

Total: 656 4352 5008

Locus Population pair P-Value S.E.

----------- --------------------- ------- -------

rs6108746 AMR & AFR 0.33021 0.00597

rs6108746 EAS & AFR 0.00000 0.00000

rs6108746 EAS & AMR 0.00000 0.00000

rs6108746 EUR & AFR 0.00000 0.00000

rs6108746 EUR & AMR 0.00313 0.00059

rs6108746 EUR & EAS 0.00000 0.00000

rs6108746 SAS & AFR 0.01713 0.00153

rs6108746 SAS & AMR 0.30813 0.00535

rs6108746 SAS & EAS 0.00000 0.00000

rs6108746 SAS & EUR 0.03958 0.00301

Locus: rs6111609

=================================

Pop Alleles:

----------------------------------------

1 2 Total

AFR 11 1311 1322

AMR 67 627 694

EAS 25 983 1008

EUR 208 798 1006

SAS 55 923 978

Total: 366 4642 5008

Locus Population pair P-Value S.E.

----------- --------------------- ------- -------

rs6111609 AMR & AFR 0.00000 0.00000

rs6111609 EAS & AFR 0.00185 0.00028

rs6111609 EAS & AMR 0.00000 0.00000

rs6111609 EUR & AFR 0.00000 0.00000

rs6111609 EUR & AMR 0.00000 0.00000

rs6111609 EUR & EAS 0.00000 0.00000

rs6111609 SAS & AFR 0.00000 0.00000

rs6111609 SAS & AMR 0.00229 0.00037

rs6111609 SAS & EAS 0.00032 0.00010

rs6111609 SAS & EUR 0.00000 0.00000

Locus: rs2922921

=================================

Pop Alleles:

----------------------------------------

1 3 Total

AFR 1 1321 1322

AMR 5 689 694

EAS 0 1008 1008

EUR 18 988 1006

SAS 34 944 978

Total: 58 4950 5008

Locus Population pair P-Value S.E.

----------- --------------------- ------- -------

rs2922921 AMR & AFR 0.02029 0.00039

rs2922921 EAS & AFR 1.00000 0.00000

rs2922921 EAS & AMR 0.01067 0.00026

rs2922921 EUR & AFR 0.00000 0.00000

rs2922921 EUR & AMR 0.06067 0.00106

rs2922921 EUR & EAS 0.00000 0.00000

rs2922921 SAS & AFR 0.00000 0.00000

rs2922921 SAS & AMR 0.00022 0.00006

rs2922921 SAS & EAS 0.00000 0.00000

rs2922921 SAS & EUR 0.02296 0.00092

Locus: rs3922636

=================================

Pop Alleles:

----------------------------------------

1 2 3 Total

AFR 8 13 1301 1322

AMR 143 1 550 694

EAS 230 0 778 1008

EUR 209 0 797 1006

SAS 273 0 705 978

Total: 863 14 4131 5008

Locus Population pair P-Value S.E.

----------- --------------------- ------- -------

rs3922636  AMR & AFR 0.00000 0.00000

rs3922636  EAS & AFR 0.00000 0.00000

rs3922636  EAS & AMR 0.23611 0.00954

rs3922636  EUR & AFR 0.00000 0.00000

rs3922636  EUR & AMR 0.61747 0.00645

rs3922636  EUR & EAS 0.26949 0.00746

rs3922636  SAS & AFR 0.00000 0.00000

rs3922636  SAS & AMR 0.00068 0.00032

rs3922636  SAS & EAS 0.01216 0.00158

rs3922636  SAS & EUR 0.00028 0.00020

=================================

P-value for each population pair across all loci

(Fisher's method)

-----------------------------------------------------

Population pair Chi2 df P-Value

-------------------- --------- ----- ---------

AFR & AMR >634.16796 64 <0.00e+00

AFR & EAS >714.82072 64 <0.00e+00

AMR & EAS >522.35786 64 <0.00e+00

AFR & EUR >720.69858 64 <0.00e+00

AMR & EUR >405.48657 64 <0.00e+00

EAS & EUR >670.76655 64 <0.00e+00

AFR & SAS >706.45858 64 <0.00e+00

AMR & SAS >414.23680 64 <0.00e+00

EAS & SAS >523.72387 64 <0.00e+00

EUR & SAS >518.22774 64 <0.00e+00

**Genotypic differentiation for each ethnic superpopulation pair (exact G test)**

**Allele codes: A – 1, C – 2, G – 3, T - 4**

Markov chain parameters

Dememorisation : 10000

Batches : 100

Iterations per batch : 5000

Locus: rs12608932

=================================

Pop Genotypes:

----------------------------------------

1 1 2

1 2 2 Total

AFR 301 284 76 661

AMR 167 149 31 347

EAS 47 212 245 504

EUR 213 229 61 503

SAS 132 241 116 489

Total: 860 1115529 2504

Locus Population pair P-Value S.E.

----------- --------------------- ------- -------

rs12608932 AMR & AFR 0.25051 0.00592

rs12608932 EAS & AFR 0.00000 0.00000

rs12608932 EAS & AMR 0.00000 0.00000

rs12608932 EUR & AFR 0.35946 0.00716

rs12608932 EUR & AMR 0.06295 0.00342

rs12608932 EUR & EAS 0.00000 0.00000

rs12608932 SAS & AFR 0.00000 0.00000

rs12608932 SAS & AMR 0.00000 0.00000

rs12608932 SAS & EAS 0.00000 0.00000

rs12608932 SAS & EUR 0.00000 0.00000

Locus: rs302668

=================================

Pop Genotypes:

----------------------------------------

2 2 4

2 4 4 Total

AFR 9 117 535 661

AMR 30 130 187 347

EAS 55 211 238 504

EUR 58 235 210 503

SAS 27 162 300 489

Total: 179 855 1470 2504

Locus Population pair P-Value S.E.

----------- --------------------- ------- -------

rs302668 AMR & AFR 0.00000 0.00000

rs302668 EAS & AFR 0.00000 0.00000

rs302668 EAS & AMR 0.05703 0.00276

rs302668 EUR & AFR 0.00000 0.00000

rs302668 EUR & AMR 0.00144 0.00030

rs302668 EUR & EAS 0.15711 0.00549

rs302668 SAS & AFR 0.00000 0.00000

rs302668 SAS & AMR 0.01659 0.00152

rs302668 SAS & EAS 0.00000 0.00000

rs302668 SAS & EUR 0.00000 0.00000

Locus: rs9268856

=================================

Pop Genotypes:

---------------------------------------

1 1 2

1 2 2 Total

AFR 110 285 266 661

AMR 27 138 182 347

EAS 33 197 274 504

EUR 27 193 283 503

SAS 30 194 265 489

Total: 227 10071270 2504

Locus Population pair P-Value S.E.

----------- --------------------- ------- -------

rs9268856 AMR & AFR 0.00001 0.00001

rs9268856 EAS & AFR 0.00000 0.00000

rs9268856 EAS & AMR 0.50175 0.00503

rs9268856 EUR & AFR 0.00000 0.00000

rs9268856 EUR & AMR 0.16595 0.00505

rs9268856 EUR & EAS 0.43904 0.00647

rs9268856 SAS & AFR 0.00000 0.00000

rs9268856 SAS & AMR 0.45892 0.00576

rs9268856 SAS & EAS 0.95905 0.00075

rs9268856 SAS & EUR 0.44657 0.00549

Locus: rs906175

=================================

Pop Genotypes:

----------------------------------------

2 2 4

2 4 4 Total

AFR 635 25 1 661

AMR 66 138 143 347

EAS 64 245 195 504

EUR 156 245 102 503

SAS 84 241 164 489

Total: 1005894 605 2504

Locus Population pair P-Value S.E.

----------- --------------------- ------- -------

rs906175 AMR & AFR 0.00000 0.00000

rs906175 EAS & AFR 0.00000 0.00000

rs906175 EAS & AMR 0.45756 0.00503

rs906175 EUR & AFR 0.00000 0.00000

rs906175 EUR & AMR 0.00000 0.00000

rs906175 EUR & EAS 0.00000 0.00000

rs906175 SAS & AFR 0.00000 0.00000

rs906175 SAS & AMR 0.25750 0.00569

rs906175 SAS & EAS 0.03151 0.00206

rs906175 SAS & EUR 0.00000 0.00000

Locus: rs17042852

=================================

Pop Genotypes:

----------------------------------------

2 2 4

2 4 4 Total

AFR 0 5 656 661

AMR 1 14 332 347

EAS 4 69 431 504

EUR 3 40 460 503

SAS 3 34 452 489

Total: 11 162 2331 2504

Locus Population pair P-Value S.E.

----------- --------------------- ------- -------

rs17042852 AMR & AFR 0.00041 0.00009

rs17042852 EAS & AFR 0.00000 0.00000

rs17042852 EAS & AMR 0.00000 0.00000

rs17042852 EUR & AFR 0.00000 0.00000

rs17042852 EUR & AMR 0.02265 0.00114

rs17042852 EUR & EAS 0.00573 0.00067

rs17042852 SAS & AFR 0.00000 0.00000

rs17042852 SAS & AMR 0.06679 0.00194

rs17042852 SAS & EAS 0.00131 0.00033

rs17042852 SAS & EUR 0.67535 0.00329

Locus: rs11099660

=================================

Pop Genotypes:

----------------------------------------

2 2 4

2 4 4 Total

AFR 183 339 139 661

AMR 184 131 32 347

EAS 311 172 21 504

EUR 277 196 30 503

SAS 301 165 23 489

Total: 12561003245 2504

Locus Population pair P-Value S.E.

----------- --------------------- ------- -------

rs11099660 AMR & AFR 0.00000 0.00000

rs11099660 EAS & AFR 0.00000 0.00000

rs11099660 EAS & AMR 0.00094 0.00025

rs11099660 EUR & AFR 0.00000 0.00000

rs11099660 EUR & AMR 0.24796 0.00598

rs11099660 EUR & EAS 0.02680 0.00205

rs11099660 SAS & AFR 0.00000 0.00000

rs11099660 SAS & AMR 0.00317 0.00057

rs11099660 SAS & EAS 0.86418 0.00219

rs11099660 SAS & EUR 0.04288 0.00267

Locus: rs4980079

=================================

Pop Genotypes:

----------------------------------------

2 2 4

2 4 4 Total

AFR 492 156 13 661

AMR 154 154 39 347

EAS 220 224 60 504

EUR 167 252 84 503

SAS 278 179 32 489

Total: 1311965 228 2504

Locus Population pair P-Value S.E.

----------- --------------------- ------- -------

rs4980079 AMR & AFR 0.00000 0.00000

rs4980079 EAS & AFR 0.00000 0.00000

rs4980079 EAS & AMR 0.79375 0.00331

rs4980079 EUR & AFR 0.00000 0.00000

rs4980079 EUR & AMR 0.00058 0.00019

rs4980079 EUR & EAS 0.00058 0.00021

rs4980079 SAS & AFR 0.00000 0.00000

rs4980079 SAS & AMR 0.00012 0.00010

rs4980079 SAS & EAS 0.00020 0.00020

rs4980079 SAS & EUR 0.00000 0.00000

Locus: rs6857

=================================

Pop Genotypes:

----------------------------------------

2 2 4

2 4 4 Total

AFR 592 68 1 661

AMR 279 63 5 347

EAS 408 91 5 504

EUR 353 134 16 503

SAS 356 123 10 489

Total: 1988479 37 2504

Locus Population pair P-Value S.E.

----------- --------------------- ------- -------

rs6857 AMR & AFR 0.00005 0.00003

rs6857 EAS & AFR 0.00002 0.00002

rs6857 EAS & AMR 0.74795 0.00328

rs6857 EUR & AFR 0.00000 0.00000

rs6857 EUR & AMR 0.00081 0.00023

rs6857 EUR & EAS 0.00002 0.00001

rs6857 SAS & AFR 0.00000 0.00000

rs6857 SAS & AMR 0.01399 0.00113

rs6857 SAS & EAS 0.00215 0.00043

rs6857 SAS & EUR 0.28032 0.00625

Locus: rs3110643

=================================

Pop Genotypes:

----------------------------------------

2 2 4

2 4 4 Total

AFR 5 102 554 661

AMR 5 64 278 347

EAS 0 1 503 504

EUR 11 153 339 503

SAS 1 50 438 489

Total: 22 370 2112 2504

Locus Population pair P-Value S.E.

----------- --------------------- ------- -------

rs3110643 AMR & AFR 0.12664 0.00421

rs3110643 EAS & AFR 0.00000 0.00000

rs3110643 EAS & AMR 0.00000 0.00000

rs3110643 EUR & AFR 0.00000 0.00000

rs3110643 EUR & AMR 0.00025 0.00014

rs3110643 EUR & EAS 0.00000 0.00000

rs3110643 SAS & AFR 0.00332 0.00051

rs3110643 SAS & AMR 0.00008 0.00005

rs3110643 SAS & EAS 0.00000 0.00000

rs3110643 SAS & EUR 0.00000 0.00000

Locus: rs10816848

=================================

Pop Genotypes:

----------------------------------------

1 1 4

1 4 4 Total

AFR 108 329 224 661

AMR 46 146 155 347

EAS 28 178 298 504

EUR 111 233 159 503

SAS 35 192 262 489

Total: 328 10781098 2504

Locus Population pair P-Value S.E.

----------- --------------------- ------- -------

rs10816848 AMR & AFR 0.00209 0.00041

rs10816848 EAS & AFR 0.00000 0.00000

rs10816848 EAS & AMR 0.00000 0.00000

rs10816848 EUR & AFR 0.05819 0.00351

rs10816848 EUR & AMR 0.00001 0.00001

rs10816848 EUR & EAS 0.00000 0.00000

rs10816848 SAS & AFR 0.00000 0.00000

rs10816848 SAS & AMR 0.00115 0.00029

rs10816848 SAS & EAS 0.07187 0.00382

rs10816848 SAS & EUR 0.00000 0.00000

Locus: rs79095029

=================================

Pop Genotypes:

----------------------------------------

2 2 3

2 3 3 Total

AFR 540 116 5 661

AMR 324 21 2 347

EAS 464 38 2 504

EUR 453 48 2 503

SAS 465 24 0 489

Total: 2246247 11 2504

Locus Population pair P-Value S.E.

----------- --------------------- ------- -------

rs79095029 AMR & AFR 0.00000 0.00000

rs79095029 EAS & AFR 0.00000 0.00000

rs79095029 EAS & AMR 0.62644 0.00338

rs79095029 EUR & AFR 0.00026 0.00023

rs79095029 EUR & AMR 0.13812 0.00343

rs79095029 EUR & EAS 0.30681 0.00518

rs79095029 SAS & AFR 0.00000 0.00000

rs79095029 SAS & AMR 0.20006 0.00314

rs79095029 SAS & EAS 0.03379 0.00170

rs79095029 SAS & EUR 0.00324 0.00056

Locus: rs13393316

=================================

Pop Genotypes:

----------------------------------------

1 1 3

1 3 3 Total

AFR 535 119 7 661

AMR 181 139 27 347

EAS 504 0 0 504

EUR 368 120 15 503

SAS 453 35 1 489

Total: 2041413 50 2504

Locus Population pair P-Value S.E.

----------- --------------------- ------- -------

rs13393316 AMR & AFR 0.00000 0.00000

rs13393316 EAS & AFR 0.00000 0.00000

rs13393316 EAS & AMR 0.00000 0.00000

rs13393316 EUR & AFR 0.00068 0.00022

rs13393316 EUR & AMR 0.00000 0.00000

rs13393316 EUR & EAS 0.00000 0.00000

rs13393316 SAS & AFR 0.00000 0.00000

rs13393316 SAS & AMR 0.00000 0.00000

rs13393316 SAS & EAS 0.00000 0.00000

rs13393316 SAS & EUR 0.00000 0.00000

Locus: rs7791726

=================================

Pop Genotypes:

----------------------------------------

2 2 3

2 3 3 Total

AFR 360 257 44 661

AMR 115 170 62 347

EAS 219 217 68 504

EUR 80 250 173 503

SAS 201 228 60 489

Total: 975 1122407 2504

Locus Population pair P-Value S.E.

----------- --------------------- ------- -------

rs7791726 AMR & AFR 0.00000 0.00000

rs7791726 EAS & AFR 0.00001 0.00001

rs7791726 EAS & AMR 0.00242 0.00036

rs7791726 EUR & AFR 0.00000 0.00000

rs7791726 EUR & AMR 0.00000 0.00000

rs7791726 EUR & EAS 0.00000 0.00000

rs7791726 SAS & AFR 0.00000 0.00000

rs7791726 SAS & AMR 0.00628 0.00089

rs7791726 SAS & EAS 0.81031 0.00339

rs7791726 SAS & EUR 0.00000 0.00000

Locus: rs10101195

=================================

Pop Genotypes:

----------------------------------------

1 1 2

1 2 2 Total

AFR 179 336 146 661

AMR 27 133 187 347

EAS 10 148 346 504

EUR 37 198 268 503

SAS 14 119 356 489

Total: 267 934 1303 2504

Locus Population pair P-Value S.E.

----------- --------------------- ------- -------

rs10101195 AMR & AFR 0.00000 0.00000

rs10101195 EAS & AFR 0.00000 0.00000

rs10101195 EAS & AMR 0.00000 0.00000

rs10101195 EUR & AFR 0.00000 0.00000

rs10101195 EUR & AMR 1.00000 0.00000

rs10101195 EUR & EAS 0.00000 0.00000

rs10101195 SAS & AFR 0.00000 0.00000

rs10101195 SAS & AMR 0.00000 0.00000

rs10101195 SAS & EAS 0.33125 0.00655

rs10101195 SAS & EUR 0.00000 0.00000

Locus: rs36196656

=================================

Pop Genotypes:

----------------------------------------

1 1 2

1 2 2 Total

AFR 444 195 22 661

AMR 68 173 106 347

EAS 182 236 86 504

EUR 62 271 170 503

SAS 67 233 189 489

Total: 823 1108573 2504

Locus Population pair P-Value S.E.

----------- --------------------- ------- -------

rs36196656 AMR & AFR 0.00000 0.00000

rs36196656 EAS & AFR 0.00000 0.00000

rs36196656 EAS & AMR 0.00000 0.00000

rs36196656 EUR & AFR 0.00000 0.00000

rs36196656 EUR & AMR 0.02765 0.00196

rs36196656 EUR & EAS 0.00000 0.00000

rs36196656 SAS & AFR 0.00000 0.00000

rs36196656 SAS & AMR 0.00453 0.00061

rs36196656 SAS & EAS 0.00000 0.00000

rs36196656 SAS & EUR 0.41212 0.00684

Locus: rs13072484

=================================

Pop Genotypes:

----------------------------------------

1 1 3

1 3 3 Total

AFR 39 252 370 661

AMR 8 76 263 347

EAS 9 111 384 504

EUR 29 174 300 503

SAS 44 199 246 489

Total: 129 812 1563 2504

Locus Population pair P-Value S.E.

----------- --------------------- ------- -------

rs13072484 AMR & AFR 0.00000 0.00000

rs13072484 EAS & AFR 0.00000 0.00000

rs13072484 EAS & AMR 0.82608 0.00238

rs13072484 EUR & AFR 0.31005 0.00675

rs13072484 EUR & AMR 0.00000 0.00000

rs13072484 EUR & EAS 0.00000 0.00000

rs13072484 SAS & AFR 0.01641 0.00169

rs13072484 SAS & AMR 0.00000 0.00000

rs13072484 SAS & EAS 0.00000 0.00000

rs13072484 SAS & EUR 0.00136 0.00023

Locus: rs62443267

=================================

Pop Genotypes:

----------------------------------------

2 2 4

2 4 4 Total

AFR 536 115 10 661

AMR 194 129 24 347

EAS 347 145 12 504

EUR 288 191 24 503

SAS 394 90 5 489

Total: 1759670 75 2504

Locus Population pair P-Value S.E.

----------- --------------------- ------- -------

rs62443267 AMR & AFR 0.00000 0.00000

rs62443267 EAS & AFR 0.00000 0.00000

rs62443267 EAS & AMR 0.00001 0.00001

rs62443267 EUR & AFR 0.00000 0.00000

rs62443267 EUR & AMR 0.42108 0.00576

rs62443267 EUR & EAS 0.00004 0.00002

rs62443267 SAS & AFR 1.00000 0.00000

rs62443267 SAS & AMR 0.00000 0.00000

rs62443267 SAS & EAS 0.00000 0.00000

rs62443267 SAS & EUR 0.00000 0.00000

Locus: rs10860097

=================================

Pop Genotypes:

----------------------------------------

1 1 4

1 4 4 Total

AFR 660 1 0 661

AMR 336 11 0 347

EAS 424 77 3 504

EUR 476 27 0 503

SAS 433 56 0 489

Total: 2329172 3 2504

Locus Population pair P-Value S.E.

----------- --------------------- ------- -------

rs10860097 AMR & AFR 0.00009 0.00003

rs10860097 EAS & AFR 0.00000 0.00000

rs10860097 EAS & AMR 0.00000 0.00000

rs10860097 EUR & AFR 0.00000 0.00000

rs10860097 EUR & AMR 0.13229 0.00199

rs10860097 EUR & EAS 0.00000 0.00000

rs10860097 SAS & AFR 0.00000 0.00000

rs10860097 SAS & AMR 0.00000 0.00000

rs10860097 SAS & EAS 0.03311 0.00199

rs10860097 SAS & EUR 0.00068 0.00013

Locus: rs7240419

=================================

Pop Genotypes:

----------------------------------------

1 1 3

1 3 3 Total

AFR 38 228 395 661

AMR 20 102 225 347

EAS 94 265 145 504

EUR 44 170 289 503

SAS 104 213 172 489

Total: 300 978 1226 2504

Locus Population pair P-Value S.E.

----------- --------------------- ------- -------

rs7240419 AMR & AFR 0.20928 0.00582

rs7240419 EAS & AFR 0.00000 0.00000

rs7240419 EAS & AMR 0.00000 0.00000

rs7240419 EUR & AFR 0.15582 0.00589

rs7240419 EUR & AMR 0.02086 0.00191

rs7240419 EUR & EAS 0.00000 0.00000

rs7240419 SAS & AFR 0.00000 0.00000

rs7240419 SAS & AMR 0.00000 0.00000

rs7240419 SAS & EAS 0.42646 0.00626

rs7240419 SAS & EUR 0.00000 0.00000

Locus: rs6076187

=================================

Pop Genotypes:

----------------------------------------

1 1 3

1 3 3 Total

AFR 58 260 343 661

AMR 2 43 302 347

EAS 4 99 401 504

EUR 3 39 461 503

SAS 1 37 451 489

Total: 68 478 1958 2504

Locus Population pair P-Value S.E.

----------- --------------------- ------- -------

rs6076187 AMR & AFR 0.00000 0.00000

rs6076187 EAS & AFR 0.00000 0.00000

rs6076187 EAS & AMR 0.00784 0.00082

rs6076187 EUR & AFR 0.00000 0.00000

rs6076187 EUR & AMR 0.05601 0.00220

rs6076187 EUR & EAS 0.00000 0.00000

rs6076187 SAS & AFR 0.00000 0.00000

rs6076187 SAS & AMR 0.01475 0.00130

rs6076187 SAS & EAS 0.00000 0.00000

rs6076187 SAS & EUR 0.66935 0.00356

Locus: rs4676496

=================================

Pop Genotypes:

----------------------------------------

1 1 3

1 3 3 Total

AFR 251 304 106 661

AMR 115 170 62 347

EAS 224 211 69 504

EUR 139 245 119 503

SAS 203 233 53 489

Total: 932 1163409 2504

Locus Population pair P-Value S.E.

----------- --------------------- ------- -------

rs4676496 AMR & AFR 0.15731 0.00501

rs4676496 EAS & AFR 0.03673 0.00279

rs4676496 EAS & AMR 0.00251 0.00048

rs4676496 EUR & AFR 0.00000 0.00000

rs4676496 EUR & AMR 0.02388 0.00180

rs4676496 EUR & EAS 0.00000 0.00000

rs4676496 SAS & AFR 0.03203 0.00249

rs4676496 SAS & AMR 0.00134 0.00027

rs4676496 SAS & EAS 1.00000 0.00000

rs4676496 SAS & EUR 0.00000 0.00000

Locus: rs517339

=================================

Pop Genotypes:

----------------------------------------

2 2 4

2 4 4 Total

AFR 137 366 158 661

AMR 29 143 175 347

EAS 5 105 394 504

EUR 71 247 185 503

SAS 38 198 253 489

Total: 280 10591165 2504

Locus Population pair P-Value S.E.

----------- --------------------- ------- -------

rs517339 AMR & AFR 0.00000 0.00000

rs517339 EAS & AFR 0.00000 0.00000

rs517339 EAS & AMR 0.00000 0.00000

rs517339 EUR & AFR 0.00000 0.00000

rs517339 EUR & AMR 0.00000 0.00000

rs517339 EUR & EAS 0.00000 0.00000

rs517339 SAS & AFR 0.00000 0.00000

rs517339 SAS & AMR 0.69761 0.00379

rs517339 SAS & EAS 0.00000 0.00000

rs517339 SAS & EUR 0.00000 0.00000

Locus: rs12554036

=================================

Pop Genotypes:

----------------------------------------

3 3 4

3 4 4 Total

AFR 555 102 4 661

AMR 265 68 14 347

EAS 450 53 1 504

EUR 294 187 22 503

SAS 326 150 13 489

Total: 1890560 54 2504

Locus Population pair P-Value S.E.

----------- --------------------- ------- -------

rs12554036 AMR & AFR 0.00009 0.00004

rs12554036 EAS & AFR 0.00642 0.00092

rs12554036 EAS & AMR 0.00000 0.00000

rs12554036 EUR & AFR 0.00000 0.00000

rs12554036 EUR & AMR 0.00002 0.00002

rs12554036 EUR & EAS 0.00000 0.00000

rs12554036 SAS & AFR 0.00000 0.00000

rs12554036 SAS & AMR 0.02849 0.00173

rs12554036 SAS & EAS 0.00000 0.00000

rs12554036 SAS & EUR 0.00477 0.00086

Locus: rs4810992

=================================

Pop Genotypes:

----------------------------------------

1 1 3

1 3 3 Total

AFR 0 15 646 661

AMR 9 97 241 347

EAS 4 68 432 504

EUR 52 218 233 503

SAS 3 133 353 489

Total: 68 531 1905 2504

Locus Population pair P-Value S.E.

----------- --------------------- ------- -------

rs4810992 AMR & AFR 0.00000 0.00000

rs4810992 EAS & AFR 0.00000 0.00000

rs4810992 EAS & AMR 0.00000 0.00000

rs4810992 EUR & AFR 0.00000 0.00000

rs4810992 EUR & AMR 0.00000 0.00000

rs4810992 EUR & EAS 0.00000 0.00000

rs4810992 SAS & AFR 0.00000 0.00000

rs4810992 SAS & AMR 0.17787 0.00547

rs4810992 SAS & EAS 0.00000 0.00000

rs4810992 SAS & EUR 0.00000 0.00000

Locus: rs229243

=================================

Pop Genotypes:

----------------------------------------

1 1 2

1 2 2 Total

AFR 278 298 85 661

AMR 91 160 96 347

EAS 216 224 64 504

EUR 55 241 207 503

SAS 85 245 159 489

Total: 725 1168611 2504

Locus Population pair P-Value S.E.

----------- --------------------- ------- -------

rs229243 AMR & AFR 0.00000 0.00000

rs229243 EAS & AFR 0.83091 0.00295

rs229243 EAS & AMR 0.00000 0.00000

rs229243 EUR & AFR 0.00000 0.00000

rs229243 EUR & AMR 0.00000 0.00000

rs229243 EUR & EAS 0.00000 0.00000

rs229243 SAS & AFR 0.00000 0.00000

rs229243 SAS & AMR 0.00659 0.00094

rs229243 SAS & EAS 0.00000 0.00000

rs229243 SAS & EUR 0.00036 0.00014

Locus: rs6809184

=================================

Pop Genotypes:

----------------------------------------

2 2 4

2 4 4 Total

AFR 525 127 9 661

AMR 328 18 1 347

EAS 497 7 0 504

EUR 447 55 1 503

SAS 399 83 7 489

Total: 2196290 18 2504

Locus Population pair P-Value S.E.

----------- --------------------- ------- -------

rs6809184 AMR & AFR 0.00000 0.00000

rs6809184 EAS & AFR 0.00000 0.00000

rs6809184 EAS & AMR 0.00057 0.00012

rs6809184 EUR & AFR 0.00001 0.00001

rs6809184 EUR & AMR 0.00550 0.00065

rs6809184 EUR & EAS 0.00000 0.00000

rs6809184 SAS & AFR 0.46161 0.00607

rs6809184 SAS & AMR 0.00000 0.00000

rs6809184 SAS & EAS 0.00000 0.00000

rs6809184 SAS & EUR 0.00054 0.00015

Locus: rs6962939

=================================

Pop Genotypes:

----------------------------------------

1 1 4

1 4 4 Total

AFR 12 164 485 661

AMR 0 23 324 347

EAS 0 3 501 504

EUR 1 32 470 503

SAS 0 36 453 489

Total: 13 258 2233 2504

Locus Population pair P-Value S.E.

----------- --------------------- ------- -------

rs6962939 AMR & AFR 0.00000 0.00000

rs6962939 EAS & AFR 0.00000 0.00000

rs6962939 EAS & AMR 0.00000 0.00000

rs6962939 EUR & AFR 0.00000 0.00000

rs6962939 EUR & AMR 1.00000 0.00000

rs6962939 EUR & EAS 0.00000 0.00000

rs6962939 SAS & AFR 0.00000 0.00000

rs6962939 SAS & AMR 0.78416 0.00164

rs6962939 SAS & EAS 0.00000 0.00000

rs6962939 SAS & EUR 0.80635 0.00249

Locus: rs9792144

=================================

Pop Genotypes:

----------------------------------------

2 2 3

2 3 3 Total

AFR 432 200 29 661

AMR 249 88 10 347

EAS 340 149 15 504

EUR 379 109 15 503

SAS 363 116 10 489

Total: 1763662 79 2504

Locus Population pair P-Value S.E.

----------- --------------------- ------- -------

rs9792144 AMR & AFR 0.03473 0.00190

rs9792144 EAS & AFR 0.29521 0.00718

rs9792144 EAS & AMR 0.23473 0.00494

rs9792144 EUR & AFR 0.00037 0.00012

rs9792144 EUR & AMR 0.34581 0.00555

rs9792144 EUR & EAS 0.01829 0.00140

rs9792144 SAS & AFR 0.00034 0.00011

rs9792144 SAS & AMR 0.36169 0.00543

rs9792144 SAS & EAS 0.01975 0.00176

rs9792144 SAS & EUR 1.00000 0.00000

Locus: rs6108746

=================================

Pop Genotypes:

----------------------------------------

2 2 4

2 4 4 Total

AFR 12 142 507 661

AMR 7 84 256 347

EAS 0 38 466 504

EUR 21 155 327 503

SAS 15 127 347 489

Total: 55 546 1903 2504

Locus Population pair P-Value S.E.

----------- --------------------- ------- -------

rs6108746 AMR & AFR 0.34013 0.00594

rs6108746 EAS & AFR 0.00000 0.00000

rs6108746 EAS & AMR 0.00000 0.00000

rs6108746 EUR & AFR 0.00000 0.00000

rs6108746 EUR & AMR 0.00353 0.00056

rs6108746 EUR & EAS 0.00000 0.00000

rs6108746 SAS & AFR 0.01700 0.00193

rs6108746 SAS & AMR 0.31050 0.00604

rs6108746 SAS & EAS 0.00000 0.00000

rs6108746 SAS & EUR 0.04400 0.00277

Locus: rs6111609

=================================

Pop Genotypes:

----------------------------------------

1 1 2

1 2 2 Total

AFR 0 11 650 661

AMR 3 61 283 347

EAS 0 25 479 504

EUR 18 172 313 503

SAS 4 47 438 489

Total: 25 316 2163 2504

Locus Population pair P-Value S.E.

----------- --------------------- ------- -------

rs6111609 AMR & AFR 0.00000 0.00000

rs6111609 EAS & AFR 0.00192 0.00021

rs6111609 EAS & AMR 0.00000 0.00000

rs6111609 EUR & AFR 0.00000 0.00000

rs6111609 EUR & AMR 0.00000 0.00000

rs6111609 EUR & EAS 0.00000 0.00000

rs6111609 SAS & AFR 0.00000 0.00000

rs6111609 SAS & AMR 0.00261 0.00035

rs6111609 SAS & EAS 0.00078 0.00029

rs6111609 SAS & EUR 0.00000 0.00000

Locus: rs2922921

=================================

Pop Genotypes:

----------------------------------------

1 3

3 3 Total

AFR 1 660 661

AMR 5 342 347

EAS 0 504 504

EUR 18 485 503

SAS 34 455 489

Total: 58 2446 2504

Locus Population pair P-Value S.E.

----------- --------------------- ------- -------

rs2922921 AMR & AFR 0.02002 0.00046

rs2922921 EAS & AFR 1.00000 0.00000

rs2922921 EAS & AMR 0.01119 0.00030

rs2922921 EUR & AFR 0.00000 0.00000

rs2922921 EUR & AMR 0.05928 0.00106

rs2922921 EUR & EAS 0.00002 0.00001

rs2922921 SAS & AFR 0.00000 0.00000

rs2922921 SAS & AMR 0.00007 0.00002

rs2922921 SAS & EAS 0.00000 0.00000

rs2922921 SAS & EUR 0.02250 0.00080

Locus: rs3922636

=================================

Pop Genotypes:

----------------------------------------

1 1 2 3

1 3 3 3 Total

AFR 0 8 13 640 661

AMR 17 109 1 220 347

EAS 24 182 0 298 504

EUR 23 163 0 317 503

SAS 39 195 0 255 489

Total: 103 657 14 1730 2504

Locus Population pair P-Value S.E.

----------- --------------------- ------- -------

rs3922636  AMR & AFR 0.00000 0.00000

rs3922636  EAS & AFR 0.00000 0.00000

rs3922636  EAS & AMR 0.23352 0.00707

rs3922636  EUR & AFR 0.00000 0.00000

rs3922636  EUR & AMR 0.61959 0.00528

rs3922636  EUR & EAS 0.28115 0.00604

rs3922636  SAS & AFR 0.00000 0.00000

rs3922636  SAS & AMR 0.00116 0.00038

rs3922636  SAS & EAS 0.01059 0.00128

rs3922636  SAS & EUR 0.00022 0.00011

=================================

P-value for each population pair across all loci

(Fisher's method)

-----------------------------------------------------

Population pair Chi2 df P-Value

-------------------- --------- ----- ---------

AFR & AMR >636.78735 64 <0.00e+00

AFR & EAS >707.46017 64 <0.00e+00

AMR & EAS >531.83260 64 <0.00e+00

AFR & EUR >711.88449 64 <0.00e+00

AMR & EUR >401.40107 64 <0.00e+00

EAS & EUR >666.26688 64 <0.00e+00

AFR & SAS >708.29544 64 <0.00e+00

AMR & SAS >414.89326 64 <0.00e+00

EAS & SAS >524.28714 64 <0.00e+00

EUR & SAS >519.42008 64 <0.00e+00
