# Supplementary material for: Genetic structure of different ethnic populations at the frontotemporal dementia risk loci
Source: PLoS One. 2025 Aug 5;20(8):e0329809. doi: 10.1371/journal.pone.0329809 (PMC12324132; doi:10.1371/journal.pone.0329809)
Supplement: S2 Appendix — (DOCX) [file pone.0329809.s008.docx]

**Genic differentiation for each ethnic subpopulation pair (exact G test)**

**Allele codes: A – 1, C – 2, G – 3, T - 4**

Markov chain parameters

Dememorisation : 10000

Batches : 100

Iterations per batch : 5000

Locus: rs12608932

=================================

Pop Alleles:

----------------------------------------

1 2 Total

ACB 118 74 192

ASW 76 46 122

ESN 140 58 198

GWD 163 63 226

LWK 151 47 198

MSL 97 73 170

YRI 141 75 216

PUR 144 64 208

CLM 128 60 188

PEL 117 53 170

MXL 94 34 128

CHS 76 134 210

CDX 44 142 186

KHV 72 126 198

CHB 44 162 206

JPT 70 138 208

CEU 133 65 198

FIN 114 84 198

GBR 114 68 182

IBS 145 69 214

TSI 149 65 214

BEB 88 84 172

GIH 103 103 206

ITU 102 102 204

PJL 103 89 192

STU 109 95 204

Total: 28352173 5008

Locus Population pair P-Value S.E.

----------- --------------------- ------- -------

rs12608932 ASW & ACB 0.90789 0.00091

rs12608932 ESN & ACB 0.05581 0.00192

rs12608932 ESN & ASW 0.13896 0.00244

rs12608932 GWD & ACB 0.02222 0.00119

rs12608932 GWD & ASW 0.06832 0.00190

rs12608932 GWD & ESN 0.83135 0.00140

rs12608932 LWK & ACB 0.00238 0.00025

rs12608932 LWK & ASW 0.01029 0.00065

rs12608932 LWK & ESN 0.25698 0.00337

rs12608932 LWK & GWD 0.37533 0.00376

rs12608932 MSL & ACB 0.45586 0.00316

rs12608932 MSL & ASW 0.39706 0.00352

rs12608932 MSL & ESN 0.01033 0.00071

rs12608932 MSL & GWD 0.00214 0.00029

rs12608932 MSL & LWK 0.00008 0.00003

rs12608932 YRI & ACB 0.47662 0.00437

rs12608932 YRI & ASW 0.63716 0.00302

rs12608932 YRI & ESN 0.24677 0.00353

rs12608932 YRI & GWD 0.12369 0.00285

rs12608932 YRI & LWK 0.01774 0.00097

rs12608932 YRI & MSL 0.11322 0.00243

rs12608932 PUR & ACB 0.11402 0.00273

rs12608932 PUR & ASW 0.23082 0.00338

rs12608932 PUR & ESN 0.82964 0.00167

rs12608932 PUR & GWD 0.51905 0.00305

rs12608932 PUR & LWK 0.12476 0.00249

rs12608932 PUR & MSL 0.01808 0.00099

rs12608932 PUR & YRI 0.40690 0.00365

rs12608932 CLM & ACB 0.19546 0.00326

rs12608932 CLM & ASW 0.32989 0.00315

rs12608932 CLM & ESN 0.58109 0.00358

rs12608932 CLM & GWD 0.38933 0.00356

rs12608932 CLM & LWK 0.08896 0.00219

rs12608932 CLM & MSL 0.03652 0.00154

rs12608932 CLM & YRI 0.59757 0.00327

rs12608932 CLM & PUR 0.83111 0.00156

rs12608932 PEL & ACB 0.15433 0.00292

rs12608932 PEL & ASW 0.26513 0.00316

rs12608932 PEL & ESN 0.73415 0.00208

rs12608932 PEL & GWD 0.50232 0.00328

rs12608932 PEL & LWK 0.12615 0.00269

rs12608932 PEL & MSL 0.03302 0.00124

rs12608932 PEL & YRI 0.50973 0.00348

rs12608932 PEL & PUR 1.00000 0.00000

rs12608932 PEL & CLM 0.91008 0.00080

rs12608932 MXL & ACB 0.03015 0.00108

rs12608932 MXL & ASW 0.07767 0.00169

rs12608932 MXL & ESN 0.61800 0.00273

rs12608932 MXL & GWD 0.80503 0.00173

rs12608932 MXL & LWK 0.60349 0.00246

rs12608932 MXL & MSL 0.00422 0.00047

rs12608932 MXL & YRI 0.11806 0.00235

rs12608932 MXL & PUR 0.46398 0.00295

rs12608932 MXL & CLM 0.31627 0.00352

rs12608932 MXL & PEL 0.44031 0.00315

rs12608932 CHS & ACB 0.00000 0.00000

rs12608932 CHS & ASW 0.00002 0.00002

rs12608932 CHS & ESN 0.00000 0.00000

rs12608932 CHS & GWD 0.00000 0.00000

rs12608932 CHS & LWK 0.00000 0.00000

rs12608932 CHS & MSL 0.00010 0.00008

rs12608932 CHS & YRI 0.00000 0.00000

rs12608932 CHS & PUR 0.00000 0.00000

rs12608932 CHS & CLM 0.00000 0.00000

rs12608932 CHS & PEL 0.00000 0.00000

rs12608932 CHS & MXL 0.00000 0.00000

rs12608932 CDX & ACB 0.00000 0.00000

rs12608932 CDX & ASW 0.00000 0.00000

rs12608932 CDX & ESN 0.00000 0.00000

rs12608932 CDX & GWD 0.00000 0.00000

rs12608932 CDX & LWK 0.00000 0.00000

rs12608932 CDX & MSL 0.00000 0.00000

rs12608932 CDX & YRI 0.00000 0.00000

rs12608932 CDX & PUR 0.00000 0.00000

rs12608932 CDX & CLM 0.00000 0.00000

rs12608932 CDX & PEL 0.00000 0.00000

rs12608932 CDX & MXL 0.00000 0.00000

rs12608932 CDX & CHS 0.00923 0.00070

rs12608932 KHV & ACB 0.00000 0.00000

rs12608932 KHV & ASW 0.00000 0.00000

rs12608932 KHV & ESN 0.00000 0.00000

rs12608932 KHV & GWD 0.00000 0.00000

rs12608932 KHV & LWK 0.00000 0.00000

rs12608932 KHV & MSL 0.00009 0.00003

rs12608932 KHV & YRI 0.00000 0.00000

rs12608932 KHV & PUR 0.00000 0.00000

rs12608932 KHV & CLM 0.00000 0.00000

rs12608932 KHV & PEL 0.00000 0.00000

rs12608932 KHV & MXL 0.00000 0.00000

rs12608932 KHV & CHS 1.00000 0.00000

rs12608932 KHV & CDX 0.00700 0.00055

rs12608932 CHB & ACB 0.00000 0.00000

rs12608932 CHB & ASW 0.00000 0.00000

rs12608932 CHB & ESN 0.00000 0.00000

rs12608932 CHB & GWD 0.00000 0.00000

rs12608932 CHB & LWK 0.00000 0.00000

rs12608932 CHB & MSL 0.00000 0.00000

rs12608932 CHB & YRI 0.00000 0.00000

rs12608932 CHB & PUR 0.00000 0.00000

rs12608932 CHB & CLM 0.00000 0.00000

rs12608932 CHB & PEL 0.00000 0.00000

rs12608932 CHB & MXL 0.00000 0.00000

rs12608932 CHB & CHS 0.00115 0.00021

rs12608932 CHB & CDX 0.62726 0.00278

rs12608932 CHB & KHV 0.00092 0.00019

rs12608932 JPT & ACB 0.00000 0.00000

rs12608932 JPT & ASW 0.00000 0.00000

rs12608932 JPT & ESN 0.00000 0.00000

rs12608932 JPT & GWD 0.00000 0.00000

rs12608932 JPT & LWK 0.00000 0.00000

rs12608932 JPT & MSL 0.00000 0.00000

rs12608932 JPT & YRI 0.00000 0.00000

rs12608932 JPT & PUR 0.00000 0.00000

rs12608932 JPT & CLM 0.00000 0.00000

rs12608932 JPT & PEL 0.00000 0.00000

rs12608932 JPT & MXL 0.00000 0.00000

rs12608932 JPT & CHS 0.61345 0.00356

rs12608932 JPT & CDX 0.03221 0.00132

rs12608932 JPT & KHV 0.60150 0.00320

rs12608932 JPT & CHB 0.00490 0.00046

rs12608932 CEU & ACB 0.24726 0.00358

rs12608932 CEU & ASW 0.39925 0.00332

rs12608932 CEU & ESN 0.52148 0.00333

rs12608932 CEU & GWD 0.28650 0.00355

rs12608932 CEU & LWK 0.05798 0.00173

rs12608932 CEU & MSL 0.05125 0.00187

rs12608932 CEU & YRI 0.75425 0.00217

rs12608932 CEU & PUR 0.66821 0.00290

rs12608932 CEU & CLM 0.91476 0.00084

rs12608932 CEU & PEL 0.73979 0.00248

rs12608932 CEU & MXL 0.26013 0.00327

rs12608932 CEU & CHS 0.00000 0.00000

rs12608932 CEU & CDX 0.00000 0.00000

rs12608932 CEU & KHV 0.00000 0.00000

rs12608932 CEU & CHB 0.00000 0.00000

rs12608932 CEU & JPT 0.00000 0.00000

rs12608932 FIN & ACB 0.46967 0.00435

rs12608932 FIN & ASW 0.41834 0.00405

rs12608932 FIN & ESN 0.00780 0.00068

rs12608932 FIN & GWD 0.00229 0.00035

rs12608932 FIN & LWK 0.00027 0.00009

rs12608932 FIN & MSL 1.00000 0.00000

rs12608932 FIN & YRI 0.12948 0.00266

rs12608932 FIN & PUR 0.01859 0.00112

rs12608932 FIN & CLM 0.03565 0.00157

rs12608932 FIN & PEL 0.03056 0.00145

rs12608932 FIN & MXL 0.00459 0.00037

rs12608932 FIN & CHS 0.00004 0.00003

rs12608932 FIN & CDX 0.00000 0.00000

rs12608932 FIN & KHV 0.00004 0.00004

rs12608932 FIN & CHB 0.00000 0.00000

rs12608932 FIN & JPT 0.00000 0.00000

rs12608932 FIN & CEU 0.05988 0.00201

rs12608932 GBR & ACB 0.83379 0.00179

rs12608932 GBR & ASW 1.00000 0.00000

rs12608932 GBR & ESN 0.10206 0.00240

rs12608932 GBR & GWD 0.04359 0.00176

rs12608932 GBR & LWK 0.00503 0.00050

rs12608932 GBR & MSL 0.32295 0.00330

rs12608932 GBR & YRI 0.59845 0.00277

rs12608932 GBR & PUR 0.19842 0.00332

rs12608932 GBR & CLM 0.27599 0.00404

rs12608932 GBR & PEL 0.25705 0.00366

rs12608932 GBR & MXL 0.04986 0.00149

rs12608932 GBR & CHS 0.00000 0.00000

rs12608932 GBR & CDX 0.00000 0.00000

rs12608932 GBR & KHV 0.00000 0.00000

rs12608932 GBR & CHB 0.00000 0.00000

rs12608932 GBR & JPT 0.00000 0.00000

rs12608932 GBR & CEU 0.39180 0.00374

rs12608932 GBR & FIN 0.34972 0.00380

rs12608932 IBS & ACB 0.20336 0.00375

rs12608932 IBS & ASW 0.33949 0.00354

rs12608932 IBS & ESN 0.52548 0.00340

rs12608932 IBS & GWD 0.34474 0.00335

rs12608932 IBS & LWK 0.06346 0.00168

rs12608932 IBS & MSL 0.03464 0.00140

rs12608932 IBS & YRI 0.60870 0.00316

rs12608932 IBS & PUR 0.75472 0.00271

rs12608932 IBS & CLM 1.00000 0.00000

rs12608932 IBS & PEL 0.82653 0.00161

rs12608932 IBS & MXL 0.27965 0.00343

rs12608932 IBS & CHS 0.00000 0.00000

rs12608932 IBS & CDX 0.00000 0.00000

rs12608932 IBS & KHV 0.00000 0.00000

rs12608932 IBS & CHB 0.00000 0.00000

rs12608932 IBS & JPT 0.00000 0.00000

rs12608932 IBS & CEU 0.91625 0.00086

rs12608932 IBS & FIN 0.03973 0.00167

rs12608932 IBS & GBR 0.29360 0.00444

rs12608932 TSI & ACB 0.09795 0.00255

rs12608932 TSI & ASW 0.18753 0.00275

rs12608932 TSI & ESN 0.83005 0.00167

rs12608932 TSI & GWD 0.59906 0.00312

rs12608932 TSI & LWK 0.15312 0.00312

rs12608932 TSI & MSL 0.01407 0.00098

rs12608932 TSI & YRI 0.35693 0.00444

rs12608932 TSI & PUR 1.00000 0.00000

rs12608932 TSI & CLM 0.74440 0.00220

rs12608932 TSI & PEL 0.91103 0.00089

rs12608932 TSI & MXL 0.46319 0.00325

rs12608932 TSI & CHS 0.00000 0.00000

rs12608932 TSI & CDX 0.00000 0.00000

rs12608932 TSI & KHV 0.00000 0.00000

rs12608932 TSI & CHB 0.00000 0.00000

rs12608932 TSI & JPT 0.00000 0.00000

rs12608932 TSI & CEU 0.59642 0.00322

rs12608932 TSI & FIN 0.01277 0.00088

rs12608932 TSI & GBR 0.16842 0.00321

rs12608932 TSI & IBS 0.75375 0.00219

rs12608932 BEB & ACB 0.05862 0.00206

rs12608932 BEB & ASW 0.07351 0.00212

rs12608932 BEB & ESN 0.00007 0.00004

rs12608932 BEB & GWD 0.00002 0.00001

rs12608932 BEB & LWK 0.00000 0.00000

rs12608932 BEB & MSL 0.28247 0.00341

rs12608932 BEB & YRI 0.00680 0.00057

rs12608932 BEB & PUR 0.00053 0.00012

rs12608932 BEB & CLM 0.00094 0.00016

rs12608932 BEB & PEL 0.00087 0.00017

rs12608932 BEB & MXL 0.00007 0.00003

rs12608932 BEB & CHS 0.00288 0.00041

rs12608932 BEB & CDX 0.00000 0.00000

rs12608932 BEB & KHV 0.00422 0.00040

rs12608932 BEB & CHB 0.00000 0.00000

rs12608932 BEB & JPT 0.00079 0.00016

rs12608932 BEB & CEU 0.00168 0.00022

rs12608932 BEB & FIN 0.25639 0.00388

rs12608932 BEB & GBR 0.03214 0.00120

rs12608932 BEB & IBS 0.00077 0.00016

rs12608932 BEB & TSI 0.00029 0.00012

rs12608932 GIH & ACB 0.02592 0.00115

rs12608932 GIH & ASW 0.03667 0.00136

rs12608932 GIH & ESN 0.00002 0.00002

rs12608932 GIH & GWD 0.00000 0.00000

rs12608932 GIH & LWK 0.00000 0.00000

rs12608932 GIH & MSL 0.18221 0.00361

rs12608932 GIH & YRI 0.00177 0.00025

rs12608932 GIH & PUR 0.00003 0.00002

rs12608932 GIH & CLM 0.00040 0.00012

rs12608932 GIH & PEL 0.00019 0.00006

rs12608932 GIH & MXL 0.00005 0.00005

rs12608932 GIH & CHS 0.00628 0.00067

rs12608932 GIH & CDX 0.00000 0.00000

rs12608932 GIH & KHV 0.00762 0.00072

rs12608932 GIH & CHB 0.00000 0.00000

rs12608932 GIH & JPT 0.00090 0.00023

rs12608932 GIH & CEU 0.00042 0.00010

rs12608932 GIH & FIN 0.13469 0.00327

rs12608932 GIH & GBR 0.01317 0.00096

rs12608932 GIH & IBS 0.00028 0.00009

rs12608932 GIH & TSI 0.00004 0.00002

rs12608932 GIH & BEB 0.83836 0.00160

rs12608932 ITU & ACB 0.02699 0.00133

rs12608932 ITU & ASW 0.03853 0.00153

rs12608932 ITU & ESN 0.00021 0.00014

rs12608932 ITU & GWD 0.00000 0.00000

rs12608932 ITU & LWK 0.00000 0.00000

rs12608932 ITU & MSL 0.17533 0.00333

rs12608932 ITU & YRI 0.00164 0.00027

rs12608932 ITU & PUR 0.00002 0.00001

rs12608932 ITU & CLM 0.00018 0.00008

rs12608932 ITU & PEL 0.00026 0.00007

rs12608932 ITU & MXL 0.00000 0.00000

rs12608932 ITU & CHS 0.00485 0.00045

rs12608932 ITU & CDX 0.00000 0.00000

rs12608932 ITU & KHV 0.00587 0.00057

rs12608932 ITU & CHB 0.00000 0.00000

rs12608932 ITU & JPT 0.00106 0.00020

rs12608932 ITU & CEU 0.00067 0.00019

rs12608932 ITU & FIN 0.12989 0.00358

rs12608932 ITU & GBR 0.01307 0.00095

rs12608932 ITU & IBS 0.00012 0.00004

rs12608932 ITU & TSI 0.00004 0.00003

rs12608932 ITU & BEB 0.83779 0.00168

rs12608932 ITU & GIH 1.00000 0.00000

rs12608932 PJL & ACB 0.14747 0.00293

rs12608932 PJL & ASW 0.16000 0.00303

rs12608932 PJL & ESN 0.00035 0.00009

rs12608932 PJL & GWD 0.00002 0.00001

rs12608932 PJL & LWK 0.00000 0.00000

rs12608932 PJL & MSL 0.52993 0.00352

rs12608932 PJL & YRI 0.02022 0.00118

rs12608932 PJL & PUR 0.00159 0.00028

rs12608932 PJL & CLM 0.00496 0.00042

rs12608932 PJL & PEL 0.00364 0.00041

rs12608932 PJL & MXL 0.00039 0.00010

rs12608932 PJL & CHS 0.00085 0.00016

rs12608932 PJL & CDX 0.00000 0.00000

rs12608932 PJL & KHV 0.00090 0.00019

rs12608932 PJL & CHB 0.00000 0.00000

rs12608932 PJL & JPT 0.00001 0.00001

rs12608932 PJL & CEU 0.00796 0.00067

rs12608932 PJL & FIN 0.47374 0.00346

rs12608932 PJL & GBR 0.09819 0.00268

rs12608932 PJL & IBS 0.00411 0.00042

rs12608932 PJL & TSI 0.00092 0.00017

rs12608932 PJL & BEB 0.67389 0.00305

rs12608932 PJL & GIH 0.47647 0.00408

rs12608932 PJL & ITU 0.48263 0.00424

rs12608932 STU & ACB 0.12687 0.00274

rs12608932 STU & ASW 0.13791 0.00292

rs12608932 STU & ESN 0.00051 0.00016

rs12608932 STU & GWD 0.00005 0.00003

rs12608932 STU & LWK 0.00000 0.00000

rs12608932 STU & MSL 0.53640 0.00329

rs12608932 STU & YRI 0.01640 0.00122

rs12608932 STU & PUR 0.00133 0.00020

rs12608932 STU & CLM 0.00348 0.00040

rs12608932 STU & PEL 0.00301 0.00041

rs12608932 STU & MXL 0.00021 0.00007

rs12608932 STU & CHS 0.00040 0.00009

rs12608932 STU & CDX 0.00000 0.00000

rs12608932 STU & KHV 0.00063 0.00013

rs12608932 STU & CHB 0.00000 0.00000

rs12608932 STU & JPT 0.00009 0.00004

rs12608932 STU & CEU 0.00606 0.00065

rs12608932 STU & FIN 0.42068 0.00373

rs12608932 STU & GBR 0.08565 0.00242

rs12608932 STU & IBS 0.00326 0.00038

rs12608932 STU & TSI 0.00109 0.00024

rs12608932 STU & BEB 0.68430 0.00313

rs12608932 STU & GIH 0.49799 0.00374

rs12608932 STU & ITU 0.56014 0.00350

rs12608932 STU & PJL 1.00000 0.00000

Locus: rs302668

=================================

Pop Alleles:

-----------------------------------------------------------

2 4 Total

ACB 23 169 192

ASW 17 105 122

ESN 23 175 198

GWD 11 215 226

LWK 24 174 198

MSL 15 155 170

YRI 22 194 216

PUR 51 157 208

CLM 36 152 188

PEL 49 121 170

MXL 54 74 128

CHS 52 158 210

CDX 71 115 186

KHV 76 122 198

CHB 51 155 206

JPT 71 137 208

CEU 70 128 198

FIN 87 111 198

GBR 55 127 182

IBS 70 144 214

TSI 69 145 214

BEB 44 128 172

GIH 34 172 206

ITU 53 151 204

PJL 48 144 192

STU 37 167 204

Total: 12133795 5008

Locus Population pair P-Value S.E.

----------- --------------------- ------- -------

rs302668 ASW & ACB 0.73015 0.00142

rs302668 ESN & ACB 1.00000 0.00000

rs302668 ESN & ASW 0.60033 0.00208

rs302668 GWD & ACB 0.01149 0.00054

rs302668 GWD & ASW 0.00617 0.00027

rs302668 GWD & ESN 0.01212 0.00053

rs302668 LWK & ACB 1.00000 0.00000

rs302668 LWK & ASW 0.73091 0.00148

rs302668 LWK & ESN 1.00000 0.00000

rs302668 LWK & GWD 0.00782 0.00042

rs302668 MSL & ACB 0.39173 0.00268

rs302668 MSL & ASW 0.18633 0.00182

rs302668 MSL & ESN 0.39722 0.00251

rs302668 MSL & GWD 0.15213 0.00171

rs302668 MSL & LWK 0.31319 0.00242

rs302668 YRI & ACB 0.63541 0.00226

rs302668 YRI & ASW 0.37515 0.00211

rs302668 YRI & ESN 0.75208 0.00156

rs302668 YRI & GWD 0.04719 0.00112

rs302668 YRI & LWK 0.64046 0.00237

rs302668 YRI & MSL 0.72942 0.00162

rs302668 PUR & ACB 0.00099 0.00017

rs302668 PUR & ASW 0.02521 0.00115

rs302668 PUR & ESN 0.00066 0.00013

rs302668 PUR & GWD 0.00000 0.00000

rs302668 PUR & LWK 0.00156 0.00023

rs302668 PUR & MSL 0.00004 0.00001

rs302668 PUR & YRI 0.00013 0.00006

rs302668 CLM & ACB 0.06410 0.00165

rs302668 CLM & ASW 0.28660 0.00250

rs302668 CLM & ESN 0.04740 0.00141

rs302668 CLM & GWD 0.00001 0.00001

rs302668 CLM & LWK 0.06819 0.00164

rs302668 CLM & MSL 0.00649 0.00045

rs302668 CLM & YRI 0.01429 0.00068

rs302668 CLM & PUR 0.23230 0.00325

rs302668 PEL & ACB 0.00008 0.00005

rs302668 PEL & ASW 0.00324 0.00032

rs302668 PEL & ESN 0.00001 0.00001

rs302668 PEL & GWD 0.00000 0.00000

rs302668 PEL & LWK 0.00006 0.00002

rs302668 PEL & MSL 0.00004 0.00004

rs302668 PEL & YRI 0.00001 0.00001

rs302668 PEL & PUR 0.35270 0.00345

rs302668 PEL & CLM 0.03384 0.00127

rs302668 MXL & ACB 0.00000 0.00000

rs302668 MXL & ASW 0.00000 0.00000

rs302668 MXL & ESN 0.00000 0.00000

rs302668 MXL & GWD 0.00000 0.00000

rs302668 MXL & LWK 0.00000 0.00000

rs302668 MXL & MSL 0.00000 0.00000

rs302668 MXL & YRI 0.00000 0.00000

rs302668 MXL & PUR 0.00076 0.00012

rs302668 MXL & CLM 0.00001 0.00001

rs302668 MXL & PEL 0.01917 0.00082

rs302668 CHS & ACB 0.00118 0.00017

rs302668 CHS & ASW 0.01841 0.00090

rs302668 CHS & ESN 0.00086 0.00016

rs302668 CHS & GWD 0.00000 0.00000

rs302668 CHS & LWK 0.00125 0.00018

rs302668 CHS & MSL 0.00003 0.00002

rs302668 CHS & YRI 0.00010 0.00005

rs302668 CHS & PUR 1.00000 0.00000

rs302668 CHS & CLM 0.18772 0.00294

rs302668 CHS & PEL 0.41420 0.00373

rs302668 CHS & MXL 0.00109 0.00022

rs302668 CDX & ACB 0.00000 0.00000

rs302668 CDX & ASW 0.00001 0.00001

rs302668 CDX & ESN 0.00000 0.00000

rs302668 CDX & GWD 0.00000 0.00000

rs302668 CDX & LWK 0.00000 0.00000

rs302668 CDX & MSL 0.00000 0.00000

rs302668 CDX & YRI 0.00000 0.00000

rs302668 CDX & PUR 0.00469 0.00052

rs302668 CDX & CLM 0.00009 0.00004

rs302668 CDX & PEL 0.07394 0.00215

rs302668 CDX & MXL 0.48713 0.00349

rs302668 CDX & CHS 0.00428 0.00041

rs302668 KHV & ACB 0.00000 0.00000

rs302668 KHV & ASW 0.00000 0.00000

rs302668 KHV & ESN 0.00000 0.00000

rs302668 KHV & GWD 0.00000 0.00000

rs302668 KHV & LWK 0.00000 0.00000

rs302668 KHV & MSL 0.00000 0.00000

rs302668 KHV & YRI 0.00000 0.00000

rs302668 KHV & PUR 0.00278 0.00035

rs302668 KHV & CLM 0.00002 0.00001

rs302668 KHV & PEL 0.06241 0.00171

rs302668 KHV & MXL 0.55619 0.00332

rs302668 KHV & CHS 0.00344 0.00044

rs302668 KHV & CDX 1.00000 0.00000

rs302668 CHB & ACB 0.00111 0.00016

rs302668 CHB & ASW 0.02373 0.00096

rs302668 CHB & ESN 0.00065 0.00012

rs302668 CHB & GWD 0.00000 0.00000

rs302668 CHB & LWK 0.00116 0.00016

rs302668 CHB & MSL 0.00004 0.00002

rs302668 CHB & YRI 0.00007 0.00002

rs302668 CHB & PUR 1.00000 0.00000

rs302668 CHB & CLM 0.18978 0.00318

rs302668 CHB & PEL 0.41257 0.00311

rs302668 CHB & MXL 0.00108 0.00020

rs302668 CHB & CHS 1.00000 0.00000

rs302668 CHB & CDX 0.00446 0.00045

rs302668 CHB & KHV 0.00398 0.00042

rs302668 JPT & ACB 0.00000 0.00000

rs302668 JPT & ASW 0.00003 0.00001

rs302668 JPT & ESN 0.00000 0.00000

rs302668 JPT & GWD 0.00000 0.00000

rs302668 JPT & LWK 0.00000 0.00000

rs302668 JPT & MSL 0.00000 0.00000

rs302668 JPT & YRI 0.00000 0.00000

rs302668 JPT & PUR 0.04102 0.00154

rs302668 JPT & CLM 0.00075 0.00015

rs302668 JPT & PEL 0.31256 0.00371

rs302668 JPT & MXL 0.16407 0.00297

rs302668 JPT & CHS 0.03978 0.00141

rs302668 JPT & CDX 0.46333 0.00386

rs302668 JPT & KHV 0.40805 0.00408

rs302668 JPT & CHB 0.04266 0.00181

rs302668 CEU & ACB 0.00000 0.00000

rs302668 CEU & ASW 0.00001 0.00000

rs302668 CEU & ESN 0.00000 0.00000

rs302668 CEU & GWD 0.00000 0.00000

rs302668 CEU & LWK 0.00000 0.00000

rs302668 CEU & MSL 0.00000 0.00000

rs302668 CEU & YRI 0.00000 0.00000

rs302668 CEU & PUR 0.02222 0.00114

rs302668 CEU & CLM 0.00041 0.00010

rs302668 CEU & PEL 0.21517 0.00338

rs302668 CEU & MXL 0.23713 0.00313

rs302668 CEU & CHS 0.02421 0.00112

rs302668 CEU & CDX 0.59600 0.00345

rs302668 CEU & KHV 0.59932 0.00335

rs302668 CEU & CHB 0.02308 0.00124

rs302668 CEU & JPT 0.83464 0.00176

rs302668 FIN & ACB 0.00000 0.00000

rs302668 FIN & ASW 0.00000 0.00000

rs302668 FIN & ESN 0.00000 0.00000

rs302668 FIN & GWD 0.00000 0.00000

rs302668 FIN & LWK 0.00000 0.00000

rs302668 FIN & MSL 0.00000 0.00000

rs302668 FIN & YRI 0.00000 0.00000

rs302668 FIN & PUR 0.00002 0.00002

rs302668 FIN & CLM 0.00000 0.00000

rs302668 FIN & PEL 0.00343 0.00036

rs302668 FIN & MXL 0.81732 0.00161

rs302668 FIN & CHS 0.00002 0.00001

rs302668 FIN & CDX 0.25115 0.00374

rs302668 FIN & KHV 0.30202 0.00422

rs302668 FIN & CHB 0.00002 0.00002

rs302668 FIN & JPT 0.05259 0.00198

rs302668 FIN & CEU 0.09438 0.00235

rs302668 GBR & ACB 0.00003 0.00003

rs302668 GBR & ASW 0.00094 0.00013

rs302668 GBR & ESN 0.00001 0.00001

rs302668 GBR & GWD 0.00000 0.00000

rs302668 GBR & LWK 0.00001 0.00001

rs302668 GBR & MSL 0.00000 0.00000

rs302668 GBR & YRI 0.00000 0.00000

rs302668 GBR & PUR 0.20672 0.00319

rs302668 GBR & CLM 0.01656 0.00089

rs302668 GBR & PEL 0.81573 0.00179

rs302668 GBR & MXL 0.03907 0.00155

rs302668 GBR & CHS 0.26159 0.00325

rs302668 GBR & CDX 0.12735 0.00274

rs302668 GBR & KHV 0.10882 0.00286

rs302668 GBR & CHB 0.25852 0.00305

rs302668 GBR & JPT 0.45110 0.00384

rs302668 GBR & CEU 0.32245 0.00345

rs302668 GBR & FIN 0.00578 0.00059

rs302668 IBS & ACB 0.00000 0.00000

rs302668 IBS & ASW 0.00015 0.00006

rs302668 IBS & ESN 0.00001 0.00001

rs302668 IBS & GWD 0.00000 0.00000

rs302668 IBS & LWK 0.00000 0.00000

rs302668 IBS & MSL 0.00000 0.00000

rs302668 IBS & YRI 0.00000 0.00000

rs302668 IBS & PUR 0.06465 0.00208

rs302668 IBS & CLM 0.00233 0.00028

rs302668 IBS & PEL 0.44471 0.00345

rs302668 IBS & MXL 0.08384 0.00250

rs302668 IBS & CHS 0.08300 0.00235

rs302668 IBS & CDX 0.29515 0.00392

rs302668 IBS & KHV 0.26182 0.00384

rs302668 IBS & CHB 0.08386 0.00206

rs302668 IBS & JPT 0.75417 0.00207

rs302668 IBS & CEU 0.61127 0.00298

rs302668 IBS & FIN 0.01901 0.00103

rs302668 IBS & GBR 0.66359 0.00262

rs302668 TSI & ACB 0.00000 0.00000

rs302668 TSI & ASW 0.00009 0.00004

rs302668 TSI & ESN 0.00000 0.00000

rs302668 TSI & GWD 0.00000 0.00000

rs302668 TSI & LWK 0.00000 0.00000

rs302668 TSI & MSL 0.00000 0.00000

rs302668 TSI & YRI 0.00000 0.00000

rs302668 TSI & PUR 0.08387 0.00248

rs302668 TSI & CLM 0.00281 0.00031

rs302668 TSI & PEL 0.50330 0.00330

rs302668 TSI & MXL 0.07902 0.00218

rs302668 TSI & CHS 0.10190 0.00252

rs302668 TSI & CDX 0.24596 0.00392

rs302668 TSI & KHV 0.21872 0.00391

rs302668 TSI & CHB 0.10958 0.00267

rs302668 TSI & JPT 0.75273 0.00224

rs302668 TSI & CEU 0.53453 0.00338

rs302668 TSI & FIN 0.01529 0.00095

rs302668 TSI & GBR 0.74123 0.00226

rs302668 TSI & IBS 1.00000 0.00000

rs302668 BEB & ACB 0.00115 0.00019

rs302668 BEB & ASW 0.01957 0.00082

rs302668 BEB & ESN 0.00056 0.00013

rs302668 BEB & GWD 0.00000 0.00000

rs302668 BEB & LWK 0.00111 0.00016

rs302668 BEB & MSL 0.00003 0.00003

rs302668 BEB & YRI 0.00010 0.00005

rs302668 BEB & PUR 0.90483 0.00091

rs302668 BEB & CLM 0.16761 0.00282

rs302668 BEB & PEL 0.54541 0.00328

rs302668 BEB & MXL 0.00286 0.00030

rs302668 BEB & CHS 0.90639 0.00092

rs302668 BEB & CDX 0.01285 0.00073

rs302668 BEB & KHV 0.01062 0.00079

rs302668 BEB & CHB 0.90345 0.00079

rs302668 BEB & JPT 0.07252 0.00213

rs302668 BEB & CEU 0.04394 0.00167

rs302668 BEB & FIN 0.00035 0.00012

rs302668 BEB & GBR 0.34213 0.00349

rs302668 BEB & IBS 0.14504 0.00292

rs302668 BEB & TSI 0.17820 0.00282

rs302668 GIH & ACB 0.19919 0.00252

rs302668 GIH & ASW 0.63779 0.00214

rs302668 GIH & ESN 0.19627 0.00263

rs302668 GIH & GWD 0.00007 0.00003

rs302668 GIH & LWK 0.25690 0.00323

rs302668 GIH & MSL 0.03019 0.00088

rs302668 GIH & YRI 0.06250 0.00177

rs302668 GIH & PUR 0.05179 0.00168

rs302668 GIH & CLM 0.51538 0.00300

rs302668 GIH & PEL 0.00649 0.00053

rs302668 GIH & MXL 0.00000 0.00000

rs302668 GIH & CHS 0.04233 0.00147

rs302668 GIH & CDX 0.00000 0.00000

rs302668 GIH & KHV 0.00000 0.00000

rs302668 GIH & CHB 0.05387 0.00177

rs302668 GIH & JPT 0.00003 0.00002

rs302668 GIH & CEU 0.00005 0.00003

rs302668 GIH & FIN 0.00000 0.00000

rs302668 GIH & GBR 0.00151 0.00025

rs302668 GIH & IBS 0.00005 0.00002

rs302668 GIH & TSI 0.00014 0.00007

rs302668 GIH & BEB 0.04003 0.00134

rs302668 ITU & ACB 0.00045 0.00010

rs302668 ITU & ASW 0.01238 0.00059

rs302668 ITU & ESN 0.00029 0.00009

rs302668 ITU & GWD 0.00000 0.00000

rs302668 ITU & LWK 0.00063 0.00012

rs302668 ITU & MSL 0.00000 0.00000

rs302668 ITU & YRI 0.00006 0.00004

rs302668 ITU & PUR 0.73458 0.00242

rs302668 ITU & CLM 0.11638 0.00237

rs302668 ITU & PEL 0.55804 0.00354

rs302668 ITU & MXL 0.00269 0.00029

rs302668 ITU & CHS 0.82065 0.00158

rs302668 ITU & CDX 0.01295 0.00089

rs302668 ITU & KHV 0.00973 0.00067

rs302668 ITU & CHB 0.81935 0.00162

rs302668 ITU & JPT 0.08956 0.00243

rs302668 ITU & CEU 0.05153 0.00175

rs302668 ITU & FIN 0.00026 0.00009

rs302668 ITU & GBR 0.36237 0.00322

rs302668 ITU & IBS 0.13546 0.00308

rs302668 ITU & TSI 0.16293 0.00330

rs302668 ITU & BEB 1.00000 0.00000

rs302668 ITU & GIH 0.02077 0.00100

rs302668 PJL & ACB 0.00134 0.00018

rs302668 PJL & ASW 0.02276 0.00091

rs302668 PJL & ESN 0.00068 0.00012

rs302668 PJL & GWD 0.00000 0.00000

rs302668 PJL & LWK 0.00118 0.00015

rs302668 PJL & MSL 0.00006 0.00004

rs302668 PJL & YRI 0.00009 0.00003

rs302668 PJL & PUR 1.00000 0.00000

rs302668 PJL & CLM 0.17408 0.00302

rs302668 PJL & PEL 0.46676 0.00339

rs302668 PJL & MXL 0.00177 0.00023

rs302668 PJL & CHS 1.00000 0.00000

rs302668 PJL & CDX 0.00796 0.00064

rs302668 PJL & KHV 0.00485 0.00042

rs302668 PJL & CHB 1.00000 0.00000

rs302668 PJL & JPT 0.04998 0.00199

rs302668 PJL & CEU 0.02780 0.00139

rs302668 PJL & FIN 0.00024 0.00010

rs302668 PJL & GBR 0.30042 0.00335

rs302668 PJL & IBS 0.09932 0.00205

rs302668 PJL & TSI 0.12694 0.00248

rs302668 PJL & BEB 0.90535 0.00083

rs302668 PJL & GIH 0.04747 0.00158

rs302668 PJL & ITU 0.90724 0.00094

rs302668 STU & ACB 0.09369 0.00214

rs302668 STU & ASW 0.36051 0.00307

rs302668 STU & ESN 0.07228 0.00185

rs302668 STU & GWD 0.00001 0.00000

rs302668 STU & LWK 0.09547 0.00189

rs302668 STU & MSL 0.01052 0.00053

rs302668 STU & YRI 0.02374 0.00091

rs302668 STU & PUR 0.11808 0.00223

rs302668 STU & CLM 0.89511 0.00091

rs302668 STU & PEL 0.01973 0.00087

rs302668 STU & MXL 0.00001 0.00001

rs302668 STU & CHS 0.11478 0.00215

rs302668 STU & CDX 0.00000 0.00000

rs302668 STU & KHV 0.00000 0.00000

rs302668 STU & CHB 0.11494 0.00268

rs302668 STU & JPT 0.00034 0.00012

rs302668 STU & CEU 0.00028 0.00010

rs302668 STU & FIN 0.00000 0.00000

rs302668 STU & GBR 0.00548 0.00039

rs302668 STU & IBS 0.00062 0.00011

rs302668 STU & TSI 0.00097 0.00017

rs302668 STU & BEB 0.10422 0.00223

rs302668 STU & GIH 0.69966 0.00194

rs302668 STU & ITU 0.07137 0.00175

rs302668 STU & PJL 0.11151 0.00237

Locus: rs9268856

=================================

Pop Alleles:

-----------------------------------------------------------

1 2 Total

ACB 68 124 192

ASW 40 82 122

ESN 80 118 198

GWD 70 156 226

LWK 70 128 198

MSL 76 94 170

YRI 101 115 216

PUR 56 152 208

CLM 60 128 188

PEL 45 125 170

MXL 31 97 128

CHS 47 163 210

CDX 40 146 186

KHV 24 174 198

CHB 73 133 206

JPT 79 129 208

CEU 34 164 198

FIN 60 138 198

GBR 48 134 182

IBS 49 165 214

TSI 56 158 214

BEB 50 122 172

GIH 37 169 206

ITU 67 137 204

PJL 46 146 192

STU 54 150 204

Total: 14613547 5008

Locus Population pair P-Value S.E.

----------- --------------------- ------- -------

rs9268856 ASW & ACB 0.71334 0.00217

rs9268856 ESN & ACB 0.34509 0.00406

rs9268856 ESN & ASW 0.19884 0.00313

rs9268856 GWD & ACB 0.34699 0.00363

rs9268856 GWD & ASW 0.80835 0.00152

rs9268856 GWD & ESN 0.05055 0.00191

rs9268856 LWK & ACB 1.00000 0.00000

rs9268856 LWK & ASW 0.71894 0.00208

rs9268856 LWK & ESN 0.35326 0.00369

rs9268856 LWK & GWD 0.34769 0.00366

rs9268856 MSL & ACB 0.08563 0.00217

rs9268856 MSL & ASW 0.05417 0.00174

rs9268856 MSL & ESN 0.45540 0.00380

rs9268856 MSL & GWD 0.00571 0.00060

rs9268856 MSL & LWK 0.07267 0.00200

rs9268856 YRI & ACB 0.02146 0.00120

rs9268856 YRI & ASW 0.01591 0.00093

rs9268856 YRI & ESN 0.19792 0.00365

rs9268856 YRI & GWD 0.00094 0.00017

rs9268856 YRI & LWK 0.02237 0.00106

rs9268856 YRI & MSL 0.75573 0.00248

rs9268856 PUR & ACB 0.08037 0.00258

rs9268856 PUR & ASW 0.31654 0.00339

rs9268856 PUR & ESN 0.00464 0.00056

rs9268856 PUR & GWD 0.39765 0.00379

rs9268856 PUR & LWK 0.07153 0.00195

rs9268856 PUR & MSL 0.00047 0.00019

rs9268856 PUR & YRI 0.00004 0.00002

rs9268856 CLM & ACB 0.51398 0.00309

rs9268856 CLM & ASW 0.90075 0.00093

rs9268856 CLM & ESN 0.09151 0.00218

rs9268856 CLM & GWD 0.91548 0.00089

rs9268856 CLM & LWK 0.51805 0.00331

rs9268856 CLM & MSL 0.01491 0.00091

rs9268856 CLM & YRI 0.00358 0.00042

rs9268856 CLM & PUR 0.32077 0.00337

rs9268856 PEL & ACB 0.07269 0.00230

rs9268856 PEL & ASW 0.29720 0.00302

rs9268856 PEL & ESN 0.00518 0.00047

rs9268856 PEL & GWD 0.37429 0.00359

rs9268856 PEL & LWK 0.07184 0.00199

rs9268856 PEL & MSL 0.00038 0.00009

rs9268856 PEL & YRI 0.00008 0.00004

rs9268856 PEL & PUR 1.00000 0.00000

rs9268856 PEL & CLM 0.29232 0.00335

rs9268856 MXL & ACB 0.03445 0.00118

rs9268856 MXL & ASW 0.16132 0.00252

rs9268856 MXL & ESN 0.00353 0.00043

rs9268856 MXL & GWD 0.17963 0.00342

rs9268856 MXL & LWK 0.03923 0.00136

rs9268856 MXL & MSL 0.00015 0.00007

rs9268856 MXL & YRI 0.00004 0.00004

rs9268856 MXL & PUR 0.60733 0.00274

rs9268856 MXL & CLM 0.17002 0.00271

rs9268856 MXL & PEL 0.69272 0.00231

rs9268856 CHS & ACB 0.00462 0.00046

rs9268856 CHS & ASW 0.05246 0.00135

rs9268856 CHS & ESN 0.00011 0.00005

rs9268856 CHS & GWD 0.05068 0.00173

rs9268856 CHS & LWK 0.00516 0.00047

rs9268856 CHS & MSL 0.00000 0.00000

rs9268856 CHS & YRI 0.00000 0.00000

rs9268856 CHS & PUR 0.30987 0.00345

rs9268856 CHS & CLM 0.04588 0.00173

rs9268856 CHS & PEL 0.39700 0.00256

rs9268856 CHS & MXL 0.78968 0.00152

rs9268856 CDX & ACB 0.00305 0.00034

rs9268856 CDX & ASW 0.03266 0.00107

rs9268856 CDX & ESN 0.00004 0.00003

rs9268856 CDX & GWD 0.03197 0.00138

rs9268856 CDX & LWK 0.00377 0.00045

rs9268856 CDX & MSL 0.00000 0.00000

rs9268856 CDX & YRI 0.00000 0.00000

rs9268856 CDX & PUR 0.23645 0.00345

rs9268856 CDX & CLM 0.02605 0.00104

rs9268856 CDX & PEL 0.32291 0.00297

rs9268856 CDX & MXL 0.58628 0.00243

rs9268856 CDX & CHS 0.90381 0.00100

rs9268856 KHV & ACB 0.00000 0.00000

rs9268856 KHV & ASW 0.00000 0.00000

rs9268856 KHV & ESN 0.00000 0.00000

rs9268856 KHV & GWD 0.00000 0.00000

rs9268856 KHV & LWK 0.00000 0.00000

rs9268856 KHV & MSL 0.00000 0.00000

rs9268856 KHV & YRI 0.00000 0.00000

rs9268856 KHV & PUR 0.00012 0.00006

rs9268856 KHV & CLM 0.00000 0.00000

rs9268856 KHV & PEL 0.00033 0.00007

rs9268856 KHV & MXL 0.00594 0.00041

rs9268856 KHV & CHS 0.00955 0.00058

rs9268856 KHV & CDX 0.01887 0.00091

rs9268856 CHB & ACB 1.00000 0.00000

rs9268856 CHB & ASW 0.63691 0.00262

rs9268856 CHB & ESN 0.30329 0.00393

rs9268856 CHB & GWD 0.35720 0.00444

rs9268856 CHB & LWK 1.00000 0.00000

rs9268856 CHB & MSL 0.06878 0.00207

rs9268856 CHB & YRI 0.02018 0.00100

rs9268856 CHB & PUR 0.07279 0.00237

rs9268856 CHB & CLM 0.52205 0.00356

rs9268856 CHB & PEL 0.07165 0.00216

rs9268856 CHB & MXL 0.04073 0.00142

rs9268856 CHB & CHS 0.00333 0.00039

rs9268856 CHB & CDX 0.00219 0.00031

rs9268856 CHB & KHV 0.00000 0.00000

rs9268856 JPT & ACB 0.60810 0.00322

rs9268856 JPT & ASW 0.33742 0.00362

rs9268856 JPT & ESN 0.69021 0.00246

rs9268856 JPT & GWD 0.12921 0.00269

rs9268856 JPT & LWK 0.60432 0.00297

rs9268856 JPT & MSL 0.20234 0.00334

rs9268856 JPT & YRI 0.07709 0.00234

rs9268856 JPT & PUR 0.01959 0.00096

rs9268856 JPT & CLM 0.25013 0.00381

rs9268856 JPT & PEL 0.02090 0.00106

rs9268856 JPT & MXL 0.00898 0.00061

rs9268856 JPT & CHS 0.00062 0.00013

rs9268856 JPT & CDX 0.00027 0.00007

rs9268856 JPT & KHV 0.00000 0.00000

rs9268856 JPT & CHB 0.61362 0.00335

rs9268856 CEU & ACB 0.00007 0.00003

rs9268856 CEU & ASW 0.00148 0.00021

rs9268856 CEU & ESN 0.00000 0.00000

rs9268856 CEU & GWD 0.00116 0.00020

rs9268856 CEU & LWK 0.00006 0.00004

rs9268856 CEU & MSL 0.00000 0.00000

rs9268856 CEU & YRI 0.00000 0.00000

rs9268856 CEU & PUR 0.02431 0.00102

rs9268856 CEU & CLM 0.00090 0.00016

rs9268856 CEU & PEL 0.04276 0.00144

rs9268856 CEU & MXL 0.15456 0.00234

rs9268856 CEU & CHS 0.21532 0.00281

rs9268856 CEU & CDX 0.29896 0.00331

rs9268856 CEU & KHV 0.19916 0.00281

rs9268856 CEU & CHB 0.00001 0.00001

rs9268856 CEU & JPT 0.00000 0.00000

rs9268856 FIN & ACB 0.32710 0.00357

rs9268856 FIN & ASW 0.71218 0.00212

rs9268856 FIN & ESN 0.04382 0.00166

rs9268856 FIN & GWD 0.91534 0.00086

rs9268856 FIN & LWK 0.33400 0.00383

rs9268856 FIN & MSL 0.00516 0.00048

rs9268856 FIN & YRI 0.00057 0.00016

rs9268856 FIN & PUR 0.50771 0.00364

rs9268856 FIN & CLM 0.74211 0.00236

rs9268856 FIN & PEL 0.41993 0.00377

rs9268856 FIN & MXL 0.26059 0.00316

rs9268856 FIN & CHS 0.07392 0.00217

rs9268856 FIN & CDX 0.06439 0.00206

rs9268856 FIN & KHV 0.00002 0.00002

rs9268856 FIN & CHB 0.28705 0.00377

rs9268856 FIN & JPT 0.11896 0.00266

rs9268856 FIN & CEU 0.00343 0.00041

rs9268856 GBR & ACB 0.07289 0.00226

rs9268856 GBR & ASW 0.24655 0.00307

rs9268856 GBR & ESN 0.00419 0.00049

rs9268856 GBR & GWD 0.32439 0.00349

rs9268856 GBR & LWK 0.06053 0.00204

rs9268856 GBR & MSL 0.00036 0.00011

rs9268856 GBR & YRI 0.00002 0.00001

rs9268856 GBR & PUR 0.90847 0.00085

rs9268856 GBR & CLM 0.25462 0.00289

rs9268856 GBR & PEL 1.00000 0.00000

rs9268856 GBR & MXL 0.69159 0.00222

rs9268856 GBR & CHS 0.40868 0.00384

rs9268856 GBR & CDX 0.33416 0.00300

rs9268856 GBR & KHV 0.00073 0.00015

rs9268856 GBR & CHB 0.06254 0.00217

rs9268856 GBR & JPT 0.01796 0.00100

rs9268856 GBR & CEU 0.03390 0.00115

rs9268856 GBR & FIN 0.42673 0.00322

rs9268856 IBS & ACB 0.00646 0.00057

rs9268856 IBS & ASW 0.05352 0.00162

rs9268856 IBS & ESN 0.00019 0.00010

rs9268856 IBS & GWD 0.06812 0.00207

rs9268856 IBS & LWK 0.00586 0.00049

rs9268856 IBS & MSL 0.00000 0.00000

rs9268856 IBS & YRI 0.00000 0.00000

rs9268856 IBS & PUR 0.36816 0.00340

rs9268856 IBS & CLM 0.04340 0.00167

rs9268856 IBS & PEL 0.47524 0.00366

rs9268856 IBS & MXL 0.79001 0.00155

rs9268856 IBS & CHS 0.90776 0.00083

rs9268856 IBS & CDX 0.80517 0.00163

rs9268856 IBS & KHV 0.00508 0.00046

rs9268856 IBS & CHB 0.00576 0.00057

rs9268856 IBS & JPT 0.00112 0.00020

rs9268856 IBS & CEU 0.17647 0.00280

rs9268856 IBS & FIN 0.09187 0.00245

rs9268856 IBS & GBR 0.48093 0.00318

rs9268856 TSI & ACB 0.05120 0.00177

rs9268856 TSI & ASW 0.21335 0.00271

rs9268856 TSI & ESN 0.00255 0.00033

rs9268856 TSI & GWD 0.29275 0.00384

rs9268856 TSI & LWK 0.05582 0.00180

rs9268856 TSI & MSL 0.00009 0.00003

rs9268856 TSI & YRI 0.00000 0.00000

rs9268856 TSI & PUR 0.91182 0.00088

rs9268856 TSI & CLM 0.22668 0.00319

rs9268856 TSI & PEL 1.00000 0.00000

rs9268856 TSI & MXL 0.70595 0.00214

rs9268856 TSI & CHS 0.36918 0.00359

rs9268856 TSI & CDX 0.28824 0.00371

rs9268856 TSI & KHV 0.00022 0.00006

rs9268856 TSI & CHB 0.04362 0.00170

rs9268856 TSI & JPT 0.01181 0.00069

rs9268856 TSI & CEU 0.03308 0.00135

rs9268856 TSI & FIN 0.38030 0.00361

rs9268856 TSI & GBR 1.00000 0.00000

rs9268856 TSI & IBS 0.50160 0.00358

rs9268856 BEB & ACB 0.21971 0.00360

rs9268856 BEB & ASW 0.52169 0.00273

rs9268856 BEB & ESN 0.02930 0.00145

rs9268856 BEB & GWD 0.74293 0.00221

rs9268856 BEB & LWK 0.21707 0.00295

rs9268856 BEB & MSL 0.00308 0.00036

rs9268856 BEB & YRI 0.00047 0.00015

rs9268856 BEB & PUR 0.64520 0.00285

rs9268856 BEB & CLM 0.56946 0.00303

rs9268856 BEB & PEL 0.62972 0.00291

rs9268856 BEB & MXL 0.36310 0.00311

rs9268856 BEB & CHS 0.15978 0.00286

rs9268856 BEB & CDX 0.11563 0.00232

rs9268856 BEB & KHV 0.00010 0.00005

rs9268856 BEB & CHB 0.22511 0.00319

rs9268856 BEB & JPT 0.08134 0.00216

rs9268856 BEB & CEU 0.00831 0.00064

rs9268856 BEB & FIN 0.81914 0.00165

rs9268856 BEB & GBR 0.63217 0.00301

rs9268856 BEB & IBS 0.19747 0.00330

rs9268856 BEB & TSI 0.56340 0.00346

rs9268856 GIH & ACB 0.00003 0.00001

rs9268856 GIH & ASW 0.00246 0.00025

rs9268856 GIH & ESN 0.00000 0.00000

rs9268856 GIH & GWD 0.00199 0.00035

rs9268856 GIH & LWK 0.00008 0.00004

rs9268856 GIH & MSL 0.00000 0.00000

rs9268856 GIH & YRI 0.00000 0.00000

rs9268856 GIH & PUR 0.03257 0.00128

rs9268856 GIH & CLM 0.00132 0.00020

rs9268856 GIH & PEL 0.05988 0.00167

rs9268856 GIH & MXL 0.21172 0.00234

rs9268856 GIH & CHS 0.27601 0.00327

rs9268856 GIH & CDX 0.44470 0.00281

rs9268856 GIH & KHV 0.12582 0.00223

rs9268856 GIH & CHB 0.00006 0.00003

rs9268856 GIH & JPT 0.00000 0.00000

rs9268856 GIH & CEU 0.89511 0.00088

rs9268856 GIH & FIN 0.00504 0.00050

rs9268856 GIH & GBR 0.04909 0.00186

rs9268856 GIH & IBS 0.23153 0.00281

rs9268856 GIH & TSI 0.04830 0.00152

rs9268856 GIH & BEB 0.01370 0.00074

rs9268856 ITU & ACB 0.59703 0.00332

rs9268856 ITU & ASW 1.00000 0.00000

rs9268856 ITU & ESN 0.12007 0.00311

rs9268856 ITU & GWD 0.67790 0.00305

rs9268856 ITU & LWK 0.59883 0.00319

rs9268856 ITU & MSL 0.02425 0.00109

rs9268856 ITU & YRI 0.00365 0.00043

rs9268856 ITU & PUR 0.19769 0.00368

rs9268856 ITU & CLM 0.91182 0.00085

rs9268856 ITU & PEL 0.20996 0.00343

rs9268856 ITU & MXL 0.11003 0.00243

rs9268856 ITU & CHS 0.02052 0.00109

rs9268856 ITU & CDX 0.01280 0.00082

rs9268856 ITU & KHV 0.00000 0.00000

rs9268856 ITU & CHB 0.60068 0.00335

rs9268856 ITU & JPT 0.30506 0.00396

rs9268856 ITU & CEU 0.00037 0.00011

rs9268856 ITU & FIN 0.58881 0.00282

rs9268856 ITU & GBR 0.18325 0.00316

rs9268856 ITU & IBS 0.02876 0.00129

rs9268856 ITU & TSI 0.16388 0.00297

rs9268856 ITU & BEB 0.43439 0.00338

rs9268856 ITU & GIH 0.00063 0.00015

rs9268856 PJL & ACB 0.01962 0.00102

rs9268856 PJL & ASW 0.09421 0.00224

rs9268856 PJL & ESN 0.00044 0.00012

rs9268856 PJL & GWD 0.12750 0.00266

rs9268856 PJL & LWK 0.01393 0.00096

rs9268856 PJL & MSL 0.00002 0.00001

rs9268856 PJL & YRI 0.00000 0.00000

rs9268856 PJL & PUR 0.56733 0.00310

rs9268856 PJL & CLM 0.08693 0.00237

rs9268856 PJL & PEL 0.62929 0.00269

rs9268856 PJL & MXL 1.00000 0.00000

rs9268856 PJL & CHS 0.72516 0.00213

rs9268856 PJL & CDX 0.62482 0.00279

rs9268856 PJL & KHV 0.00250 0.00026

rs9268856 PJL & CHB 0.01667 0.00091

rs9268856 PJL & JPT 0.00319 0.00036

rs9268856 PJL & CEU 0.10991 0.00248

rs9268856 PJL & FIN 0.17746 0.00318

rs9268856 PJL & GBR 0.63633 0.00267

rs9268856 PJL & IBS 0.81583 0.00160

rs9268856 PJL & TSI 0.65438 0.00275

rs9268856 PJL & BEB 0.28297 0.00296

rs9268856 PJL & GIH 0.17489 0.00283

rs9268856 PJL & ITU 0.06176 0.00185

rs9268856 STU & ACB 0.06226 0.00198

rs9268856 STU & ASW 0.25527 0.00291

rs9268856 STU & ESN 0.00380 0.00044

rs9268856 STU & GWD 0.33760 0.00357

rs9268856 STU & LWK 0.06469 0.00190

rs9268856 STU & MSL 0.00020 0.00006

rs9268856 STU & YRI 0.00004 0.00003

rs9268856 STU & PUR 1.00000 0.00000

rs9268856 STU & CLM 0.26176 0.00318

rs9268856 STU & PEL 1.00000 0.00000

rs9268856 STU & MXL 0.70180 0.00206

rs9268856 STU & CHS 0.35972 0.00321

rs9268856 STU & CDX 0.28995 0.00367

rs9268856 STU & KHV 0.00029 0.00007

rs9268856 STU & CHB 0.05557 0.00187

rs9268856 STU & JPT 0.01338 0.00086

rs9268856 STU & CEU 0.03060 0.00129

rs9268856 STU & FIN 0.43343 0.00339

rs9268856 STU & GBR 1.00000 0.00000

rs9268856 STU & IBS 0.42871 0.00299

rs9268856 STU & TSI 1.00000 0.00000

rs9268856 STU & BEB 0.64556 0.00297

rs9268856 STU & GIH 0.04223 0.00141

rs9268856 STU & ITU 0.19373 0.00335

rs9268856 STU & PJL 0.64520 0.00270

Locus: rs906175

=================================

Pop Alleles:

-----------------------------------------------------------

2 4 Total

ACB 184 8 192

ASW 107 15 122

ESN 197 1 198

GWD 226 0 226

LWK 197 1 198

MSL 170 0 170

YRI 214 2 216

PUR 86 122 208

CLM 52 136 188

PEL 62 108 170

MXL 70 58 128

CHS 91 119 210

CDX 63 123 186

KHV 65 133 198

CHB 94 112 206

JPT 60 148 208

CEU 112 86 198

FIN 102 96 198

GBR 101 81 182

IBS 132 82 214

TSI 110 104 214

BEB 74 98 172

GIH 78 128 206

ITU 86 118 204

PJL 78 114 192

STU 93 111 204

Total: 29042104 5008

Locus Population pair P-Value S.E.

----------- --------------------- ------- -------

rs906175 ASW & ACB 0.01326 0.00042

rs906175 ESN & ACB 0.01848 0.00038

rs906175 ESN & ASW 0.00000 0.00000

rs906175 GWD & ACB 0.00176 0.00010

rs906175 GWD & ASW 0.00000 0.00000

rs906175 GWD & ESN 0.46770 0.00076

rs906175 LWK & ACB 0.01827 0.00037

rs906175 LWK & ASW 0.00000 0.00000

rs906175 LWK & ESN 1.00000 0.00000

rs906175 LWK & GWD 0.46733 0.00076

rs906175 MSL & ACB 0.00778 0.00023

rs906175 MSL & ASW 0.00000 0.00000

rs906175 MSL & ESN 1.00000 0.00000

rs906175 MSL & GWD No table

rs906175 MSL & LWK 1.00000 0.00000

rs906175 YRI & ACB 0.04990 0.00069

rs906175 YRI & ASW 0.00000 0.00000

rs906175 YRI & ESN 1.00000 0.00000

rs906175 YRI & GWD 0.23804 0.00094

rs906175 YRI & LWK 1.00000 0.00000

rs906175 YRI & MSL 0.50577 0.00072

rs906175 PUR & ACB 0.00000 0.00000

rs906175 PUR & ASW 0.00000 0.00000

rs906175 PUR & ESN 0.00000 0.00000

rs906175 PUR & GWD 0.00000 0.00000

rs906175 PUR & LWK 0.00000 0.00000

rs906175 PUR & MSL 0.00000 0.00000

rs906175 PUR & YRI 0.00000 0.00000

rs906175 CLM & ACB 0.00000 0.00000

rs906175 CLM & ASW 0.00000 0.00000

rs906175 CLM & ESN 0.00000 0.00000

rs906175 CLM & GWD 0.00000 0.00000

rs906175 CLM & LWK 0.00000 0.00000

rs906175 CLM & MSL 0.00000 0.00000

rs906175 CLM & YRI 0.00000 0.00000

rs906175 CLM & PUR 0.00417 0.00040

rs906175 PEL & ACB 0.00000 0.00000

rs906175 PEL & ASW 0.00000 0.00000

rs906175 PEL & ESN 0.00000 0.00000

rs906175 PEL & GWD 0.00000 0.00000

rs906175 PEL & LWK 0.00000 0.00000

rs906175 PEL & MSL 0.00000 0.00000

rs906175 PEL & YRI 0.00000 0.00000

rs906175 PEL & PUR 0.34707 0.00405

rs906175 PEL & CLM 0.08790 0.00234

rs906175 MXL & ACB 0.00000 0.00000

rs906175 MXL & ASW 0.00000 0.00000

rs906175 MXL & ESN 0.00000 0.00000

rs906175 MXL & GWD 0.00000 0.00000

rs906175 MXL & LWK 0.00000 0.00000

rs906175 MXL & MSL 0.00000 0.00000

rs906175 MXL & YRI 0.00000 0.00000

rs906175 MXL & PUR 0.01961 0.00101

rs906175 MXL & CLM 0.00000 0.00000

rs906175 MXL & PEL 0.00220 0.00029

rs906175 CHS & ACB 0.00000 0.00000

rs906175 CHS & ASW 0.00000 0.00000

rs906175 CHS & ESN 0.00000 0.00000

rs906175 CHS & GWD 0.00000 0.00000

rs906175 CHS & LWK 0.00000 0.00000

rs906175 CHS & MSL 0.00000 0.00000

rs906175 CHS & YRI 0.00000 0.00000

rs906175 CHS & PUR 0.69333 0.00300

rs906175 CHS & CLM 0.00124 0.00023

rs906175 CHS & PEL 0.21193 0.00344

rs906175 CHS & MXL 0.04129 0.00146

rs906175 CDX & ACB 0.00000 0.00000

rs906175 CDX & ASW 0.00000 0.00000

rs906175 CDX & ESN 0.00000 0.00000

rs906175 CDX & GWD 0.00000 0.00000

rs906175 CDX & LWK 0.00000 0.00000

rs906175 CDX & MSL 0.00000 0.00000

rs906175 CDX & YRI 0.00000 0.00000

rs906175 CDX & PUR 0.13802 0.00301

rs906175 CDX & CLM 0.21354 0.00299

rs906175 CDX & PEL 0.65735 0.00281

rs906175 CDX & MXL 0.00038 0.00013

rs906175 CDX & CHS 0.06309 0.00208

rs906175 KHV & ACB 0.00000 0.00000

rs906175 KHV & ASW 0.00000 0.00000

rs906175 KHV & ESN 0.00000 0.00000

rs906175 KHV & GWD 0.00000 0.00000

rs906175 KHV & LWK 0.00000 0.00000

rs906175 KHV & MSL 0.00000 0.00000

rs906175 KHV & YRI 0.00000 0.00000

rs906175 KHV & PUR 0.08275 0.00221

rs906175 KHV & CLM 0.32479 0.00343

rs906175 KHV & PEL 0.50682 0.00310

rs906175 KHV & MXL 0.00019 0.00008

rs906175 KHV & CHS 0.03122 0.00157

rs906175 KHV & CDX 0.91369 0.00073

rs906175 CHB & ACB 0.00000 0.00000

rs906175 CHB & ASW 0.00000 0.00000

rs906175 CHB & ESN 0.00000 0.00000

rs906175 CHB & GWD 0.00000 0.00000

rs906175 CHB & LWK 0.00000 0.00000

rs906175 CHB & MSL 0.00000 0.00000

rs906175 CHB & YRI 0.00000 0.00000

rs906175 CHB & PUR 0.43093 0.00385

rs906175 CHB & CLM 0.00033 0.00011

rs906175 CHB & PEL 0.07369 0.00240

rs906175 CHB & MXL 0.11642 0.00253

rs906175 CHB & CHS 0.68962 0.00293

rs906175 CHB & CDX 0.01965 0.00112

rs906175 CHB & KHV 0.00963 0.00074

rs906175 JPT & ACB 0.00000 0.00000

rs906175 JPT & ASW 0.00000 0.00000

rs906175 JPT & ESN 0.00000 0.00000

rs906175 JPT & GWD 0.00000 0.00000

rs906175 JPT & LWK 0.00000 0.00000

rs906175 JPT & MSL 0.00000 0.00000

rs906175 JPT & YRI 0.00000 0.00000

rs906175 JPT & PUR 0.00946 0.00068

rs906175 JPT & CLM 0.82227 0.00179

rs906175 JPT & PEL 0.12526 0.00304

rs906175 JPT & MXL 0.00001 0.00001

rs906175 JPT & CHS 0.00225 0.00031

rs906175 JPT & CDX 0.32832 0.00370

rs906175 JPT & KHV 0.39088 0.00373

rs906175 JPT & CHB 0.00038 0.00008

rs906175 CEU & ACB 0.00000 0.00000

rs906175 CEU & ASW 0.00000 0.00000

rs906175 CEU & ESN 0.00000 0.00000

rs906175 CEU & GWD 0.00000 0.00000

rs906175 CEU & LWK 0.00000 0.00000

rs906175 CEU & MSL 0.00000 0.00000

rs906175 CEU & YRI 0.00000 0.00000

rs906175 CEU & PUR 0.00227 0.00028

rs906175 CEU & CLM 0.00000 0.00000

rs906175 CEU & PEL 0.00017 0.00006

rs906175 CEU & MXL 0.81921 0.00179

rs906175 CEU & CHS 0.00797 0.00058

rs906175 CEU & CDX 0.00000 0.00000

rs906175 CEU & KHV 0.00000 0.00000

rs906175 CEU & CHB 0.02729 0.00145

rs906175 CEU & JPT 0.00000 0.00000

rs906175 FIN & ACB 0.00000 0.00000

rs906175 FIN & ASW 0.00000 0.00000

rs906175 FIN & ESN 0.00000 0.00000

rs906175 FIN & GWD 0.00000 0.00000

rs906175 FIN & LWK 0.00000 0.00000

rs906175 FIN & MSL 0.00000 0.00000

rs906175 FIN & YRI 0.00000 0.00000

rs906175 FIN & PUR 0.04550 0.00167

rs906175 FIN & CLM 0.00000 0.00000

rs906175 FIN & PEL 0.00402 0.00049

rs906175 FIN & MXL 0.64969 0.00267

rs906175 FIN & CHS 0.11334 0.00326

rs906175 FIN & CDX 0.00062 0.00014

rs906175 FIN & KHV 0.00027 0.00011

rs906175 FIN & CHB 0.26948 0.00381

rs906175 FIN & JPT 0.00000 0.00000

rs906175 FIN & CEU 0.36337 0.00359

rs906175 GBR & ACB 0.00000 0.00000

rs906175 GBR & ASW 0.00000 0.00000

rs906175 GBR & ESN 0.00000 0.00000

rs906175 GBR & GWD 0.00000 0.00000

rs906175 GBR & LWK 0.00000 0.00000

rs906175 GBR & MSL 0.00000 0.00000

rs906175 GBR & YRI 0.00000 0.00000

rs906175 GBR & PUR 0.00658 0.00066

rs906175 GBR & CLM 0.00000 0.00000

rs906175 GBR & PEL 0.00041 0.00010

rs906175 GBR & MXL 0.90790 0.00090

rs906175 GBR & CHS 0.01950 0.00102

rs906175 GBR & CDX 0.00003 0.00003

rs906175 GBR & KHV 0.00000 0.00000

rs906175 GBR & CHB 0.05133 0.00210

rs906175 GBR & JPT 0.00000 0.00000

rs906175 GBR & CEU 0.83485 0.00161

rs906175 GBR & FIN 0.47666 0.00414

rs906175 IBS & ACB 0.00000 0.00000

rs906175 IBS & ASW 0.00000 0.00000

rs906175 IBS & ESN 0.00000 0.00000

rs906175 IBS & GWD 0.00000 0.00000

rs906175 IBS & LWK 0.00000 0.00000

rs906175 IBS & MSL 0.00000 0.00000

rs906175 IBS & YRI 0.00000 0.00000

rs906175 IBS & PUR 0.00003 0.00002

rs906175 IBS & CLM 0.00000 0.00000

rs906175 IBS & PEL 0.00000 0.00000

rs906175 IBS & MXL 0.21485 0.00312

rs906175 IBS & CHS 0.00015 0.00005

rs906175 IBS & CDX 0.00000 0.00000

rs906175 IBS & KHV 0.00000 0.00000

rs906175 IBS & CHB 0.00115 0.00026

rs906175 IBS & JPT 0.00000 0.00000

rs906175 IBS & CEU 0.31229 0.00392

rs906175 IBS & FIN 0.04626 0.00180

rs906175 IBS & GBR 0.22570 0.00366

rs906175 TSI & ACB 0.00000 0.00000

rs906175 TSI & ASW 0.00000 0.00000

rs906175 TSI & ESN 0.00000 0.00000

rs906175 TSI & GWD 0.00000 0.00000

rs906175 TSI & LWK 0.00000 0.00000

rs906175 TSI & MSL 0.00000 0.00000

rs906175 TSI & YRI 0.00000 0.00000

rs906175 TSI & PUR 0.04048 0.00202

rs906175 TSI & CLM 0.00000 0.00000

rs906175 TSI & PEL 0.00431 0.00044

rs906175 TSI & MXL 0.57272 0.00295

rs906175 TSI & CHS 0.09645 0.00288

rs906175 TSI & CDX 0.00063 0.00016

rs906175 TSI & KHV 0.00008 0.00004

rs906175 TSI & CHB 0.23984 0.00378

rs906175 TSI & JPT 0.00000 0.00000

rs906175 TSI & CEU 0.32504 0.00402

rs906175 TSI & FIN 1.00000 0.00000

rs906175 TSI & GBR 0.41824 0.00371

rs906175 TSI & IBS 0.03971 0.00179

rs906175 BEB & ACB 0.00000 0.00000

rs906175 BEB & ASW 0.00000 0.00000

rs906175 BEB & ESN 0.00000 0.00000

rs906175 BEB & GWD 0.00000 0.00000

rs906175 BEB & LWK 0.00000 0.00000

rs906175 BEB & MSL 0.00000 0.00000

rs906175 BEB & YRI 0.00000 0.00000

rs906175 BEB & PUR 0.75862 0.00241

rs906175 BEB & CLM 0.00379 0.00054

rs906175 BEB & PEL 0.22963 0.00319

rs906175 BEB & MXL 0.05021 0.00172

rs906175 BEB & CHS 1.00000 0.00000

rs906175 BEB & CDX 0.07991 0.00226

rs906175 BEB & KHV 0.05243 0.00197

rs906175 BEB & CHB 0.67335 0.00338

rs906175 BEB & JPT 0.00589 0.00058

rs906175 BEB & CEU 0.01136 0.00072

rs906175 BEB & FIN 0.11934 0.00249

rs906175 BEB & GBR 0.01887 0.00113

rs906175 BEB & IBS 0.00023 0.00008

rs906175 BEB & TSI 0.12376 0.00301

rs906175 GIH & ACB 0.00000 0.00000

rs906175 GIH & ASW 0.00000 0.00000

rs906175 GIH & ESN 0.00000 0.00000

rs906175 GIH & GWD 0.00000 0.00000

rs906175 GIH & LWK 0.00000 0.00000

rs906175 GIH & MSL 0.00000 0.00000

rs906175 GIH & YRI 0.00000 0.00000

rs906175 GIH & PUR 0.48866 0.00380

rs906175 GIH & CLM 0.03178 0.00152

rs906175 GIH & PEL 0.83175 0.00159

rs906175 GIH & MXL 0.00373 0.00039

rs906175 GIH & CHS 0.27122 0.00383

rs906175 GIH & CDX 0.45700 0.00405

rs906175 GIH & KHV 0.29739 0.00416

rs906175 GIH & CHB 0.13691 0.00304

rs906175 GIH & JPT 0.06090 0.00201

rs906175 GIH & CEU 0.00014 0.00007

rs906175 GIH & FIN 0.00692 0.00054

rs906175 GIH & GBR 0.00049 0.00013

rs906175 GIH & IBS 0.00000 0.00000

rs906175 GIH & TSI 0.00497 0.00053

rs906175 GIH & BEB 0.34274 0.00385

rs906175 ITU & ACB 0.00000 0.00000

rs906175 ITU & ASW 0.00000 0.00000

rs906175 ITU & ESN 0.00000 0.00000

rs906175 ITU & GWD 0.00000 0.00000

rs906175 ITU & LWK 0.00000 0.00000

rs906175 ITU & MSL 0.00000 0.00000

rs906175 ITU & YRI 0.00000 0.00000

rs906175 ITU & PUR 0.91766 0.00083

rs906175 ITU & CLM 0.00308 0.00038

rs906175 ITU & PEL 0.28877 0.00381

rs906175 ITU & MXL 0.02986 0.00111

rs906175 ITU & CHS 0.84169 0.00185

rs906175 ITU & CDX 0.09243 0.00237

rs906175 ITU & KHV 0.06513 0.00220

rs906175 ITU & CHB 0.48573 0.00431

rs906175 ITU & JPT 0.00560 0.00050

rs906175 ITU & CEU 0.00552 0.00058

rs906175 ITU & FIN 0.07171 0.00239

rs906175 ITU & GBR 0.01022 0.00077

rs906175 ITU & IBS 0.00015 0.00007

rs906175 ITU & TSI 0.06258 0.00192

rs906175 ITU & BEB 0.91815 0.00090

rs906175 ITU & GIH 0.41444 0.00383

rs906175 PJL & ACB 0.00000 0.00000

rs906175 PJL & ASW 0.00000 0.00000

rs906175 PJL & ESN 0.00000 0.00000

rs906175 PJL & GWD 0.00000 0.00000

rs906175 PJL & LWK 0.00000 0.00000

rs906175 PJL & MSL 0.00000 0.00000

rs906175 PJL & YRI 0.00000 0.00000

rs906175 PJL & PUR 0.92164 0.00074

rs906175 PJL & CLM 0.00992 0.00082

rs906175 PJL & PEL 0.45681 0.00412

rs906175 PJL & MXL 0.01490 0.00084

rs906175 PJL & CHS 0.61423 0.00326

rs906175 PJL & CDX 0.20550 0.00313

rs906175 PJL & KHV 0.11842 0.00305

rs906175 PJL & CHB 0.36294 0.00406

rs906175 PJL & JPT 0.01516 0.00085

rs906175 PJL & CEU 0.00175 0.00027

rs906175 PJL & FIN 0.03397 0.00155

rs906175 PJL & GBR 0.00567 0.00058

rs906175 PJL & IBS 0.00002 0.00002

rs906175 PJL & TSI 0.03727 0.00158

rs906175 PJL & BEB 0.67026 0.00300

rs906175 PJL & GIH 0.60746 0.00327

rs906175 PJL & ITU 0.76248 0.00264

rs906175 STU & ACB 0.00000 0.00000

rs906175 STU & ASW 0.00000 0.00000

rs906175 STU & ESN 0.00000 0.00000

rs906175 STU & GWD 0.00000 0.00000

rs906175 STU & LWK 0.00000 0.00000

rs906175 STU & MSL 0.00000 0.00000

rs906175 STU & YRI 0.00000 0.00000

rs906175 STU & PUR 0.42918 0.00395

rs906175 STU & CLM 0.00024 0.00009

rs906175 STU & PEL 0.09049 0.00264

rs906175 STU & MXL 0.11375 0.00270

rs906175 STU & CHS 0.69457 0.00281

rs906175 STU & CDX 0.02274 0.00132

rs906175 STU & KHV 0.00878 0.00059

rs906175 STU & CHB 1.00000 0.00000

rs906175 STU & JPT 0.00064 0.00014

rs906175 STU & CEU 0.02729 0.00140

rs906175 STU & FIN 0.27176 0.00400

rs906175 STU & GBR 0.05099 0.00183

rs906175 STU & IBS 0.00180 0.00027

rs906175 STU & TSI 0.24087 0.00380

rs906175 STU & BEB 0.67970 0.00238

rs906175 STU & GIH 0.14113 0.00355

rs906175 STU & ITU 0.55210 0.00371

rs906175 STU & PJL 0.36621 0.00418

Locus: rs17042852

=================================

Pop Alleles:

-----------------------------------------------------------

2 4 Total

ACB 0 192 192

ASW 0 122 122

ESN 0 198 198

GWD 0 226 226

LWK 0 198 198

MSL 0 170 170

YRI 5 211 216

PUR 9 199 208

CLM 0 188 188

PEL 4 166 170

MXL 3 125 128

CHS 14 196 210

CDX 11 175 186

KHV 16 182 198

CHB 9 197 206

JPT 27 181 208

CEU 6 192 198

FIN 5 193 198

GBR 1 181 182

IBS 14 200 214

TSI 20 194 214

BEB 7 165 172

GIH 8 198 206

ITU 8 196 204

PJL 8 184 192

STU 9 195 204

Total: 184 4824 5008

Locus Population pair P-Value S.E.

----------- --------------------- ------- -------

rs17042852 ASW & ACB No table

rs17042852 ESN & ACB No table

rs17042852 ESN & ASW No table

rs17042852 GWD & ACB No table

rs17042852 GWD & ASW No table

rs17042852 GWD & ESN No table

rs17042852 LWK & ACB No table

rs17042852 LWK & ASW No table

rs17042852 LWK & ESN No table

rs17042852 LWK & GWD No table

rs17042852 MSL & ACB No table

rs17042852 MSL & ASW No table

rs17042852 MSL & ESN No table

rs17042852 MSL & GWD No table

rs17042852 MSL & LWK No table

rs17042852 YRI & ACB 0.06309 0.00061

rs17042852 YRI & ASW 0.11048 0.00077

rs17042852 YRI & ESN 0.06156 0.00053

rs17042852 YRI & GWD 0.02714 0.00043

rs17042852 YRI & LWK 0.06126 0.00064

rs17042852 YRI & MSL 0.06969 0.00060

rs17042852 PUR & ACB 0.00358 0.00017

rs17042852 PUR & ASW 0.01697 0.00037

rs17042852 PUR & ESN 0.00354 0.00016

rs17042852 PUR & GWD 0.00111 0.00008

rs17042852 PUR & LWK 0.00341 0.00014

rs17042852 PUR & MSL 0.00497 0.00018

rs17042852 PUR & YRI 0.28492 0.00177

rs17042852 CLM & ACB No table

rs17042852 CLM & ASW No table

rs17042852 CLM & ESN No table

rs17042852 CLM & GWD No table

rs17042852 CLM & LWK No table

rs17042852 CLM & MSL No table

rs17042852 CLM & YRI 0.06454 0.00068

rs17042852 CLM & PUR 0.00403 0.00017

rs17042852 PEL & ACB 0.04816 0.00060

rs17042852 PEL & ASW 0.14154 0.00081

rs17042852 PEL & ESN 0.04404 0.00051

rs17042852 PEL & GWD 0.03322 0.00041

rs17042852 PEL & LWK 0.04492 0.00047

rs17042852 PEL & MSL 0.12273 0.00070

rs17042852 PEL & YRI 1.00000 0.00000

rs17042852 PEL & PUR 0.39534 0.00156

rs17042852 PEL & CLM 0.05015 0.00050

rs17042852 MXL & ACB 0.06235 0.00055

rs17042852 MXL & ASW 0.24718 0.00089

rs17042852 MXL & ESN 0.05909 0.00061

rs17042852 MXL & GWD 0.04645 0.00060

rs17042852 MXL & LWK 0.06000 0.00063

rs17042852 MXL & MSL 0.07797 0.00068

rs17042852 MXL & YRI 1.00000 0.00000

rs17042852 MXL & PUR 0.38711 0.00161

rs17042852 MXL & CLM 0.06628 0.00062

rs17042852 MXL & PEL 1.00000 0.00000

rs17042852 CHS & ACB 0.00010 0.00002

rs17042852 CHS & ASW 0.00139 0.00009

rs17042852 CHS & ESN 0.00009 0.00002

rs17042852 CHS & GWD 0.00006 0.00002

rs17042852 CHS & LWK 0.00010 0.00002

rs17042852 CHS & MSL 0.00022 0.00004

rs17042852 CHS & YRI 0.03399 0.00070

rs17042852 CHS & PUR 0.39218 0.00182

rs17042852 CHS & CLM 0.00012 0.00002

rs17042852 CHS & PEL 0.05452 0.00099

rs17042852 CHS & MXL 0.07991 0.00107

rs17042852 CDX & ACB 0.00030 0.00005

rs17042852 CDX & ASW 0.00388 0.00016

rs17042852 CDX & ESN 0.00024 0.00004

rs17042852 CDX & GWD 0.00011 0.00003

rs17042852 CDX & LWK 0.00027 0.00005

rs17042852 CDX & MSL 0.00092 0.00009

rs17042852 CDX & YRI 0.07638 0.00123

rs17042852 CDX & PUR 0.50011 0.00164

rs17042852 CDX & CLM 0.00034 0.00005

rs17042852 CDX & PEL 0.11856 0.00129

rs17042852 CDX & MXL 0.17160 0.00141

rs17042852 CDX & CHS 0.83815 0.00085

rs17042852 KHV & ACB 0.00004 0.00002

rs17042852 KHV & ASW 0.00037 0.00005

rs17042852 KHV & ESN 0.00003 0.00001

rs17042852 KHV & GWD 0.00000 0.00000

rs17042852 KHV & LWK 0.00002 0.00001

rs17042852 KHV & MSL 0.00004 0.00001

rs17042852 KHV & YRI 0.01207 0.00037

rs17042852 KHV & PUR 0.14984 0.00149

rs17042852 KHV & CLM 0.00001 0.00001

rs17042852 KHV & PEL 0.01986 0.00046

rs17042852 KHV & MXL 0.03018 0.00062

rs17042852 KHV & CHS 0.70553 0.00147

rs17042852 KHV & CDX 0.43218 0.00192

rs17042852 CHB & ACB 0.00366 0.00016

rs17042852 CHB & ASW 0.01653 0.00037

rs17042852 CHB & ESN 0.00372 0.00014

rs17042852 CHB & GWD 0.00122 0.00009

rs17042852 CHB & LWK 0.00392 0.00018

rs17042852 CHB & MSL 0.00485 0.00018

rs17042852 CHB & YRI 0.28388 0.00152

rs17042852 CHB & PUR 1.00000 0.00000

rs17042852 CHB & CLM 0.00390 0.00016

rs17042852 CHB & PEL 0.39792 0.00173

rs17042852 CHB & MXL 0.38541 0.00148

rs17042852 CHB & CHS 0.39136 0.00207

rs17042852 CHB & CDX 0.50181 0.00182

rs17042852 CHB & KHV 0.14768 0.00161

rs17042852 JPT & ACB 0.00000 0.00000

rs17042852 JPT & ASW 0.00001 0.00001

rs17042852 JPT & ESN 0.00000 0.00000

rs17042852 JPT & GWD 0.00000 0.00000

rs17042852 JPT & LWK 0.00000 0.00000

rs17042852 JPT & MSL 0.00000 0.00000

rs17042852 JPT & YRI 0.00001 0.00001

rs17042852 JPT & PUR 0.00248 0.00021

rs17042852 JPT & CLM 0.00000 0.00000

rs17042852 JPT & PEL 0.00008 0.00002

rs17042852 JPT & MXL 0.00049 0.00007

rs17042852 JPT & CHS 0.03258 0.00099

rs17042852 JPT & CDX 0.02596 0.00101

rs17042852 JPT & KHV 0.14345 0.00217

rs17042852 JPT & CHB 0.00256 0.00023

rs17042852 CEU & ACB 0.03061 0.00040

rs17042852 CEU & ASW 0.05676 0.00064

rs17042852 CEU & ESN 0.03045 0.00039

rs17042852 CEU & GWD 0.01004 0.00024

rs17042852 CEU & LWK 0.02980 0.00047

rs17042852 CEU & MSL 0.03242 0.00045

rs17042852 CEU & YRI 0.76349 0.00083

rs17042852 CEU & PUR 0.60110 0.00120

rs17042852 CEU & CLM 0.03094 0.00047

rs17042852 CEU & PEL 0.75843 0.00077

rs17042852 CEU & MXL 0.74992 0.00081

rs17042852 CEU & CHS 0.10820 0.00137

rs17042852 CEU & CDX 0.21665 0.00165

rs17042852 CEU & KHV 0.04536 0.00083

rs17042852 CEU & CHB 0.60366 0.00128

rs17042852 CEU & JPT 0.00018 0.00004

rs17042852 FIN & ACB 0.06068 0.00057

rs17042852 FIN & ASW 0.09743 0.00076

rs17042852 FIN & ESN 0.06066 0.00056

rs17042852 FIN & GWD 0.02099 0.00036

rs17042852 FIN & LWK 0.06058 0.00061

rs17042852 FIN & MSL 0.06468 0.00057

rs17042852 FIN & YRI 1.00000 0.00000

rs17042852 FIN & PUR 0.41509 0.00165

rs17042852 FIN & CLM 0.06110 0.00057

rs17042852 FIN & PEL 1.00000 0.00000

rs17042852 FIN & MXL 1.00000 0.00000

rs17042852 FIN & CHS 0.05926 0.00094

rs17042852 FIN & CDX 0.12572 0.00154

rs17042852 FIN & KHV 0.02195 0.00057

rs17042852 FIN & CHB 0.41500 0.00164

rs17042852 FIN & JPT 0.00009 0.00004

rs17042852 FIN & CEU 1.00000 0.00000

rs17042852 GBR & ACB 0.48714 0.00067

rs17042852 GBR & ASW 1.00000 0.00000

rs17042852 GBR & ESN 0.47984 0.00071

rs17042852 GBR & GWD 0.44740 0.00086

rs17042852 GBR & LWK 0.47869 0.00072

rs17042852 GBR & MSL 1.00000 0.00000

rs17042852 GBR & YRI 0.22618 0.00113

rs17042852 GBR & PUR 0.02301 0.00043

rs17042852 GBR & CLM 0.49218 0.00077

rs17042852 GBR & PEL 0.20081 0.00099

rs17042852 GBR & MXL 0.30932 0.00111

rs17042852 GBR & CHS 0.00101 0.00009

rs17042852 GBR & CDX 0.00590 0.00023

rs17042852 GBR & KHV 0.00024 0.00004

rs17042852 GBR & CHB 0.02256 0.00045

rs17042852 GBR & JPT 0.00000 0.00000

rs17042852 GBR & CEU 0.12342 0.00085

rs17042852 GBR & FIN 0.21669 0.00098

rs17042852 IBS & ACB 0.00012 0.00003

rs17042852 IBS & ASW 0.00158 0.00012

rs17042852 IBS & ESN 0.00014 0.00003

rs17042852 IBS & GWD 0.00002 0.00001

rs17042852 IBS & LWK 0.00011 0.00003

rs17042852 IBS & MSL 0.00023 0.00004

rs17042852 IBS & YRI 0.03630 0.00079

rs17042852 IBS & PUR 0.39336 0.00204

rs17042852 IBS & CLM 0.00017 0.00004

rs17042852 IBS & PEL 0.05555 0.00097

rs17042852 IBS & MXL 0.12096 0.00122

rs17042852 IBS & CHS 1.00000 0.00000

rs17042852 IBS & CDX 0.83910 0.00083

rs17042852 IBS & KHV 0.57304 0.00177

rs17042852 IBS & CHB 0.39060 0.00216

rs17042852 IBS & JPT 0.03254 0.00102

rs17042852 IBS & CEU 0.11321 0.00150

rs17042852 IBS & FIN 0.06167 0.00092

rs17042852 IBS & GBR 0.00125 0.00010

rs17042852 TSI & ACB 0.00000 0.00000

rs17042852 TSI & ASW 0.00006 0.00002

rs17042852 TSI & ESN 0.00001 0.00001

rs17042852 TSI & GWD 0.00000 0.00000

rs17042852 TSI & LWK 0.00000 0.00000

rs17042852 TSI & MSL 0.00003 0.00001

rs17042852 TSI & YRI 0.00152 0.00014

rs17042852 TSI & PUR 0.05254 0.00110

rs17042852 TSI & CLM 0.00000 0.00000

rs17042852 TSI & PEL 0.00513 0.00028

rs17042852 TSI & MXL 0.01285 0.00046

rs17042852 TSI & CHS 0.37086 0.00223

rs17042852 TSI & CDX 0.25861 0.00193

rs17042852 TSI & KHV 0.73011 0.00147

rs17042852 TSI & CHB 0.05438 0.00104

rs17042852 TSI & JPT 0.27911 0.00249

rs17042852 TSI & CEU 0.00840 0.00037

rs17042852 TSI & FIN 0.00364 0.00023

rs17042852 TSI & GBR 0.00005 0.00002

rs17042852 TSI & IBS 0.37151 0.00223

rs17042852 BEB & ACB 0.00507 0.00019

rs17042852 BEB & ASW 0.02500 0.00036

rs17042852 BEB & ESN 0.00439 0.00018

rs17042852 BEB & GWD 0.00262 0.00014

rs17042852 BEB & LWK 0.00445 0.00019

rs17042852 BEB & MSL 0.01437 0.00028

rs17042852 BEB & YRI 0.38450 0.00151

rs17042852 BEB & PUR 1.00000 0.00000

rs17042852 BEB & CLM 0.00532 0.00019

rs17042852 BEB & PEL 0.54053 0.00134

rs17042852 BEB & MXL 0.52478 0.00135

rs17042852 BEB & CHS 0.36607 0.00182

rs17042852 BEB & CDX 0.47515 0.00156

rs17042852 BEB & KHV 0.13569 0.00158

rs17042852 BEB & CHB 1.00000 0.00000

rs17042852 BEB & JPT 0.00213 0.00021

rs17042852 BEB & CEU 0.77818 0.00083

rs17042852 BEB & FIN 0.55816 0.00138

rs17042852 BEB & GBR 0.03195 0.00050

rs17042852 BEB & IBS 0.36724 0.00181

rs17042852 BEB & TSI 0.04658 0.00098

rs17042852 GIH & ACB 0.00762 0.00022

rs17042852 GIH & ASW 0.02805 0.00044

rs17042852 GIH & ESN 0.00792 0.00025

rs17042852 GIH & GWD 0.00246 0.00014

rs17042852 GIH & LWK 0.00721 0.00019

rs17042852 GIH & MSL 0.00913 0.00025

rs17042852 GIH & YRI 0.40746 0.00164

rs17042852 GIH & PUR 1.00000 0.00000

rs17042852 GIH & CLM 0.00766 0.00026

rs17042852 GIH & PEL 0.55702 0.00140

rs17042852 GIH & MXL 0.54082 0.00136

rs17042852 GIH & CHS 0.27347 0.00176

rs17042852 GIH & CDX 0.47924 0.00150

rs17042852 GIH & KHV 0.09380 0.00121

rs17042852 GIH & CHB 1.00000 0.00000

rs17042852 GIH & JPT 0.00114 0.00013

rs17042852 GIH & CEU 0.78873 0.00080

rs17042852 GIH & FIN 0.57566 0.00142

rs17042852 GIH & GBR 0.03944 0.00060

rs17042852 GIH & IBS 0.27366 0.00186

rs17042852 GIH & TSI 0.03032 0.00074

rs17042852 GIH & BEB 1.00000 0.00000

rs17042852 ITU & ACB 0.00756 0.00023

rs17042852 ITU & ASW 0.02744 0.00041

rs17042852 ITU & ESN 0.00725 0.00025

rs17042852 ITU & GWD 0.00242 0.00013

rs17042852 ITU & LWK 0.00730 0.00021

rs17042852 ITU & MSL 0.00881 0.00025

rs17042852 ITU & YRI 0.40518 0.00169

rs17042852 ITU & PUR 1.00000 0.00000

rs17042852 ITU & CLM 0.00754 0.00022

rs17042852 ITU & PEL 0.55763 0.00150

rs17042852 ITU & MXL 0.53894 0.00144

rs17042852 ITU & CHS 0.27394 0.00169

rs17042852 ITU & CDX 0.47996 0.00165

rs17042852 ITU & KHV 0.09292 0.00130

rs17042852 ITU & CHB 1.00000 0.00000

rs17042852 ITU & JPT 0.00136 0.00015

rs17042852 ITU & CEU 0.78786 0.00085

rs17042852 ITU & FIN 0.57753 0.00139

rs17042852 ITU & GBR 0.03904 0.00060

rs17042852 ITU & IBS 0.27395 0.00187

rs17042852 ITU & TSI 0.03109 0.00084

rs17042852 ITU & BEB 1.00000 0.00000

rs17042852 ITU & GIH 1.00000 0.00000

rs17042852 PJL & ACB 0.00707 0.00022

rs17042852 PJL & ASW 0.02536 0.00043

rs17042852 PJL & ESN 0.00308 0.00013

rs17042852 PJL & GWD 0.00182 0.00011

rs17042852 PJL & LWK 0.00323 0.00015

rs17042852 PJL & MSL 0.00807 0.00023

rs17042852 PJL & YRI 0.39976 0.00160

rs17042852 PJL & PUR 1.00000 0.00000

rs17042852 PJL & CLM 0.00776 0.00024

rs17042852 PJL & PEL 0.39045 0.00147

rs17042852 PJL & MXL 0.53660 0.00126

rs17042852 PJL & CHS 0.28654 0.00202

rs17042852 PJL & CDX 0.48700 0.00180

rs17042852 PJL & KHV 0.14445 0.00144

rs17042852 PJL & CHB 1.00000 0.00000

rs17042852 PJL & JPT 0.00218 0.00021

rs17042852 PJL & CEU 0.59650 0.00141

rs17042852 PJL & FIN 0.40542 0.00161

rs17042852 PJL & GBR 0.03690 0.00058

rs17042852 PJL & IBS 0.37969 0.00201

rs17042852 PJL & TSI 0.05098 0.00090

rs17042852 PJL & BEB 1.00000 0.00000

rs17042852 PJL & GIH 1.00000 0.00000

rs17042852 PJL & ITU 1.00000 0.00000

rs17042852 STU & ACB 0.00368 0.00015

rs17042852 STU & ASW 0.01604 0.00038

rs17042852 STU & ESN 0.00374 0.00015

rs17042852 STU & GWD 0.00100 0.00008

rs17042852 STU & LWK 0.00353 0.00016

rs17042852 STU & MSL 0.00516 0.00019

rs17042852 STU & YRI 0.28143 0.00158

rs17042852 STU & PUR 1.00000 0.00000

rs17042852 STU & CLM 0.00369 0.00016

rs17042852 STU & PEL 0.39782 0.00145

rs17042852 STU & MXL 0.38218 0.00151

rs17042852 STU & CHS 0.39363 0.00195

rs17042852 STU & CDX 0.64477 0.00130

rs17042852 STU & KHV 0.15029 0.00169

rs17042852 STU & CHB 1.00000 0.00000

rs17042852 STU & JPT 0.00312 0.00027

rs17042852 STU & CEU 0.59883 0.00142

rs17042852 STU & FIN 0.41616 0.00171

rs17042852 STU & GBR 0.02237 0.00041

rs17042852 STU & IBS 0.39178 0.00193

rs17042852 STU & TSI 0.05549 0.00116

rs17042852 STU & BEB 1.00000 0.00000

rs17042852 STU & GIH 0.80986 0.00072

rs17042852 STU & ITU 1.00000 0.00000

rs17042852 STU & PJL 1.00000 0.00000

Locus: rs11099660

=================================

Pop Alleles:

-----------------------------------------------------------

2 4 Total

ACB 104 88 192

ASW 60 62 122

ESN 99 99 198

GWD 106 120 226

LWK 126 72 198

MSL 91 79 170

YRI 119 97 216

PUR 142 66 208

CLM 141 47 188

PEL 126 44 170

MXL 90 38 128

CHS 164 46 210

CDX 157 29 186

KHV 150 48 198

CHB 160 46 206

JPT 163 45 208

CEU 147 51 198

FIN 154 44 198

GBR 122 60 182

IBS 166 48 214

TSI 161 53 214

BEB 134 38 172

GIH 161 45 206

ITU 157 47 204

PJL 147 45 192

STU 168 36 204

Total: 35151493 5008

Locus Population pair P-Value S.E.

----------- --------------------- ------- -------

rs11099660 ASW & ACB 0.41630 0.00361

rs11099660 ESN & ACB 0.41484 0.00374

rs11099660 ESN & ASW 0.90792 0.00089

rs11099660 GWD & ACB 0.14845 0.00359

rs11099660 GWD & ASW 0.73643 0.00250

rs11099660 GWD & ESN 0.56207 0.00343

rs11099660 LWK & ACB 0.06624 0.00215

rs11099660 LWK & ASW 0.01503 0.00089

rs11099660 LWK & ESN 0.00799 0.00073

rs11099660 LWK & GWD 0.00064 0.00018

rs11099660 MSL & ACB 0.91615 0.00090

rs11099660 MSL & ASW 0.47267 0.00331

rs11099660 MSL & ESN 0.53179 0.00297

rs11099660 MSL & GWD 0.22645 0.00371

rs11099660 MSL & LWK 0.05902 0.00188

rs11099660 YRI & ACB 0.92040 0.00092

rs11099660 YRI & ASW 0.31747 0.00341

rs11099660 YRI & ESN 0.32746 0.00474

rs11099660 YRI & GWD 0.08808 0.00284

rs11099660 YRI & LWK 0.08609 0.00243

rs11099660 YRI & MSL 0.83687 0.00167

rs11099660 PUR & ACB 0.00411 0.00048

rs11099660 PUR & ASW 0.00088 0.00022

rs11099660 PUR & ESN 0.00011 0.00004

rs11099660 PUR & GWD 0.00000 0.00000

rs11099660 PUR & LWK 0.34621 0.00372

rs11099660 PUR & MSL 0.00453 0.00049

rs11099660 PUR & YRI 0.00713 0.00061

rs11099660 CLM & ACB 0.00001 0.00001

rs11099660 CLM & ASW 0.00000 0.00000

rs11099660 CLM & ESN 0.00000 0.00000

rs11099660 CLM & GWD 0.00000 0.00000

rs11099660 CLM & LWK 0.01990 0.00092

rs11099660 CLM & MSL 0.00000 0.00000

rs11099660 CLM & YRI 0.00000 0.00000

rs11099660 CLM & PUR 0.14609 0.00311

rs11099660 PEL & ACB 0.00005 0.00002

rs11099660 PEL & ASW 0.00005 0.00003

rs11099660 PEL & ESN 0.00003 0.00003

rs11099660 PEL & GWD 0.00000 0.00000

rs11099660 PEL & LWK 0.03427 0.00141

rs11099660 PEL & MSL 0.00023 0.00010

rs11099660 PEL & YRI 0.00021 0.00008

rs11099660 PEL & PUR 0.25141 0.00381

rs11099660 PEL & CLM 0.90447 0.00101

rs11099660 MXL & ACB 0.00446 0.00042

rs11099660 MXL & ASW 0.00077 0.00015

rs11099660 MXL & ESN 0.00044 0.00010

rs11099660 MXL & GWD 0.00001 0.00001

rs11099660 MXL & LWK 0.23247 0.00320

rs11099660 MXL & MSL 0.00454 0.00042

rs11099660 MXL & YRI 0.00615 0.00050

rs11099660 MXL & PUR 0.71713 0.00244

rs11099660 MXL & CLM 0.37244 0.00352

rs11099660 MXL & PEL 0.51435 0.00297

rs11099660 CHS & ACB 0.00000 0.00000

rs11099660 CHS & ASW 0.00000 0.00000

rs11099660 CHS & ESN 0.00000 0.00000

rs11099660 CHS & GWD 0.00000 0.00000

rs11099660 CHS & LWK 0.00178 0.00026

rs11099660 CHS & MSL 0.00000 0.00000

rs11099660 CHS & YRI 0.00000 0.00000

rs11099660 CHS & PUR 0.02708 0.00115

rs11099660 CHS & CLM 0.47266 0.00285

rs11099660 CHS & PEL 0.39251 0.00329

rs11099660 CHS & MXL 0.12045 0.00238

rs11099660 CDX & ACB 0.00000 0.00000

rs11099660 CDX & ASW 0.00000 0.00000

rs11099660 CDX & ESN 0.00000 0.00000

rs11099660 CDX & GWD 0.00000 0.00000

rs11099660 CDX & LWK 0.00001 0.00001

rs11099660 CDX & MSL 0.00000 0.00000

rs11099660 CDX & YRI 0.00000 0.00000

rs11099660 CDX & PUR 0.00035 0.00009

rs11099660 CDX & CLM 0.02852 0.00107

rs11099660 CDX & PEL 0.01891 0.00098

rs11099660 CDX & MXL 0.00314 0.00028

rs11099660 CDX & CHS 0.12338 0.00241

rs11099660 KHV & ACB 0.00002 0.00002

rs11099660 KHV & ASW 0.00000 0.00000

rs11099660 KHV & ESN 0.00000 0.00000

rs11099660 KHV & GWD 0.00000 0.00000

rs11099660 KHV & LWK 0.01199 0.00072

rs11099660 KHV & MSL 0.00000 0.00000

rs11099660 KHV & YRI 0.00001 0.00001

rs11099660 KHV & PUR 0.09738 0.00218

rs11099660 KHV & CLM 0.90703 0.00080

rs11099660 KHV & PEL 0.80744 0.00168

rs11099660 KHV & MXL 0.29977 0.00290

rs11099660 KHV & CHS 0.63411 0.00283

rs11099660 KHV & CDX 0.03952 0.00133

rs11099660 CHB & ACB 0.00000 0.00000

rs11099660 CHB & ASW 0.00000 0.00000

rs11099660 CHB & ESN 0.00000 0.00000

rs11099660 CHB & GWD 0.00000 0.00000

rs11099660 CHB & LWK 0.00227 0.00030

rs11099660 CHB & MSL 0.00000 0.00000

rs11099660 CHB & YRI 0.00000 0.00000

rs11099660 CHB & PUR 0.03453 0.00142

rs11099660 CHB & CLM 0.55063 0.00275

rs11099660 CHB & PEL 0.46747 0.00336

rs11099660 CHB & MXL 0.15027 0.00241

rs11099660 CHB & CHS 1.00000 0.00000

rs11099660 CHB & CDX 0.09840 0.00208

rs11099660 CHB & KHV 0.72754 0.00240

rs11099660 JPT & ACB 0.00000 0.00000

rs11099660 JPT & ASW 0.00000 0.00000

rs11099660 JPT & ESN 0.00000 0.00000

rs11099660 JPT & GWD 0.00000 0.00000

rs11099660 JPT & LWK 0.00143 0.00023

rs11099660 JPT & MSL 0.00000 0.00000

rs11099660 JPT & YRI 0.00000 0.00000

rs11099660 JPT & PUR 0.02829 0.00142

rs11099660 JPT & CLM 0.47371 0.00315

rs11099660 JPT & PEL 0.39892 0.00325

rs11099660 JPT & MXL 0.11831 0.00219

rs11099660 JPT & CHS 1.00000 0.00000

rs11099660 JPT & CDX 0.15364 0.00270

rs11099660 JPT & KHV 0.55311 0.00298

rs11099660 JPT & CHB 0.90485 0.00090

rs11099660 CEU & ACB 0.00001 0.00001

rs11099660 CEU & ASW 0.00001 0.00001

rs11099660 CEU & ESN 0.00000 0.00000

rs11099660 CEU & GWD 0.00000 0.00000

rs11099660 CEU & LWK 0.03047 0.00144

rs11099660 CEU & MSL 0.00004 0.00002

rs11099660 CEU & YRI 0.00010 0.00008

rs11099660 CEU & PUR 0.19594 0.00355

rs11099660 CEU & CLM 0.90643 0.00090

rs11099660 CEU & PEL 1.00000 0.00000

rs11099660 CEU & MXL 0.44738 0.00297

rs11099660 CEU & CHS 0.41681 0.00315

rs11099660 CEU & CDX 0.01757 0.00086

rs11099660 CEU & KHV 0.81648 0.00162

rs11099660 CEU & CHB 0.48384 0.00334

rs11099660 CEU & JPT 0.35551 0.00400

rs11099660 FIN & ACB 0.00000 0.00000

rs11099660 FIN & ASW 0.00000 0.00000

rs11099660 FIN & ESN 0.00000 0.00000

rs11099660 FIN & GWD 0.00000 0.00000

rs11099660 FIN & LWK 0.00259 0.00033

rs11099660 FIN & MSL 0.00000 0.00000

rs11099660 FIN & YRI 0.00000 0.00000

rs11099660 FIN & PUR 0.03537 0.00147

rs11099660 FIN & CLM 0.54877 0.00347

rs11099660 FIN & PEL 0.46310 0.00318

rs11099660 FIN & MXL 0.15027 0.00247

rs11099660 FIN & CHS 1.00000 0.00000

rs11099660 FIN & CDX 0.11492 0.00207

rs11099660 FIN & KHV 0.72358 0.00237

rs11099660 FIN & CHB 1.00000 0.00000

rs11099660 FIN & JPT 0.90601 0.00090

rs11099660 FIN & CEU 0.48181 0.00317

rs11099660 GBR & ACB 0.01257 0.00089

rs11099660 GBR & ASW 0.00269 0.00030

rs11099660 GBR & ESN 0.00097 0.00020

rs11099660 GBR & GWD 0.00009 0.00007

rs11099660 GBR & LWK 0.51875 0.00363

rs11099660 GBR & MSL 0.01315 0.00091

rs11099660 GBR & YRI 0.01841 0.00106

rs11099660 GBR & PUR 0.82788 0.00165

rs11099660 GBR & CLM 0.10800 0.00247

rs11099660 GBR & PEL 0.16051 0.00249

rs11099660 GBR & MXL 0.61531 0.00284

rs11099660 GBR & CHS 0.01493 0.00083

rs11099660 GBR & CDX 0.00016 0.00005

rs11099660 GBR & KHV 0.07237 0.00185

rs11099660 GBR & CHB 0.02356 0.00112

rs11099660 GBR & JPT 0.01673 0.00090

rs11099660 GBR & CEU 0.14182 0.00339

rs11099660 GBR & FIN 0.02088 0.00108

rs11099660 IBS & ACB 0.00000 0.00000

rs11099660 IBS & ASW 0.00000 0.00000

rs11099660 IBS & ESN 0.00000 0.00000

rs11099660 IBS & GWD 0.00000 0.00000

rs11099660 IBS & LWK 0.00241 0.00030

rs11099660 IBS & MSL 0.00000 0.00000

rs11099660 IBS & YRI 0.00000 0.00000

rs11099660 IBS & PUR 0.03693 0.00168

rs11099660 IBS & CLM 0.55852 0.00287

rs11099660 IBS & PEL 0.46912 0.00323

rs11099660 IBS & MXL 0.16291 0.00268

rs11099660 IBS & CHS 0.90745 0.00085

rs11099660 IBS & CDX 0.10078 0.00213

rs11099660 IBS & KHV 0.72841 0.00201

rs11099660 IBS & CHB 1.00000 0.00000

rs11099660 IBS & JPT 0.90932 0.00094

rs11099660 IBS & CEU 0.48531 0.00320

rs11099660 IBS & FIN 1.00000 0.00000

rs11099660 IBS & GBR 0.02347 0.00120

rs11099660 TSI & ACB 0.00000 0.00000

rs11099660 TSI & ASW 0.00001 0.00001

rs11099660 TSI & ESN 0.00000 0.00000

rs11099660 TSI & GWD 0.00000 0.00000

rs11099660 TSI & LWK 0.01231 0.00071

rs11099660 TSI & MSL 0.00000 0.00000

rs11099660 TSI & YRI 0.00000 0.00000

rs11099660 TSI & PUR 0.13015 0.00270

rs11099660 TSI & CLM 1.00000 0.00000

rs11099660 TSI & PEL 0.81819 0.00157

rs11099660 TSI & MXL 0.37893 0.00310

rs11099660 TSI & CHS 0.48949 0.00338

rs11099660 TSI & CDX 0.02695 0.00119

rs11099660 TSI & KHV 0.90962 0.00086

rs11099660 TSI & CHB 0.56156 0.00307

rs11099660 TSI & JPT 0.49575 0.00317

rs11099660 TSI & CEU 0.81998 0.00164

rs11099660 TSI & FIN 0.56630 0.00313

rs11099660 TSI & GBR 0.07320 0.00213

rs11099660 TSI & IBS 0.64718 0.00260

rs11099660 BEB & ACB 0.00000 0.00000

rs11099660 BEB & ASW 0.00000 0.00000

rs11099660 BEB & ESN 0.00000 0.00000

rs11099660 BEB & GWD 0.00000 0.00000

rs11099660 BEB & LWK 0.00332 0.00037

rs11099660 BEB & MSL 0.00000 0.00000

rs11099660 BEB & YRI 0.00000 0.00000

rs11099660 BEB & PUR 0.03894 0.00162

rs11099660 BEB & CLM 0.53681 0.00298

rs11099660 BEB & PEL 0.44704 0.00278

rs11099660 BEB & MXL 0.14274 0.00275

rs11099660 BEB & CHS 1.00000 0.00000

rs11099660 BEB & CDX 0.13381 0.00233

rs11099660 BEB & KHV 0.71043 0.00213

rs11099660 BEB & CHB 1.00000 0.00000

rs11099660 BEB & JPT 1.00000 0.00000

rs11099660 BEB & CEU 0.46220 0.00313

rs11099660 BEB & FIN 1.00000 0.00000

rs11099660 BEB & GBR 0.02449 0.00115

rs11099660 BEB & IBS 1.00000 0.00000

rs11099660 BEB & TSI 0.55168 0.00309

rs11099660 GIH & ACB 0.00000 0.00000

rs11099660 GIH & ASW 0.00000 0.00000

rs11099660 GIH & ESN 0.00000 0.00000

rs11099660 GIH & GWD 0.00000 0.00000

rs11099660 GIH & LWK 0.00217 0.00031

rs11099660 GIH & MSL 0.00000 0.00000

rs11099660 GIH & YRI 0.00000 0.00000

rs11099660 GIH & PUR 0.02526 0.00115

rs11099660 GIH & CLM 0.47234 0.00345

rs11099660 GIH & PEL 0.39297 0.00309

rs11099660 GIH & MXL 0.11547 0.00204

rs11099660 GIH & CHS 1.00000 0.00000

rs11099660 GIH & CDX 0.12508 0.00268

rs11099660 GIH & KHV 0.63405 0.00239

rs11099660 GIH & CHB 1.00000 0.00000

rs11099660 GIH & JPT 1.00000 0.00000

rs11099660 GIH & CEU 0.41214 0.00312

rs11099660 GIH & FIN 1.00000 0.00000

rs11099660 GIH & GBR 0.01670 0.00093

rs11099660 GIH & IBS 0.90653 0.00087

rs11099660 GIH & TSI 0.49115 0.00365

rs11099660 GIH & BEB 1.00000 0.00000

rs11099660 ITU & ACB 0.00000 0.00000

rs11099660 ITU & ASW 0.00000 0.00000

rs11099660 ITU & ESN 0.00000 0.00000

rs11099660 ITU & GWD 0.00000 0.00000

rs11099660 ITU & LWK 0.00419 0.00038

rs11099660 ITU & MSL 0.00000 0.00000

rs11099660 ITU & YRI 0.00000 0.00000

rs11099660 ITU & PUR 0.05780 0.00182

rs11099660 ITU & CLM 0.72084 0.00238

rs11099660 ITU & PEL 0.54698 0.00346

rs11099660 ITU & MXL 0.20046 0.00281

rs11099660 ITU & CHS 0.81491 0.00149

rs11099660 ITU & CDX 0.07240 0.00187

rs11099660 ITU & KHV 0.81264 0.00164

rs11099660 ITU & CHB 0.90550 0.00084

rs11099660 ITU & JPT 0.81630 0.00185

rs11099660 ITU & CEU 0.56122 0.00289

rs11099660 ITU & FIN 0.90535 0.00091

rs11099660 ITU & GBR 0.03000 0.00131

rs11099660 ITU & IBS 0.90563 0.00095

rs11099660 ITU & TSI 0.73043 0.00224

rs11099660 ITU & BEB 0.90320 0.00094

rs11099660 ITU & GIH 0.81556 0.00164

rs11099660 PJL & ACB 0.00000 0.00000

rs11099660 PJL & ASW 0.00000 0.00000

rs11099660 PJL & ESN 0.00000 0.00000

rs11099660 PJL & GWD 0.00000 0.00000

rs11099660 PJL & LWK 0.00540 0.00049

rs11099660 PJL & MSL 0.00000 0.00000

rs11099660 PJL & YRI 0.00000 0.00000

rs11099660 PJL & PUR 0.06940 0.00193

rs11099660 PJL & CLM 0.81072 0.00146

rs11099660 PJL & PEL 0.63034 0.00278

rs11099660 PJL & MXL 0.24070 0.00277

rs11099660 PJL & CHS 0.72467 0.00235

rs11099660 PJL & CDX 0.06772 0.00193

rs11099660 PJL & KHV 0.90496 0.00088

rs11099660 PJL & CHB 0.81002 0.00142

rs11099660 PJL & JPT 0.72292 0.00236

rs11099660 PJL & CEU 0.63780 0.00271

rs11099660 PJL & FIN 0.80915 0.00146

rs11099660 PJL & GBR 0.05167 0.00170

rs11099660 PJL & IBS 0.81555 0.00190

rs11099660 PJL & TSI 0.81692 0.00156

rs11099660 PJL & BEB 0.80397 0.00163

rs11099660 PJL & GIH 0.71855 0.00246

rs11099660 PJL & ITU 1.00000 0.00000

rs11099660 STU & ACB 0.00000 0.00000

rs11099660 STU & ASW 0.00000 0.00000

rs11099660 STU & ESN 0.00000 0.00000

rs11099660 STU & GWD 0.00000 0.00000

rs11099660 STU & LWK 0.00003 0.00002

rs11099660 STU & MSL 0.00000 0.00000

rs11099660 STU & YRI 0.00000 0.00000

rs11099660 STU & PUR 0.00115 0.00017

rs11099660 STU & CLM 0.08555 0.00210

rs11099660 STU & PEL 0.05986 0.00169

rs11099660 STU & MXL 0.01494 0.00078

rs11099660 STU & CHS 0.32791 0.00346

rs11099660 STU & CDX 0.58902 0.00226

rs11099660 STU & KHV 0.10883 0.00216

rs11099660 STU & CHB 0.27319 0.00315

rs11099660 STU & JPT 0.32204 0.00336

rs11099660 STU & CEU 0.04998 0.00147

rs11099660 STU & FIN 0.26305 0.00318

rs11099660 STU & GBR 0.00062 0.00013

rs11099660 STU & IBS 0.27222 0.00298

rs11099660 STU & TSI 0.09791 0.00226

rs11099660 STU & BEB 0.30091 0.00303

rs11099660 STU & GIH 0.32410 0.00323

rs11099660 STU & ITU 0.22355 0.00268

rs11099660 STU & PJL 0.17268 0.00285

Locus: rs4980079

=================================

Pop Alleles:

-----------------------------------------------------------

2 4 Total

ACB 153 39 192

ASW 102 20 122

ESN 156 42 198

GWD 211 15 226

LWK 179 19 198

MSL 159 11 170

YRI 180 36 216

PUR 125 83 208

CLM 120 68 188

PEL 131 39 170

MXL 86 42 128

CHS 133 77 210

CDX 124 62 186

KHV 133 65 198

CHB 149 57 206

JPT 125 83 208

CEU 104 94 198

FIN 126 72 198

GBR 107 75 182

IBS 124 90 214

TSI 125 89 214

BEB 124 48 172

GIH 152 54 206

ITU 157 47 204

PJL 143 49 192

STU 159 45 204

Total: 35871421 5008

Locus Population pair P-Value S.E.

----------- --------------------- ------- -------

rs4980079 ASW & ACB 0.45824 0.00256

rs4980079 ESN & ACB 0.90013 0.00081

rs4980079 ESN & ASW 0.31039 0.00307

rs4980079 GWD & ACB 0.00002 0.00001

rs4980079 GWD & ASW 0.00780 0.00038

rs4980079 GWD & ESN 0.00002 0.00002

rs4980079 LWK & ACB 0.00366 0.00028

rs4980079 LWK & ASW 0.07885 0.00145

rs4980079 LWK & ESN 0.00227 0.00023

rs4980079 LWK & GWD 0.28532 0.00250

rs4980079 MSL & ACB 0.00013 0.00008

rs4980079 MSL & ASW 0.01134 0.00048

rs4980079 MSL & ESN 0.00005 0.00003

rs4980079 MSL & GWD 1.00000 0.00000

rs4980079 MSL & LWK 0.34198 0.00243

rs4980079 YRI & ACB 0.36949 0.00322

rs4980079 YRI & ASW 1.00000 0.00000

rs4980079 YRI & ESN 0.25928 0.00354

rs4980079 YRI & GWD 0.00114 0.00016

rs4980079 YRI & LWK 0.04280 0.00112

rs4980079 YRI & MSL 0.00299 0.00033

rs4980079 PUR & ACB 0.00003 0.00002

rs4980079 PUR & ASW 0.00000 0.00000

rs4980079 PUR & ESN 0.00000 0.00000

rs4980079 PUR & GWD 0.00000 0.00000

rs4980079 PUR & LWK 0.00000 0.00000

rs4980079 PUR & MSL 0.00000 0.00000

rs4980079 PUR & YRI 0.00000 0.00000

rs4980079 CLM & ACB 0.00049 0.00011

rs4980079 CLM & ASW 0.00019 0.00005

rs4980079 CLM & ESN 0.00148 0.00022

rs4980079 CLM & GWD 0.00000 0.00000

rs4980079 CLM & LWK 0.00000 0.00000

rs4980079 CLM & MSL 0.00000 0.00000

rs4980079 CLM & YRI 0.00000 0.00000

rs4980079 CLM & PUR 0.46734 0.00352

rs4980079 PEL & ACB 0.61279 0.00284

rs4980079 PEL & ASW 0.18424 0.00234

rs4980079 PEL & ESN 0.70556 0.00221

rs4980079 PEL & GWD 0.00002 0.00002

rs4980079 PEL & LWK 0.00043 0.00010

rs4980079 PEL & MSL 0.00001 0.00001

rs4980079 PEL & YRI 0.15383 0.00252

rs4980079 PEL & PUR 0.00063 0.00013

rs4980079 PEL & CLM 0.00826 0.00057

rs4980079 MXL & ACB 0.01807 0.00081

rs4980079 MXL & ASW 0.00333 0.00031

rs4980079 MXL & ESN 0.02618 0.00113

rs4980079 MXL & GWD 0.00000 0.00000

rs4980079 MXL & LWK 0.00000 0.00000

rs4980079 MXL & MSL 0.00000 0.00000

rs4980079 MXL & YRI 0.00089 0.00015

rs4980079 MXL & PUR 0.20512 0.00319

rs4980079 MXL & CLM 0.54703 0.00322

rs4980079 MXL & PEL 0.06941 0.00189

rs4980079 CHS & ACB 0.00055 0.00016

rs4980079 CHS & ASW 0.00007 0.00004

rs4980079 CHS & ESN 0.00088 0.00018

rs4980079 CHS & GWD 0.00000 0.00000

rs4980079 CHS & LWK 0.00000 0.00000

rs4980079 CHS & MSL 0.00000 0.00000

rs4980079 CHS & YRI 0.00000 0.00000

rs4980079 CHS & PUR 0.54868 0.00347

rs4980079 CHS & CLM 1.00000 0.00000

rs4980079 CHS & PEL 0.00445 0.00039

rs4980079 CHS & MXL 0.48512 0.00270

rs4980079 CDX & ACB 0.00506 0.00040

rs4980079 CDX & ASW 0.00096 0.00015

rs4980079 CDX & ESN 0.00835 0.00055

rs4980079 CDX & GWD 0.00000 0.00000

rs4980079 CDX & LWK 0.00000 0.00000

rs4980079 CDX & MSL 0.00000 0.00000

rs4980079 CDX & YRI 0.00010 0.00004

rs4980079 CDX & PUR 0.20450 0.00400

rs4980079 CDX & CLM 0.59175 0.00301

rs4980079 CDX & PEL 0.03464 0.00123

rs4980079 CDX & MXL 1.00000 0.00000

rs4980079 CDX & CHS 0.52697 0.00388

rs4980079 KHV & ACB 0.00579 0.00053

rs4980079 KHV & ASW 0.00095 0.00017

rs4980079 KHV & ESN 0.01374 0.00077

rs4980079 KHV & GWD 0.00000 0.00000

rs4980079 KHV & LWK 0.00000 0.00000

rs4980079 KHV & MSL 0.00000 0.00000

rs4980079 KHV & YRI 0.00017 0.00005

rs4980079 KHV & PUR 0.15157 0.00300

rs4980079 KHV & CLM 0.51855 0.00319

rs4980079 KHV & PEL 0.03633 0.00145

rs4980079 KHV & MXL 1.00000 0.00000

rs4980079 KHV & CHS 0.46619 0.00430

rs4980079 KHV & CDX 1.00000 0.00000

rs4980079 CHB & ACB 0.09735 0.00261

rs4980079 CHB & ASW 0.02270 0.00101

rs4980079 CHB & ESN 0.13219 0.00279

rs4980079 CHB & GWD 0.00000 0.00000

rs4980079 CHB & LWK 0.00000 0.00000

rs4980079 CHB & MSL 0.00000 0.00000

rs4980079 CHB & YRI 0.00673 0.00048

rs4980079 CHB & PUR 0.00859 0.00060

rs4980079 CHB & CLM 0.08525 0.00241

rs4980079 CHB & PEL 0.34499 0.00348

rs4980079 CHB & MXL 0.32463 0.00333

rs4980079 CHB & CHS 0.06166 0.00187

rs4980079 CHB & CDX 0.23034 0.00329

rs4980079 CHB & KHV 0.27947 0.00368

rs4980079 JPT & ACB 0.00000 0.00000

rs4980079 JPT & ASW 0.00000 0.00000

rs4980079 JPT & ESN 0.00010 0.00005

rs4980079 JPT & GWD 0.00000 0.00000

rs4980079 JPT & LWK 0.00000 0.00000

rs4980079 JPT & MSL 0.00000 0.00000

rs4980079 JPT & YRI 0.00000 0.00000

rs4980079 JPT & PUR 1.00000 0.00000

rs4980079 JPT & CLM 0.46814 0.00334

rs4980079 JPT & PEL 0.00064 0.00017

rs4980079 JPT & MXL 0.20367 0.00289

rs4980079 JPT & CHS 0.54546 0.00308

rs4980079 JPT & CDX 0.20780 0.00408

rs4980079 JPT & KHV 0.15342 0.00335

rs4980079 JPT & CHB 0.00982 0.00083

rs4980079 CEU & ACB 0.00000 0.00000

rs4980079 CEU & ASW 0.00000 0.00000

rs4980079 CEU & ESN 0.00000 0.00000

rs4980079 CEU & GWD 0.00000 0.00000

rs4980079 CEU & LWK 0.00000 0.00000

rs4980079 CEU & MSL 0.00000 0.00000

rs4980079 CEU & YRI 0.00000 0.00000

rs4980079 CEU & PUR 0.12982 0.00269

rs4980079 CEU & CLM 0.03073 0.00140

rs4980079 CEU & PEL 0.00000 0.00000

rs4980079 CEU & MXL 0.01086 0.00070

rs4980079 CEU & CHS 0.02636 0.00124

rs4980079 CEU & CDX 0.00572 0.00050

rs4980079 CEU & KHV 0.00344 0.00040

rs4980079 CEU & CHB 0.00007 0.00004

rs4980079 CEU & JPT 0.13310 0.00292

rs4980079 FIN & ACB 0.00068 0.00014

rs4980079 FIN & ASW 0.00012 0.00005

rs4980079 FIN & ESN 0.00135 0.00025

rs4980079 FIN & GWD 0.00000 0.00000

rs4980079 FIN & LWK 0.00000 0.00000

rs4980079 FIN & MSL 0.00000 0.00000

rs4980079 FIN & YRI 0.00003 0.00002

rs4980079 FIN & PUR 0.48007 0.00368

rs4980079 FIN & CLM 1.00000 0.00000

rs4980079 FIN & PEL 0.00659 0.00053

rs4980079 FIN & MXL 0.55234 0.00295

rs4980079 FIN & CHS 1.00000 0.00000

rs4980079 FIN & CDX 0.59659 0.00323

rs4980079 FIN & KHV 0.53138 0.00387

rs4980079 FIN & CHB 0.06892 0.00223

rs4980079 FIN & JPT 0.48204 0.00388

rs4980079 FIN & CEU 0.02989 0.00133

rs4980079 GBR & ACB 0.00001 0.00001

rs4980079 GBR & ASW 0.00000 0.00000

rs4980079 GBR & ESN 0.00014 0.00010

rs4980079 GBR & GWD 0.00000 0.00000

rs4980079 GBR & LWK 0.00000 0.00000

rs4980079 GBR & MSL 0.00000 0.00000

rs4980079 GBR & YRI 0.00000 0.00000

rs4980079 GBR & PUR 0.83864 0.00170

rs4980079 GBR & CLM 0.33902 0.00332

rs4980079 GBR & PEL 0.00026 0.00009

rs4980079 GBR & MXL 0.15541 0.00277

rs4980079 GBR & CHS 0.40704 0.00389

rs4980079 GBR & CDX 0.13258 0.00302

rs4980079 GBR & KHV 0.10930 0.00283

rs4980079 GBR & CHB 0.00513 0.00052

rs4980079 GBR & JPT 0.83553 0.00138

rs4980079 GBR & CEU 0.26012 0.00389

rs4980079 GBR & FIN 0.33914 0.00370

rs4980079 IBS & ACB 0.00000 0.00000

rs4980079 IBS & ASW 0.00000 0.00000

rs4980079 IBS & ESN 0.00000 0.00000

rs4980079 IBS & GWD 0.00000 0.00000

rs4980079 IBS & LWK 0.00000 0.00000

rs4980079 IBS & MSL 0.00000 0.00000

rs4980079 IBS & YRI 0.00000 0.00000

rs4980079 IBS & PUR 0.69138 0.00305

rs4980079 IBS & CLM 0.26559 0.00378

rs4980079 IBS & PEL 0.00007 0.00005

rs4980079 IBS & MXL 0.10633 0.00214

rs4980079 IBS & CHS 0.27861 0.00421

rs4980079 IBS & CDX 0.07791 0.00226

rs4980079 IBS & KHV 0.07229 0.00243

rs4980079 IBS & CHB 0.00208 0.00027

rs4980079 IBS & JPT 0.69268 0.00273

rs4980079 IBS & CEU 0.28134 0.00433

rs4980079 IBS & FIN 0.26140 0.00373

rs4980079 IBS & GBR 0.91924 0.00094

rs4980079 TSI & ACB 0.00000 0.00000

rs4980079 TSI & ASW 0.00000 0.00000

rs4980079 TSI & ESN 0.00000 0.00000

rs4980079 TSI & GWD 0.00000 0.00000

rs4980079 TSI & LWK 0.00000 0.00000

rs4980079 TSI & MSL 0.00000 0.00000

rs4980079 TSI & YRI 0.00000 0.00000

rs4980079 TSI & PUR 0.76969 0.00218

rs4980079 TSI & CLM 0.30688 0.00411

rs4980079 TSI & PEL 0.00011 0.00006

rs4980079 TSI & MXL 0.11253 0.00249

rs4980079 TSI & CHS 0.31495 0.00395

rs4980079 TSI & CDX 0.09363 0.00246

rs4980079 TSI & KHV 0.06615 0.00208

rs4980079 TSI & CHB 0.00270 0.00035

rs4980079 TSI & JPT 0.76815 0.00249

rs4980079 TSI & CEU 0.23344 0.00337

rs4980079 TSI & FIN 0.30914 0.00360

rs4980079 TSI & GBR 1.00000 0.00000

rs4980079 TSI & IBS 1.00000 0.00000

rs4980079 BEB & ACB 0.10882 0.00221

rs4980079 BEB & ASW 0.02351 0.00092

rs4980079 BEB & ESN 0.14743 0.00269

rs4980079 BEB & GWD 0.00000 0.00000

rs4980079 BEB & LWK 0.00000 0.00000

rs4980079 BEB & MSL 0.00000 0.00000

rs4980079 BEB & YRI 0.00906 0.00069

rs4980079 BEB & PUR 0.01815 0.00101

rs4980079 BEB & CLM 0.11082 0.00273

rs4980079 BEB & PEL 0.32267 0.00327

rs4980079 BEB & MXL 0.37889 0.00334

rs4980079 BEB & CHS 0.07794 0.00219

rs4980079 BEB & CDX 0.30008 0.00351

rs4980079 BEB & KHV 0.31402 0.00383

rs4980079 BEB & CHB 1.00000 0.00000

rs4980079 BEB & JPT 0.01697 0.00092

rs4980079 BEB & CEU 0.00012 0.00006

rs4980079 BEB & FIN 0.09650 0.00226

rs4980079 BEB & GBR 0.00870 0.00063

rs4980079 BEB & IBS 0.00400 0.00041

rs4980079 BEB & TSI 0.00635 0.00054

rs4980079 GIH & ACB 0.19161 0.00306

rs4980079 GIH & ASW 0.04117 0.00131

rs4980079 GIH & ESN 0.24743 0.00316

rs4980079 GIH & GWD 0.00000 0.00000

rs4980079 GIH & LWK 0.00002 0.00001

rs4980079 GIH & MSL 0.00000 0.00000

rs4980079 GIH & YRI 0.01723 0.00083

rs4980079 GIH & PUR 0.00382 0.00050

rs4980079 GIH & CLM 0.03726 0.00161

rs4980079 GIH & PEL 0.47691 0.00334

rs4980079 GIH & MXL 0.21558 0.00314

rs4980079 GIH & CHS 0.02676 0.00128

rs4980079 GIH & CDX 0.14405 0.00270

rs4980079 GIH & KHV 0.16075 0.00267

rs4980079 GIH & CHB 0.82391 0.00173

rs4980079 GIH & JPT 0.00382 0.00042

rs4980079 GIH & CEU 0.00000 0.00000

rs4980079 GIH & FIN 0.02923 0.00128

rs4980079 GIH & GBR 0.00226 0.00030

rs4980079 GIH & IBS 0.00053 0.00011

rs4980079 GIH & TSI 0.00087 0.00017

rs4980079 GIH & BEB 0.72990 0.00213

rs4980079 ITU & ACB 0.54460 0.00347

rs4980079 ITU & ASW 0.16076 0.00223

rs4980079 ITU & ESN 0.72129 0.00203

rs4980079 ITU & GWD 0.00000 0.00000

rs4980079 ITU & LWK 0.00023 0.00007

rs4980079 ITU & MSL 0.00003 0.00003

rs4980079 ITU & YRI 0.11347 0.00233

rs4980079 ITU & PUR 0.00013 0.00004

rs4980079 ITU & CLM 0.00564 0.00057

rs4980079 ITU & PEL 1.00000 0.00000

rs4980079 ITU & MXL 0.05540 0.00175

rs4980079 ITU & CHS 0.00199 0.00027

rs4980079 ITU & CDX 0.03198 0.00152

rs4980079 ITU & KHV 0.03402 0.00154

rs4980079 ITU & CHB 0.31008 0.00365

rs4980079 ITU & JPT 0.00027 0.00011

rs4980079 ITU & CEU 0.00000 0.00000

rs4980079 ITU & FIN 0.00455 0.00047

rs4980079 ITU & GBR 0.00007 0.00003

rs4980079 ITU & IBS 0.00004 0.00004

rs4980079 ITU & TSI 0.00002 0.00002

rs4980079 ITU & BEB 0.28344 0.00351

rs4980079 ITU & GIH 0.49457 0.00368

rs4980079 PJL & ACB 0.27788 0.00385

rs4980079 PJL & ASW 0.07129 0.00168

rs4980079 PJL & ESN 0.34021 0.00371

rs4980079 PJL & GWD 0.00000 0.00000

rs4980079 PJL & LWK 0.00001 0.00001

rs4980079 PJL & MSL 0.00000 0.00000

rs4980079 PJL & YRI 0.03593 0.00125

rs4980079 PJL & PUR 0.00243 0.00032

rs4980079 PJL & CLM 0.02709 0.00121

rs4980079 PJL & PEL 0.62410 0.00268

rs4980079 PJL & MXL 0.16355 0.00238

rs4980079 PJL & CHS 0.01765 0.00097

rs4980079 PJL & CDX 0.11637 0.00239

rs4980079 PJL & KHV 0.11864 0.00235

rs4980079 PJL & CHB 0.64863 0.00311

rs4980079 PJL & JPT 0.00305 0.00033

rs4980079 PJL & CEU 0.00000 0.00000

rs4980079 PJL & FIN 0.02233 0.00109

rs4980079 PJL & GBR 0.00132 0.00020

rs4980079 PJL & IBS 0.00050 0.00014

rs4980079 PJL & TSI 0.00056 0.00011

rs4980079 PJL & BEB 0.64508 0.00253

rs4980079 PJL & GIH 0.90891 0.00083

rs4980079 PJL & ITU 0.64539 0.00215

rs4980079 STU & ACB 0.71477 0.00232

rs4980079 STU & ASW 0.25633 0.00271

rs4980079 STU & ESN 0.90385 0.00086

rs4980079 STU & GWD 0.00003 0.00003

rs4980079 STU & LWK 0.00042 0.00008

rs4980079 STU & MSL 0.00002 0.00001

rs4980079 STU & YRI 0.17770 0.00288

rs4980079 STU & PUR 0.00017 0.00008

rs4980079 STU & CLM 0.00253 0.00034

rs4980079 STU & PEL 0.90024 0.00091

rs4980079 STU & MXL 0.03875 0.00123

rs4980079 STU & CHS 0.00121 0.00020

rs4980079 STU & CDX 0.01798 0.00098

rs4980079 STU & KHV 0.01810 0.00097

rs4980079 STU & CHB 0.21649 0.00327

rs4980079 STU & JPT 0.00018 0.00006

rs4980079 STU & CEU 0.00000 0.00000

rs4980079 STU & FIN 0.00228 0.00035

rs4980079 STU & GBR 0.00007 0.00003

rs4980079 STU & IBS 0.00000 0.00000

rs4980079 STU & TSI 0.00003 0.00002

rs4980079 STU & BEB 0.23146 0.00332

rs4980079 STU & GIH 0.36315 0.00398

rs4980079 STU & ITU 0.90611 0.00080

rs4980079 STU & PJL 0.48052 0.00344

Locus: rs6857

=================================

Pop Alleles:

-----------------------------------------------------------

2 4 Total

ACB 181 11 192

ASW 110 12 122

ESN 193 5 198

GWD 214 12 226

LWK 182 16 198

MSL 164 6 170

YRI 208 8 216

PUR 174 34 208

CLM 172 16 188

PEL 160 10 170

MXL 115 13 128

CHS 183 27 210

CDX 163 23 186

KHV 188 10 198

CHB 182 24 206

JPT 191 17 208

CEU 170 28 198

FIN 162 36 198

GBR 145 37 182

IBS 174 40 214

TSI 189 25 214

BEB 146 26 172

GIH 172 34 206

ITU 175 29 204

PJL 161 31 192

STU 181 23 204

Total: 4455553 5008

Locus Population pair P-Value S.E.

----------- --------------------- ------- -------

rs6857 ASW & ACB 0.18669 0.00189

rs6857 ESN & ACB 0.13142 0.00129

rs6857 ESN & ASW 0.00765 0.00030

rs6857 GWD & ACB 1.00000 0.00000

rs6857 GWD & ASW 0.18091 0.00150

rs6857 GWD & ESN 0.21576 0.00165

rs6857 LWK & ACB 0.42803 0.00200

rs6857 LWK & ASW 0.68569 0.00149

rs6857 LWK & ESN 0.02346 0.00064

rs6857 LWK & GWD 0.32457 0.00210

rs6857 MSL & ACB 0.45826 0.00166

rs6857 MSL & ASW 0.04497 0.00081

rs6857 MSL & ESN 0.76132 0.00070

rs6857 MSL & GWD 0.47066 0.00174

rs6857 MSL & LWK 0.08002 0.00122

rs6857 YRI & ACB 0.35605 0.00173

rs6857 YRI & ASW 0.02987 0.00079

rs6857 YRI & ESN 0.57886 0.00125

rs6857 YRI & GWD 0.49721 0.00226

rs6857 YRI & LWK 0.08798 0.00127

rs6857 YRI & MSL 1.00000 0.00000

rs6857 PUR & ACB 0.00072 0.00011

rs6857 PUR & ASW 0.09981 0.00183

rs6857 PUR & ESN 0.00000 0.00000

rs6857 PUR & GWD 0.00018 0.00004

rs6857 PUR & LWK 0.01525 0.00066

rs6857 PUR & MSL 0.00002 0.00001

rs6857 PUR & YRI 0.00001 0.00001

rs6857 CLM & ACB 0.32161 0.00190

rs6857 CLM & ASW 0.84037 0.00087

rs6857 CLM & ESN 0.01186 0.00038

rs6857 CLM & GWD 0.24023 0.00231

rs6857 CLM & LWK 1.00000 0.00000

rs6857 CLM & MSL 0.07652 0.00135

rs6857 CLM & YRI 0.05733 0.00094

rs6857 CLM & PUR 0.02145 0.00084

rs6857 PEL & ACB 1.00000 0.00000

rs6857 PEL & ASW 0.26715 0.00201

rs6857 PEL & ESN 0.11846 0.00129

rs6857 PEL & GWD 0.82903 0.00083

rs6857 PEL & LWK 0.42253 0.00194

rs6857 PEL & MSL 0.44250 0.00171

rs6857 PEL & YRI 0.33712 0.00176

rs6857 PEL & PUR 0.00208 0.00019

rs6857 PEL & CLM 0.41432 0.00211

rs6857 MXL & ACB 0.19232 0.00192

rs6857 MXL & ASW 1.00000 0.00000

rs6857 MXL & ESN 0.00505 0.00025

rs6857 MXL & GWD 0.13122 0.00178

rs6857 MXL & LWK 0.55037 0.00196

rs6857 MXL & MSL 0.03041 0.00069

rs6857 MXL & YRI 0.03485 0.00070

rs6857 MXL & PUR 0.14506 0.00186

rs6857 MXL & CLM 0.69390 0.00147

rs6857 MXL & PEL 0.19411 0.00170

rs6857 CHS & ACB 0.01635 0.00059

rs6857 CHS & ASW 0.48074 0.00229

rs6857 CHS & ESN 0.00006 0.00002

rs6857 CHS & GWD 0.00726 0.00044

rs6857 CHS & LWK 0.14636 0.00197

rs6857 CHS & MSL 0.00177 0.00018

rs6857 CHS & YRI 0.00068 0.00011

rs6857 CHS & PUR 0.33321 0.00344

rs6857 CHS & CLM 0.19456 0.00201

rs6857 CHS & PEL 0.02600 0.00078

rs6857 CHS & MXL 0.49320 0.00221

rs6857 CDX & ACB 0.03144 0.00089

rs6857 CDX & ASW 0.58577 0.00207

rs6857 CDX & ESN 0.00025 0.00005

rs6857 CDX & GWD 0.01336 0.00050

rs6857 CDX & LWK 0.18005 0.00216

rs6857 CDX & MSL 0.00253 0.00019

rs6857 CDX & YRI 0.00121 0.00012

rs6857 CDX & PUR 0.31450 0.00258

rs6857 CDX & CLM 0.23923 0.00237

rs6857 CDX & PEL 0.04067 0.00095

rs6857 CDX & MXL 0.59373 0.00181

rs6857 CDX & CHS 1.00000 0.00000

rs6857 KHV & ACB 0.82532 0.00084

rs6857 KHV & ASW 0.11612 0.00150

rs6857 KHV & ESN 0.29370 0.00146

rs6857 KHV & GWD 1.00000 0.00000

rs6857 KHV & LWK 0.30791 0.00192

rs6857 KHV & MSL 0.61100 0.00123

rs6857 KHV & YRI 0.62931 0.00130

rs6857 KHV & PUR 0.00019 0.00007

rs6857 KHV & CLM 0.22364 0.00171

rs6857 KHV & PEL 0.81921 0.00076

rs6857 KHV & MXL 0.12105 0.00154

rs6857 KHV & CHS 0.00959 0.00049

rs6857 KHV & CDX 0.01710 0.00066

rs6857 CHB & ACB 0.05108 0.00107

rs6857 CHB & ASW 0.71768 0.00145

rs6857 CHB & ESN 0.00039 0.00007

rs6857 CHB & GWD 0.02104 0.00074

rs6857 CHB & LWK 0.24511 0.00236

rs6857 CHB & MSL 0.00388 0.00027

rs6857 CHB & YRI 0.00248 0.00020

rs6857 CHB & PUR 0.20590 0.00268

rs6857 CHB & CLM 0.32021 0.00257

rs6857 CHB & PEL 0.06858 0.00125

rs6857 CHB & MXL 0.72311 0.00150

rs6857 CHB & CHS 0.76559 0.00183

rs6857 CHB & CDX 0.87751 0.00089

rs6857 CHB & KHV 0.01987 0.00072

rs6857 JPT & ACB 0.43104 0.00223

rs6857 JPT & ASW 0.68495 0.00144

rs6857 JPT & ESN 0.01490 0.00052

rs6857 JPT & GWD 0.25478 0.00218

rs6857 JPT & LWK 1.00000 0.00000

rs6857 JPT & MSL 0.08331 0.00128

rs6857 JPT & YRI 0.06263 0.00103

rs6857 JPT & PUR 0.01583 0.00066

rs6857 JPT & CLM 1.00000 0.00000

rs6857 JPT & PEL 0.43176 0.00206

rs6857 JPT & MXL 0.55808 0.00203

rs6857 JPT & CHS 0.15239 0.00229

rs6857 JPT & CDX 0.18505 0.00231

rs6857 JPT & KHV 0.23720 0.00186

rs6857 JPT & CHB 0.25004 0.00208

rs6857 CEU & ACB 0.00621 0.00037

rs6857 CEU & ASW 0.29400 0.00256

rs6857 CEU & ESN 0.00003 0.00002

rs6857 CEU & GWD 0.00226 0.00019

rs6857 CEU & LWK 0.07826 0.00157

rs6857 CEU & MSL 0.00048 0.00008

rs6857 CEU & YRI 0.00015 0.00005

rs6857 CEU & PUR 0.58165 0.00272

rs6857 CEU & CLM 0.10756 0.00188

rs6857 CEU & PEL 0.00988 0.00048

rs6857 CEU & MXL 0.30726 0.00229

rs6857 CEU & CHS 0.77352 0.00148

rs6857 CEU & CDX 0.65219 0.00215

rs6857 CEU & KHV 0.00351 0.00028

rs6857 CEU & CHB 0.46575 0.00252

rs6857 CEU & JPT 0.05718 0.00119

rs6857 FIN & ACB 0.00018 0.00006

rs6857 FIN & ASW 0.05531 0.00130

rs6857 FIN & ESN 0.00000 0.00000

rs6857 FIN & GWD 0.00001 0.00000

rs6857 FIN & LWK 0.00431 0.00033

rs6857 FIN & MSL 0.00000 0.00000

rs6857 FIN & YRI 0.00000 0.00000

rs6857 FIN & PUR 0.69649 0.00207

rs6857 FIN & CLM 0.00694 0.00046

rs6857 FIN & PEL 0.00038 0.00007

rs6857 FIN & MXL 0.05762 0.00137

rs6857 FIN & CHS 0.17539 0.00262

rs6857 FIN & CDX 0.12185 0.00232

rs6857 FIN & KHV 0.00010 0.00004

rs6857 FIN & CHB 0.06929 0.00163

rs6857 FIN & JPT 0.00274 0.00025

rs6857 FIN & CEU 0.34239 0.00298

rs6857 GBR & ACB 0.00003 0.00001

rs6857 GBR & ASW 0.01753 0.00071

rs6857 GBR & ESN 0.00000 0.00000

rs6857 GBR & GWD 0.00000 0.00000

rs6857 GBR & LWK 0.00058 0.00010

rs6857 GBR & MSL 0.00000 0.00000

rs6857 GBR & YRI 0.00000 0.00000

rs6857 GBR & PUR 0.36099 0.00346

rs6857 GBR & CLM 0.00170 0.00019

rs6857 GBR & PEL 0.00004 0.00002

rs6857 GBR & MXL 0.01909 0.00069

rs6857 GBR & CHS 0.05434 0.00153

rs6857 GBR & CDX 0.04767 0.00152

rs6857 GBR & KHV 0.00001 0.00001

rs6857 GBR & CHB 0.02495 0.00094

rs6857 GBR & JPT 0.00053 0.00010

rs6857 GBR & CEU 0.13122 0.00229

rs6857 GBR & FIN 0.60551 0.00260

rs6857 IBS & ACB 0.00011 0.00004

rs6857 IBS & ASW 0.03096 0.00109

rs6857 IBS & ESN 0.00000 0.00000

rs6857 IBS & GWD 0.00002 0.00001

rs6857 IBS & LWK 0.00228 0.00026

rs6857 IBS & MSL 0.00000 0.00000

rs6857 IBS & YRI 0.00000 0.00000

rs6857 IBS & PUR 0.60653 0.00264

rs6857 IBS & CLM 0.00392 0.00029

rs6857 IBS & PEL 0.00015 0.00005

rs6857 IBS & MXL 0.04573 0.00145

rs6857 IBS & CHS 0.11148 0.00234

rs6857 IBS & CDX 0.09857 0.00211

rs6857 IBS & KHV 0.00001 0.00001

rs6857 IBS & CHB 0.05588 0.00156

rs6857 IBS & JPT 0.00179 0.00022

rs6857 IBS & CEU 0.23834 0.00338

rs6857 IBS & FIN 0.90051 0.00089

rs6857 IBS & GBR 0.70181 0.00208

rs6857 TSI & ACB 0.03723 0.00101

rs6857 TSI & ASW 0.71717 0.00135

rs6857 TSI & ESN 0.00034 0.00007

rs6857 TSI & GWD 0.02367 0.00076

rs6857 TSI & LWK 0.24865 0.00223

rs6857 TSI & MSL 0.00461 0.00028

rs6857 TSI & YRI 0.00215 0.00018

rs6857 TSI & PUR 0.20693 0.00247

rs6857 TSI & CLM 0.32202 0.00245

rs6857 TSI & PEL 0.05135 0.00119

rs6857 TSI & MXL 0.72445 0.00170

rs6857 TSI & CHS 0.76604 0.00175

rs6857 TSI & CDX 0.87895 0.00088

rs6857 TSI & KHV 0.02047 0.00069

rs6857 TSI & CHB 1.00000 0.00000

rs6857 TSI & JPT 0.25508 0.00257

rs6857 TSI & CEU 0.46439 0.00273

rs6857 TSI & FIN 0.07194 0.00173

rs6857 TSI & GBR 0.02503 0.00078

rs6857 TSI & IBS 0.05893 0.00166

rs6857 BEB & ACB 0.00451 0.00030

rs6857 BEB & ASW 0.22194 0.00211

rs6857 BEB & ESN 0.00002 0.00002

rs6857 BEB & GWD 0.00163 0.00018

rs6857 BEB & LWK 0.04639 0.00118

rs6857 BEB & MSL 0.00030 0.00006

rs6857 BEB & YRI 0.00009 0.00003

rs6857 BEB & PUR 0.78251 0.00167

rs6857 BEB & CLM 0.06726 0.00146

rs6857 BEB & PEL 0.00795 0.00043

rs6857 BEB & MXL 0.22760 0.00222

rs6857 BEB & CHS 0.55220 0.00242

rs6857 BEB & CDX 0.54201 0.00261

rs6857 BEB & KHV 0.00173 0.00018

rs6857 BEB & CHB 0.36498 0.00291

rs6857 BEB & JPT 0.04926 0.00138

rs6857 BEB & CEU 0.88301 0.00081

rs6857 BEB & FIN 0.48810 0.00267

rs6857 BEB & GBR 0.21364 0.00304

rs6857 BEB & IBS 0.41502 0.00278

rs6857 BEB & TSI 0.36511 0.00268

rs6857 GIH & ACB 0.00077 0.00012

rs6857 GIH & ASW 0.10174 0.00158

rs6857 GIH & ESN 0.00000 0.00000

rs6857 GIH & GWD 0.00021 0.00005

rs6857 GIH & LWK 0.01032 0.00046

rs6857 GIH & MSL 0.00005 0.00002

rs6857 GIH & YRI 0.00003 0.00001

rs6857 GIH & PUR 1.00000 0.00000

rs6857 GIH & CLM 0.02219 0.00085

rs6857 GIH & PEL 0.00137 0.00016

rs6857 GIH & MXL 0.10859 0.00190

rs6857 GIH & CHS 0.32933 0.00321

rs6857 GIH & CDX 0.25314 0.00265

rs6857 GIH & KHV 0.00014 0.00004

rs6857 GIH & CHB 0.20418 0.00237

rs6857 GIH & JPT 0.01141 0.00063

rs6857 GIH & CEU 0.57793 0.00248

rs6857 GIH & FIN 0.69629 0.00197

rs6857 GIH & GBR 0.35728 0.00301

rs6857 GIH & IBS 0.61072 0.00287

rs6857 GIH & TSI 0.16600 0.00216

rs6857 GIH & BEB 0.77644 0.00177

rs6857 ITU & ACB 0.00686 0.00037

rs6857 ITU & ASW 0.30509 0.00230

rs6857 ITU & ESN 0.00002 0.00001

rs6857 ITU & GWD 0.00320 0.00025

rs6857 ITU & LWK 0.05915 0.00153

rs6857 ITU & MSL 0.00036 0.00007

rs6857 ITU & YRI 0.00014 0.00004

rs6857 ITU & PUR 0.58433 0.00257

rs6857 ITU & CLM 0.08340 0.00154

rs6857 ITU & PEL 0.01023 0.00049

rs6857 ITU & MXL 0.31339 0.00251

rs6857 ITU & CHS 0.77288 0.00142

rs6857 ITU & CDX 0.65624 0.00215

rs6857 ITU & KHV 0.00187 0.00018

rs6857 ITU & CHB 0.46617 0.00262

rs6857 ITU & JPT 0.06060 0.00147

rs6857 ITU & CEU 1.00000 0.00000

rs6857 ITU & FIN 0.34654 0.00305

rs6857 ITU & GBR 0.13922 0.00261

rs6857 ITU & IBS 0.23169 0.00260

rs6857 ITU & TSI 0.46781 0.00279

rs6857 ITU & BEB 0.88384 0.00082

rs6857 ITU & GIH 0.58446 0.00257

rs6857 PJL & ACB 0.00143 0.00016

rs6857 PJL & ASW 0.13370 0.00184

rs6857 PJL & ESN 0.00000 0.00000

rs6857 PJL & GWD 0.00033 0.00006

rs6857 PJL & LWK 0.01875 0.00076

rs6857 PJL & MSL 0.00004 0.00002

rs6857 PJL & YRI 0.00008 0.00004

rs6857 PJL & PUR 1.00000 0.00000

rs6857 PJL & CLM 0.03049 0.00086

rs6857 PJL & PEL 0.00257 0.00023

rs6857 PJL & MXL 0.13889 0.00190

rs6857 PJL & CHS 0.39260 0.00297

rs6857 PJL & CDX 0.31024 0.00285

rs6857 PJL & KHV 0.00033 0.00006

rs6857 PJL & CHB 0.24636 0.00263

rs6857 PJL & JPT 0.02075 0.00071

rs6857 PJL & CEU 0.66912 0.00210

rs6857 PJL & FIN 0.68836 0.00227

rs6857 PJL & GBR 0.35276 0.00314

rs6857 PJL & IBS 0.51801 0.00319

rs6857 PJL & TSI 0.20031 0.00245

rs6857 PJL & BEB 0.88435 0.00097

rs6857 PJL & GIH 1.00000 0.00000

rs6857 PJL & ITU 0.67455 0.00228

rs6857 STU & ACB 0.05090 0.00128

rs6857 STU & ASW 0.71413 0.00163

rs6857 STU & ESN 0.00065 0.00010

rs6857 STU & GWD 0.03220 0.00085

rs6857 STU & LWK 0.31715 0.00259

rs6857 STU & MSL 0.00652 0.00035

rs6857 STU & YRI 0.00421 0.00031

rs6857 STU & PUR 0.15547 0.00255

rs6857 STU & CLM 0.40249 0.00219

rs6857 STU & PEL 0.06993 0.00137

rs6857 STU & MXL 0.85682 0.00087

rs6857 STU & CHS 0.65351 0.00201

rs6857 STU & CDX 0.75511 0.00129

rs6857 STU & KHV 0.02757 0.00089

rs6857 STU & CHB 1.00000 0.00000

rs6857 STU & JPT 0.32155 0.00253

rs6857 STU & CEU 0.46095 0.00279

rs6857 STU & FIN 0.06545 0.00174

rs6857 STU & GBR 0.01753 0.00083

rs6857 STU & IBS 0.03883 0.00128

rs6857 STU & TSI 1.00000 0.00000

rs6857 STU & BEB 0.28436 0.00275

rs6857 STU & GIH 0.14968 0.00228

rs6857 STU & ITU 0.45599 0.00288

rs6857 STU & PJL 0.19108 0.00247

Locus: rs3110643

=================================

Pop Alleles:

-----------------------------------------------------------

2 4 Total

ACB 20 172 192

ASW 19 103 122

ESN 21 177 198

GWD 12 214 226

LWK 17 181 198

MSL 7 163 170

YRI 16 200 216

PUR 23 185 208

CLM 18 170 188

PEL 14 156 170

MXL 19 109 128

CHS 0 210 210

CDX 1 185 186

KHV 0 198 198

CHB 0 206 206

JPT 0 208 208

CEU 36 162 198

FIN 49 149 198

GBR 32 150 182

IBS 24 190 214

TSI 34 180 214

BEB 5 167 172

GIH 12 194 206

ITU 14 190 204

PJL 14 178 192

STU 7 197 204

Total: 414 4594 5008

Locus Population pair P-Value S.E.

----------- --------------------- ------- -------

rs3110643 ASW & ACB 0.22061 0.00219

rs3110643 ESN & ACB 1.00000 0.00000

rs3110643 ESN & ASW 0.22355 0.00213

rs3110643 GWD & ACB 0.06418 0.00115

rs3110643 GWD & ASW 0.00294 0.00024

rs3110643 GWD & ESN 0.04679 0.00114

rs3110643 LWK & ACB 0.60497 0.00200

rs3110643 LWK & ASW 0.06861 0.00134

rs3110643 LWK & ESN 0.61147 0.00210

rs3110643 LWK & GWD 0.24566 0.00234

rs3110643 MSL & ACB 0.02716 0.00077

rs3110643 MSL & ASW 0.00132 0.00014

rs3110643 MSL & ESN 0.02802 0.00075

rs3110643 MSL & GWD 0.64360 0.00157

rs3110643 MSL & LWK 0.09481 0.00130

rs3110643 YRI & ACB 0.29436 0.00240

rs3110643 YRI & ASW 0.02480 0.00075

rs3110643 YRI & ESN 0.30317 0.00244

rs3110643 YRI & GWD 0.43658 0.00207

rs3110643 YRI & LWK 0.71605 0.00139

rs3110643 YRI & MSL 0.20164 0.00177

rs3110643 PUR & ACB 0.87169 0.00075

rs3110643 PUR & ASW 0.30755 0.00234

rs3110643 PUR & ESN 1.00000 0.00000

rs3110643 PUR & GWD 0.03422 0.00101

rs3110643 PUR & LWK 0.41173 0.00222

rs3110643 PUR & MSL 0.01335 0.00057

rs3110643 PUR & YRI 0.24112 0.00225

rs3110643 CLM & ACB 0.86250 0.00097

rs3110643 CLM & ASW 0.14977 0.00186

rs3110643 CLM & ESN 0.86651 0.00073

rs3110643 CLM & GWD 0.12357 0.00164

rs3110643 CLM & LWK 0.85832 0.00081

rs3110643 CLM & MSL 0.05972 0.00104

rs3110643 CLM & YRI 0.47696 0.00226

rs3110643 CLM & PUR 0.74167 0.00139

rs3110643 PEL & ACB 0.58638 0.00175

rs3110643 PEL & ASW 0.06373 0.00142

rs3110643 PEL & ESN 0.47955 0.00223

rs3110643 PEL & GWD 0.30326 0.00217

rs3110643 PEL & LWK 1.00000 0.00000

rs3110643 PEL & MSL 0.17657 0.00154

rs3110643 PEL & YRI 0.84778 0.00078

rs3110643 PEL & PUR 0.39161 0.00255

rs3110643 PEL & CLM 0.71503 0.00151

rs3110643 MXL & ACB 0.29972 0.00250

rs3110643 MXL & ASW 1.00000 0.00000

rs3110643 MXL & ESN 0.29877 0.00214

rs3110643 MXL & GWD 0.00552 0.00029

rs3110643 MXL & LWK 0.10641 0.00157

rs3110643 MXL & MSL 0.00186 0.00018

rs3110643 MXL & YRI 0.04047 0.00102

rs3110643 MXL & PUR 0.39806 0.00272

rs3110643 MXL & CLM 0.21072 0.00229

rs3110643 MXL & PEL 0.09213 0.00173

rs3110643 CHS & ACB 0.00001 0.00001

rs3110643 CHS & ASW 0.00000 0.00000

rs3110643 CHS & ESN 0.00000 0.00000

rs3110643 CHS & GWD 0.00047 0.00006

rs3110643 CHS & LWK 0.00001 0.00001

rs3110643 CHS & MSL 0.00322 0.00016

rs3110643 CHS & YRI 0.00003 0.00001

rs3110643 CHS & PUR 0.00000 0.00000

rs3110643 CHS & CLM 0.00000 0.00000

rs3110643 CHS & PEL 0.00001 0.00001

rs3110643 CHS & MXL 0.00000 0.00000

rs3110643 CDX & ACB 0.00000 0.00000

rs3110643 CDX & ASW 0.00000 0.00000

rs3110643 CDX & ESN 0.00004 0.00002

rs3110643 CDX & GWD 0.00456 0.00022

rs3110643 CDX & LWK 0.00018 0.00005

rs3110643 CDX & MSL 0.03032 0.00052

rs3110643 CDX & YRI 0.00036 0.00006

rs3110643 CDX & PUR 0.00000 0.00000

rs3110643 CDX & CLM 0.00005 0.00002

rs3110643 CDX & PEL 0.00027 0.00005

rs3110643 CDX & MXL 0.00000 0.00000

rs3110643 CDX & CHS 0.46973 0.00083

rs3110643 KHV & ACB 0.00000 0.00000

rs3110643 KHV & ASW 0.00000 0.00000

rs3110643 KHV & ESN 0.00000 0.00000

rs3110643 KHV & GWD 0.00053 0.00006

rs3110643 KHV & LWK 0.00001 0.00001

rs3110643 KHV & MSL 0.00404 0.00016

rs3110643 KHV & YRI 0.00002 0.00001

rs3110643 KHV & PUR 0.00000 0.00000

rs3110643 KHV & CLM 0.00000 0.00000

rs3110643 KHV & PEL 0.00001 0.00001

rs3110643 KHV & MXL 0.00000 0.00000

rs3110643 KHV & CHS No table

rs3110643 KHV & CDX 0.48249 0.00060

rs3110643 CHB & ACB 0.00000 0.00000

rs3110643 CHB & ASW 0.00000 0.00000

rs3110643 CHB & ESN 0.00000 0.00000

rs3110643 CHB & GWD 0.00046 0.00006

rs3110643 CHB & LWK 0.00001 0.00001

rs3110643 CHB & MSL 0.00374 0.00016

rs3110643 CHB & YRI 0.00003 0.00001

rs3110643 CHB & PUR 0.00000 0.00000

rs3110643 CHB & CLM 0.00001 0.00001

rs3110643 CHB & PEL 0.00000 0.00000

rs3110643 CHB & MXL 0.00000 0.00000

rs3110643 CHB & CHS No table

rs3110643 CHB & CDX 0.47453 0.00079

rs3110643 CHB & KHV No table

rs3110643 JPT & ACB 0.00000 0.00000

rs3110643 JPT & ASW 0.00000 0.00000

rs3110643 JPT & ESN 0.00000 0.00000

rs3110643 JPT & GWD 0.00043 0.00005

rs3110643 JPT & LWK 0.00001 0.00001

rs3110643 JPT & MSL 0.00353 0.00015

rs3110643 JPT & YRI 0.00004 0.00002

rs3110643 JPT & PUR 0.00000 0.00000

rs3110643 JPT & CLM 0.00000 0.00000

rs3110643 JPT & PEL 0.00001 0.00000

rs3110643 JPT & MXL 0.00000 0.00000

rs3110643 JPT & CHS No table

rs3110643 JPT & CDX 0.47253 0.00082

rs3110643 JPT & KHV No table

rs3110643 JPT & CHB No table

rs3110643 CEU & ACB 0.03112 0.00100

rs3110643 CEU & ASW 0.65117 0.00220

rs3110643 CEU & ESN 0.04465 0.00125

rs3110643 CEU & GWD 0.00003 0.00001

rs3110643 CEU & LWK 0.00786 0.00041

rs3110643 CEU & MSL 0.00002 0.00001

rs3110643 CEU & YRI 0.00155 0.00023

rs3110643 CEU & PUR 0.04820 0.00118

rs3110643 CEU & CLM 0.01834 0.00077

rs3110643 CEU & PEL 0.00576 0.00039

rs3110643 CEU & MXL 0.45330 0.00268

rs3110643 CEU & CHS 0.00000 0.00000

rs3110643 CEU & CDX 0.00000 0.00000

rs3110643 CEU & KHV 0.00000 0.00000

rs3110643 CEU & CHB 0.00000 0.00000

rs3110643 CEU & JPT 0.00000 0.00000

rs3110643 FIN & ACB 0.00031 0.00007

rs3110643 FIN & ASW 0.05055 0.00133

rs3110643 FIN & ESN 0.00051 0.00012

rs3110643 FIN & GWD 0.00000 0.00000

rs3110643 FIN & LWK 0.00001 0.00001

rs3110643 FIN & MSL 0.00000 0.00000

rs3110643 FIN & YRI 0.00000 0.00000

rs3110643 FIN & PUR 0.00038 0.00011

rs3110643 FIN & CLM 0.00002 0.00001

rs3110643 FIN & PEL 0.00006 0.00004

rs3110643 FIN & MXL 0.03738 0.00133

rs3110643 FIN & CHS 0.00000 0.00000

rs3110643 FIN & CDX 0.00000 0.00000

rs3110643 FIN & KHV 0.00000 0.00000

rs3110643 FIN & CHB 0.00000 0.00000

rs3110643 FIN & JPT 0.00000 0.00000

rs3110643 FIN & CEU 0.13852 0.00235

rs3110643 GBR & ACB 0.05069 0.00119

rs3110643 GBR & ASW 0.75575 0.00164

rs3110643 GBR & ESN 0.05226 0.00128

rs3110643 GBR & GWD 0.00015 0.00006

rs3110643 GBR & LWK 0.01447 0.00072

rs3110643 GBR & MSL 0.00003 0.00001

rs3110643 GBR & YRI 0.00277 0.00023

rs3110643 GBR & PUR 0.08183 0.00161

rs3110643 GBR & CLM 0.03302 0.00109

rs3110643 GBR & PEL 0.01135 0.00051

rs3110643 GBR & MXL 0.53824 0.00238

rs3110643 GBR & CHS 0.00000 0.00000

rs3110643 GBR & CDX 0.00000 0.00000

rs3110643 GBR & KHV 0.00000 0.00000

rs3110643 GBR & CHB 0.00000 0.00000

rs3110643 GBR & JPT 0.00000 0.00000

rs3110643 GBR & CEU 0.89564 0.00082

rs3110643 GBR & FIN 0.10451 0.00251

rs3110643 IBS & ACB 0.87516 0.00085

rs3110643 IBS & ASW 0.30625 0.00250

rs3110643 IBS & ESN 0.87521 0.00087

rs3110643 IBS & GWD 0.03510 0.00103

rs3110643 IBS & LWK 0.41762 0.00266

rs3110643 IBS & MSL 0.01257 0.00049

rs3110643 IBS & YRI 0.18980 0.00236

rs3110643 IBS & PUR 1.00000 0.00000

rs3110643 IBS & CLM 0.62837 0.00163

rs3110643 IBS & PEL 0.38989 0.00236

rs3110643 IBS & MXL 0.40491 0.00280

rs3110643 IBS & CHS 0.00000 0.00000

rs3110643 IBS & CDX 0.00001 0.00001

rs3110643 IBS & KHV 0.00000 0.00000

rs3110643 IBS & CHB 0.00000 0.00000

rs3110643 IBS & JPT 0.00000 0.00000

rs3110643 IBS & CEU 0.05157 0.00134

rs3110643 IBS & FIN 0.00038 0.00010

rs3110643 IBS & GBR 0.08432 0.00187

rs3110643 TSI & ACB 0.10895 0.00178

rs3110643 TSI & ASW 1.00000 0.00000

rs3110643 TSI & ESN 0.14670 0.00207

rs3110643 TSI & GWD 0.00031 0.00008

rs3110643 TSI & LWK 0.02445 0.00091

rs3110643 TSI & MSL 0.00016 0.00005

rs3110643 TSI & YRI 0.00697 0.00038

rs3110643 TSI & PUR 0.15956 0.00240

rs3110643 TSI & CLM 0.07567 0.00165

rs3110643 TSI & PEL 0.02790 0.00098

rs3110643 TSI & MXL 0.87794 0.00087

rs3110643 TSI & CHS 0.00000 0.00000

rs3110643 TSI & CDX 0.00000 0.00000

rs3110643 TSI & KHV 0.00000 0.00000

rs3110643 TSI & CHB 0.00000 0.00000

rs3110643 TSI & JPT 0.00000 0.00000

rs3110643 TSI & CEU 0.59713 0.00295

rs3110643 TSI & FIN 0.02824 0.00117

rs3110643 TSI & GBR 0.68691 0.00209

rs3110643 TSI & IBS 0.20334 0.00276

rs3110643 BEB & ACB 0.00610 0.00033

rs3110643 BEB & ASW 0.00012 0.00003

rs3110643 BEB & ESN 0.00386 0.00022

rs3110643 BEB & GWD 0.31929 0.00152

rs3110643 BEB & LWK 0.02618 0.00073

rs3110643 BEB & MSL 0.57160 0.00144

rs3110643 BEB & YRI 0.07138 0.00101

rs3110643 BEB & PUR 0.00251 0.00017

rs3110643 BEB & CLM 0.01069 0.00041

rs3110643 BEB & PEL 0.03548 0.00073

rs3110643 BEB & MXL 0.00031 0.00005

rs3110643 BEB & CHS 0.01776 0.00033

rs3110643 BEB & CDX 0.10887 0.00085

rs3110643 BEB & KHV 0.02070 0.00042

rs3110643 BEB & CHB 0.01811 0.00035

rs3110643 BEB & JPT 0.01865 0.00038

rs3110643 BEB & CEU 0.00000 0.00000

rs3110643 BEB & FIN 0.00000 0.00000

rs3110643 BEB & GBR 0.00000 0.00000

rs3110643 BEB & IBS 0.00197 0.00019

rs3110643 BEB & TSI 0.00002 0.00001

rs3110643 GIH & ACB 0.09885 0.00164

rs3110643 GIH & ASW 0.00631 0.00036

rs3110643 GIH & ESN 0.10247 0.00147

rs3110643 GIH & GWD 0.83795 0.00081

rs3110643 GIH & LWK 0.33596 0.00215

rs3110643 GIH & MSL 0.48955 0.00170

rs3110643 GIH & YRI 0.56118 0.00181

rs3110643 GIH & PUR 0.07732 0.00149

rs3110643 GIH & CLM 0.18612 0.00220

rs3110643 GIH & PEL 0.42064 0.00175

rs3110643 GIH & MXL 0.00957 0.00041

rs3110643 GIH & CHS 0.00018 0.00003

rs3110643 GIH & CDX 0.00301 0.00017

rs3110643 GIH & KHV 0.00042 0.00006

rs3110643 GIH & CHB 0.00042 0.00005

rs3110643 GIH & JPT 0.00023 0.00004

rs3110643 GIH & CEU 0.00018 0.00005

rs3110643 GIH & FIN 0.00000 0.00000

rs3110643 GIH & GBR 0.00020 0.00006

rs3110643 GIH & IBS 0.05737 0.00127

rs3110643 GIH & TSI 0.00110 0.00016

rs3110643 GIH & BEB 0.21197 0.00169

rs3110643 ITU & ACB 0.21575 0.00210

rs3110643 ITU & ASW 0.02006 0.00064

rs3110643 ITU & ESN 0.21538 0.00203

rs3110643 ITU & GWD 0.54633 0.00184

rs3110643 ITU & LWK 0.57630 0.00194

rs3110643 ITU & MSL 0.26955 0.00169

rs3110643 ITU & YRI 0.85320 0.00076

rs3110643 ITU & PUR 0.16796 0.00214

rs3110643 ITU & CLM 0.36212 0.00252

rs3110643 ITU & PEL 0.69513 0.00143

rs3110643 ITU & MXL 0.02275 0.00068

rs3110643 ITU & CHS 0.00005 0.00002

rs3110643 ITU & CDX 0.00083 0.00007

rs3110643 ITU & KHV 0.00007 0.00002

rs3110643 ITU & CHB 0.00003 0.00001

rs3110643 ITU & JPT 0.00002 0.00001

rs3110643 ITU & CEU 0.00051 0.00010

rs3110643 ITU & FIN 0.00000 0.00000

rs3110643 ITU & GBR 0.00155 0.00019

rs3110643 ITU & IBS 0.13008 0.00196

rs3110643 ITU & TSI 0.00520 0.00034

rs3110643 ITU & BEB 0.10090 0.00126

rs3110643 ITU & GIH 0.69309 0.00143

rs3110643 PJL & ACB 0.37616 0.00217

rs3110643 PJL & ASW 0.02362 0.00081

rs3110643 PJL & ESN 0.29185 0.00251

rs3110643 PJL & GWD 0.42595 0.00201

rs3110643 PJL & LWK 0.70900 0.00141

rs3110643 PJL & MSL 0.26227 0.00164

rs3110643 PJL & YRI 1.00000 0.00000

rs3110643 PJL & PUR 0.22744 0.00249

rs3110643 PJL & CLM 0.46464 0.00224

rs3110643 PJL & PEL 0.84457 0.00080

rs3110643 PJL & MXL 0.03945 0.00101

rs3110643 PJL & CHS 0.00002 0.00001

rs3110643 PJL & CDX 0.00097 0.00010

rs3110643 PJL & KHV 0.00003 0.00002

rs3110643 PJL & CHB 0.00001 0.00001

rs3110643 PJL & JPT 0.00002 0.00001

rs3110643 PJL & CEU 0.00145 0.00017

rs3110643 PJL & FIN 0.00000 0.00000

rs3110643 PJL & GBR 0.00261 0.00025

rs3110643 PJL & IBS 0.23047 0.00238

rs3110643 PJL & TSI 0.00907 0.00044

rs3110643 PJL & BEB 0.06392 0.00093

rs3110643 PJL & GIH 0.68711 0.00150

rs3110643 PJL & ITU 1.00000 0.00000

rs3110643 STU & ACB 0.00848 0.00036

rs3110643 STU & ASW 0.00014 0.00005

rs3110643 STU & ESN 0.00574 0.00034

rs3110643 STU & GWD 0.35919 0.00184

rs3110643 STU & LWK 0.03543 0.00083

rs3110643 STU & MSL 0.78834 0.00073

rs3110643 STU & YRI 0.08769 0.00124

rs3110643 STU & PUR 0.00381 0.00023

rs3110643 STU & CLM 0.02105 0.00073

rs3110643 STU & PEL 0.06911 0.00111

rs3110643 STU & MXL 0.00061 0.00007

rs3110643 STU & CHS 0.00682 0.00023

rs3110643 STU & CDX 0.07012 0.00072

rs3110643 STU & KHV 0.01468 0.00031

rs3110643 STU & CHB 0.00712 0.00023

rs3110643 STU & JPT 0.00662 0.00020

rs3110643 STU & CEU 0.00000 0.00000

rs3110643 STU & FIN 0.00000 0.00000

rs3110643 STU & GBR 0.00000 0.00000

rs3110643 STU & IBS 0.00237 0.00020

rs3110643 STU & TSI 0.00001 0.00000

rs3110643 STU & BEB 1.00000 0.00000

rs3110643 STU & GIH 0.35143 0.00191

rs3110643 STU & ITU 0.17717 0.00159

rs3110643 STU & PJL 0.11402 0.00150

Locus: rs10816848

=================================

Pop Alleles:

-----------------------------------------------------------

1 4 Total

ACB 77 115 192

ASW 46 76 122

ESN 86 112 198

GWD 93 133 226

LWK 94 104 198

MSL 69 101 170

YRI 80 136 216

PUR 77 131 208

CLM 46 142 188

PEL 52 118 170

MXL 63 65 128

CHS 44 166 210

CDX 45 141 186

KHV 43 155 198

CHB 65 141 206

JPT 37 171 208

CEU 100 98 198

FIN 69 129 198

GBR 83 99 182

IBS 109 105 214

TSI 94 120 214

BEB 35 137 172

GIH 46 160 206

ITU 59 145 204

PJL 72 120 192

STU 50 154 204

Total: 17343274 5008

Locus Population pair P-Value S.E.

----------- --------------------- ------- -------

rs10816848 ASW & ACB 0.72331 0.00194

rs10816848 ESN & ACB 0.53711 0.00321

rs10816848 ESN & ASW 0.34921 0.00343

rs10816848 GWD & ACB 0.84257 0.00160

rs10816848 GWD & ASW 0.57664 0.00232

rs10816848 GWD & ESN 0.69425 0.00264

rs10816848 LWK & ACB 0.15362 0.00370

rs10816848 LWK & ASW 0.10669 0.00246

rs10816848 LWK & ESN 0.48472 0.00387

rs10816848 LWK & GWD 0.19734 0.00358

rs10816848 MSL & ACB 1.00000 0.00000

rs10816848 MSL & ASW 0.62996 0.00278

rs10816848 MSL & ESN 0.59580 0.00352

rs10816848 MSL & GWD 0.91673 0.00088

rs10816848 MSL & LWK 0.20056 0.00300

rs10816848 YRI & ACB 0.53690 0.00361

rs10816848 YRI & ASW 0.90770 0.00083

rs10816848 YRI & ESN 0.19168 0.00367

rs10816848 YRI & GWD 0.38405 0.00429

rs10816848 YRI & LWK 0.03612 0.00147

rs10816848 YRI & MSL 0.52915 0.00276

rs10816848 PUR & ACB 0.53538 0.00368

rs10816848 PUR & ASW 0.90569 0.00085

rs10816848 PUR & ESN 0.18844 0.00344

rs10816848 PUR & GWD 0.43411 0.00387

rs10816848 PUR & LWK 0.03363 0.00182

rs10816848 PUR & MSL 0.52846 0.00358

rs10816848 PUR & YRI 1.00000 0.00000

rs10816848 CLM & ACB 0.00155 0.00025

rs10816848 CLM & ASW 0.01452 0.00077

rs10816848 CLM & ESN 0.00003 0.00001

rs10816848 CLM & GWD 0.00027 0.00008

rs10816848 CLM & LWK 0.00000 0.00000

rs10816848 CLM & MSL 0.00149 0.00024

rs10816848 CLM & YRI 0.00670 0.00053

rs10816848 CLM & PUR 0.00904 0.00065

rs10816848 PEL & ACB 0.06474 0.00203

rs10816848 PEL & ASW 0.21103 0.00299

rs10816848 PEL & ESN 0.01320 0.00083

rs10816848 PEL & GWD 0.03468 0.00137

rs10816848 PEL & LWK 0.00081 0.00017

rs10816848 PEL & MSL 0.06850 0.00199

rs10816848 PEL & YRI 0.19831 0.00327

rs10816848 PEL & PUR 0.19386 0.00283

rs10816848 PEL & CLM 0.22987 0.00362

rs10816848 MXL & ACB 0.13285 0.00241

rs10816848 MXL & ASW 0.07568 0.00214

rs10816848 MXL & ESN 0.36521 0.00344

rs10816848 MXL & GWD 0.15114 0.00288

rs10816848 MXL & LWK 0.81890 0.00163

rs10816848 MXL & MSL 0.16167 0.00257

rs10816848 MXL & YRI 0.02969 0.00128

rs10816848 MXL & PUR 0.03005 0.00117

rs10816848 MXL & CLM 0.00001 0.00001

rs10816848 MXL & PEL 0.00131 0.00021

rs10816848 CHS & ACB 0.00008 0.00006

rs10816848 CHS & ASW 0.00184 0.00026

rs10816848 CHS & ESN 0.00000 0.00000

rs10816848 CHS & GWD 0.00000 0.00000

rs10816848 CHS & LWK 0.00000 0.00000

rs10816848 CHS & MSL 0.00000 0.00000

rs10816848 CHS & YRI 0.00018 0.00007

rs10816848 CHS & PUR 0.00039 0.00009

rs10816848 CHS & CLM 0.47659 0.00338

rs10816848 CHS & PEL 0.03350 0.00129

rs10816848 CHS & MXL 0.00000 0.00000

rs10816848 CDX & ACB 0.00084 0.00020

rs10816848 CDX & ASW 0.01489 0.00075

rs10816848 CDX & ESN 0.00004 0.00002

rs10816848 CDX & GWD 0.00043 0.00010

rs10816848 CDX & LWK 0.00001 0.00001

rs10816848 CDX & MSL 0.00131 0.00022

rs10816848 CDX & YRI 0.00688 0.00060

rs10816848 CDX & PUR 0.00603 0.00050

rs10816848 CDX & CLM 1.00000 0.00000

rs10816848 CDX & PEL 0.19297 0.00300

rs10816848 CDX & MXL 0.00000 0.00000

rs10816848 CDX & CHS 0.47439 0.00305

rs10816848 KHV & ACB 0.00005 0.00003

rs10816848 KHV & ASW 0.00340 0.00034

rs10816848 KHV & ESN 0.00000 0.00000

rs10816848 KHV & GWD 0.00003 0.00002

rs10816848 KHV & LWK 0.00000 0.00000

rs10816848 KHV & MSL 0.00015 0.00005

rs10816848 KHV & YRI 0.00089 0.00017

rs10816848 KHV & PUR 0.00072 0.00013

rs10816848 KHV & CLM 0.54363 0.00360

rs10816848 KHV & PEL 0.05846 0.00188

rs10816848 KHV & MXL 0.00000 0.00000

rs10816848 KHV & CHS 0.90293 0.00090

rs10816848 KHV & CDX 0.62837 0.00281

rs10816848 CHB & ACB 0.09190 0.00230

rs10816848 CHB & ASW 0.27884 0.00326

rs10816848 CHB & ESN 0.01880 0.00111

rs10816848 CHB & GWD 0.04262 0.00152

rs10816848 CHB & LWK 0.00125 0.00021

rs10816848 CHB & MSL 0.08418 0.00235

rs10816848 CHB & YRI 0.25733 0.00389

rs10816848 CHB & PUR 0.25507 0.00373

rs10816848 CHB & CLM 0.14237 0.00295

rs10816848 CHB & PEL 0.91239 0.00082

rs10816848 CHB & MXL 0.00198 0.00027

rs10816848 CHB & CHS 0.01538 0.00096

rs10816848 CHB & CDX 0.11367 0.00234

rs10816848 CHB & KHV 0.03309 0.00128

rs10816848 JPT & ACB 0.00000 0.00000

rs10816848 JPT & ASW 0.00006 0.00002

rs10816848 JPT & ESN 0.00000 0.00000

rs10816848 JPT & GWD 0.00000 0.00000

rs10816848 JPT & LWK 0.00000 0.00000

rs10816848 JPT & MSL 0.00000 0.00000

rs10816848 JPT & YRI 0.00008 0.00007

rs10816848 JPT & PUR 0.00000 0.00000

rs10816848 JPT & CLM 0.10885 0.00245

rs10816848 JPT & PEL 0.00442 0.00040

rs10816848 JPT & MXL 0.00000 0.00000

rs10816848 JPT & CHS 0.45280 0.00296

rs10816848 JPT & CDX 0.14044 0.00267

rs10816848 JPT & KHV 0.38351 0.00339

rs10816848 JPT & CHB 0.00104 0.00016

rs10816848 CEU & ACB 0.03972 0.00148

rs10816848 CEU & ASW 0.02868 0.00099

rs10816848 CEU & ESN 0.19207 0.00333

rs10816848 CEU & GWD 0.06493 0.00238

rs10816848 CEU & LWK 0.61583 0.00362

rs10816848 CEU & MSL 0.05747 0.00185

rs10816848 CEU & YRI 0.00813 0.00071

rs10816848 CEU & PUR 0.00651 0.00065

rs10816848 CEU & CLM 0.00000 0.00000

rs10816848 CEU & PEL 0.00012 0.00007

rs10816848 CEU & MXL 0.90874 0.00088

rs10816848 CEU & CHS 0.00000 0.00000

rs10816848 CEU & CDX 0.00004 0.00004

rs10816848 CEU & KHV 0.00000 0.00000

rs10816848 CEU & CHB 0.00009 0.00004

rs10816848 CEU & JPT 0.00000 0.00000

rs10816848 FIN & ACB 0.29938 0.00343

rs10816848 FIN & ASW 0.62983 0.00256

rs10816848 FIN & ESN 0.10326 0.00269

rs10816848 FIN & GWD 0.19266 0.00357

rs10816848 FIN & LWK 0.01413 0.00098

rs10816848 FIN & MSL 0.28365 0.00388

rs10816848 FIN & YRI 0.68492 0.00285

rs10816848 FIN & PUR 0.67922 0.00268

rs10816848 FIN & CLM 0.02428 0.00112

rs10816848 FIN & PEL 0.44046 0.00334

rs10816848 FIN & MXL 0.01148 0.00078

rs10816848 FIN & CHS 0.00185 0.00028

rs10816848 FIN & CDX 0.02625 0.00141

rs10816848 FIN & KHV 0.00522 0.00051

rs10816848 FIN & CHB 0.52351 0.00376

rs10816848 FIN & JPT 0.00010 0.00007

rs10816848 FIN & CEU 0.00188 0.00028

rs10816848 GBR & ACB 0.30337 0.00391

rs10816848 GBR & ASW 0.19323 0.00289

rs10816848 GBR & ESN 0.68377 0.00302

rs10816848 GBR & GWD 0.42323 0.00412

rs10816848 GBR & LWK 0.75886 0.00233

rs10816848 GBR & MSL 0.39021 0.00376

rs10816848 GBR & YRI 0.10139 0.00258

rs10816848 GBR & PUR 0.09921 0.00263

rs10816848 GBR & CLM 0.00004 0.00002

rs10816848 GBR & PEL 0.00435 0.00041

rs10816848 GBR & MXL 0.57108 0.00272

rs10816848 GBR & CHS 0.00000 0.00000

rs10816848 GBR & CDX 0.00004 0.00003

rs10816848 GBR & KHV 0.00000 0.00000

rs10816848 GBR & CHB 0.00394 0.00043

rs10816848 GBR & JPT 0.00000 0.00000

rs10816848 GBR & CEU 0.35146 0.00377

rs10816848 GBR & FIN 0.03425 0.00153

rs10816848 IBS & ACB 0.03652 0.00155

rs10816848 IBS & ASW 0.02386 0.00103

rs10816848 IBS & ESN 0.13859 0.00323

rs10816848 IBS & GWD 0.04263 0.00195

rs10816848 IBS & LWK 0.49259 0.00444

rs10816848 IBS & MSL 0.05104 0.00176

rs10816848 IBS & YRI 0.00475 0.00060

rs10816848 IBS & PUR 0.00399 0.00049

rs10816848 IBS & CLM 0.00000 0.00000

rs10816848 IBS & PEL 0.00006 0.00004

rs10816848 IBS & MXL 0.82306 0.00157

rs10816848 IBS & CHS 0.00000 0.00000

rs10816848 IBS & CDX 0.00000 0.00000

rs10816848 IBS & KHV 0.00000 0.00000

rs10816848 IBS & CHB 0.00002 0.00002

rs10816848 IBS & JPT 0.00000 0.00000

rs10816848 IBS & CEU 1.00000 0.00000

rs10816848 IBS & FIN 0.00094 0.00020

rs10816848 IBS & GBR 0.31438 0.00401

rs10816848 TSI & ACB 0.48265 0.00413

rs10816848 TSI & ASW 0.30046 0.00344

rs10816848 TSI & ESN 0.92271 0.00102

rs10816848 TSI & GWD 0.56564 0.00351

rs10816848 TSI & LWK 0.48899 0.00362

rs10816848 TSI & MSL 0.53331 0.00331

rs10816848 TSI & YRI 0.17482 0.00365

rs10816848 TSI & PUR 0.16753 0.00324

rs10816848 TSI & CLM 0.00002 0.00001

rs10816848 TSI & PEL 0.00779 0.00075

rs10816848 TSI & MXL 0.36376 0.00338

rs10816848 TSI & CHS 0.00000 0.00000

rs10816848 TSI & CDX 0.00002 0.00002

rs10816848 TSI & KHV 0.00000 0.00000

rs10816848 TSI & CHB 0.01230 0.00083

rs10816848 TSI & JPT 0.00000 0.00000

rs10816848 TSI & CEU 0.20086 0.00362

rs10816848 TSI & FIN 0.06795 0.00226

rs10816848 TSI & GBR 0.75999 0.00229

rs10816848 TSI & IBS 0.17436 0.00318

rs10816848 BEB & ACB 0.00000 0.00000

rs10816848 BEB & ASW 0.00152 0.00023

rs10816848 BEB & ESN 0.00000 0.00000

rs10816848 BEB & GWD 0.00000 0.00000

rs10816848 BEB & LWK 0.00000 0.00000

rs10816848 BEB & MSL 0.00007 0.00004

rs10816848 BEB & YRI 0.00023 0.00007

rs10816848 BEB & PUR 0.00035 0.00008

rs10816848 BEB & CLM 0.38209 0.00334

rs10816848 BEB & PEL 0.03458 0.00124

rs10816848 BEB & MXL 0.00000 0.00000

rs10816848 BEB & CHS 0.89980 0.00077

rs10816848 BEB & CDX 0.44670 0.00343

rs10816848 BEB & KHV 0.79779 0.00164

rs10816848 BEB & CHB 0.01325 0.00074

rs10816848 BEB & JPT 0.59971 0.00264

rs10816848 BEB & CEU 0.00000 0.00000

rs10816848 BEB & FIN 0.00266 0.00033

rs10816848 BEB & GBR 0.00000 0.00000

rs10816848 BEB & IBS 0.00000 0.00000

rs10816848 BEB & TSI 0.00003 0.00003

rs10816848 GIH & ACB 0.00008 0.00004

rs10816848 GIH & ASW 0.00326 0.00036

rs10816848 GIH & ESN 0.00001 0.00001

rs10816848 GIH & GWD 0.00006 0.00006

rs10816848 GIH & LWK 0.00000 0.00000

rs10816848 GIH & MSL 0.00024 0.00008

rs10816848 GIH & YRI 0.00091 0.00019

rs10816848 GIH & PUR 0.00109 0.00018

rs10816848 GIH & CLM 0.63522 0.00267

rs10816848 GIH & PEL 0.07538 0.00219

rs10816848 GIH & MXL 0.00000 0.00000

rs10816848 GIH & CHS 0.81290 0.00184

rs10816848 GIH & CDX 0.72028 0.00219

rs10816848 GIH & KHV 0.90541 0.00087

rs10816848 GIH & CHB 0.04355 0.00180

rs10816848 GIH & JPT 0.26715 0.00345

rs10816848 GIH & CEU 0.00000 0.00000

rs10816848 GIH & FIN 0.00575 0.00054

rs10816848 GIH & GBR 0.00000 0.00000

rs10816848 GIH & IBS 0.00000 0.00000

rs10816848 GIH & TSI 0.00000 0.00000

rs10816848 GIH & BEB 0.70633 0.00240

rs10816848 ITU & ACB 0.01942 0.00098

rs10816848 ITU & ASW 0.11092 0.00211

rs10816848 ITU & ESN 0.00287 0.00034

rs10816848 ITU & GWD 0.00886 0.00071

rs10816848 ITU & LWK 0.00015 0.00005

rs10816848 ITU & MSL 0.02126 0.00107

rs10816848 ITU & YRI 0.07581 0.00235

rs10816848 ITU & PUR 0.09261 0.00287

rs10816848 ITU & CLM 0.36290 0.00369

rs10816848 ITU & PEL 0.73831 0.00188

rs10816848 ITU & MXL 0.00014 0.00005

rs10816848 ITU & CHS 0.07001 0.00196

rs10816848 ITU & CDX 0.30192 0.00327

rs10816848 ITU & KHV 0.11264 0.00249

rs10816848 ITU & CHB 0.58727 0.00312

rs10816848 ITU & JPT 0.01101 0.00072

rs10816848 ITU & CEU 0.00000 0.00000

rs10816848 ITU & FIN 0.23965 0.00365

rs10816848 ITU & GBR 0.00062 0.00014

rs10816848 ITU & IBS 0.00007 0.00006

rs10816848 ITU & TSI 0.00175 0.00028

rs10816848 ITU & BEB 0.05830 0.00194

rs10816848 ITU & GIH 0.14292 0.00267

rs10816848 PJL & ACB 0.67259 0.00286

rs10816848 PJL & ASW 1.00000 0.00000

rs10816848 PJL & ESN 0.26435 0.00409

rs10816848 PJL & GWD 0.49104 0.00345

rs10816848 PJL & LWK 0.05315 0.00190

rs10816848 PJL & MSL 0.58913 0.00344

rs10816848 PJL & YRI 1.00000 0.00000

rs10816848 PJL & PUR 1.00000 0.00000

rs10816848 PJL & CLM 0.00747 0.00061

rs10816848 PJL & PEL 0.18358 0.00352

rs10816848 PJL & MXL 0.04967 0.00140

rs10816848 PJL & CHS 0.00013 0.00004

rs10816848 PJL & CDX 0.00502 0.00054

rs10816848 PJL & KHV 0.00078 0.00016

rs10816848 PJL & CHB 0.23833 0.00351

rs10816848 PJL & JPT 0.00001 0.00000

rs10816848 PJL & CEU 0.01102 0.00086

rs10816848 PJL & FIN 0.59871 0.00344

rs10816848 PJL & GBR 0.11671 0.00294

rs10816848 PJL & IBS 0.00697 0.00066

rs10816848 PJL & TSI 0.18882 0.00324

rs10816848 PJL & BEB 0.00030 0.00008

rs10816848 PJL & GIH 0.00096 0.00018

rs10816848 PJL & ITU 0.08749 0.00225

rs10816848 STU & ACB 0.00136 0.00025

rs10816848 STU & ASW 0.01769 0.00094

rs10816848 STU & ESN 0.00010 0.00006

rs10816848 STU & GWD 0.00026 0.00009

rs10816848 STU & LWK 0.00000 0.00000

rs10816848 STU & MSL 0.00149 0.00025

rs10816848 STU & YRI 0.00511 0.00049

rs10816848 STU & PUR 0.00644 0.00053

rs10816848 STU & CLM 1.00000 0.00000

rs10816848 STU & PEL 0.19871 0.00336

rs10816848 STU & MXL 0.00000 0.00000

rs10816848 STU & CHS 0.41525 0.00333

rs10816848 STU & CDX 1.00000 0.00000

rs10816848 STU & KHV 0.55943 0.00297

rs10816848 STU & CHB 0.12572 0.00281

rs10816848 STU & JPT 0.11579 0.00241

rs10816848 STU & CEU 0.00000 0.00000

rs10816848 STU & FIN 0.03020 0.00148

rs10816848 STU & GBR 0.00000 0.00000

rs10816848 STU & IBS 0.00000 0.00000

rs10816848 STU & TSI 0.00010 0.00005

rs10816848 STU & BEB 0.38552 0.00318

rs10816848 STU & GIH 0.63906 0.00262

rs10816848 STU & ITU 0.36697 0.00337

rs10816848 STU & PJL 0.00591 0.00049

Locus: rs79095029

=================================

Pop Alleles:

-----------------------------------------------------------

2 3 Total

ACB 179 13 192

ASW 116 6 122

ESN 174 24 198

GWD 204 22 226

LWK 180 18 198

MSL 146 24 170

YRI 197 19 216

PUR 195 13 208

CLM 179 9 188

PEL 170 0 170

MXL 125 3 128

CHS 201 9 210

CDX 177 9 186

KHV 183 15 198

CHB 199 7 206

JPT 206 2 208

CEU 179 19 198

FIN 193 5 198

GBR 170 12 182

IBS 206 8 214

TSI 206 8 214

BEB 168 4 172

GIH 204 2 206

ITU 199 5 204

PJL 182 10 192

STU 201 3 204

Total: 4739269 5008

Locus Population pair P-Value S.E.

----------- --------------------- ------- -------

rs79095029 ASW & ACB 0.62851 0.00135

rs79095029 ESN & ACB 0.08605 0.00131

rs79095029 ESN & ASW 0.03172 0.00076

rs79095029 GWD & ACB 0.29208 0.00243

rs79095029 GWD & ASW 0.14765 0.00154

rs79095029 GWD & ESN 0.43859 0.00272

rs79095029 LWK & ACB 0.45519 0.00220

rs79095029 LWK & ASW 0.19381 0.00185

rs79095029 LWK & ESN 0.41794 0.00239

rs79095029 LWK & GWD 0.86983 0.00075

rs79095029 MSL & ACB 0.02413 0.00079

rs79095029 MSL & ASW 0.01104 0.00049

rs79095029 MSL & ESN 0.64374 0.00191

rs79095029 MSL & GWD 0.20560 0.00250

rs79095029 MSL & LWK 0.14365 0.00191

rs79095029 YRI & ACB 0.46668 0.00225

rs79095029 YRI & ASW 0.20493 0.00195

rs79095029 YRI & ESN 0.33149 0.00258

rs79095029 YRI & GWD 0.74643 0.00170

rs79095029 YRI & LWK 1.00000 0.00000

rs79095029 YRI & MSL 0.10527 0.00182

rs79095029 PUR & ACB 0.84282 0.00086

rs79095029 PUR & ASW 0.64027 0.00145

rs79095029 PUR & ESN 0.05809 0.00131

rs79095029 PUR & GWD 0.21948 0.00220

rs79095029 PUR & LWK 0.35256 0.00241

rs79095029 PUR & MSL 0.01337 0.00056

rs79095029 PUR & YRI 0.36323 0.00211

rs79095029 CLM & ACB 0.51367 0.00192

rs79095029 CLM & ASW 1.00000 0.00000

rs79095029 CLM & ESN 0.01027 0.00049

rs79095029 CLM & GWD 0.06151 0.00118

rs79095029 CLM & LWK 0.11136 0.00137

rs79095029 CLM & MSL 0.00318 0.00027

rs79095029 CLM & YRI 0.12145 0.00161

rs79095029 CLM & PUR 0.66128 0.00136

rs79095029 PEL & ACB 0.00027 0.00004

rs79095029 PEL & ASW 0.00499 0.00020

rs79095029 PEL & ESN 0.00000 0.00000

rs79095029 PEL & GWD 0.00000 0.00000

rs79095029 PEL & LWK 0.00002 0.00001

rs79095029 PEL & MSL 0.00000 0.00000

rs79095029 PEL & YRI 0.00001 0.00001

rs79095029 PEL & PUR 0.00042 0.00005

rs79095029 PEL & CLM 0.00390 0.00016

rs79095029 MXL & ACB 0.07679 0.00101

rs79095029 MXL & ASW 0.32443 0.00144

rs79095029 MXL & ESN 0.00152 0.00013

rs79095029 MXL & GWD 0.00819 0.00034

rs79095029 MXL & LWK 0.01874 0.00052

rs79095029 MXL & MSL 0.00035 0.00006

rs79095029 MXL & YRI 0.02096 0.00056

rs79095029 MXL & PUR 0.11882 0.00130

rs79095029 MXL & CLM 0.37234 0.00158

rs79095029 MXL & PEL 0.07876 0.00071

rs79095029 CHS & ACB 0.37843 0.00184

rs79095029 CHS & ASW 1.00000 0.00000

rs79095029 CHS & ESN 0.00552 0.00034

rs79095029 CHS & GWD 0.03893 0.00088

rs79095029 CHS & LWK 0.07054 0.00115

rs79095029 CHS & MSL 0.00086 0.00011

rs79095029 CHS & YRI 0.07699 0.00136

rs79095029 CHS & PUR 0.39164 0.00205

rs79095029 CHS & CLM 1.00000 0.00000

rs79095029 CHS & PEL 0.00510 0.00021

rs79095029 CHS & MXL 0.38869 0.00144

rs79095029 CDX & ACB 0.50932 0.00153

rs79095029 CDX & ASW 1.00000 0.00000

rs79095029 CDX & ESN 0.01199 0.00052

rs79095029 CDX & GWD 0.06320 0.00121

rs79095029 CDX & LWK 0.11304 0.00144

rs79095029 CDX & MSL 0.00273 0.00019

rs79095029 CDX & YRI 0.16563 0.00182

rs79095029 CDX & PUR 0.66271 0.00140

rs79095029 CDX & CLM 1.00000 0.00000

rs79095029 CDX & PEL 0.00401 0.00016

rs79095029 CDX & MXL 0.37057 0.00160

rs79095029 CDX & CHS 0.81276 0.00073

rs79095029 KHV & ACB 0.84426 0.00089

rs79095029 KHV & ASW 0.36931 0.00200

rs79095029 KHV & ESN 0.17859 0.00211

rs79095029 KHV & GWD 0.49427 0.00232

rs79095029 KHV & LWK 0.71787 0.00146

rs79095029 KHV & MSL 0.06016 0.00144

rs79095029 KHV & YRI 0.72168 0.00152

rs79095029 KHV & PUR 0.69745 0.00143

rs79095029 KHV & CLM 0.29407 0.00175

rs79095029 KHV & PEL 0.00007 0.00002

rs79095029 KHV & MXL 0.04821 0.00075

rs79095029 KHV & CHS 0.20331 0.00167

rs79095029 KHV & CDX 0.29930 0.00191

rs79095029 CHB & ACB 0.17140 0.00161

rs79095029 CHB & ASW 0.56111 0.00146

rs79095029 CHB & ESN 0.00118 0.00012

rs79095029 CHB & GWD 0.01027 0.00042

rs79095029 CHB & LWK 0.02188 0.00071

rs79095029 CHB & MSL 0.00033 0.00006

rs79095029 CHB & YRI 0.02508 0.00065

rs79095029 CHB & PUR 0.25308 0.00176

rs79095029 CHB & CLM 0.61153 0.00132

rs79095029 CHB & PEL 0.01820 0.00038

rs79095029 CHB & MXL 0.74766 0.00072

rs79095029 CHB & CHS 0.79917 0.00085

rs79095029 CHB & CDX 0.60864 0.00143

rs79095029 CHB & KHV 0.08199 0.00116

rs79095029 JPT & ACB 0.00269 0.00019

rs79095029 JPT & ASW 0.05519 0.00063

rs79095029 JPT & ESN 0.00000 0.00000

rs79095029 JPT & GWD 0.00002 0.00001

rs79095029 JPT & LWK 0.00013 0.00003

rs79095029 JPT & MSL 0.00000 0.00000

rs79095029 JPT & YRI 0.00015 0.00004

rs79095029 JPT & PUR 0.00662 0.00025

rs79095029 JPT & CLM 0.02961 0.00058

rs79095029 JPT & PEL 0.50310 0.00061

rs79095029 JPT & MXL 0.37366 0.00115

rs79095029 JPT & CHS 0.06230 0.00074

rs79095029 JPT & CDX 0.02937 0.00059

rs79095029 JPT & KHV 0.00088 0.00008

rs79095029 JPT & CHB 0.10478 0.00113

rs79095029 CEU & ACB 0.35992 0.00246

rs79095029 CEU & ASW 0.13802 0.00137

rs79095029 CEU & ESN 0.52154 0.00237

rs79095029 CEU & GWD 1.00000 0.00000

rs79095029 CEU & LWK 1.00000 0.00000

rs79095029 CEU & MSL 0.19453 0.00214

rs79095029 CEU & YRI 0.86456 0.00091

rs79095029 CEU & PUR 0.27099 0.00220

rs79095029 CEU & CLM 0.07624 0.00129

rs79095029 CEU & PEL 0.00000 0.00000

rs79095029 CEU & MXL 0.01224 0.00046

rs79095029 CEU & CHS 0.04934 0.00104

rs79095029 CEU & CDX 0.08057 0.00152

rs79095029 CEU & KHV 0.59012 0.00193

rs79095029 CEU & CHB 0.01463 0.00058

rs79095029 CEU & JPT 0.00006 0.00003

rs79095029 FIN & ACB 0.05437 0.00096

rs79095029 FIN & ASW 0.34463 0.00152

rs79095029 FIN & ESN 0.00034 0.00008

rs79095029 FIN & GWD 0.00249 0.00019

rs79095029 FIN & LWK 0.00833 0.00038

rs79095029 FIN & MSL 0.00005 0.00002

rs79095029 FIN & YRI 0.00592 0.00029

rs79095029 FIN & PUR 0.09090 0.00116

rs79095029 FIN & CLM 0.28171 0.00157

rs79095029 FIN & PEL 0.06367 0.00059

rs79095029 FIN & MXL 1.00000 0.00000

rs79095029 FIN & CHS 0.41730 0.00160

rs79095029 FIN & CDX 0.27915 0.00163

rs79095029 FIN & KHV 0.03632 0.00085

rs79095029 FIN & CHB 0.77175 0.00073

rs79095029 FIN & JPT 0.27339 0.00124

rs79095029 FIN & CEU 0.00481 0.00026

rs79095029 GBR & ACB 1.00000 0.00000

rs79095029 GBR & ASW 0.62754 0.00137

rs79095029 GBR & ESN 0.07650 0.00152

rs79095029 GBR & GWD 0.28223 0.00202

rs79095029 GBR & LWK 0.44802 0.00224

rs79095029 GBR & MSL 0.02175 0.00072

rs79095029 GBR & YRI 0.45798 0.00204

rs79095029 GBR & PUR 1.00000 0.00000

rs79095029 GBR & CLM 0.50456 0.00191

rs79095029 GBR & PEL 0.00042 0.00005

rs79095029 GBR & MXL 0.10977 0.00102

rs79095029 GBR & CHS 0.37215 0.00191

rs79095029 GBR & CDX 0.50879 0.00183

rs79095029 GBR & KHV 0.84270 0.00076

rs79095029 GBR & CHB 0.16337 0.00138

rs79095029 GBR & JPT 0.00445 0.00019

rs79095029 GBR & CEU 0.34692 0.00218

rs79095029 GBR & FIN 0.08042 0.00122

rs79095029 IBS & ACB 0.18400 0.00184

rs79095029 IBS & ASW 0.77704 0.00070

rs79095029 IBS & ESN 0.00175 0.00017

rs79095029 IBS & GWD 0.01317 0.00049

rs79095029 IBS & LWK 0.04112 0.00088

rs79095029 IBS & MSL 0.00028 0.00006

rs79095029 IBS & YRI 0.04480 0.00102

rs79095029 IBS & PUR 0.26695 0.00208

rs79095029 IBS & CLM 0.62811 0.00131

rs79095029 IBS & PEL 0.01048 0.00028

rs79095029 IBS & MXL 0.54409 0.00131

rs79095029 IBS & CHS 0.80800 0.00079

rs79095029 IBS & CDX 0.62684 0.00146

rs79095029 IBS & KHV 0.13193 0.00140

rs79095029 IBS & CHB 1.00000 0.00000

rs79095029 IBS & JPT 0.10668 0.00105

rs79095029 IBS & CEU 0.02684 0.00082

rs79095029 IBS & FIN 0.57790 0.00130

rs79095029 IBS & GBR 0.25173 0.00136

rs79095029 TSI & ACB 0.18518 0.00172

rs79095029 TSI & ASW 0.77738 0.00071

rs79095029 TSI & ESN 0.00170 0.00017

rs79095029 TSI & GWD 0.01303 0.00051

rs79095029 TSI & LWK 0.03995 0.00087

rs79095029 TSI & MSL 0.00037 0.00007

rs79095029 TSI & YRI 0.04546 0.00090

rs79095029 TSI & PUR 0.26774 0.00188

rs79095029 TSI & CLM 0.62762 0.00141

rs79095029 TSI & PEL 0.01012 0.00025

rs79095029 TSI & MXL 0.54615 0.00131

rs79095029 TSI & CHS 0.81098 0.00087

rs79095029 TSI & CDX 0.62943 0.00168

rs79095029 TSI & KHV 0.13066 0.00143

rs79095029 TSI & CHB 1.00000 0.00000

rs79095029 TSI & JPT 0.10535 0.00095

rs79095029 TSI & CEU 0.02684 0.00065

rs79095029 TSI & FIN 0.57939 0.00141

rs79095029 TSI & GBR 0.24680 0.00191

rs79095029 TSI & IBS 1.00000 0.00000

rs79095029 BEB & ACB 0.04912 0.00079

rs79095029 BEB & ASW 0.32580 0.00133

rs79095029 BEB & ESN 0.00034 0.00007

rs79095029 BEB & GWD 0.00311 0.00019

rs79095029 BEB & LWK 0.00701 0.00030

rs79095029 BEB & MSL 0.00008 0.00003

rs79095029 BEB & YRI 0.00866 0.00039

rs79095029 BEB & PUR 0.08122 0.00107

rs79095029 BEB & CLM 0.26482 0.00150

rs79095029 BEB & PEL 0.12159 0.00076

rs79095029 BEB & MXL 1.00000 0.00000

rs79095029 BEB & CHS 0.39706 0.00150

rs79095029 BEB & CDX 0.26319 0.00165

rs79095029 BEB & KHV 0.03116 0.00069

rs79095029 BEB & CHB 0.56168 0.00097

rs79095029 BEB & JPT 0.41737 0.00125

rs79095029 BEB & CEU 0.00376 0.00024

rs79095029 BEB & FIN 1.00000 0.00000

rs79095029 BEB & GBR 0.07266 0.00107

rs79095029 BEB & IBS 0.56113 0.00142

rs79095029 BEB & TSI 0.56065 0.00124

rs79095029 GIH & ACB 0.00269 0.00015

rs79095029 GIH & ASW 0.05654 0.00065

rs79095029 GIH & ESN 0.00000 0.00000

rs79095029 GIH & GWD 0.00005 0.00002

rs79095029 GIH & LWK 0.00013 0.00003

rs79095029 GIH & MSL 0.00000 0.00000

rs79095029 GIH & YRI 0.00020 0.00004

rs79095029 GIH & PUR 0.00642 0.00029

rs79095029 GIH & CLM 0.03002 0.00062

rs79095029 GIH & PEL 0.50297 0.00071

rs79095029 GIH & MXL 0.37609 0.00122

rs79095029 GIH & CHS 0.06218 0.00071

rs79095029 GIH & CDX 0.02837 0.00046

rs79095029 GIH & KHV 0.00074 0.00008

rs79095029 GIH & CHB 0.17682 0.00109

rs79095029 GIH & JPT 1.00000 0.00000

rs79095029 GIH & CEU 0.00006 0.00002

rs79095029 GIH & FIN 0.27480 0.00116

rs79095029 GIH & GBR 0.00429 0.00020

rs79095029 GIH & IBS 0.10702 0.00091

rs79095029 GIH & TSI 0.10369 0.00105

rs79095029 GIH & BEB 0.41838 0.00126

rs79095029 ITU & ACB 0.05115 0.00102

rs79095029 ITU & ASW 0.34262 0.00132

rs79095029 ITU & ESN 0.00014 0.00004

rs79095029 ITU & GWD 0.00229 0.00019

rs79095029 ITU & LWK 0.00482 0.00027

rs79095029 ITU & MSL 0.00006 0.00003

rs79095029 ITU & YRI 0.00562 0.00031

rs79095029 ITU & PUR 0.09051 0.00127

rs79095029 ITU & CLM 0.28066 0.00149

rs79095029 ITU & PEL 0.06580 0.00063

rs79095029 ITU & MXL 1.00000 0.00000

rs79095029 ITU & CHS 0.41785 0.00182

rs79095029 ITU & CDX 0.27734 0.00142

rs79095029 ITU & KHV 0.02227 0.00061

rs79095029 ITU & CHB 0.77070 0.00076

rs79095029 ITU & JPT 0.28076 0.00142

rs79095029 ITU & CEU 0.00272 0.00019

rs79095029 ITU & FIN 1.00000 0.00000

rs79095029 ITU & GBR 0.07893 0.00106

rs79095029 ITU & IBS 0.57675 0.00118

rs79095029 ITU & TSI 0.57407 0.00134

rs79095029 ITU & BEB 1.00000 0.00000

rs79095029 ITU & GIH 0.28257 0.00120

rs79095029 PJL & ACB 0.66754 0.00140

rs79095029 PJL & ASW 1.00000 0.00000

rs79095029 PJL & ESN 0.01876 0.00066

rs79095029 PJL & GWD 0.09994 0.00146

rs79095029 PJL & LWK 0.16710 0.00173

rs79095029 PJL & MSL 0.00554 0.00029

rs79095029 PJL & YRI 0.17868 0.00173

rs79095029 PJL & PUR 0.67337 0.00145

rs79095029 PJL & CLM 1.00000 0.00000

rs79095029 PJL & PEL 0.00208 0.00011

rs79095029 PJL & MXL 0.25848 0.00152

rs79095029 PJL & CHS 0.81386 0.00076

rs79095029 PJL & CDX 1.00000 0.00000

rs79095029 PJL & KHV 0.41111 0.00224

rs79095029 PJL & CHB 0.45988 0.00157

rs79095029 PJL & JPT 0.01722 0.00041

rs79095029 PJL & CEU 0.12384 0.00153

rs79095029 PJL & FIN 0.19613 0.00137

rs79095029 PJL & GBR 0.66407 0.00162

rs79095029 PJL & IBS 0.63088 0.00155

rs79095029 PJL & TSI 0.63194 0.00131

rs79095029 PJL & BEB 0.17992 0.00157

rs79095029 PJL & GIH 0.01766 0.00048

rs79095029 PJL & ITU 0.18809 0.00145

rs79095029 STU & ACB 0.00823 0.00028

rs79095029 STU & ASW 0.16125 0.00104

rs79095029 STU & ESN 0.00004 0.00002

rs79095029 STU & GWD 0.00019 0.00004

rs79095029 STU & LWK 0.00059 0.00008

rs79095029 STU & MSL 0.00000 0.00000

rs79095029 STU & YRI 0.00061 0.00008

rs79095029 STU & PUR 0.01885 0.00045

rs79095029 STU & CLM 0.07742 0.00092

rs79095029 STU & PEL 0.25419 0.00083

rs79095029 STU & MXL 0.67905 0.00081

rs79095029 STU & CHS 0.13921 0.00130

rs79095029 STU & CDX 0.07673 0.00097

rs79095029 STU & KHV 0.00323 0.00017

rs79095029 STU & CHB 0.33938 0.00136

rs79095029 STU & JPT 0.68356 0.00084

rs79095029 STU & CEU 0.00022 0.00004

rs79095029 STU & FIN 0.49718 0.00135

rs79095029 STU & GBR 0.01472 0.00038

rs79095029 STU & IBS 0.22305 0.00154

rs79095029 STU & TSI 0.22362 0.00121

rs79095029 STU & BEB 0.70708 0.00077

rs79095029 STU & GIH 0.68348 0.00083

rs79095029 STU & ITU 0.72535 0.00077

rs79095029 STU & PJL 0.04827 0.00079

Locus: rs13393316

=================================

Pop Alleles:

-----------------------------------------------------------

1 3 Total

ACB 168 24 192

ASW 108 14 122

ESN 173 25 198

GWD 201 25 226

LWK 184 14 198

MSL 158 12 170

YRI 197 19 216

PUR 153 55 208

CLM 120 68 188

PEL 122 48 170

MXL 106 22 128

CHS 210 0 210

CDX 186 0 186

KHV 198 0 198

CHB 206 0 206

JPT 208 0 208

CEU 171 27 198

FIN 173 25 198

GBR 160 22 182

IBS 177 37 214

TSI 175 39 214

BEB 166 6 172

GIH 195 11 206

ITU 200 4 204

PJL 185 7 192

STU 195 9 204

Total: 4495513 5008

Locus Population pair P-Value S.E.

----------- --------------------- ------- -------

rs13393316 ASW & ACB 0.86205 0.00084

rs13393316 ESN & ACB 1.00000 0.00000

rs13393316 ESN & ASW 0.86090 0.00076

rs13393316 GWD & ACB 0.75948 0.00158

rs13393316 GWD & ASW 1.00000 0.00000

rs13393316 GWD & ESN 0.65239 0.00196

rs13393316 LWK & ACB 0.08664 0.00159

rs13393316 LWK & ASW 0.22060 0.00176

rs13393316 LWK & ESN 0.09209 0.00168

rs13393316 LWK & GWD 0.17880 0.00209

rs13393316 MSL & ACB 0.11576 0.00174

rs13393316 MSL & ASW 0.21408 0.00177

rs13393316 MSL & ESN 0.08596 0.00163

rs13393316 MSL & GWD 0.22421 0.00187

rs13393316 MSL & LWK 1.00000 0.00000

rs13393316 YRI & ACB 0.25836 0.00237

rs13393316 YRI & ASW 0.44715 0.00225

rs13393316 YRI & ESN 0.26379 0.00280

rs13393316 YRI & GWD 0.43420 0.00242

rs13393316 YRI & LWK 0.58931 0.00204

rs13393316 YRI & MSL 0.58003 0.00219

rs13393316 PUR & ACB 0.00059 0.00012

rs13393316 PUR & ASW 0.00140 0.00019

rs13393316 PUR & ESN 0.00051 0.00010

rs13393316 PUR & GWD 0.00005 0.00004

rs13393316 PUR & LWK 0.00000 0.00000

rs13393316 PUR & MSL 0.00000 0.00000

rs13393316 PUR & YRI 0.00000 0.00000

rs13393316 CLM & ACB 0.00000 0.00000

rs13393316 CLM & ASW 0.00000 0.00000

rs13393316 CLM & ESN 0.00000 0.00000

rs13393316 CLM & GWD 0.00000 0.00000

rs13393316 CLM & LWK 0.00000 0.00000

rs13393316 CLM & MSL 0.00000 0.00000

rs13393316 CLM & YRI 0.00000 0.00000

rs13393316 CLM & PUR 0.04154 0.00170

rs13393316 PEL & ACB 0.00020 0.00005

rs13393316 PEL & ASW 0.00042 0.00008

rs13393316 PEL & ESN 0.00024 0.00009

rs13393316 PEL & GWD 0.00003 0.00002

rs13393316 PEL & LWK 0.00000 0.00000

rs13393316 PEL & MSL 0.00000 0.00000

rs13393316 PEL & YRI 0.00000 0.00000

rs13393316 PEL & PUR 0.73093 0.00227

rs13393316 PEL & CLM 0.11046 0.00276

rs13393316 MXL & ACB 0.25763 0.00252

rs13393316 MXL & ASW 0.21088 0.00201

rs13393316 MXL & ESN 0.26358 0.00256

rs13393316 MXL & GWD 0.14125 0.00206

rs13393316 MXL & LWK 0.00602 0.00034

rs13393316 MXL & MSL 0.00950 0.00044

rs13393316 MXL & YRI 0.02635 0.00094

rs13393316 MXL & PUR 0.05853 0.00140

rs13393316 MXL & CLM 0.00018 0.00007

rs13393316 MXL & PEL 0.02782 0.00099

rs13393316 CHS & ACB 0.00000 0.00000

rs13393316 CHS & ASW 0.00000 0.00000

rs13393316 CHS & ESN 0.00000 0.00000

rs13393316 CHS & GWD 0.00000 0.00000

rs13393316 CHS & LWK 0.00002 0.00001

rs13393316 CHS & MSL 0.00008 0.00003

rs13393316 CHS & YRI 0.00000 0.00000

rs13393316 CHS & PUR 0.00000 0.00000

rs13393316 CHS & CLM 0.00000 0.00000

rs13393316 CHS & PEL 0.00000 0.00000

rs13393316 CHS & MXL 0.00000 0.00000

rs13393316 CDX & ACB 0.00000 0.00000

rs13393316 CDX & ASW 0.00001 0.00001

rs13393316 CDX & ESN 0.00000 0.00000

rs13393316 CDX & GWD 0.00000 0.00000

rs13393316 CDX & LWK 0.00009 0.00002

rs13393316 CDX & MSL 0.00010 0.00002

rs13393316 CDX & YRI 0.00001 0.00001

rs13393316 CDX & PUR 0.00000 0.00000

rs13393316 CDX & CLM 0.00000 0.00000

rs13393316 CDX & PEL 0.00000 0.00000

rs13393316 CDX & MXL 0.00000 0.00000

rs13393316 CDX & CHS No table

rs13393316 KHV & ACB 0.00000 0.00000

rs13393316 KHV & ASW 0.00000 0.00000

rs13393316 KHV & ESN 0.00000 0.00000

rs13393316 KHV & GWD 0.00000 0.00000

rs13393316 KHV & LWK 0.00010 0.00002

rs13393316 KHV & MSL 0.00005 0.00002

rs13393316 KHV & YRI 0.00001 0.00001

rs13393316 KHV & PUR 0.00000 0.00000

rs13393316 KHV & CLM 0.00000 0.00000

rs13393316 KHV & PEL 0.00000 0.00000

rs13393316 KHV & MXL 0.00000 0.00000

rs13393316 KHV & CHS No table

rs13393316 KHV & CDX No table

rs13393316 CHB & ACB 0.00000 0.00000

rs13393316 CHB & ASW 0.00000 0.00000

rs13393316 CHB & ESN 0.00000 0.00000

rs13393316 CHB & GWD 0.00000 0.00000

rs13393316 CHB & LWK 0.00006 0.00002

rs13393316 CHB & MSL 0.00003 0.00001

rs13393316 CHB & YRI 0.00001 0.00001

rs13393316 CHB & PUR 0.00000 0.00000

rs13393316 CHB & CLM 0.00000 0.00000

rs13393316 CHB & PEL 0.00000 0.00000

rs13393316 CHB & MXL 0.00000 0.00000

rs13393316 CHB & CHS No table

rs13393316 CHB & CDX No table

rs13393316 CHB & KHV No table

rs13393316 JPT & ACB 0.00000 0.00000

rs13393316 JPT & ASW 0.00000 0.00000

rs13393316 JPT & ESN 0.00000 0.00000

rs13393316 JPT & GWD 0.00000 0.00000

rs13393316 JPT & LWK 0.00003 0.00001

rs13393316 JPT & MSL 0.00007 0.00002

rs13393316 JPT & YRI 0.00000 0.00000

rs13393316 JPT & PUR 0.00000 0.00000

rs13393316 JPT & CLM 0.00000 0.00000

rs13393316 JPT & PEL 0.00000 0.00000

rs13393316 JPT & MXL 0.00000 0.00000

rs13393316 JPT & CHS No table

rs13393316 JPT & CDX No table

rs13393316 JPT & KHV No table

rs13393316 JPT & CHB No table

rs13393316 CEU & ACB 0.76451 0.00160

rs13393316 CEU & ASW 0.61016 0.00196

rs13393316 CEU & ESN 0.88181 0.00073

rs13393316 CEU & GWD 0.46020 0.00298

rs13393316 CEU & LWK 0.04619 0.00108

rs13393316 CEU & MSL 0.04335 0.00112

rs13393316 CEU & YRI 0.15741 0.00214

rs13393316 CEU & PUR 0.00134 0.00019

rs13393316 CEU & CLM 0.00000 0.00000

rs13393316 CEU & PEL 0.00078 0.00014

rs13393316 CEU & MXL 0.42708 0.00271

rs13393316 CEU & CHS 0.00000 0.00000

rs13393316 CEU & CDX 0.00000 0.00000

rs13393316 CEU & KHV 0.00000 0.00000

rs13393316 CEU & CHB 0.00000 0.00000

rs13393316 CEU & JPT 0.00000 0.00000

rs13393316 FIN & ACB 1.00000 0.00000

rs13393316 FIN & ASW 0.86031 0.00079

rs13393316 FIN & ESN 1.00000 0.00000

rs13393316 FIN & GWD 0.65425 0.00210

rs13393316 FIN & LWK 0.09150 0.00158

rs13393316 FIN & MSL 0.08338 0.00160

rs13393316 FIN & YRI 0.26567 0.00244

rs13393316 FIN & PUR 0.00049 0.00010

rs13393316 FIN & CLM 0.00000 0.00000

rs13393316 FIN & PEL 0.00032 0.00011

rs13393316 FIN & MXL 0.26164 0.00234

rs13393316 FIN & CHS 0.00000 0.00000

rs13393316 FIN & CDX 0.00000 0.00000

rs13393316 FIN & KHV 0.00000 0.00000

rs13393316 FIN & CHB 0.00000 0.00000

rs13393316 FIN & JPT 0.00000 0.00000

rs13393316 FIN & CEU 0.88228 0.00083

rs13393316 GBR & ACB 1.00000 0.00000

rs13393316 GBR & ASW 1.00000 0.00000

rs13393316 GBR & ESN 0.87842 0.00082

rs13393316 GBR & GWD 0.75702 0.00172

rs13393316 GBR & LWK 0.11625 0.00153

rs13393316 GBR & MSL 0.14584 0.00187

rs13393316 GBR & YRI 0.32306 0.00246

rs13393316 GBR & PUR 0.00020 0.00005

rs13393316 GBR & CLM 0.00000 0.00000

rs13393316 GBR & PEL 0.00020 0.00007

rs13393316 GBR & MXL 0.25011 0.00239

rs13393316 GBR & CHS 0.00000 0.00000

rs13393316 GBR & CDX 0.00000 0.00000

rs13393316 GBR & KHV 0.00000 0.00000

rs13393316 GBR & CHB 0.00000 0.00000

rs13393316 GBR & JPT 0.00000 0.00000

rs13393316 GBR & CEU 0.75911 0.00168

rs13393316 GBR & FIN 0.87896 0.00083

rs13393316 IBS & ACB 0.21078 0.00276

rs13393316 IBS & ASW 0.15951 0.00223

rs13393316 IBS & ESN 0.21613 0.00236

rs13393316 IBS & GWD 0.07266 0.00181

rs13393316 IBS & LWK 0.00150 0.00017

rs13393316 IBS & MSL 0.00313 0.00033

rs13393316 IBS & YRI 0.01032 0.00058

rs13393316 IBS & PUR 0.02650 0.00115

rs13393316 IBS & CLM 0.00000 0.00000

rs13393316 IBS & PEL 0.01433 0.00095

rs13393316 IBS & MXL 1.00000 0.00000

rs13393316 IBS & CHS 0.00000 0.00000

rs13393316 IBS & CDX 0.00000 0.00000

rs13393316 IBS & KHV 0.00000 0.00000

rs13393316 IBS & CHB 0.00000 0.00000

rs13393316 IBS & JPT 0.00000 0.00000

rs13393316 IBS & CEU 0.34082 0.00289

rs13393316 IBS & FIN 0.21727 0.00297

rs13393316 IBS & GBR 0.15991 0.00240

rs13393316 TSI & ACB 0.13307 0.00234

rs13393316 TSI & ASW 0.12067 0.00205

rs13393316 TSI & ESN 0.13990 0.00219

rs13393316 TSI & GWD 0.04236 0.00127

rs13393316 TSI & LWK 0.00057 0.00012

rs13393316 TSI & MSL 0.00120 0.00013

rs13393316 TSI & YRI 0.00499 0.00036

rs13393316 TSI & PUR 0.04479 0.00145

rs13393316 TSI & CLM 0.00012 0.00007

rs13393316 TSI & PEL 0.02591 0.00111

rs13393316 TSI & MXL 0.88592 0.00085

rs13393316 TSI & CHS 0.00000 0.00000

rs13393316 TSI & CDX 0.00000 0.00000

rs13393316 TSI & KHV 0.00000 0.00000

rs13393316 TSI & CHB 0.00000 0.00000

rs13393316 TSI & JPT 0.00000 0.00000

rs13393316 TSI & CEU 0.23057 0.00300

rs13393316 TSI & FIN 0.13237 0.00237

rs13393316 TSI & GBR 0.09814 0.00200

rs13393316 TSI & IBS 0.89824 0.00084

rs13393316 BEB & ACB 0.00164 0.00015

rs13393316 BEB & ASW 0.00846 0.00034

rs13393316 BEB & ESN 0.00132 0.00014

rs13393316 BEB & GWD 0.00444 0.00028

rs13393316 BEB & LWK 0.16999 0.00142

rs13393316 BEB & MSL 0.15276 0.00130

rs13393316 BEB & YRI 0.03754 0.00085

rs13393316 BEB & PUR 0.00000 0.00000

rs13393316 BEB & CLM 0.00000 0.00000

rs13393316 BEB & PEL 0.00000 0.00000

rs13393316 BEB & MXL 0.00007 0.00003

rs13393316 BEB & CHS 0.00784 0.00025

rs13393316 BEB & CDX 0.01176 0.00029

rs13393316 BEB & KHV 0.00974 0.00027

rs13393316 BEB & CHB 0.00832 0.00025

rs13393316 BEB & JPT 0.00832 0.00022

rs13393316 BEB & CEU 0.00079 0.00011

rs13393316 BEB & FIN 0.00154 0.00015

rs13393316 BEB & GBR 0.00282 0.00021

rs13393316 BEB & IBS 0.00000 0.00000

rs13393316 BEB & TSI 0.00001 0.00001

rs13393316 GIH & ACB 0.01339 0.00056

rs13393316 GIH & ASW 0.05318 0.00098

rs13393316 GIH & ESN 0.01389 0.00056

rs13393316 GIH & GWD 0.03792 0.00109

rs13393316 GIH & LWK 0.53933 0.00194

rs13393316 GIH & MSL 0.52261 0.00170

rs13393316 GIH & YRI 0.18941 0.00183

rs13393316 GIH & PUR 0.00000 0.00000

rs13393316 GIH & CLM 0.00000 0.00000

rs13393316 GIH & PEL 0.00000 0.00000

rs13393316 GIH & MXL 0.00124 0.00013

rs13393316 GIH & CHS 0.00038 0.00005

rs13393316 GIH & CDX 0.00112 0.00010

rs13393316 GIH & KHV 0.00079 0.00007

rs13393316 GIH & CHB 0.00085 0.00008

rs13393316 GIH & JPT 0.00034 0.00005

rs13393316 GIH & CEU 0.00614 0.00037

rs13393316 GIH & FIN 0.01338 0.00054

rs13393316 GIH & GBR 0.02585 0.00074

rs13393316 GIH & IBS 0.00014 0.00005

rs13393316 GIH & TSI 0.00001 0.00001

rs13393316 GIH & BEB 0.46306 0.00148

rs13393316 ITU & ACB 0.00005 0.00002

rs13393316 ITU & ASW 0.00060 0.00009

rs13393316 ITU & ESN 0.00004 0.00002

rs13393316 ITU & GWD 0.00020 0.00004

rs13393316 ITU & LWK 0.01578 0.00050

rs13393316 ITU & MSL 0.02007 0.00053

rs13393316 ITU & YRI 0.00220 0.00018

rs13393316 ITU & PUR 0.00000 0.00000

rs13393316 ITU & CLM 0.00000 0.00000

rs13393316 ITU & PEL 0.00000 0.00000

rs13393316 ITU & MXL 0.00000 0.00000

rs13393316 ITU & CHS 0.05854 0.00060

rs13393316 ITU & CDX 0.12615 0.00072

rs13393316 ITU & KHV 0.12412 0.00074

rs13393316 ITU & CHB 0.05967 0.00065

rs13393316 ITU & JPT 0.05821 0.00061

rs13393316 ITU & CEU 0.00000 0.00000

rs13393316 ITU & FIN 0.00004 0.00003

rs13393316 ITU & GBR 0.00005 0.00002

rs13393316 ITU & IBS 0.00000 0.00000

rs13393316 ITU & TSI 0.00000 0.00000

rs13393316 ITU & BEB 0.51955 0.00128

rs13393316 ITU & GIH 0.11221 0.00115

rs13393316 PJL & ACB 0.00210 0.00020

rs13393316 PJL & ASW 0.00892 0.00031

rs13393316 PJL & ESN 0.00152 0.00016

rs13393316 PJL & GWD 0.00536 0.00035

rs13393316 PJL & LWK 0.17741 0.00172

rs13393316 PJL & MSL 0.16138 0.00130

rs13393316 PJL & YRI 0.04143 0.00087

rs13393316 PJL & PUR 0.00000 0.00000

rs13393316 PJL & CLM 0.00000 0.00000

rs13393316 PJL & PEL 0.00000 0.00000

rs13393316 PJL & MXL 0.00009 0.00003

rs13393316 PJL & CHS 0.00550 0.00020

rs13393316 PJL & CDX 0.01538 0.00033

rs13393316 PJL & KHV 0.00651 0.00020

rs13393316 PJL & CHB 0.00578 0.00022

rs13393316 PJL & JPT 0.00554 0.00019

rs13393316 PJL & CEU 0.00043 0.00008

rs13393316 PJL & FIN 0.00141 0.00016

rs13393316 PJL & GBR 0.00279 0.00019

rs13393316 PJL & IBS 0.00000 0.00000

rs13393316 PJL & TSI 0.00001 0.00001

rs13393316 PJL & BEB 1.00000 0.00000

rs13393316 PJL & GIH 0.47467 0.00156

rs13393316 PJL & ITU 0.37074 0.00143

rs13393316 STU & ACB 0.00586 0.00036

rs13393316 STU & ASW 0.02471 0.00075

rs13393316 STU & ESN 0.00359 0.00027

rs13393316 STU & GWD 0.01116 0.00047

rs13393316 STU & LWK 0.28748 0.00227

rs13393316 STU & MSL 0.37028 0.00185

rs13393316 STU & YRI 0.07830 0.00114

rs13393316 STU & PUR 0.00000 0.00000

rs13393316 STU & CLM 0.00000 0.00000

rs13393316 STU & PEL 0.00000 0.00000

rs13393316 STU & MXL 0.00012 0.00003

rs13393316 STU & CHS 0.00150 0.00011

rs13393316 STU & CDX 0.00395 0.00016

rs13393316 STU & KHV 0.00374 0.00016

rs13393316 STU & CHB 0.00175 0.00012

rs13393316 STU & JPT 0.00160 0.00011

rs13393316 STU & CEU 0.00139 0.00015

rs13393316 STU & FIN 0.00387 0.00027

rs13393316 STU & GBR 0.00785 0.00039

rs13393316 STU & IBS 0.00001 0.00001

rs13393316 STU & TSI 0.00002 0.00001

rs13393316 STU & BEB 0.79309 0.00084

rs13393316 STU & GIH 0.81938 0.00078

rs13393316 STU & ITU 0.25701 0.00148

rs13393316 STU & PJL 0.80044 0.00089

Locus: rs7791726

=================================

Pop Alleles:

-----------------------------------------------------------

2 3 Total

ACB 146 46 192

ASW 84 38 122

ESN 146 52 198

GWD 186 40 226

LWK 138 60 198

MSL 126 44 170

YRI 151 65 216

PUR 102 106 208

CLM 117 71 188

PEL 110 60 170

MXL 71 57 128

CHS 129 81 210

CDX 122 64 186

KHV 137 61 198

CHB 126 80 206

JPT 141 67 208

CEU 96 102 198

FIN 66 132 198

GBR 68 114 182

IBS 97 117 214

TSI 83 131 214

BEB 107 65 172

GIH 130 76 206

ITU 135 69 204

PJL 124 68 192

STU 134 70 204

Total: 30721936 5008

Locus Population pair P-Value S.E.

----------- --------------------- ------- -------

rs7791726 ASW & ACB 0.19644 0.00283

rs7791726 ESN & ACB 0.63914 0.00257

rs7791726 ESN & ASW 0.37542 0.00348

rs7791726 GWD & ACB 0.14830 0.00282

rs7791726 GWD & ASW 0.00614 0.00043

rs7791726 GWD & ESN 0.03548 0.00138

rs7791726 LWK & ACB 0.17577 0.00280

rs7791726 LWK & ASW 0.90077 0.00098

rs7791726 LWK & ESN 0.43116 0.00320

rs7791726 LWK & GWD 0.00216 0.00025

rs7791726 MSL & ACB 0.71392 0.00229

rs7791726 MSL & ASW 0.36141 0.00301

rs7791726 MSL & ESN 1.00000 0.00000

rs7791726 MSL & GWD 0.06286 0.00167

rs7791726 MSL & LWK 0.35693 0.00370

rs7791726 YRI & ACB 0.18150 0.00308

rs7791726 YRI & ASW 0.90152 0.00082

rs7791726 YRI & ESN 0.44770 0.00371

rs7791726 YRI & GWD 0.00290 0.00039

rs7791726 YRI & LWK 1.00000 0.00000

rs7791726 YRI & MSL 0.36518 0.00332

rs7791726 PUR & ACB 0.00000 0.00000

rs7791726 PUR & ASW 0.00073 0.00016

rs7791726 PUR & ESN 0.00000 0.00000

rs7791726 PUR & GWD 0.00000 0.00000

rs7791726 PUR & LWK 0.00000 0.00000

rs7791726 PUR & MSL 0.00000 0.00000

rs7791726 PUR & YRI 0.00000 0.00000

rs7791726 CLM & ACB 0.00351 0.00040

rs7791726 CLM & ASW 0.27444 0.00344

rs7791726 CLM & ESN 0.01865 0.00110

rs7791726 CLM & GWD 0.00000 0.00000

rs7791726 CLM & LWK 0.13060 0.00330

rs7791726 CLM & MSL 0.01890 0.00091

rs7791726 CLM & YRI 0.11531 0.00294

rs7791726 CLM & PUR 0.00870 0.00082

rs7791726 PEL & ACB 0.02174 0.00107

rs7791726 PEL & ASW 0.53366 0.00308

rs7791726 PEL & ESN 0.06870 0.00173

rs7791726 PEL & GWD 0.00003 0.00002

rs7791726 PEL & LWK 0.31850 0.00353

rs7791726 PEL & MSL 0.07564 0.00191

rs7791726 PEL & YRI 0.32151 0.00393

rs7791726 PEL & PUR 0.00248 0.00033

rs7791726 PEL & CLM 0.66343 0.00308

rs7791726 MXL & ACB 0.00017 0.00006

rs7791726 MXL & ASW 0.03711 0.00120

rs7791726 MXL & ESN 0.00074 0.00012

rs7791726 MXL & GWD 0.00000 0.00000

rs7791726 MXL & LWK 0.01332 0.00074

rs7791726 MXL & MSL 0.00076 0.00013

rs7791726 MXL & YRI 0.00749 0.00050

rs7791726 MXL & PUR 0.27138 0.00380

rs7791726 MXL & CLM 0.24611 0.00384

rs7791726 MXL & PEL 0.11739 0.00270

rs7791726 CHS & ACB 0.00190 0.00029

rs7791726 CHS & ASW 0.20020 0.00322

rs7791726 CHS & ESN 0.00777 0.00067

rs7791726 CHS & GWD 0.00000 0.00000

rs7791726 CHS & LWK 0.09766 0.00285

rs7791726 CHS & MSL 0.01105 0.00069

rs7791726 CHS & YRI 0.06937 0.00214

rs7791726 CHS & PUR 0.01389 0.00085

rs7791726 CHS & CLM 0.91763 0.00075

rs7791726 CHS & PEL 0.52707 0.00373

rs7791726 CHS & MXL 0.30911 0.00332

rs7791726 CDX & ACB 0.03140 0.00120

rs7791726 CDX & ASW 0.61996 0.00266

rs7791726 CDX & ESN 0.09673 0.00247

rs7791726 CDX & GWD 0.00032 0.00011

rs7791726 CDX & LWK 0.44855 0.00394

rs7791726 CDX & MSL 0.08816 0.00201

rs7791726 CDX & YRI 0.38856 0.00348

rs7791726 CDX & PUR 0.00151 0.00025

rs7791726 CDX & CLM 0.52017 0.00378

rs7791726 CDX & PEL 0.91269 0.00073

rs7791726 CDX & MXL 0.07620 0.00226

rs7791726 CDX & CHS 0.40367 0.00389

rs7791726 KHV & ACB 0.14256 0.00312

rs7791726 KHV & ASW 1.00000 0.00000

rs7791726 KHV & ESN 0.37696 0.00422

rs7791726 KHV & GWD 0.00196 0.00028

rs7791726 KHV & LWK 1.00000 0.00000

rs7791726 KHV & MSL 0.29682 0.00345

rs7791726 KHV & YRI 0.91343 0.00105

rs7791726 KHV & PUR 0.00012 0.00006

rs7791726 KHV & CLM 0.16882 0.00274

rs7791726 KHV & PEL 0.37436 0.00332

rs7791726 KHV & MXL 0.01391 0.00083

rs7791726 KHV & CHS 0.11540 0.00253

rs7791726 KHV & CDX 0.50588 0.00424

rs7791726 CHB & ACB 0.00172 0.00026

rs7791726 CHB & ASW 0.18932 0.00350

rs7791726 CHB & ESN 0.00697 0.00058

rs7791726 CHB & GWD 0.00000 0.00000

rs7791726 CHB & LWK 0.07266 0.00232

rs7791726 CHB & MSL 0.00788 0.00062

rs7791726 CHB & YRI 0.06411 0.00215

rs7791726 CHB & PUR 0.01578 0.00089

rs7791726 CHB & CLM 0.83547 0.00137

rs7791726 CHB & PEL 0.52160 0.00317

rs7791726 CHB & MXL 0.35309 0.00351

rs7791726 CHB & CHS 1.00000 0.00000

rs7791726 CHB & CDX 0.40812 0.00394

rs7791726 CHB & KHV 0.09834 0.00252

rs7791726 JPT & ACB 0.07559 0.00204

rs7791726 JPT & ASW 0.90359 0.00093

rs7791726 JPT & ESN 0.19641 0.00360

rs7791726 JPT & GWD 0.00058 0.00014

rs7791726 JPT & LWK 0.74427 0.00216

rs7791726 JPT & MSL 0.21277 0.00361

rs7791726 JPT & YRI 0.67666 0.00332

rs7791726 JPT & PUR 0.00011 0.00004

rs7791726 JPT & CLM 0.29109 0.00334

rs7791726 JPT & PEL 0.58572 0.00312

rs7791726 JPT & MXL 0.02793 0.00124

rs7791726 JPT & CHS 0.18473 0.00337

rs7791726 JPT & CDX 0.67500 0.00314

rs7791726 JPT & KHV 0.83302 0.00183

rs7791726 JPT & CHB 0.17972 0.00340

rs7791726 CEU & ACB 0.00000 0.00000

rs7791726 CEU & ASW 0.00058 0.00015

rs7791726 CEU & ESN 0.00000 0.00000

rs7791726 CEU & GWD 0.00000 0.00000

rs7791726 CEU & LWK 0.00002 0.00001

rs7791726 CEU & MSL 0.00000 0.00000

rs7791726 CEU & YRI 0.00003 0.00002

rs7791726 CEU & PUR 0.92281 0.00078

rs7791726 CEU & CLM 0.00828 0.00066

rs7791726 CEU & PEL 0.00221 0.00025

rs7791726 CEU & MXL 0.25943 0.00346

rs7791726 CEU & CHS 0.00885 0.00071

rs7791726 CEU & CDX 0.00082 0.00016

rs7791726 CEU & KHV 0.00002 0.00001

rs7791726 CEU & CHB 0.01207 0.00088

rs7791726 CEU & JPT 0.00006 0.00003

rs7791726 FIN & ACB 0.00000 0.00000

rs7791726 FIN & ASW 0.00000 0.00000

rs7791726 FIN & ESN 0.00000 0.00000

rs7791726 FIN & GWD 0.00000 0.00000

rs7791726 FIN & LWK 0.00000 0.00000

rs7791726 FIN & MSL 0.00000 0.00000

rs7791726 FIN & YRI 0.00000 0.00000

rs7791726 FIN & PUR 0.00195 0.00030

rs7791726 FIN & CLM 0.00000 0.00000

rs7791726 FIN & PEL 0.00000 0.00000

rs7791726 FIN & MXL 0.00007 0.00003

rs7791726 FIN & CHS 0.00000 0.00000

rs7791726 FIN & CDX 0.00000 0.00000

rs7791726 FIN & KHV 0.00000 0.00000

rs7791726 FIN & CHB 0.00000 0.00000

rs7791726 FIN & JPT 0.00000 0.00000

rs7791726 FIN & CEU 0.00384 0.00050

rs7791726 GBR & ACB 0.00000 0.00000

rs7791726 GBR & ASW 0.00000 0.00000

rs7791726 GBR & ESN 0.00000 0.00000

rs7791726 GBR & GWD 0.00000 0.00000

rs7791726 GBR & LWK 0.00000 0.00000

rs7791726 GBR & MSL 0.00000 0.00000

rs7791726 GBR & YRI 0.00000 0.00000

rs7791726 GBR & PUR 0.02517 0.00136

rs7791726 GBR & CLM 0.00000 0.00000

rs7791726 GBR & PEL 0.00000 0.00000

rs7791726 GBR & MXL 0.00155 0.00023

rs7791726 GBR & CHS 0.00000 0.00000

rs7791726 GBR & CDX 0.00000 0.00000

rs7791726 GBR & KHV 0.00000 0.00000

rs7791726 GBR & CHB 0.00000 0.00000

rs7791726 GBR & JPT 0.00000 0.00000

rs7791726 GBR & CEU 0.02923 0.00138

rs7791726 GBR & FIN 0.45139 0.00391

rs7791726 IBS & ACB 0.00000 0.00000

rs7791726 IBS & ASW 0.00014 0.00007

rs7791726 IBS & ESN 0.00000 0.00000

rs7791726 IBS & GWD 0.00000 0.00000

rs7791726 IBS & LWK 0.00000 0.00000

rs7791726 IBS & MSL 0.00000 0.00000

rs7791726 IBS & YRI 0.00000 0.00000

rs7791726 IBS & PUR 0.50102 0.00383

rs7791726 IBS & CLM 0.00098 0.00022

rs7791726 IBS & PEL 0.00027 0.00011

rs7791726 IBS & MXL 0.07126 0.00218

rs7791726 IBS & CHS 0.00100 0.00020

rs7791726 IBS & CDX 0.00001 0.00001

rs7791726 IBS & KHV 0.00000 0.00000

rs7791726 IBS & CHB 0.00159 0.00027

rs7791726 IBS & JPT 0.00000 0.00000

rs7791726 IBS & CEU 0.55283 0.00396

rs7791726 IBS & FIN 0.01654 0.00105

rs7791726 IBS & GBR 0.12636 0.00301

rs7791726 TSI & ACB 0.00000 0.00000

rs7791726 TSI & ASW 0.00000 0.00000

rs7791726 TSI & ESN 0.00000 0.00000

rs7791726 TSI & GWD 0.00000 0.00000

rs7791726 TSI & LWK 0.00000 0.00000

rs7791726 TSI & MSL 0.00000 0.00000

rs7791726 TSI & YRI 0.00000 0.00000

rs7791726 TSI & PUR 0.03775 0.00187

rs7791726 TSI & CLM 0.00000 0.00000

rs7791726 TSI & PEL 0.00000 0.00000

rs7791726 TSI & MXL 0.00353 0.00037

rs7791726 TSI & CHS 0.00001 0.00001

rs7791726 TSI & CDX 0.00000 0.00000

rs7791726 TSI & KHV 0.00000 0.00000

rs7791726 TSI & CHB 0.00003 0.00002

rs7791726 TSI & JPT 0.00000 0.00000

rs7791726 TSI & CEU 0.05746 0.00189

rs7791726 TSI & FIN 0.25490 0.00348

rs7791726 TSI & GBR 0.83650 0.00168

rs7791726 TSI & IBS 0.20354 0.00344

rs7791726 BEB & ACB 0.00473 0.00042

rs7791726 BEB & ASW 0.26509 0.00330

rs7791726 BEB & ESN 0.02116 0.00112

rs7791726 BEB & GWD 0.00000 0.00000

rs7791726 BEB & LWK 0.15184 0.00280

rs7791726 BEB & MSL 0.02251 0.00119

rs7791726 BEB & YRI 0.13194 0.00288

rs7791726 BEB & PUR 0.01249 0.00092

rs7791726 BEB & CLM 1.00000 0.00000

rs7791726 BEB & PEL 0.65423 0.00315

rs7791726 BEB & MXL 0.28708 0.00328

rs7791726 BEB & CHS 0.91681 0.00086

rs7791726 BEB & CDX 0.51220 0.00313

rs7791726 BEB & KHV 0.18980 0.00314

rs7791726 BEB & CHB 0.91598 0.00079

rs7791726 BEB & JPT 0.27843 0.00347

rs7791726 BEB & CEU 0.00896 0.00069

rs7791726 BEB & FIN 0.00000 0.00000

rs7791726 BEB & GBR 0.00005 0.00005

rs7791726 BEB & IBS 0.00098 0.00021

rs7791726 BEB & TSI 0.00000 0.00000

rs7791726 GIH & ACB 0.00634 0.00052

rs7791726 GIH & ASW 0.33790 0.00325

rs7791726 GIH & ESN 0.02455 0.00121

rs7791726 GIH & GWD 0.00001 0.00001

rs7791726 GIH & LWK 0.17052 0.00294

rs7791726 GIH & MSL 0.02814 0.00119

rs7791726 GIH & YRI 0.14798 0.00286

rs7791726 GIH & PUR 0.00366 0.00044

rs7791726 GIH & CLM 0.91817 0.00085

rs7791726 GIH & PEL 0.82707 0.00161

rs7791726 GIH & MXL 0.17516 0.00295

rs7791726 GIH & CHS 0.76382 0.00212

rs7791726 GIH & CDX 0.66993 0.00314

rs7791726 GIH & KHV 0.21062 0.00399

rs7791726 GIH & CHB 0.76154 0.00221

rs7791726 GIH & JPT 0.34473 0.00370

rs7791726 GIH & CEU 0.00366 0.00047

rs7791726 GIH & FIN 0.00000 0.00000

rs7791726 GIH & GBR 0.00000 0.00000

rs7791726 GIH & IBS 0.00032 0.00011

rs7791726 GIH & TSI 0.00000 0.00000

rs7791726 GIH & BEB 0.91458 0.00091

rs7791726 ITU & ACB 0.03514 0.00131

rs7791726 ITU & ASW 0.63036 0.00261

rs7791726 ITU & ESN 0.10144 0.00259

rs7791726 ITU & GWD 0.00013 0.00007

rs7791726 ITU & LWK 0.45695 0.00369

rs7791726 ITU & MSL 0.11215 0.00250

rs7791726 ITU & YRI 0.46268 0.00422

rs7791726 ITU & PUR 0.00057 0.00014

rs7791726 ITU & CLM 0.46428 0.00352

rs7791726 ITU & PEL 0.82739 0.00142

rs7791726 ITU & MXL 0.06029 0.00189

rs7791726 ITU & CHS 0.36039 0.00418

rs7791726 ITU & CDX 0.91591 0.00094

rs7791726 ITU & KHV 0.52560 0.00346

rs7791726 ITU & CHB 0.31345 0.00383

rs7791726 ITU & JPT 0.75158 0.00233

rs7791726 ITU & CEU 0.00048 0.00018

rs7791726 ITU & FIN 0.00000 0.00000

rs7791726 ITU & GBR 0.00000 0.00000

rs7791726 ITU & IBS 0.00003 0.00003

rs7791726 ITU & TSI 0.00000 0.00000

rs7791726 ITU & BEB 0.45222 0.00361

rs7791726 ITU & GIH 0.53201 0.00392

rs7791726 PJL & ACB 0.02307 0.00108

rs7791726 PJL & ASW 0.46656 0.00342

rs7791726 PJL & ESN 0.06078 0.00190

rs7791726 PJL & GWD 0.00008 0.00004

rs7791726 PJL & LWK 0.32821 0.00326

rs7791726 PJL & MSL 0.05484 0.00161

rs7791726 PJL & YRI 0.28769 0.00375

rs7791726 PJL & PUR 0.00212 0.00034

rs7791726 PJL & CLM 0.67300 0.00260

rs7791726 PJL & PEL 1.00000 0.00000

rs7791726 PJL & MXL 0.12808 0.00276

rs7791726 PJL & CHS 0.53304 0.00318

rs7791726 PJL & CDX 0.91512 0.00104

rs7791726 PJL & KHV 0.39206 0.00367

rs7791726 PJL & CHB 0.53422 0.00314

rs7791726 PJL & JPT 0.52624 0.00344

rs7791726 PJL & CEU 0.00138 0.00021

rs7791726 PJL & FIN 0.00000 0.00000

rs7791726 PJL & GBR 0.00000 0.00000

rs7791726 PJL & IBS 0.00011 0.00006

rs7791726 PJL & TSI 0.00000 0.00000

rs7791726 PJL & BEB 0.66310 0.00311

rs7791726 PJL & GIH 0.83415 0.00168

rs7791726 PJL & ITU 0.75712 0.00218

rs7791726 STU & ACB 0.02606 0.00110

rs7791726 STU & ASW 0.62195 0.00267

rs7791726 STU & ESN 0.08769 0.00245

rs7791726 STU & GWD 0.00010 0.00007

rs7791726 STU & LWK 0.40634 0.00382

rs7791726 STU & MSL 0.09004 0.00223

rs7791726 STU & YRI 0.40280 0.00366

rs7791726 STU & PUR 0.00072 0.00013

rs7791726 STU & CLM 0.52779 0.00344

rs7791726 STU & PEL 0.91436 0.00094

rs7791726 STU & MXL 0.06201 0.00182

rs7791726 STU & CHS 0.42018 0.00353

rs7791726 STU & CDX 1.00000 0.00000

rs7791726 STU & KHV 0.45550 0.00412

rs7791726 STU & CHB 0.35647 0.00383

rs7791726 STU & JPT 0.67652 0.00292

rs7791726 STU & CEU 0.00063 0.00015

rs7791726 STU & FIN 0.00000 0.00000

rs7791726 STU & GBR 0.00000 0.00000

rs7791726 STU & IBS 0.00000 0.00000

rs7791726 STU & TSI 0.00000 0.00000

rs7791726 STU & BEB 0.52749 0.00369

rs7791726 STU & GIH 0.61276 0.00325

rs7791726 STU & ITU 1.00000 0.00000

rs7791726 STU & PJL 0.83258 0.00163

Locus: rs10101195

=================================

Pop Alleles:

-----------------------------------------------------------

1 2 Total

ACB 102 90 192

ASW 59 63 122

ESN 105 93 198

GWD 122 104 226

LWK 103 95 198

MSL 94 76 170

YRI 109 107 216

PUR 55 153 208

CLM 43 145 188

PEL 57 113 170

MXL 32 96 128

CHS 30 180 210

CDX 30 156 186

KHV 30 168 198

CHB 41 165 206

JPT 37 171 208

CEU 44 154 198

FIN 48 150 198

GBR 55 127 182

IBS 69 145 214

TSI 56 158 214

BEB 19 153 172

GIH 31 175 206

ITU 41 163 204

PJL 27 165 192

STU 29 175 204

Total: 14683540 5008

Locus Population pair P-Value S.E.

----------- --------------------- ------- -------

rs10101195 ASW & ACB 0.42475 0.00344

rs10101195 ESN & ACB 1.00000 0.00000

rs10101195 ESN & ASW 0.41888 0.00349

rs10101195 GWD & ACB 0.92145 0.00076

rs10101195 GWD & ASW 0.36971 0.00301

rs10101195 GWD & ESN 0.84582 0.00191

rs10101195 LWK & ACB 0.84010 0.00164

rs10101195 LWK & ASW 0.56545 0.00308

rs10101195 LWK & ESN 0.92016 0.00089

rs10101195 LWK & GWD 0.69705 0.00327

rs10101195 MSL & ACB 0.74933 0.00206

rs10101195 MSL & ASW 0.28429 0.00362

rs10101195 MSL & ESN 0.67926 0.00285

rs10101195 MSL & GWD 0.84035 0.00174

rs10101195 MSL & LWK 0.60013 0.00350

rs10101195 YRI & ACB 0.61835 0.00380

rs10101195 YRI & ASW 0.73244 0.00242

rs10101195 YRI & ESN 0.61969 0.00332

rs10101195 YRI & GWD 0.50443 0.00352

rs10101195 YRI & LWK 0.76586 0.00222

rs10101195 YRI & MSL 0.35776 0.00378

rs10101195 PUR & ACB 0.00000 0.00000

rs10101195 PUR & ASW 0.00009 0.00005

rs10101195 PUR & ESN 0.00000 0.00000

rs10101195 PUR & GWD 0.00000 0.00000

rs10101195 PUR & LWK 0.00000 0.00000

rs10101195 PUR & MSL 0.00000 0.00000

rs10101195 PUR & YRI 0.00000 0.00000

rs10101195 CLM & ACB 0.00000 0.00000

rs10101195 CLM & ASW 0.00000 0.00000

rs10101195 CLM & ESN 0.00001 0.00001

rs10101195 CLM & GWD 0.00000 0.00000

rs10101195 CLM & LWK 0.00000 0.00000

rs10101195 CLM & MSL 0.00000 0.00000

rs10101195 CLM & YRI 0.00000 0.00000

rs10101195 CLM & PUR 0.42333 0.00362

rs10101195 PEL & ACB 0.00019 0.00007

rs10101195 PEL & ASW 0.01643 0.00092

rs10101195 PEL & ESN 0.00022 0.00009

rs10101195 PEL & GWD 0.00009 0.00006

rs10101195 PEL & LWK 0.00035 0.00008

rs10101195 PEL & MSL 0.00008 0.00004

rs10101195 PEL & YRI 0.00087 0.00021

rs10101195 PEL & PUR 0.14722 0.00300

rs10101195 PEL & CLM 0.03390 0.00130

rs10101195 MXL & ACB 0.00000 0.00000

rs10101195 MXL & ASW 0.00013 0.00005

rs10101195 MXL & ESN 0.00000 0.00000

rs10101195 MXL & GWD 0.00000 0.00000

rs10101195 MXL & LWK 0.00001 0.00001

rs10101195 MXL & MSL 0.00000 0.00000

rs10101195 MXL & YRI 0.00000 0.00000

rs10101195 MXL & PUR 0.80136 0.00153

rs10101195 MXL & CLM 0.68792 0.00225

rs10101195 MXL & PEL 0.12132 0.00256

rs10101195 CHS & ACB 0.00000 0.00000

rs10101195 CHS & ASW 0.00000 0.00000

rs10101195 CHS & ESN 0.00000 0.00000

rs10101195 CHS & GWD 0.00000 0.00000

rs10101195 CHS & LWK 0.00000 0.00000

rs10101195 CHS & MSL 0.00000 0.00000

rs10101195 CHS & YRI 0.00000 0.00000

rs10101195 CHS & PUR 0.00198 0.00028

rs10101195 CHS & CLM 0.03592 0.00128

rs10101195 CHS & PEL 0.00000 0.00000

rs10101195 CHS & MXL 0.02097 0.00090

rs10101195 CDX & ACB 0.00000 0.00000

rs10101195 CDX & ASW 0.00000 0.00000

rs10101195 CDX & ESN 0.00000 0.00000

rs10101195 CDX & GWD 0.00000 0.00000

rs10101195 CDX & LWK 0.00000 0.00000

rs10101195 CDX & MSL 0.00000 0.00000

rs10101195 CDX & YRI 0.00000 0.00000

rs10101195 CDX & PUR 0.01431 0.00072

rs10101195 CDX & CLM 0.11367 0.00181

rs10101195 CDX & PEL 0.00032 0.00009

rs10101195 CDX & MXL 0.05818 0.00139

rs10101195 CDX & CHS 0.67526 0.00210

rs10101195 KHV & ACB 0.00000 0.00000

rs10101195 KHV & ASW 0.00000 0.00000

rs10101195 KHV & ESN 0.00000 0.00000

rs10101195 KHV & GWD 0.00000 0.00000

rs10101195 KHV & LWK 0.00000 0.00000

rs10101195 KHV & MSL 0.00000 0.00000

rs10101195 KHV & YRI 0.00000 0.00000

rs10101195 KHV & PUR 0.00774 0.00057

rs10101195 KHV & CLM 0.06827 0.00189

rs10101195 KHV & PEL 0.00001 0.00001

rs10101195 KHV & MXL 0.03183 0.00112

rs10101195 KHV & CHS 0.88760 0.00086

rs10101195 KHV & CDX 0.88941 0.00093

rs10101195 CHB & ACB 0.00000 0.00000

rs10101195 CHB & ASW 0.00000 0.00000

rs10101195 CHB & ESN 0.00000 0.00000

rs10101195 CHB & GWD 0.00000 0.00000

rs10101195 CHB & LWK 0.00000 0.00000

rs10101195 CHB & MSL 0.00000 0.00000

rs10101195 CHB & YRI 0.00000 0.00000

rs10101195 CHB & PUR 0.13726 0.00263

rs10101195 CHB & CLM 0.54074 0.00280

rs10101195 CHB & PEL 0.00315 0.00037

rs10101195 CHB & MXL 0.33638 0.00342

rs10101195 CHB & CHS 0.15217 0.00269

rs10101195 CHB & CDX 0.35631 0.00305

rs10101195 CHB & KHV 0.24181 0.00286

rs10101195 JPT & ACB 0.00000 0.00000

rs10101195 JPT & ASW 0.00000 0.00000

rs10101195 JPT & ESN 0.00000 0.00000

rs10101195 JPT & GWD 0.00000 0.00000

rs10101195 JPT & LWK 0.00000 0.00000

rs10101195 JPT & MSL 0.00000 0.00000

rs10101195 JPT & YRI 0.00000 0.00000

rs10101195 JPT & PUR 0.04465 0.00154

rs10101195 JPT & CLM 0.21061 0.00269

rs10101195 JPT & PEL 0.00040 0.00008

rs10101195 JPT & MXL 0.12943 0.00184

rs10101195 JPT & CHS 0.35071 0.00363

rs10101195 JPT & CDX 0.69126 0.00211

rs10101195 JPT & KHV 0.50417 0.00281

rs10101195 JPT & CHB 0.61660 0.00282

rs10101195 CEU & ACB 0.00000 0.00000

rs10101195 CEU & ASW 0.00000 0.00000

rs10101195 CEU & ESN 0.00000 0.00000

rs10101195 CEU & GWD 0.00000 0.00000

rs10101195 CEU & LWK 0.00000 0.00000

rs10101195 CEU & MSL 0.00000 0.00000

rs10101195 CEU & YRI 0.00000 0.00000

rs10101195 CEU & PUR 0.35009 0.00358

rs10101195 CEU & CLM 0.90420 0.00065

rs10101195 CEU & PEL 0.01699 0.00090

rs10101195 CEU & MXL 0.58927 0.00253

rs10101195 CEU & CHS 0.04157 0.00132

rs10101195 CEU & CDX 0.15285 0.00238

rs10101195 CEU & KHV 0.09413 0.00211

rs10101195 CEU & CHB 0.62321 0.00269

rs10101195 CEU & JPT 0.32084 0.00279

rs10101195 FIN & ACB 0.00000 0.00000

rs10101195 FIN & ASW 0.00000 0.00000

rs10101195 FIN & ESN 0.00000 0.00000

rs10101195 FIN & GWD 0.00000 0.00000

rs10101195 FIN & LWK 0.00000 0.00000

rs10101195 FIN & MSL 0.00000 0.00000

rs10101195 FIN & YRI 0.00000 0.00000

rs10101195 FIN & PUR 0.64593 0.00268

rs10101195 FIN & CLM 0.81333 0.00141

rs10101195 FIN & PEL 0.06666 0.00218

rs10101195 FIN & MXL 0.89458 0.00090

rs10101195 FIN & CHS 0.01241 0.00067

rs10101195 FIN & CDX 0.05451 0.00148

rs10101195 FIN & KHV 0.02931 0.00103

rs10101195 FIN & CHB 0.33242 0.00325

rs10101195 FIN & JPT 0.11632 0.00244

rs10101195 FIN & CEU 0.72117 0.00232

rs10101195 GBR & ACB 0.00000 0.00000

rs10101195 GBR & ASW 0.00125 0.00019

rs10101195 GBR & ESN 0.00000 0.00000

rs10101195 GBR & GWD 0.00000 0.00000

rs10101195 GBR & LWK 0.00002 0.00001

rs10101195 GBR & MSL 0.00000 0.00000

rs10101195 GBR & YRI 0.00004 0.00002

rs10101195 GBR & PUR 0.42836 0.00348

rs10101195 GBR & CLM 0.12629 0.00236

rs10101195 GBR & PEL 0.56965 0.00349

rs10101195 GBR & MXL 0.36996 0.00285

rs10101195 GBR & CHS 0.00017 0.00008

rs10101195 GBR & CDX 0.00172 0.00023

rs10101195 GBR & KHV 0.00038 0.00009

rs10101195 GBR & CHB 0.02588 0.00135

rs10101195 GBR & JPT 0.00431 0.00046

rs10101195 GBR & CEU 0.08173 0.00230

rs10101195 GBR & FIN 0.21019 0.00322

rs10101195 IBS & ACB 0.00001 0.00001

rs10101195 IBS & ASW 0.00526 0.00046

rs10101195 IBS & ESN 0.00000 0.00000

rs10101195 IBS & GWD 0.00005 0.00005

rs10101195 IBS & LWK 0.00003 0.00001

rs10101195 IBS & MSL 0.00007 0.00005

rs10101195 IBS & YRI 0.00015 0.00007

rs10101195 IBS & PUR 0.19697 0.00335

rs10101195 IBS & CLM 0.04308 0.00179

rs10101195 IBS & PEL 0.82466 0.00151

rs10101195 IBS & MXL 0.17743 0.00284

rs10101195 IBS & CHS 0.00001 0.00001

rs10101195 IBS & CDX 0.00026 0.00010

rs10101195 IBS & KHV 0.00005 0.00002

rs10101195 IBS & CHB 0.00585 0.00049

rs10101195 IBS & JPT 0.00059 0.00012

rs10101195 IBS & CEU 0.02600 0.00116

rs10101195 IBS & FIN 0.08072 0.00231

rs10101195 IBS & GBR 0.73954 0.00212

rs10101195 TSI & ACB 0.00000 0.00000

rs10101195 TSI & ASW 0.00003 0.00002

rs10101195 TSI & ESN 0.00000 0.00000

rs10101195 TSI & GWD 0.00000 0.00000

rs10101195 TSI & LWK 0.00000 0.00000

rs10101195 TSI & MSL 0.00000 0.00000

rs10101195 TSI & YRI 0.00000 0.00000

rs10101195 TSI & PUR 1.00000 0.00000

rs10101195 TSI & CLM 0.49102 0.00331

rs10101195 TSI & PEL 0.13881 0.00258

rs10101195 TSI & MXL 0.89859 0.00093

rs10101195 TSI & CHS 0.00254 0.00031

rs10101195 TSI & CDX 0.01578 0.00087

rs10101195 TSI & KHV 0.00636 0.00051

rs10101195 TSI & CHB 0.13591 0.00281

rs10101195 TSI & JPT 0.04419 0.00143

rs10101195 TSI & CEU 0.36015 0.00357

rs10101195 TSI & FIN 0.73264 0.00196

rs10101195 TSI & GBR 0.42647 0.00369

rs10101195 TSI & IBS 0.20672 0.00365

rs10101195 BEB & ACB 0.00000 0.00000

rs10101195 BEB & ASW 0.00000 0.00000

rs10101195 BEB & ESN 0.00000 0.00000

rs10101195 BEB & GWD 0.00000 0.00000

rs10101195 BEB & LWK 0.00000 0.00000

rs10101195 BEB & MSL 0.00000 0.00000

rs10101195 BEB & YRI 0.00000 0.00000

rs10101195 BEB & PUR 0.00025 0.00009

rs10101195 BEB & CLM 0.00325 0.00029

rs10101195 BEB & PEL 0.00000 0.00000

rs10101195 BEB & MXL 0.00207 0.00020

rs10101195 BEB & CHS 0.36326 0.00281

rs10101195 BEB & CDX 0.16741 0.00254

rs10101195 BEB & KHV 0.28144 0.00288

rs10101195 BEB & CHB 0.02284 0.00095

rs10101195 BEB & JPT 0.08074 0.00164

rs10101195 BEB & CEU 0.00572 0.00036

rs10101195 BEB & FIN 0.00089 0.00016

rs10101195 BEB & GBR 0.00002 0.00001

rs10101195 BEB & IBS 0.00000 0.00000

rs10101195 BEB & TSI 0.00010 0.00003

rs10101195 GIH & ACB 0.00000 0.00000

rs10101195 GIH & ASW 0.00000 0.00000

rs10101195 GIH & ESN 0.00000 0.00000

rs10101195 GIH & GWD 0.00000 0.00000

rs10101195 GIH & LWK 0.00000 0.00000

rs10101195 GIH & MSL 0.00000 0.00000

rs10101195 GIH & YRI 0.00000 0.00000

rs10101195 GIH & PUR 0.00517 0.00047

rs10101195 GIH & CLM 0.05568 0.00168

rs10101195 GIH & PEL 0.00001 0.00001

rs10101195 GIH & MXL 0.02981 0.00104

rs10101195 GIH & CHS 0.89121 0.00092

rs10101195 GIH & CDX 0.78054 0.00172

rs10101195 GIH & KHV 1.00000 0.00000

rs10101195 GIH & CHB 0.24246 0.00294

rs10101195 GIH & JPT 0.50890 0.00298

rs10101195 GIH & CEU 0.07386 0.00177

rs10101195 GIH & FIN 0.02387 0.00113

rs10101195 GIH & GBR 0.00060 0.00018

rs10101195 GIH & IBS 0.00007 0.00005

rs10101195 GIH & TSI 0.00544 0.00046

rs10101195 GIH & BEB 0.29028 0.00251

rs10101195 ITU & ACB 0.00000 0.00000

rs10101195 ITU & ASW 0.00000 0.00000

rs10101195 ITU & ESN 0.00000 0.00000

rs10101195 ITU & GWD 0.00000 0.00000

rs10101195 ITU & LWK 0.00000 0.00000

rs10101195 ITU & MSL 0.00000 0.00000

rs10101195 ITU & YRI 0.00000 0.00000

rs10101195 ITU & PUR 0.13385 0.00251

rs10101195 ITU & CLM 0.54069 0.00310

rs10101195 ITU & PEL 0.00458 0.00040

rs10101195 ITU & MXL 0.34269 0.00341

rs10101195 ITU & CHS 0.12041 0.00220

rs10101195 ITU & CDX 0.35878 0.00307

rs10101195 ITU & KHV 0.23970 0.00238

rs10101195 ITU & CHB 1.00000 0.00000

rs10101195 ITU & JPT 0.61747 0.00239

rs10101195 ITU & CEU 0.62680 0.00281

rs10101195 ITU & FIN 0.33620 0.00307

rs10101195 ITU & GBR 0.02376 0.00097

rs10101195 ITU & IBS 0.00566 0.00046

rs10101195 ITU & TSI 0.16518 0.00265

rs10101195 ITU & BEB 0.01841 0.00085

rs10101195 ITU & GIH 0.19364 0.00289

rs10101195 PJL & ACB 0.00000 0.00000

rs10101195 PJL & ASW 0.00000 0.00000

rs10101195 PJL & ESN 0.00000 0.00000

rs10101195 PJL & GWD 0.00000 0.00000

rs10101195 PJL & LWK 0.00000 0.00000

rs10101195 PJL & MSL 0.00000 0.00000

rs10101195 PJL & YRI 0.00000 0.00000

rs10101195 PJL & PUR 0.00303 0.00035

rs10101195 PJL & CLM 0.03523 0.00118

rs10101195 PJL & PEL 0.00003 0.00003

rs10101195 PJL & MXL 0.01822 0.00067

rs10101195 PJL & CHS 1.00000 0.00000

rs10101195 PJL & CDX 0.66680 0.00224

rs10101195 PJL & KHV 0.77504 0.00141

rs10101195 PJL & CHB 0.14109 0.00221

rs10101195 PJL & JPT 0.34228 0.00320

rs10101195 PJL & CEU 0.04692 0.00132

rs10101195 PJL & FIN 0.01442 0.00078

rs10101195 PJL & GBR 0.00026 0.00014

rs10101195 PJL & IBS 0.00003 0.00003

rs10101195 PJL & TSI 0.00316 0.00032

rs10101195 PJL & BEB 0.42910 0.00270

rs10101195 PJL & GIH 0.88864 0.00099

rs10101195 PJL & ITU 0.14645 0.00234

rs10101195 STU & ACB 0.00000 0.00000

rs10101195 STU & ASW 0.00000 0.00000

rs10101195 STU & ESN 0.00000 0.00000

rs10101195 STU & GWD 0.00000 0.00000

rs10101195 STU & LWK 0.00000 0.00000

rs10101195 STU & MSL 0.00000 0.00000

rs10101195 STU & YRI 0.00000 0.00000

rs10101195 STU & PUR 0.00208 0.00029

rs10101195 STU & CLM 0.03725 0.00135

rs10101195 STU & PEL 0.00005 0.00004

rs10101195 STU & MXL 0.01859 0.00084

rs10101195 STU & CHS 1.00000 0.00000

rs10101195 STU & CDX 0.67376 0.00207

rs10101195 STU & KHV 0.88779 0.00086

rs10101195 STU & CHB 0.14883 0.00267

rs10101195 STU & JPT 0.35238 0.00320

rs10101195 STU & CEU 0.03990 0.00138

rs10101195 STU & FIN 0.01039 0.00060

rs10101195 STU & GBR 0.00020 0.00007

rs10101195 STU & IBS 0.00000 0.00000

rs10101195 STU & TSI 0.00281 0.00033

rs10101195 STU & BEB 0.43527 0.00262

rs10101195 STU & GIH 0.88887 0.00094

rs10101195 STU & ITU 0.14658 0.00223

rs10101195 STU & PJL 1.00000 0.00000

Locus: rs36196656

=================================

Pop Alleles:

-----------------------------------------------------------

1 2 Total

ACB 148 44 192

ASW 84 38 122

ESN 168 30 198

GWD 189 37 226

LWK 161 37 198

MSL 140 30 170

YRI 193 23 216

PUR 92 116 208

CLM 87 101 188

PEL 68 102 170

MXL 62 66 128

CHS 111 99 210

CDX 121 65 186

KHV 123 75 198

CHB 136 70 206

JPT 109 99 208

CEU 93 105 198

FIN 85 113 198

GBR 61 121 182

IBS 72 142 214

TSI 84 130 214

BEB 62 110 172

GIH 84 122 206

ITU 68 136 204

PJL 74 118 192

STU 79 125 204

Total: 27542254 5008

Locus Population pair P-Value S.E.

----------- --------------------- ------- -------

rs36196656 ASW & ACB 0.11289 0.00229

rs36196656 ESN & ACB 0.05111 0.00162

rs36196656 ESN & ASW 0.00090 0.00012

rs36196656 GWD & ACB 0.10653 0.00202

rs36196656 GWD & ASW 0.00265 0.00031

rs36196656 GWD & ESN 0.78940 0.00135

rs36196656 LWK & ACB 0.31778 0.00291

rs36196656 LWK & ASW 0.01347 0.00072

rs36196656 LWK & ESN 0.42129 0.00309

rs36196656 LWK & GWD 0.61022 0.00266

rs36196656 MSL & ACB 0.24319 0.00291

rs36196656 MSL & ASW 0.01105 0.00064

rs36196656 MSL & ESN 0.57649 0.00249

rs36196656 MSL & GWD 0.78850 0.00149

rs36196656 MSL & LWK 0.89378 0.00084

rs36196656 YRI & ACB 0.00119 0.00018

rs36196656 YRI & ASW 0.00001 0.00001

rs36196656 YRI & ESN 0.18616 0.00246

rs36196656 YRI & GWD 0.09266 0.00194

rs36196656 YRI & LWK 0.02573 0.00103

rs36196656 YRI & MSL 0.05202 0.00129

rs36196656 PUR & ACB 0.00000 0.00000

rs36196656 PUR & ASW 0.00003 0.00002

rs36196656 PUR & ESN 0.00000 0.00000

rs36196656 PUR & GWD 0.00000 0.00000

rs36196656 PUR & LWK 0.00000 0.00000

rs36196656 PUR & MSL 0.00000 0.00000

rs36196656 PUR & YRI 0.00000 0.00000

rs36196656 CLM & ACB 0.00000 0.00000

rs36196656 CLM & ASW 0.00015 0.00005

rs36196656 CLM & ESN 0.00000 0.00000

rs36196656 CLM & GWD 0.00000 0.00000

rs36196656 CLM & LWK 0.00000 0.00000

rs36196656 CLM & MSL 0.00000 0.00000

rs36196656 CLM & YRI 0.00000 0.00000

rs36196656 CLM & PUR 0.68925 0.00341

rs36196656 PEL & ACB 0.00000 0.00000

rs36196656 PEL & ASW 0.00000 0.00000

rs36196656 PEL & ESN 0.00000 0.00000

rs36196656 PEL & GWD 0.00000 0.00000

rs36196656 PEL & LWK 0.00000 0.00000

rs36196656 PEL & MSL 0.00000 0.00000

rs36196656 PEL & YRI 0.00000 0.00000

rs36196656 PEL & PUR 0.47430 0.00392

rs36196656 PEL & CLM 0.24035 0.00343

rs36196656 MXL & ACB 0.00000 0.00000

rs36196656 MXL & ASW 0.00095 0.00013

rs36196656 MXL & ESN 0.00000 0.00000

rs36196656 MXL & GWD 0.00000 0.00000

rs36196656 MXL & LWK 0.00000 0.00000

rs36196656 MXL & MSL 0.00000 0.00000

rs36196656 MXL & YRI 0.00000 0.00000

rs36196656 MXL & PUR 0.49769 0.00379

rs36196656 MXL & CLM 0.73392 0.00196

rs36196656 MXL & PEL 0.15331 0.00284

rs36196656 CHS & ACB 0.00000 0.00000

rs36196656 CHS & ASW 0.00567 0.00051

rs36196656 CHS & ESN 0.00000 0.00000

rs36196656 CHS & GWD 0.00000 0.00000

rs36196656 CHS & LWK 0.00000 0.00000

rs36196656 CHS & MSL 0.00000 0.00000

rs36196656 CHS & YRI 0.00000 0.00000

rs36196656 CHS & PUR 0.08051 0.00260

rs36196656 CHS & CLM 0.19057 0.00328

rs36196656 CHS & PEL 0.01382 0.00087

rs36196656 CHS & MXL 0.43509 0.00352

rs36196656 CDX & ACB 0.01392 0.00094

rs36196656 CDX & ASW 0.53845 0.00315

rs36196656 CDX & ESN 0.00000 0.00000

rs36196656 CDX & GWD 0.00012 0.00008

rs36196656 CDX & LWK 0.00036 0.00010

rs36196656 CDX & MSL 0.00031 0.00008

rs36196656 CDX & YRI 0.00000 0.00000

rs36196656 CDX & PUR 0.00009 0.00005

rs36196656 CDX & CLM 0.00016 0.00005

rs36196656 CDX & PEL 0.00000 0.00000

rs36196656 CDX & MXL 0.00380 0.00040

rs36196656 CDX & CHS 0.01410 0.00082

rs36196656 KHV & ACB 0.00131 0.00024

rs36196656 KHV & ASW 0.22974 0.00304

rs36196656 KHV & ESN 0.00000 0.00000

rs36196656 KHV & GWD 0.00000 0.00000

rs36196656 KHV & LWK 0.00001 0.00000

rs36196656 KHV & MSL 0.00001 0.00000

rs36196656 KHV & YRI 0.00000 0.00000

rs36196656 KHV & PUR 0.00027 0.00010

rs36196656 KHV & CLM 0.00230 0.00030

rs36196656 KHV & PEL 0.00001 0.00000

rs36196656 KHV & MXL 0.01525 0.00078

rs36196656 KHV & CHS 0.07077 0.00244

rs36196656 KHV & CDX 0.59809 0.00332

rs36196656 CHB & ACB 0.01460 0.00091

rs36196656 CHB & ASW 0.62709 0.00270

rs36196656 CHB & ESN 0.00000 0.00000

rs36196656 CHB & GWD 0.00004 0.00003

rs36196656 CHB & LWK 0.00109 0.00024

rs36196656 CHB & MSL 0.00059 0.00011

rs36196656 CHB & YRI 0.00000 0.00000

rs36196656 CHB & PUR 0.00000 0.00000

rs36196656 CHB & CLM 0.00010 0.00006

rs36196656 CHB & PEL 0.00000 0.00000

rs36196656 CHB & MXL 0.00183 0.00023

rs36196656 CHB & CHS 0.00620 0.00057

rs36196656 CHB & CDX 0.91466 0.00093

rs36196656 CHB & KHV 0.46720 0.00361

rs36196656 JPT & ACB 0.00000 0.00000

rs36196656 JPT & ASW 0.00362 0.00038

rs36196656 JPT & ESN 0.00000 0.00000

rs36196656 JPT & GWD 0.00000 0.00000

rs36196656 JPT & LWK 0.00000 0.00000

rs36196656 JPT & MSL 0.00000 0.00000

rs36196656 JPT & YRI 0.00000 0.00000

rs36196656 JPT & PUR 0.12218 0.00317

rs36196656 JPT & CLM 0.23071 0.00397

rs36196656 JPT & PEL 0.01691 0.00099

rs36196656 JPT & MXL 0.49845 0.00340

rs36196656 JPT & CHS 1.00000 0.00000

rs36196656 JPT & CDX 0.01400 0.00086

rs36196656 JPT & KHV 0.05959 0.00179

rs36196656 JPT & CHB 0.00534 0.00055

rs36196656 CEU & ACB 0.00000 0.00000

rs36196656 CEU & ASW 0.00013 0.00006

rs36196656 CEU & ESN 0.00000 0.00000

rs36196656 CEU & GWD 0.00000 0.00000

rs36196656 CEU & LWK 0.00000 0.00000

rs36196656 CEU & MSL 0.00000 0.00000

rs36196656 CEU & YRI 0.00000 0.00000

rs36196656 CEU & PUR 0.61776 0.00385

rs36196656 CEU & CLM 0.91883 0.00080

rs36196656 CEU & PEL 0.20592 0.00326

rs36196656 CEU & MXL 0.82194 0.00184

rs36196656 CEU & CHS 0.27671 0.00347

rs36196656 CEU & CDX 0.00045 0.00014

rs36196656 CEU & KHV 0.00322 0.00043

rs36196656 CEU & CHB 0.00015 0.00009

rs36196656 CEU & JPT 0.27963 0.00416

rs36196656 FIN & ACB 0.00000 0.00000

rs36196656 FIN & ASW 0.00000 0.00000

rs36196656 FIN & ESN 0.00000 0.00000

rs36196656 FIN & GWD 0.00000 0.00000

rs36196656 FIN & LWK 0.00000 0.00000

rs36196656 FIN & MSL 0.00000 0.00000

rs36196656 FIN & YRI 0.00000 0.00000

rs36196656 FIN & PUR 0.84022 0.00161

rs36196656 FIN & CLM 0.53563 0.00358

rs36196656 FIN & PEL 0.59380 0.00280

rs36196656 FIN & MXL 0.35974 0.00372

rs36196656 FIN & CHS 0.05013 0.00231

rs36196656 FIN & CDX 0.00004 0.00003

rs36196656 FIN & KHV 0.00013 0.00006

rs36196656 FIN & CHB 0.00000 0.00000

rs36196656 FIN & JPT 0.05838 0.00214

rs36196656 FIN & CEU 0.48522 0.00343

rs36196656 GBR & ACB 0.00000 0.00000

rs36196656 GBR & ASW 0.00000 0.00000

rs36196656 GBR & ESN 0.00000 0.00000

rs36196656 GBR & GWD 0.00000 0.00000

rs36196656 GBR & LWK 0.00000 0.00000

rs36196656 GBR & MSL 0.00000 0.00000

rs36196656 GBR & YRI 0.00000 0.00000

rs36196656 GBR & PUR 0.03911 0.00171

rs36196656 GBR & CLM 0.01438 0.00088

rs36196656 GBR & PEL 0.22202 0.00374

rs36196656 GBR & MXL 0.00993 0.00064

rs36196656 GBR & CHS 0.00003 0.00002

rs36196656 GBR & CDX 0.00000 0.00000

rs36196656 GBR & KHV 0.00000 0.00000

rs36196656 GBR & CHB 0.00000 0.00000

rs36196656 GBR & JPT 0.00010 0.00004

rs36196656 GBR & CEU 0.00837 0.00072

rs36196656 GBR & FIN 0.07161 0.00238

rs36196656 IBS & ACB 0.00000 0.00000

rs36196656 IBS & ASW 0.00000 0.00000

rs36196656 IBS & ESN 0.00000 0.00000

rs36196656 IBS & GWD 0.00000 0.00000

rs36196656 IBS & LWK 0.00000 0.00000

rs36196656 IBS & MSL 0.00000 0.00000

rs36196656 IBS & YRI 0.00000 0.00000

rs36196656 IBS & PUR 0.02934 0.00136

rs36196656 IBS & CLM 0.01002 0.00070

rs36196656 IBS & PEL 0.19886 0.00346

rs36196656 IBS & MXL 0.00897 0.00066

rs36196656 IBS & CHS 0.00012 0.00006

rs36196656 IBS & CDX 0.00000 0.00000

rs36196656 IBS & KHV 0.00000 0.00000

rs36196656 IBS & CHB 0.00000 0.00000

rs36196656 IBS & JPT 0.00011 0.00008

rs36196656 IBS & CEU 0.00769 0.00064

rs36196656 IBS & FIN 0.05475 0.00224

rs36196656 IBS & GBR 1.00000 0.00000

rs36196656 TSI & ACB 0.00000 0.00000

rs36196656 TSI & ASW 0.00000 0.00000

rs36196656 TSI & ESN 0.00000 0.00000

rs36196656 TSI & GWD 0.00000 0.00000

rs36196656 TSI & LWK 0.00000 0.00000

rs36196656 TSI & MSL 0.00000 0.00000

rs36196656 TSI & YRI 0.00000 0.00000

rs36196656 TSI & PUR 0.32625 0.00409

rs36196656 TSI & CLM 0.15910 0.00340

rs36196656 TSI & PEL 0.91540 0.00088

rs36196656 TSI & MXL 0.11016 0.00250

rs36196656 TSI & CHS 0.00651 0.00065

rs36196656 TSI & CDX 0.00000 0.00000

rs36196656 TSI & KHV 0.00001 0.00001

rs36196656 TSI & CHB 0.00000 0.00000

rs36196656 TSI & JPT 0.00820 0.00081

rs36196656 TSI & CEU 0.13429 0.00301

rs36196656 TSI & FIN 0.48487 0.00367

rs36196656 TSI & GBR 0.25663 0.00392

rs36196656 TSI & IBS 0.26676 0.00439

rs36196656 BEB & ACB 0.00000 0.00000

rs36196656 BEB & ASW 0.00000 0.00000

rs36196656 BEB & ESN 0.00000 0.00000

rs36196656 BEB & GWD 0.00000 0.00000

rs36196656 BEB & LWK 0.00000 0.00000

rs36196656 BEB & MSL 0.00000 0.00000

rs36196656 BEB & YRI 0.00000 0.00000

rs36196656 BEB & PUR 0.11159 0.00274

rs36196656 BEB & CLM 0.05548 0.00181

rs36196656 BEB & PEL 0.50277 0.00332

rs36196656 BEB & MXL 0.03374 0.00132

rs36196656 BEB & CHS 0.00127 0.00023

rs36196656 BEB & CDX 0.00000 0.00000

rs36196656 BEB & KHV 0.00000 0.00000

rs36196656 BEB & CHB 0.00000 0.00000

rs36196656 BEB & JPT 0.00189 0.00026

rs36196656 BEB & CEU 0.03542 0.00171

rs36196656 BEB & FIN 0.20274 0.00303

rs36196656 BEB & GBR 0.65755 0.00262

rs36196656 BEB & IBS 0.67069 0.00273

rs36196656 BEB & TSI 0.52612 0.00328

rs36196656 GIH & ACB 0.00000 0.00000

rs36196656 GIH & ASW 0.00000 0.00000

rs36196656 GIH & ESN 0.00000 0.00000

rs36196656 GIH & GWD 0.00000 0.00000

rs36196656 GIH & LWK 0.00000 0.00000

rs36196656 GIH & MSL 0.00000 0.00000

rs36196656 GIH & YRI 0.00000 0.00000

rs36196656 GIH & PUR 0.48769 0.00371

rs36196656 GIH & CLM 0.31260 0.00402

rs36196656 GIH & PEL 0.91622 0.00079

rs36196656 GIH & MXL 0.17466 0.00322

rs36196656 GIH & CHS 0.01586 0.00107

rs36196656 GIH & CDX 0.00000 0.00000

rs36196656 GIH & KHV 0.00001 0.00001

rs36196656 GIH & CHB 0.00000 0.00000

rs36196656 GIH & JPT 0.01755 0.00106

rs36196656 GIH & CEU 0.23313 0.00380

rs36196656 GIH & FIN 0.68940 0.00295

rs36196656 GIH & GBR 0.14248 0.00308

rs36196656 GIH & IBS 0.15261 0.00344

rs36196656 GIH & TSI 0.76212 0.00219

rs36196656 GIH & BEB 0.39825 0.00416

rs36196656 ITU & ACB 0.00000 0.00000

rs36196656 ITU & ASW 0.00000 0.00000

rs36196656 ITU & ESN 0.00000 0.00000

rs36196656 ITU & GWD 0.00000 0.00000

rs36196656 ITU & LWK 0.00000 0.00000

rs36196656 ITU & MSL 0.00000 0.00000

rs36196656 ITU & YRI 0.00000 0.00000

rs36196656 ITU & PUR 0.02408 0.00122

rs36196656 ITU & CLM 0.00803 0.00061

rs36196656 ITU & PEL 0.19418 0.00328

rs36196656 ITU & MXL 0.00767 0.00057

rs36196656 ITU & CHS 0.00006 0.00003

rs36196656 ITU & CDX 0.00000 0.00000

rs36196656 ITU & KHV 0.00000 0.00000

rs36196656 ITU & CHB 0.00000 0.00000

rs36196656 ITU & JPT 0.00008 0.00004

rs36196656 ITU & CEU 0.00679 0.00064

rs36196656 ITU & FIN 0.05076 0.00188

rs36196656 ITU & GBR 1.00000 0.00000

rs36196656 ITU & IBS 1.00000 0.00000

rs36196656 ITU & TSI 0.22259 0.00413

rs36196656 ITU & BEB 0.58602 0.00358

rs36196656 ITU & GIH 0.12402 0.00253

rs36196656 PJL & ACB 0.00000 0.00000

rs36196656 PJL & ASW 0.00000 0.00000

rs36196656 PJL & ESN 0.00000 0.00000

rs36196656 PJL & GWD 0.00000 0.00000

rs36196656 PJL & LWK 0.00000 0.00000

rs36196656 PJL & MSL 0.00000 0.00000

rs36196656 PJL & YRI 0.00000 0.00000

rs36196656 PJL & PUR 0.25993 0.00397

rs36196656 PJL & CLM 0.14740 0.00292

rs36196656 PJL & PEL 0.82881 0.00163

rs36196656 PJL & MXL 0.08589 0.00213

rs36196656 PJL & CHS 0.00544 0.00065

rs36196656 PJL & CDX 0.00000 0.00000

rs36196656 PJL & KHV 0.00000 0.00000

rs36196656 PJL & CHB 0.00000 0.00000

rs36196656 PJL & JPT 0.00613 0.00056

rs36196656 PJL & CEU 0.10390 0.00267

rs36196656 PJL & FIN 0.41195 0.00432

rs36196656 PJL & GBR 0.33591 0.00314

rs36196656 PJL & IBS 0.35462 0.00405

rs36196656 PJL & TSI 0.92001 0.00080

rs36196656 PJL & BEB 0.66889 0.00275

rs36196656 PJL & GIH 0.67993 0.00282

rs36196656 PJL & ITU 0.30139 0.00405

rs36196656 STU & ACB 0.00000 0.00000

rs36196656 STU & ASW 0.00000 0.00000

rs36196656 STU & ESN 0.00000 0.00000

rs36196656 STU & GWD 0.00000 0.00000

rs36196656 STU & LWK 0.00000 0.00000

rs36196656 STU & MSL 0.00000 0.00000

rs36196656 STU & YRI 0.00000 0.00000

rs36196656 STU & PUR 0.27563 0.00407

rs36196656 STU & CLM 0.15772 0.00297

rs36196656 STU & PEL 0.83286 0.00159

rs36196656 STU & MXL 0.08936 0.00241

rs36196656 STU & CHS 0.00459 0.00044

rs36196656 STU & CDX 0.00000 0.00000

rs36196656 STU & KHV 0.00000 0.00000

rs36196656 STU & CHB 0.00000 0.00000

rs36196656 STU & JPT 0.00644 0.00065

rs36196656 STU & CEU 0.10882 0.00269

rs36196656 STU & FIN 0.41336 0.00362

rs36196656 STU & GBR 0.29572 0.00370

rs36196656 STU & IBS 0.31212 0.00400

rs36196656 STU & TSI 0.92054 0.00077

rs36196656 STU & BEB 0.59020 0.00332

rs36196656 STU & GIH 0.68286 0.00302

rs36196656 STU & ITU 0.30039 0.00400

rs36196656 STU & PJL 1.00000 0.00000

Locus: rs13072484

=================================

Pop Alleles:

-----------------------------------------------------------

1 3 Total

ACB 64 128 192

ASW 36 86 122

ESN 39 159 198

GWD 62 164 226

LWK 53 145 198

MSL 32 138 170

YRI 44 172 216

PUR 35 173 208

CLM 14 174 188

PEL 17 153 170

MXL 26 102 128

CHS 21 189 210

CDX 29 157 186

KHV 36 162 198

CHB 23 183 206

JPT 20 188 208

CEU 45 153 198

FIN 45 153 198

GBR 51 131 182

IBS 45 169 214

TSI 46 168 214

BEB 44 128 172

GIH 69 137 206

ITU 54 150 204

PJL 59 133 192

STU 61 143 204

Total: 10703938 5008

Locus Population pair P-Value S.E.

----------- --------------------- ------- -------

rs13072484 ASW & ACB 0.53793 0.00300

rs13072484 ESN & ACB 0.00312 0.00036

rs13072484 ESN & ASW 0.05564 0.00152

rs13072484 GWD & ACB 0.19841 0.00364

rs13072484 GWD & ASW 0.70985 0.00194

rs13072484 GWD & ESN 0.06937 0.00182

rs13072484 LWK & ACB 0.18012 0.00323

rs13072484 LWK & ASW 0.60799 0.00255

rs13072484 LWK & ESN 0.12157 0.00271

rs13072484 LWK & GWD 0.91181 0.00084

rs13072484 MSL & ACB 0.00197 0.00025

rs13072484 MSL & ASW 0.03725 0.00110

rs13072484 MSL & ESN 0.89484 0.00083

rs13072484 MSL & GWD 0.05551 0.00170

rs13072484 MSL & LWK 0.07997 0.00203

rs13072484 YRI & ACB 0.00336 0.00040

rs13072484 YRI & ASW 0.06434 0.00158

rs13072484 YRI & ESN 0.90268 0.00090

rs13072484 YRI & GWD 0.10121 0.00258

rs13072484 YRI & LWK 0.13962 0.00241

rs13072484 YRI & MSL 0.79405 0.00163

rs13072484 PUR & ACB 0.00016 0.00005

rs13072484 PUR & ASW 0.00919 0.00057

rs13072484 PUR & ESN 0.52077 0.00306

rs13072484 PUR & GWD 0.00722 0.00054

rs13072484 PUR & LWK 0.01717 0.00098

rs13072484 PUR & MSL 0.68417 0.00225

rs13072484 PUR & YRI 0.38555 0.00347

rs13072484 CLM & ACB 0.00000 0.00000

rs13072484 CLM & ASW 0.00000 0.00000

rs13072484 CLM & ESN 0.00061 0.00010

rs13072484 CLM & GWD 0.00000 0.00000

rs13072484 CLM & LWK 0.00000 0.00000

rs13072484 CLM & MSL 0.00158 0.00020

rs13072484 CLM & YRI 0.00021 0.00007

rs13072484 CLM & PUR 0.00608 0.00044

rs13072484 PEL & ACB 0.00000 0.00000

rs13072484 PEL & ASW 0.00002 0.00001

rs13072484 PEL & ESN 0.01250 0.00065

rs13072484 PEL & GWD 0.00000 0.00000

rs13072484 PEL & LWK 0.00004 0.00002

rs13072484 PEL & MSL 0.02982 0.00099

rs13072484 PEL & YRI 0.00756 0.00049

rs13072484 PEL & PUR 0.06829 0.00157

rs13072484 PEL & CLM 0.45058 0.00225

rs13072484 MXL & ACB 0.01275 0.00076

rs13072484 MXL & ASW 0.10522 0.00207

rs13072484 MXL & ESN 1.00000 0.00000

rs13072484 MXL & GWD 0.16274 0.00269

rs13072484 MXL & LWK 0.18975 0.00284

rs13072484 MXL & MSL 0.76613 0.00136

rs13072484 MXL & YRI 1.00000 0.00000

rs13072484 MXL & PUR 0.46693 0.00292

rs13072484 MXL & CLM 0.00096 0.00016

rs13072484 MXL & PEL 0.01890 0.00070

rs13072484 CHS & ACB 0.00000 0.00000

rs13072484 CHS & ASW 0.00001 0.00001

rs13072484 CHS & ESN 0.00710 0.00054

rs13072484 CHS & GWD 0.00002 0.00002

rs13072484 CHS & LWK 0.00000 0.00000

rs13072484 CHS & MSL 0.01568 0.00064

rs13072484 CHS & YRI 0.00305 0.00031

rs13072484 CHS & PUR 0.04488 0.00139

rs13072484 CHS & CLM 0.38041 0.00242

rs13072484 CHS & PEL 1.00000 0.00000

rs13072484 CHS & MXL 0.01404 0.00059

rs13072484 CDX & ACB 0.00005 0.00003

rs13072484 CDX & ASW 0.00448 0.00031

rs13072484 CDX & ESN 0.34524 0.00297

rs13072484 CDX & GWD 0.00472 0.00041

rs13072484 CDX & LWK 0.00917 0.00056

rs13072484 CDX & MSL 0.48181 0.00276

rs13072484 CDX & YRI 0.24079 0.00338

rs13072484 CDX & PUR 0.78439 0.00156

rs13072484 CDX & CLM 0.01510 0.00068

rs13072484 CDX & PEL 0.15691 0.00224

rs13072484 CDX & MXL 0.29061 0.00253

rs13072484 CDX & CHS 0.12692 0.00221

rs13072484 KHV & ACB 0.00089 0.00019

rs13072484 KHV & ASW 0.02626 0.00103

rs13072484 KHV & ESN 0.79974 0.00140

rs13072484 KHV & GWD 0.02959 0.00134

rs13072484 KHV & LWK 0.05382 0.00145

rs13072484 KHV & MSL 0.89373 0.00082

rs13072484 KHV & YRI 0.61822 0.00288

rs13072484 KHV & PUR 0.79168 0.00159

rs13072484 KHV & CLM 0.00200 0.00019

rs13072484 KHV & PEL 0.02628 0.00088

rs13072484 KHV & MXL 0.66506 0.00207

rs13072484 KHV & CHS 0.02191 0.00088

rs13072484 KHV & CDX 0.58643 0.00281

rs13072484 CHB & ACB 0.00000 0.00000

rs13072484 CHB & ASW 0.00012 0.00006

rs13072484 CHB & ESN 0.02008 0.00098

rs13072484 CHB & GWD 0.00004 0.00003

rs13072484 CHB & LWK 0.00015 0.00008

rs13072484 CHB & MSL 0.04161 0.00125

rs13072484 CHB & YRI 0.01057 0.00061

rs13072484 CHB & PUR 0.12448 0.00214

rs13072484 CHB & CLM 0.23031 0.00248

rs13072484 CHB & PEL 0.73962 0.00150

rs13072484 CHB & MXL 0.02615 0.00083

rs13072484 CHB & CHS 0.75218 0.00161

rs13072484 CHB & CDX 0.23508 0.00263

rs13072484 CHB & KHV 0.05066 0.00138

rs13072484 JPT & ACB 0.00000 0.00000

rs13072484 JPT & ASW 0.00001 0.00001

rs13072484 JPT & ESN 0.00522 0.00042

rs13072484 JPT & GWD 0.00000 0.00000

rs13072484 JPT & LWK 0.00000 0.00000

rs13072484 JPT & MSL 0.01114 0.00060

rs13072484 JPT & YRI 0.00275 0.00027

rs13072484 JPT & PUR 0.04289 0.00113

rs13072484 JPT & CLM 0.47660 0.00235

rs13072484 JPT & PEL 1.00000 0.00000

rs13072484 JPT & MXL 0.00731 0.00039

rs13072484 JPT & CHS 1.00000 0.00000

rs13072484 JPT & CDX 0.09440 0.00191

rs13072484 JPT & KHV 0.01390 0.00063

rs13072484 JPT & CHB 0.62901 0.00203

rs13072484 CEU & ACB 0.02372 0.00115

rs13072484 CEU & ASW 0.18859 0.00321

rs13072484 CEU & ESN 0.54232 0.00317

rs13072484 CEU & GWD 0.30711 0.00344

rs13072484 CEU & LWK 0.41330 0.00335

rs13072484 CEU & MSL 0.37116 0.00312

rs13072484 CEU & YRI 0.63316 0.00254

rs13072484 CEU & PUR 0.16788 0.00274

rs13072484 CEU & CLM 0.00001 0.00000

rs13072484 CEU & PEL 0.00137 0.00020

rs13072484 CEU & MXL 0.68582 0.00242

rs13072484 CEU & CHS 0.00073 0.00013

rs13072484 CEU & CDX 0.09284 0.00194

rs13072484 CEU & KHV 0.31895 0.00338

rs13072484 CEU & CHB 0.00206 0.00025

rs13072484 CEU & JPT 0.00027 0.00007

rs13072484 FIN & ACB 0.02508 0.00108

rs13072484 FIN & ASW 0.18513 0.00250

rs13072484 FIN & ESN 0.54045 0.00285

rs13072484 FIN & GWD 0.31434 0.00381

rs13072484 FIN & LWK 0.42219 0.00357

rs13072484 FIN & MSL 0.37089 0.00308

rs13072484 FIN & YRI 0.63513 0.00250

rs13072484 FIN & PUR 0.17318 0.00288

rs13072484 FIN & CLM 0.00006 0.00004

rs13072484 FIN & PEL 0.00115 0.00020

rs13072484 FIN & MXL 0.68156 0.00210

rs13072484 FIN & CHS 0.00075 0.00013

rs13072484 FIN & CDX 0.09494 0.00217

rs13072484 FIN & KHV 0.31522 0.00339

rs13072484 FIN & CHB 0.00268 0.00029

rs13072484 FIN & JPT 0.00040 0.00008

rs13072484 FIN & CEU 1.00000 0.00000

rs13072484 GBR & ACB 0.31278 0.00350

rs13072484 GBR & ASW 0.79771 0.00159

rs13072484 GBR & ESN 0.06920 0.00179

rs13072484 GBR & GWD 0.91181 0.00092

rs13072484 GBR & LWK 0.81730 0.00146

rs13072484 GBR & MSL 0.04678 0.00146

rs13072484 GBR & YRI 0.07604 0.00232

rs13072484 GBR & PUR 0.00964 0.00060

rs13072484 GBR & CLM 0.00000 0.00000

rs13072484 GBR & PEL 0.00006 0.00005

rs13072484 GBR & MXL 0.14493 0.00258

rs13072484 GBR & CHS 0.00000 0.00000

rs13072484 GBR & CDX 0.00561 0.00045

rs13072484 GBR & KHV 0.02892 0.00118

rs13072484 GBR & CHB 0.00002 0.00001

rs13072484 GBR & JPT 0.00000 0.00000

rs13072484 GBR & CEU 0.24000 0.00285

rs13072484 GBR & FIN 0.24116 0.00310

rs13072484 IBS & ACB 0.00627 0.00048

rs13072484 IBS & ASW 0.08384 0.00208

rs13072484 IBS & ESN 0.80679 0.00174

rs13072484 IBS & GWD 0.11545 0.00276

rs13072484 IBS & LWK 0.19696 0.00352

rs13072484 IBS & MSL 0.60958 0.00267

rs13072484 IBS & YRI 0.90568 0.00088

rs13072484 IBS & PUR 0.32125 0.00306

rs13072484 IBS & CLM 0.00013 0.00004

rs13072484 IBS & PEL 0.00383 0.00032

rs13072484 IBS & MXL 0.89180 0.00090

rs13072484 IBS & CHS 0.00212 0.00025

rs13072484 IBS & CDX 0.20032 0.00288

rs13072484 IBS & KHV 0.54028 0.00320

rs13072484 IBS & CHB 0.00753 0.00051

rs13072484 IBS & JPT 0.00114 0.00018

rs13072484 IBS & CEU 0.72435 0.00220

rs13072484 IBS & FIN 0.71779 0.00231

rs13072484 IBS & GBR 0.12454 0.00243

rs13072484 TSI & ACB 0.00944 0.00070

rs13072484 TSI & ASW 0.11727 0.00234

rs13072484 TSI & ESN 0.71586 0.00212

rs13072484 TSI & GWD 0.14701 0.00278

rs13072484 TSI & LWK 0.25116 0.00304

rs13072484 TSI & MSL 0.52931 0.00272

rs13072484 TSI & YRI 0.81521 0.00162

rs13072484 TSI & PUR 0.26931 0.00354

rs13072484 TSI & CLM 0.00002 0.00001

rs13072484 TSI & PEL 0.00250 0.00024

rs13072484 TSI & MXL 0.88964 0.00088

rs13072484 TSI & CHS 0.00132 0.00025

rs13072484 TSI & CDX 0.15877 0.00251

rs13072484 TSI & KHV 0.46295 0.00346

rs13072484 TSI & CHB 0.00499 0.00033

rs13072484 TSI & JPT 0.00064 0.00011

rs13072484 TSI & CEU 0.81381 0.00148

rs13072484 TSI & FIN 0.81061 0.00169

rs13072484 TSI & GBR 0.15899 0.00282

rs13072484 TSI & IBS 1.00000 0.00000

rs13072484 BEB & ACB 0.10833 0.00251

rs13072484 BEB & ASW 0.50751 0.00309

rs13072484 BEB & ESN 0.21259 0.00334

rs13072484 BEB & GWD 0.73148 0.00241

rs13072484 BEB & LWK 0.81494 0.00183

rs13072484 BEB & MSL 0.14996 0.00262

rs13072484 BEB & YRI 0.27395 0.00275

rs13072484 BEB & PUR 0.03994 0.00137

rs13072484 BEB & CLM 0.00000 0.00000

rs13072484 BEB & PEL 0.00025 0.00007

rs13072484 BEB & MXL 0.34014 0.00294

rs13072484 BEB & CHS 0.00006 0.00003

rs13072484 BEB & CDX 0.02410 0.00102

rs13072484 BEB & KHV 0.09944 0.00201

rs13072484 BEB & CHB 0.00034 0.00009

rs13072484 BEB & JPT 0.00011 0.00006

rs13072484 BEB & CEU 0.54516 0.00300

rs13072484 BEB & FIN 0.54756 0.00304

rs13072484 BEB & GBR 0.63158 0.00253

rs13072484 BEB & IBS 0.32625 0.00318

rs13072484 BEB & TSI 0.40153 0.00305

rs13072484 GIH & ACB 1.00000 0.00000

rs13072484 GIH & ASW 0.46842 0.00326

rs13072484 GIH & ESN 0.00247 0.00028

rs13072484 GIH & GWD 0.17144 0.00326

rs13072484 GIH & LWK 0.15558 0.00307

rs13072484 GIH & MSL 0.00120 0.00018

rs13072484 GIH & YRI 0.00297 0.00040

rs13072484 GIH & PUR 0.00018 0.00007

rs13072484 GIH & CLM 0.00000 0.00000

rs13072484 GIH & PEL 0.00000 0.00000

rs13072484 GIH & MXL 0.00861 0.00050

rs13072484 GIH & CHS 0.00000 0.00000

rs13072484 GIH & CDX 0.00002 0.00001

rs13072484 GIH & KHV 0.00052 0.00010

rs13072484 GIH & CHB 0.00000 0.00000

rs13072484 GIH & JPT 0.00000 0.00000

rs13072484 GIH & CEU 0.02157 0.00098

rs13072484 GIH & FIN 0.02063 0.00101

rs13072484 GIH & GBR 0.26972 0.00415

rs13072484 GIH & IBS 0.00466 0.00042

rs13072484 GIH & TSI 0.00576 0.00052

rs13072484 GIH & BEB 0.11673 0.00241

rs13072484 ITU & ACB 0.15145 0.00300

rs13072484 ITU & ASW 0.60145 0.00265

rs13072484 ITU & ESN 0.12817 0.00254

rs13072484 ITU & GWD 0.82898 0.00177

rs13072484 ITU & LWK 1.00000 0.00000

rs13072484 ITU & MSL 0.08404 0.00202

rs13072484 ITU & YRI 0.16563 0.00297

rs13072484 ITU & PUR 0.02214 0.00103

rs13072484 ITU & CLM 0.00000 0.00000

rs13072484 ITU & PEL 0.00006 0.00002

rs13072484 ITU & MXL 0.23453 0.00270

rs13072484 ITU & CHS 0.00000 0.00000

rs13072484 ITU & CDX 0.00985 0.00069

rs13072484 ITU & KHV 0.05218 0.00154

rs13072484 ITU & CHB 0.00010 0.00005

rs13072484 ITU & JPT 0.00002 0.00001

rs13072484 ITU & CEU 0.41930 0.00375

rs13072484 ITU & FIN 0.41723 0.00372

rs13072484 ITU & GBR 0.81975 0.00166

rs13072484 ITU & IBS 0.20422 0.00314

rs13072484 ITU & TSI 0.25443 0.00344

rs13072484 ITU & BEB 0.90588 0.00088

rs13072484 ITU & GIH 0.13108 0.00336

rs13072484 PJL & ACB 0.66097 0.00318

rs13072484 PJL & ASW 0.89924 0.00086

rs13072484 PJL & ESN 0.01390 0.00071

rs13072484 PJL & GWD 0.51386 0.00330

rs13072484 PJL & LWK 0.43556 0.00387

rs13072484 PJL & MSL 0.01140 0.00064

rs13072484 PJL & YRI 0.01577 0.00094

rs13072484 PJL & PUR 0.00125 0.00020

rs13072484 PJL & CLM 0.00000 0.00000

rs13072484 PJL & PEL 0.00000 0.00000

rs13072484 PJL & MXL 0.03932 0.00138

rs13072484 PJL & CHS 0.00000 0.00000

rs13072484 PJL & CDX 0.00057 0.00011

rs13072484 PJL & KHV 0.00430 0.00043

rs13072484 PJL & CHB 0.00000 0.00000

rs13072484 PJL & JPT 0.00000 0.00000

rs13072484 PJL & CEU 0.08813 0.00241

rs13072484 PJL & FIN 0.08544 0.00225

rs13072484 PJL & GBR 0.56960 0.00323

rs13072484 PJL & IBS 0.03009 0.00129

rs13072484 PJL & TSI 0.04102 0.00138

rs13072484 PJL & BEB 0.29518 0.00310

rs13072484 PJL & GIH 0.59675 0.00321

rs13072484 PJL & ITU 0.37422 0.00372

rs13072484 STU & ACB 0.51448 0.00356

rs13072484 STU & ASW 1.00000 0.00000

rs13072484 STU & ESN 0.02084 0.00098

rs13072484 STU & GWD 0.60021 0.00315

rs13072484 STU & LWK 0.50958 0.00368

rs13072484 STU & MSL 0.01756 0.00104

rs13072484 STU & YRI 0.03160 0.00133

rs13072484 STU & PUR 0.00227 0.00031

rs13072484 STU & CLM 0.00000 0.00000

rs13072484 STU & PEL 0.00000 0.00000

rs13072484 STU & MXL 0.05426 0.00167

rs13072484 STU & CHS 0.00000 0.00000

rs13072484 STU & CDX 0.00124 0.00024

rs13072484 STU & KHV 0.00845 0.00058

rs13072484 STU & CHB 0.00000 0.00000

rs13072484 STU & JPT 0.00000 0.00000

rs13072484 STU & CEU 0.11127 0.00230

rs13072484 STU & FIN 0.11208 0.00266

rs13072484 STU & GBR 0.73736 0.00205

rs13072484 STU & IBS 0.04466 0.00151

rs13072484 STU & TSI 0.05369 0.00165

rs13072484 STU & BEB 0.36039 0.00361

rs13072484 STU & GIH 0.44982 0.00437

rs13072484 STU & ITU 0.50822 0.00344

rs13072484 STU & PJL 0.91374 0.00094

Locus: rs62443267

=================================

Pop Alleles:

-----------------------------------------------------------

2 4 Total

ACB 171 21 192

ASW 105 17 122

ESN 189 9 198

GWD 212 14 226

LWK 162 36 198

MSL 154 16 170

YRI 194 22 216

PUR 166 42 208

CLM 137 51 188

PEL 113 57 170

MXL 101 27 128

CHS 172 38 210

CDX 146 40 186

KHV 157 41 198

CHB 186 20 206

JPT 178 30 208

CEU 150 48 198

FIN 141 57 198

GBR 143 39 182

IBS 160 54 214

TSI 173 41 214

BEB 152 20 172

GIH 180 26 206

ITU 186 18 204

PJL 178 14 192

STU 182 22 204

Total: 4188820 5008

Locus Population pair P-Value S.E.

----------- --------------------- ------- -------

rs62443267 ASW & ACB 0.47884 0.00224

rs62443267 ESN & ACB 0.02174 0.00076

rs62443267 ESN & ASW 0.00524 0.00029

rs62443267 GWD & ACB 0.11241 0.00168

rs62443267 GWD & ASW 0.02875 0.00073

rs62443267 GWD & ESN 0.52416 0.00196

rs62443267 LWK & ACB 0.04742 0.00136

rs62443267 LWK & ASW 0.35770 0.00260

rs62443267 LWK & ESN 0.00002 0.00001

rs62443267 LWK & GWD 0.00017 0.00004

rs62443267 MSL & ACB 0.72812 0.00127

rs62443267 MSL & ASW 0.26296 0.00207

rs62443267 MSL & ESN 0.09536 0.00127

rs62443267 MSL & GWD 0.25381 0.00233

rs62443267 MSL & LWK 0.01562 0.00073

rs62443267 YRI & ACB 0.87242 0.00093

rs62443267 YRI & ASW 0.37638 0.00236

rs62443267 YRI & ESN 0.03879 0.00099

rs62443267 YRI & GWD 0.16514 0.00226

rs62443267 YRI & LWK 0.02324 0.00093

rs62443267 YRI & MSL 0.86333 0.00087

rs62443267 PUR & ACB 0.01304 0.00064

rs62443267 PUR & ASW 0.18006 0.00260

rs62443267 PUR & ESN 0.00000 0.00000

rs62443267 PUR & GWD 0.00002 0.00002

rs62443267 PUR & LWK 0.61114 0.00283

rs62443267 PUR & MSL 0.00318 0.00029

rs62443267 PUR & YRI 0.00445 0.00037

rs62443267 CLM & ACB 0.00006 0.00003

rs62443267 CLM & ASW 0.00703 0.00054

rs62443267 CLM & ESN 0.00000 0.00000

rs62443267 CLM & GWD 0.00000 0.00000

rs62443267 CLM & LWK 0.03712 0.00113

rs62443267 CLM & MSL 0.00001 0.00001

rs62443267 CLM & YRI 0.00000 0.00000

rs62443267 CLM & PUR 0.12686 0.00256

rs62443267 PEL & ACB 0.00000 0.00000

rs62443267 PEL & ASW 0.00018 0.00006

rs62443267 PEL & ESN 0.00000 0.00000

rs62443267 PEL & GWD 0.00000 0.00000

rs62443267 PEL & LWK 0.00088 0.00013

rs62443267 PEL & MSL 0.00000 0.00000

rs62443267 PEL & YRI 0.00000 0.00000

rs62443267 PEL & PUR 0.00405 0.00040

rs62443267 PEL & CLM 0.21088 0.00330

rs62443267 MXL & ACB 0.01518 0.00059

rs62443267 MXL & ASW 0.18179 0.00201

rs62443267 MXL & ESN 0.00000 0.00000

rs62443267 MXL & GWD 0.00004 0.00003

rs62443267 MXL & LWK 0.56356 0.00241

rs62443267 MXL & MSL 0.00794 0.00042

rs62443267 MXL & YRI 0.00682 0.00042

rs62443267 MXL & PUR 0.88987 0.00082

rs62443267 MXL & CLM 0.23265 0.00303

rs62443267 MXL & PEL 0.01974 0.00091

rs62443267 CHS & ACB 0.04627 0.00108

rs62443267 CHS & ASW 0.36427 0.00255

rs62443267 CHS & ESN 0.00002 0.00001

rs62443267 CHS & GWD 0.00022 0.00008

rs62443267 CHS & LWK 1.00000 0.00000

rs62443267 CHS & MSL 0.01733 0.00062

rs62443267 CHS & YRI 0.02503 0.00085

rs62443267 CHS & PUR 0.62308 0.00268

rs62443267 CHS & CLM 0.03945 0.00153

rs62443267 CHS & PEL 0.00096 0.00019

rs62443267 CHS & MXL 0.57148 0.00241

rs62443267 CDX & ACB 0.00816 0.00050

rs62443267 CDX & ASW 0.10282 0.00202

rs62443267 CDX & ESN 0.00000 0.00000

rs62443267 CDX & GWD 0.00000 0.00000

rs62443267 CDX & LWK 0.44720 0.00314

rs62443267 CDX & MSL 0.00243 0.00026

rs62443267 CDX & YRI 0.00184 0.00025

rs62443267 CDX & PUR 0.80531 0.00143

rs62443267 CDX & CLM 0.22642 0.00356

rs62443267 CDX & PEL 0.01224 0.00076

rs62443267 CDX & MXL 1.00000 0.00000

rs62443267 CDX & CHS 0.44657 0.00322

rs62443267 KHV & ACB 0.00888 0.00050

rs62443267 KHV & ASW 0.13692 0.00232

rs62443267 KHV & ESN 0.00000 0.00000

rs62443267 KHV & GWD 0.00000 0.00000

rs62443267 KHV & LWK 0.61339 0.00275

rs62443267 KHV & MSL 0.00356 0.00033

rs62443267 KHV & YRI 0.00443 0.00042

rs62443267 KHV & PUR 0.90362 0.00091

rs62443267 KHV & CLM 0.15201 0.00296

rs62443267 KHV & PEL 0.00648 0.00051

rs62443267 KHV & MXL 1.00000 0.00000

rs62443267 KHV & CHS 0.53988 0.00327

rs62443267 KHV & CDX 0.89978 0.00088

rs62443267 CHB & ACB 0.74096 0.00144

rs62443267 CHB & ASW 0.27362 0.00224

rs62443267 CHB & ESN 0.05324 0.00118

rs62443267 CHB & GWD 0.21223 0.00228

rs62443267 CHB & LWK 0.01558 0.00081

rs62443267 CHB & MSL 1.00000 0.00000

rs62443267 CHB & YRI 0.87382 0.00088

rs62443267 CHB & PUR 0.00368 0.00033

rs62443267 CHB & CLM 0.00002 0.00001

rs62443267 CHB & PEL 0.00000 0.00000

rs62443267 CHB & MXL 0.00522 0.00038

rs62443267 CHB & CHS 0.01637 0.00073

rs62443267 CHB & CDX 0.00167 0.00021

rs62443267 CHB & KHV 0.00223 0.00024

rs62443267 JPT & ACB 0.36695 0.00265

rs62443267 JPT & ASW 1.00000 0.00000

rs62443267 JPT & ESN 0.00054 0.00008

rs62443267 JPT & GWD 0.00644 0.00041

rs62443267 JPT & LWK 0.34803 0.00316

rs62443267 JPT & MSL 0.15856 0.00237

rs62443267 JPT & YRI 0.23401 0.00246

rs62443267 JPT & PUR 0.15600 0.00267

rs62443267 JPT & CLM 0.00301 0.00033

rs62443267 JPT & PEL 0.00000 0.00000

rs62443267 JPT & MXL 0.13574 0.00225

rs62443267 JPT & CHS 0.35289 0.00308

rs62443267 JPT & CDX 0.08515 0.00200

rs62443267 JPT & KHV 0.11325 0.00242

rs62443267 JPT & CHB 0.17358 0.00242

rs62443267 CEU & ACB 0.00058 0.00011

rs62443267 CEU & ASW 0.02968 0.00108

rs62443267 CEU & ESN 0.00000 0.00000

rs62443267 CEU & GWD 0.00000 0.00000

rs62443267 CEU & LWK 0.17243 0.00262

rs62443267 CEU & MSL 0.00021 0.00006

rs62443267 CEU & YRI 0.00025 0.00008

rs62443267 CEU & PUR 0.34471 0.00372

rs62443267 CEU & CLM 0.56246 0.00306

rs62443267 CEU & PEL 0.06496 0.00200

rs62443267 CEU & MXL 0.59535 0.00294

rs62443267 CEU & CHS 0.14312 0.00264

rs62443267 CEU & CDX 0.54253 0.00288

rs62443267 CEU & KHV 0.47172 0.00314

rs62443267 CEU & CHB 0.00016 0.00006

rs62443267 CEU & JPT 0.01763 0.00084

rs62443267 FIN & ACB 0.00000 0.00000

rs62443267 FIN & ASW 0.00229 0.00028

rs62443267 FIN & ESN 0.00000 0.00000

rs62443267 FIN & GWD 0.00000 0.00000

rs62443267 FIN & LWK 0.01745 0.00103

rs62443267 FIN & MSL 0.00000 0.00000

rs62443267 FIN & YRI 0.00000 0.00000

rs62443267 FIN & PUR 0.04894 0.00171

rs62443267 FIN & CLM 0.73518 0.00243

rs62443267 FIN & PEL 0.36541 0.00376

rs62443267 FIN & MXL 0.12809 0.00232

rs62443267 FIN & CHS 0.01417 0.00079

rs62443267 FIN & CDX 0.12669 0.00262

rs62443267 FIN & KHV 0.08141 0.00232

rs62443267 FIN & CHB 0.00000 0.00000

rs62443267 FIN & JPT 0.00037 0.00008

rs62443267 FIN & CEU 0.35787 0.00305

rs62443267 GBR & ACB 0.00714 0.00053

rs62443267 GBR & ASW 0.09772 0.00174

rs62443267 GBR & ESN 0.00000 0.00000

rs62443267 GBR & GWD 0.00000 0.00000

rs62443267 GBR & LWK 0.43997 0.00344

rs62443267 GBR & MSL 0.00214 0.00024

rs62443267 GBR & YRI 0.00177 0.00020

rs62443267 GBR & PUR 0.80251 0.00156

rs62443267 GBR & CLM 0.22035 0.00326

rs62443267 GBR & PEL 0.01231 0.00078

rs62443267 GBR & MXL 1.00000 0.00000

rs62443267 GBR & CHS 0.44537 0.00275

rs62443267 GBR & CDX 1.00000 0.00000

rs62443267 GBR & KHV 0.89857 0.00085

rs62443267 GBR & CHB 0.00181 0.00021

rs62443267 GBR & JPT 0.08719 0.00191

rs62443267 GBR & CEU 0.54266 0.00337

rs62443267 GBR & FIN 0.12417 0.00244

rs62443267 IBS & ACB 0.00013 0.00003

rs62443267 IBS & ASW 0.01704 0.00076

rs62443267 IBS & ESN 0.00000 0.00000

rs62443267 IBS & GWD 0.00000 0.00000

rs62443267 IBS & LWK 0.09495 0.00237

rs62443267 IBS & MSL 0.00003 0.00002

rs62443267 IBS & YRI 0.00006 0.00004

rs62443267 IBS & PUR 0.23890 0.00344

rs62443267 IBS & CLM 0.73237 0.00251

rs62443267 IBS & PEL 0.08560 0.00213

rs62443267 IBS & MXL 0.43248 0.00328

rs62443267 IBS & CHS 0.08081 0.00205

rs62443267 IBS & CDX 0.41127 0.00261

rs62443267 IBS & KHV 0.28901 0.00310

rs62443267 IBS & CHB 0.00005 0.00003

rs62443267 IBS & JPT 0.00699 0.00052

rs62443267 IBS & CEU 0.82041 0.00172

rs62443267 IBS & FIN 0.45000 0.00321

rs62443267 IBS & GBR 0.40949 0.00332

rs62443267 TSI & ACB 0.02659 0.00106

rs62443267 TSI & ASW 0.23326 0.00237

rs62443267 TSI & ESN 0.00000 0.00000

rs62443267 TSI & GWD 0.00002 0.00001

rs62443267 TSI & LWK 0.80037 0.00177

rs62443267 TSI & MSL 0.00890 0.00059

rs62443267 TSI & YRI 0.00957 0.00052

rs62443267 TSI & PUR 0.80768 0.00144

rs62443267 TSI & CLM 0.07629 0.00177

rs62443267 TSI & PEL 0.00148 0.00020

rs62443267 TSI & MXL 0.67528 0.00210

rs62443267 TSI & CHS 0.80336 0.00154

rs62443267 TSI & CDX 0.61353 0.00263

rs62443267 TSI & KHV 0.71240 0.00219

rs62443267 TSI & CHB 0.00749 0.00045

rs62443267 TSI & JPT 0.23993 0.00286

rs62443267 TSI & CEU 0.22979 0.00295

rs62443267 TSI & FIN 0.02719 0.00121

rs62443267 TSI & GBR 0.61668 0.00269

rs62443267 TSI & IBS 0.16453 0.00290

rs62443267 BEB & ACB 0.86877 0.00085

rs62443267 BEB & ASW 0.59533 0.00192

rs62443267 BEB & ESN 0.01840 0.00060

rs62443267 BEB & GWD 0.06987 0.00135

rs62443267 BEB & LWK 0.08050 0.00170

rs62443267 BEB & MSL 0.59546 0.00195

rs62443267 BEB & YRI 0.73992 0.00167

rs62443267 BEB & PUR 0.02508 0.00101

rs62443267 BEB & CLM 0.00032 0.00010

rs62443267 BEB & PEL 0.00000 0.00000

rs62443267 BEB & MXL 0.03601 0.00096

rs62443267 BEB & CHS 0.08383 0.00181

rs62443267 BEB & CDX 0.01701 0.00092

rs62443267 BEB & KHV 0.02479 0.00086

rs62443267 BEB & CHB 0.61437 0.00200

rs62443267 BEB & JPT 0.45212 0.00266

rs62443267 BEB & CEU 0.00188 0.00023

rs62443267 BEB & FIN 0.00005 0.00002

rs62443267 BEB & GBR 0.01610 0.00072

rs62443267 BEB & IBS 0.00073 0.00013

rs62443267 BEB & TSI 0.04817 0.00128

rs62443267 GIH & ACB 0.64113 0.00221

rs62443267 GIH & ASW 0.73936 0.00148

rs62443267 GIH & ESN 0.00412 0.00026

rs62443267 GIH & GWD 0.03007 0.00095

rs62443267 GIH & LWK 0.12639 0.00209

rs62443267 GIH & MSL 0.33214 0.00293

rs62443267 GIH & YRI 0.44238 0.00289

rs62443267 GIH & PUR 0.04602 0.00142

rs62443267 GIH & CLM 0.00025 0.00010

rs62443267 GIH & PEL 0.00000 0.00000

rs62443267 GIH & MXL 0.04691 0.00105

rs62443267 GIH & CHS 0.13507 0.00240

rs62443267 GIH & CDX 0.02121 0.00087

rs62443267 GIH & KHV 0.03224 0.00113

rs62443267 GIH & CHB 0.43767 0.00233

rs62443267 GIH & JPT 0.66986 0.00221

rs62443267 GIH & CEU 0.00284 0.00036

rs62443267 GIH & FIN 0.00001 0.00001

rs62443267 GIH & GBR 0.02735 0.00101

rs62443267 GIH & IBS 0.00111 0.00020

rs62443267 GIH & TSI 0.08470 0.00199

rs62443267 GIH & BEB 0.87442 0.00097

rs62443267 ITU & ACB 0.50411 0.00229

rs62443267 ITU & ASW 0.19399 0.00206

rs62443267 ITU & ESN 0.10992 0.00151

rs62443267 ITU & GWD 0.36026 0.00245

rs62443267 ITU & LWK 0.00844 0.00050

rs62443267 ITU & MSL 0.85771 0.00090

rs62443267 ITU & YRI 0.73956 0.00143

rs62443267 ITU & PUR 0.00159 0.00023

rs62443267 ITU & CLM 0.00000 0.00000

rs62443267 ITU & PEL 0.00000 0.00000

rs62443267 ITU & MXL 0.00249 0.00023

rs62443267 ITU & CHS 0.00687 0.00047

rs62443267 ITU & CDX 0.00053 0.00011

rs62443267 ITU & KHV 0.00106 0.00013

rs62443267 ITU & CHB 0.86526 0.00086

rs62443267 ITU & JPT 0.09348 0.00188

rs62443267 ITU & CEU 0.00003 0.00002

rs62443267 ITU & FIN 0.00000 0.00000

rs62443267 ITU & GBR 0.00038 0.00007

rs62443267 ITU & IBS 0.00000 0.00000

rs62443267 ITU & TSI 0.00278 0.00028

rs62443267 ITU & BEB 0.39339 0.00221

rs62443267 ITU & GIH 0.26921 0.00264

rs62443267 PJL & ACB 0.29044 0.00227

rs62443267 PJL & ASW 0.07721 0.00152

rs62443267 PJL & ESN 0.28664 0.00208

rs62443267 PJL & GWD 0.69638 0.00147

rs62443267 PJL & LWK 0.00135 0.00017

rs62443267 PJL & MSL 0.56887 0.00194

rs62443267 PJL & YRI 0.38540 0.00231

rs62443267 PJL & PUR 0.00030 0.00009

rs62443267 PJL & CLM 0.00000 0.00000

rs62443267 PJL & PEL 0.00000 0.00000

rs62443267 PJL & MXL 0.00054 0.00010

rs62443267 PJL & CHS 0.00157 0.00020

rs62443267 PJL & CDX 0.00011 0.00004

rs62443267 PJL & KHV 0.00023 0.00006

rs62443267 PJL & CHB 0.47504 0.00204

rs62443267 PJL & JPT 0.02539 0.00087

rs62443267 PJL & CEU 0.00000 0.00000

rs62443267 PJL & FIN 0.00000 0.00000

rs62443267 PJL & GBR 0.00006 0.00003

rs62443267 PJL & IBS 0.00000 0.00000

rs62443267 PJL & TSI 0.00066 0.00016

rs62443267 PJL & BEB 0.20670 0.00192

rs62443267 PJL & GIH 0.09516 0.00165

rs62443267 PJL & ITU 0.58361 0.00204

rs62443267 STU & ACB 1.00000 0.00000

rs62443267 STU & ASW 0.48067 0.00223

rs62443267 STU & ESN 0.02365 0.00071

rs62443267 STU & GWD 0.11505 0.00169

rs62443267 STU & LWK 0.04571 0.00130

rs62443267 STU & MSL 0.73193 0.00140

rs62443267 STU & YRI 0.87370 0.00100

rs62443267 STU & PUR 0.00924 0.00059

rs62443267 STU & CLM 0.00010 0.00005

rs62443267 STU & PEL 0.00000 0.00000

rs62443267 STU & MXL 0.01735 0.00076

rs62443267 STU & CHS 0.03548 0.00119

rs62443267 STU & CDX 0.00546 0.00048

rs62443267 STU & KHV 0.00873 0.00055

rs62443267 STU & CHB 0.74651 0.00168

rs62443267 STU & JPT 0.29832 0.00275

rs62443267 STU & CEU 0.00028 0.00008

rs62443267 STU & FIN 0.00000 0.00000

rs62443267 STU & GBR 0.00484 0.00043

rs62443267 STU & IBS 0.00024 0.00008

rs62443267 STU & TSI 0.02051 0.00078

rs62443267 STU & BEB 0.86901 0.00082

rs62443267 STU & GIH 0.64156 0.00185

rs62443267 STU & ITU 0.61524 0.00231

rs62443267 STU & PJL 0.29105 0.00216

Locus: rs10860097

=================================

Pop Alleles:

-----------------------------------------------------------

1 4 Total

ACB 192 0 192

ASW 122 0 122

ESN 198 0 198

GWD 225 1 226

LWK 198 0 198

MSL 170 0 170

YRI 216 0 216

PUR 202 6 208

CLM 186 2 188

PEL 169 1 170

MXL 126 2 128

CHS 189 21 210

CDX 177 9 186

KHV 182 16 198

CHB 188 18 206

JPT 189 19 208

CEU 190 8 198

FIN 195 3 198

GBR 179 3 182

IBS 208 6 214

TSI 207 7 214

BEB 159 13 172

GIH 195 11 206

ITU 192 12 204

PJL 181 11 192

STU 195 9 204

Total: 4830178 5008

Locus Population pair P-Value S.E.

----------- --------------------- ------- -------

rs10860097 ASW & ACB No table

rs10860097 ESN & ACB No table

rs10860097 ESN & ASW No table

rs10860097 GWD & ACB 1.00000 0.00000

rs10860097 GWD & ASW 1.00000 0.00000

rs10860097 GWD & ESN 1.00000 0.00000

rs10860097 LWK & ACB No table

rs10860097 LWK & ASW No table

rs10860097 LWK & ESN No table

rs10860097 LWK & GWD 1.00000 0.00000

rs10860097 MSL & ACB No table

rs10860097 MSL & ASW No table

rs10860097 MSL & ESN No table

rs10860097 MSL & GWD 1.00000 0.00000

rs10860097 MSL & LWK No table

rs10860097 YRI & ACB No table

rs10860097 YRI & ASW No table

rs10860097 YRI & ESN No table

rs10860097 YRI & GWD 1.00000 0.00000

rs10860097 YRI & LWK No table

rs10860097 YRI & MSL No table

rs10860097 PUR & ACB 0.03166 0.00055

rs10860097 PUR & ASW 0.06259 0.00065

rs10860097 PUR & ESN 0.03048 0.00042

rs10860097 PUR & GWD 0.05794 0.00070

rs10860097 PUR & LWK 0.03089 0.00049

rs10860097 PUR & MSL 0.03503 0.00052

rs10860097 PUR & YRI 0.01361 0.00025

rs10860097 CLM & ACB 0.24333 0.00090

rs10860097 CLM & ASW 0.52056 0.00067

rs10860097 CLM & ESN 0.23661 0.00086

rs10860097 CLM & GWD 0.59303 0.00078

rs10860097 CLM & LWK 0.23643 0.00097

rs10860097 CLM & MSL 0.50015 0.00074

rs10860097 CLM & YRI 0.21538 0.00087

rs10860097 CLM & PUR 0.28887 0.00128

rs10860097 PEL & ACB 0.46955 0.00078

rs10860097 PEL & ASW 1.00000 0.00000

rs10860097 PEL & ESN 0.46158 0.00069

rs10860097 PEL & GWD 1.00000 0.00000

rs10860097 PEL & LWK 0.46351 0.00088

rs10860097 PEL & MSL 1.00000 0.00000

rs10860097 PEL & YRI 0.43999 0.00070

rs10860097 PEL & PUR 0.13289 0.00084

rs10860097 PEL & CLM 1.00000 0.00000

rs10860097 MXL & ACB 0.15904 0.00096

rs10860097 MXL & ASW 0.49960 0.00072

rs10860097 MXL & ESN 0.15470 0.00080

rs10860097 MXL & GWD 0.55726 0.00074

rs10860097 MXL & LWK 0.15267 0.00083

rs10860097 MXL & MSL 0.18480 0.00083

rs10860097 MXL & YRI 0.13846 0.00075

rs10860097 MXL & PUR 0.49725 0.00129

rs10860097 MXL & CLM 1.00000 0.00000

rs10860097 MXL & PEL 0.57808 0.00078

rs10860097 CHS & ACB 0.00000 0.00000

rs10860097 CHS & ASW 0.00007 0.00003

rs10860097 CHS & ESN 0.00000 0.00000

rs10860097 CHS & GWD 0.00001 0.00001

rs10860097 CHS & LWK 0.00000 0.00000

rs10860097 CHS & MSL 0.00001 0.00000

rs10860097 CHS & YRI 0.00001 0.00001

rs10860097 CHS & PUR 0.00423 0.00028

rs10860097 CHS & CLM 0.00003 0.00001

rs10860097 CHS & PEL 0.00006 0.00002

rs10860097 CHS & MXL 0.00167 0.00012

rs10860097 CDX & ACB 0.00145 0.00010

rs10860097 CDX & ASW 0.01270 0.00033

rs10860097 CDX & ESN 0.00132 0.00010

rs10860097 CDX & GWD 0.00648 0.00023

rs10860097 CDX & LWK 0.00134 0.00010

rs10860097 CDX & MSL 0.00378 0.00019

rs10860097 CDX & YRI 0.00095 0.00009

rs10860097 CDX & PUR 0.42795 0.00164

rs10860097 CDX & CLM 0.03576 0.00063

rs10860097 CDX & PEL 0.02137 0.00045

rs10860097 CDX & MXL 0.13689 0.00125

rs10860097 CDX & CHS 0.05718 0.00103

rs10860097 KHV & ACB 0.00002 0.00001

rs10860097 KHV & ASW 0.00033 0.00004

rs10860097 KHV & ESN 0.00003 0.00001

rs10860097 KHV & GWD 0.00006 0.00002

rs10860097 KHV & LWK 0.00002 0.00001

rs10860097 KHV & MSL 0.00001 0.00000

rs10860097 KHV & YRI 0.00001 0.00001

rs10860097 KHV & PUR 0.02741 0.00070

rs10860097 KHV & CLM 0.00117 0.00011

rs10860097 KHV & PEL 0.00034 0.00005

rs10860097 KHV & MXL 0.01286 0.00039

rs10860097 KHV & CHS 0.60436 0.00184

rs10860097 KHV & CDX 0.21801 0.00204

rs10860097 CHB & ACB 0.00000 0.00000

rs10860097 CHB & ASW 0.00020 0.00004

rs10860097 CHB & ESN 0.00000 0.00000

rs10860097 CHB & GWD 0.00001 0.00001

rs10860097 CHB & LWK 0.00001 0.00001

rs10860097 CHB & MSL 0.00002 0.00001

rs10860097 CHB & YRI 0.00000 0.00000

rs10860097 CHB & PUR 0.01145 0.00042

rs10860097 CHB & CLM 0.00039 0.00007

rs10860097 CHB & PEL 0.00017 0.00004

rs10860097 CHB & MXL 0.00745 0.00034

rs10860097 CHB & CHS 0.73727 0.00144

rs10860097 CHB & CDX 0.16544 0.00174

rs10860097 CHB & KHV 0.85983 0.00095

rs10860097 JPT & ACB 0.00000 0.00000

rs10860097 JPT & ASW 0.00012 0.00003

rs10860097 JPT & ESN 0.00000 0.00000

rs10860097 JPT & GWD 0.00001 0.00001

rs10860097 JPT & LWK 0.00000 0.00000

rs10860097 JPT & MSL 0.00000 0.00000

rs10860097 JPT & YRI 0.00000 0.00000

rs10860097 JPT & PUR 0.01186 0.00054

rs10860097 JPT & CLM 0.00017 0.00004

rs10860097 JPT & PEL 0.00006 0.00002

rs10860097 JPT & MXL 0.00463 0.00022

rs10860097 JPT & CHS 0.86888 0.00082

rs10860097 JPT & CDX 0.11655 0.00156

rs10860097 JPT & KHV 0.72586 0.00151

rs10860097 JPT & CHB 1.00000 0.00000

rs10860097 CEU & ACB 0.00735 0.00021

rs10860097 CEU & ASW 0.02620 0.00042

rs10860097 CEU & ESN 0.00725 0.00021

rs10860097 CEU & GWD 0.01414 0.00037

rs10860097 CEU & LWK 0.00715 0.00019

rs10860097 CEU & MSL 0.00832 0.00024

rs10860097 CEU & YRI 0.00253 0.00014

rs10860097 CEU & PUR 0.59414 0.00151

rs10860097 CEU & CLM 0.10599 0.00101

rs10860097 CEU & PEL 0.04232 0.00058

rs10860097 CEU & MXL 0.32775 0.00116

rs10860097 CEU & CHS 0.01997 0.00062

rs10860097 CEU & CDX 0.80754 0.00077

rs10860097 CEU & KHV 0.13738 0.00157

rs10860097 CEU & CHB 0.06869 0.00112

rs10860097 CEU & JPT 0.04847 0.00103

rs10860097 FIN & ACB 0.24987 0.00086

rs10860097 FIN & ASW 0.28869 0.00087

rs10860097 FIN & ESN 0.24774 0.00085

rs10860097 FIN & GWD 0.34548 0.00104

rs10860097 FIN & LWK 0.24848 0.00075

rs10860097 FIN & MSL 0.25090 0.00097

rs10860097 FIN & YRI 0.10864 0.00080

rs10860097 FIN & PUR 0.50381 0.00111

rs10860097 FIN & CLM 1.00000 0.00000

rs10860097 FIN & PEL 0.62797 0.00070

rs10860097 FIN & MXL 1.00000 0.00000

rs10860097 FIN & CHS 0.00029 0.00006

rs10860097 FIN & CDX 0.07876 0.00099

rs10860097 FIN & KHV 0.00382 0.00023

rs10860097 FIN & CHB 0.00132 0.00014

rs10860097 FIN & JPT 0.00070 0.00009

rs10860097 FIN & CEU 0.21885 0.00125

rs10860097 GBR & ACB 0.11407 0.00069

rs10860097 GBR & ASW 0.27871 0.00091

rs10860097 GBR & ESN 0.10923 0.00066

rs10860097 GBR & GWD 0.32856 0.00096

rs10860097 GBR & LWK 0.10979 0.00087

rs10860097 GBR & MSL 0.24948 0.00097

rs10860097 GBR & YRI 0.09560 0.00073

rs10860097 GBR & PUR 0.51129 0.00126

rs10860097 GBR & CLM 0.68117 0.00078

rs10860097 GBR & PEL 0.62407 0.00082

rs10860097 GBR & MXL 1.00000 0.00000

rs10860097 GBR & CHS 0.00057 0.00009

rs10860097 GBR & CDX 0.14047 0.00120

rs10860097 GBR & KHV 0.00397 0.00020

rs10860097 GBR & CHB 0.00261 0.00017

rs10860097 GBR & JPT 0.00167 0.00013

rs10860097 GBR & CEU 0.22565 0.00143

rs10860097 GBR & FIN 1.00000 0.00000

rs10860097 IBS & ACB 0.03143 0.00051

rs10860097 IBS & ASW 0.09067 0.00072

rs10860097 IBS & ESN 0.03084 0.00042

rs10860097 IBS & GWD 0.06200 0.00061

rs10860097 IBS & LWK 0.03038 0.00039

rs10860097 IBS & MSL 0.03636 0.00051

rs10860097 IBS & YRI 0.01484 0.00035

rs10860097 IBS & PUR 1.00000 0.00000

rs10860097 IBS & CLM 0.29257 0.00138

rs10860097 IBS & PEL 0.13933 0.00115

rs10860097 IBS & MXL 0.50273 0.00118

rs10860097 IBS & CHS 0.00252 0.00020

rs10860097 IBS & CDX 0.30287 0.00154

rs10860097 IBS & KHV 0.02545 0.00063

rs10860097 IBS & CHB 0.01060 0.00040

rs10860097 IBS & JPT 0.00654 0.00030

rs10860097 IBS & CEU 0.59131 0.00141

rs10860097 IBS & FIN 0.50510 0.00120

rs10860097 IBS & GBR 0.51752 0.00137

rs10860097 TSI & ACB 0.01572 0.00035

rs10860097 TSI & ASW 0.05118 0.00056

rs10860097 TSI & ESN 0.01499 0.00032

rs10860097 TSI & GWD 0.03303 0.00054

rs10860097 TSI & LWK 0.01556 0.00035

rs10860097 TSI & MSL 0.01888 0.00038

rs10860097 TSI & YRI 0.00728 0.00020

rs10860097 TSI & PUR 1.00000 0.00000

rs10860097 TSI & CLM 0.18360 0.00129

rs10860097 TSI & PEL 0.08324 0.00085

rs10860097 TSI & MXL 0.49368 0.00116

rs10860097 TSI & CHS 0.00584 0.00030

rs10860097 TSI & CDX 0.45538 0.00177

rs10860097 TSI & KHV 0.05122 0.00098

rs10860097 TSI & CHB 0.02250 0.00063

rs10860097 TSI & JPT 0.01449 0.00058

rs10860097 TSI & CEU 0.79522 0.00075

rs10860097 TSI & FIN 0.34019 0.00127

rs10860097 TSI & GBR 0.35670 0.00138

rs10860097 TSI & IBS 1.00000 0.00000

rs10860097 BEB & ACB 0.00005 0.00002

rs10860097 BEB & ASW 0.00085 0.00007

rs10860097 BEB & ESN 0.00002 0.00001

rs10860097 BEB & GWD 0.00011 0.00004

rs10860097 BEB & LWK 0.00006 0.00002

rs10860097 BEB & MSL 0.00022 0.00004

rs10860097 BEB & YRI 0.00003 0.00002

rs10860097 BEB & PUR 0.05519 0.00092

rs10860097 BEB & CLM 0.00261 0.00015

rs10860097 BEB & PEL 0.00143 0.00011

rs10860097 BEB & MXL 0.01775 0.00046

rs10860097 BEB & CHS 0.46807 0.00194

rs10860097 BEB & CDX 0.37875 0.00220

rs10860097 BEB & KHV 1.00000 0.00000

rs10860097 BEB & CHB 0.70753 0.00146

rs10860097 BEB & JPT 0.70858 0.00151

rs10860097 BEB & CEU 0.17647 0.00138

rs10860097 BEB & FIN 0.00796 0.00031

rs10860097 BEB & GBR 0.00881 0.00034

rs10860097 BEB & IBS 0.05662 0.00092

rs10860097 BEB & TSI 0.06604 0.00100

rs10860097 GIH & ACB 0.00101 0.00008

rs10860097 GIH & ASW 0.00558 0.00021

rs10860097 GIH & ESN 0.00074 0.00006

rs10860097 GIH & GWD 0.00192 0.00012

rs10860097 GIH & LWK 0.00101 0.00010

rs10860097 GIH & MSL 0.00109 0.00009

rs10860097 GIH & YRI 0.00037 0.00005

rs10860097 GIH & PUR 0.22416 0.00173

rs10860097 GIH & CLM 0.02228 0.00053

rs10860097 GIH & PEL 0.00837 0.00029

rs10860097 GIH & MXL 0.09034 0.00096

rs10860097 GIH & CHS 0.09792 0.00162

rs10860097 GIH & CDX 1.00000 0.00000

rs10860097 GIH & KHV 0.31873 0.00190

rs10860097 GIH & CHB 0.25121 0.00202

rs10860097 GIH & JPT 0.18687 0.00198

rs10860097 GIH & CEU 0.64113 0.00131

rs10860097 GIH & FIN 0.05385 0.00090

rs10860097 GIH & GBR 0.05959 0.00087

rs10860097 GIH & IBS 0.22084 0.00155

rs10860097 GIH & TSI 0.34221 0.00176

rs10860097 GIH & BEB 0.40261 0.00229

rs10860097 ITU & ACB 0.00031 0.00005

rs10860097 ITU & ASW 0.00322 0.00015

rs10860097 ITU & ESN 0.00046 0.00005

rs10860097 ITU & GWD 0.00101 0.00010

rs10860097 ITU & LWK 0.00034 0.00005

rs10860097 ITU & MSL 0.00069 0.00007

rs10860097 ITU & YRI 0.00017 0.00004

rs10860097 ITU & PUR 0.15667 0.00143

rs10860097 ITU & CLM 0.01300 0.00044

rs10860097 ITU & PEL 0.00436 0.00022

rs10860097 ITU & MXL 0.05808 0.00077

rs10860097 ITU & CHS 0.14774 0.00184

rs10860097 ITU & CDX 0.66363 0.00143

rs10860097 ITU & KHV 0.43434 0.00212

rs10860097 ITU & CHB 0.34327 0.00213

rs10860097 ITU & JPT 0.26072 0.00218

rs10860097 ITU & CEU 0.48882 0.00171

rs10860097 ITU & FIN 0.03210 0.00064

rs10860097 ITU & GBR 0.03511 0.00063

rs10860097 ITU & IBS 0.14872 0.00156

rs10860097 ITU & TSI 0.24231 0.00198

rs10860097 ITU & BEB 0.54154 0.00165

rs10860097 ITU & GIH 0.83444 0.00078

rs10860097 PJL & ACB 0.00090 0.00008

rs10860097 PJL & ASW 0.00450 0.00019

rs10860097 PJL & ESN 0.00034 0.00005

rs10860097 PJL & GWD 0.00172 0.00013

rs10860097 PJL & LWK 0.00040 0.00005

rs10860097 PJL & MSL 0.00093 0.00008

rs10860097 PJL & YRI 0.00020 0.00003

rs10860097 PJL & PUR 0.21433 0.00159

rs10860097 PJL & CLM 0.02066 0.00040

rs10860097 PJL & PEL 0.00663 0.00025

rs10860097 PJL & MXL 0.08373 0.00087

rs10860097 PJL & CHS 0.14174 0.00211

rs10860097 PJL & CDX 0.81894 0.00079

rs10860097 PJL & KHV 0.42582 0.00217

rs10860097 PJL & CHB 0.33411 0.00198

rs10860097 PJL & JPT 0.25474 0.00201

rs10860097 PJL & CEU 0.48695 0.00187

rs10860097 PJL & FIN 0.03038 0.00070

rs10860097 PJL & GBR 0.05395 0.00079

rs10860097 PJL & IBS 0.21695 0.00160

rs10860097 PJL & TSI 0.33581 0.00185

rs10860097 PJL & BEB 0.52909 0.00201

rs10860097 PJL & GIH 1.00000 0.00000

rs10860097 PJL & ITU 1.00000 0.00000

rs10860097 STU & ACB 0.00356 0.00015

rs10860097 STU & ASW 0.01600 0.00037

rs10860097 STU & ESN 0.00351 0.00015

rs10860097 STU & GWD 0.00793 0.00032

rs10860097 STU & LWK 0.00356 0.00015

rs10860097 STU & MSL 0.00443 0.00020

rs10860097 STU & YRI 0.00141 0.00011

rs10860097 STU & PUR 0.44468 0.00170

rs10860097 STU & CLM 0.06459 0.00076

rs10860097 STU & PEL 0.02574 0.00052

rs10860097 STU & MXL 0.21345 0.00120

rs10860097 STU & CHS 0.03652 0.00086

rs10860097 STU & CDX 1.00000 0.00000

rs10860097 STU & KHV 0.14883 0.00169

rs10860097 STU & CHB 0.10857 0.00134

rs10860097 STU & JPT 0.07783 0.00133

rs10860097 STU & CEU 1.00000 0.00000

rs10860097 STU & FIN 0.14080 0.00125

rs10860097 STU & GBR 0.14719 0.00119

rs10860097 STU & IBS 0.43734 0.00156

rs10860097 STU & TSI 0.61523 0.00135

rs10860097 STU & BEB 0.26952 0.00198

rs10860097 STU & GIH 0.81866 0.00085

rs10860097 STU & ITU 0.65544 0.00135

rs10860097 STU & PJL 0.64707 0.00150

Locus: rs7240419

=================================

Pop Alleles:

-----------------------------------------------------------

1 3 Total

ACB 41 151 192

ASW 25 97 122

ESN 44 154 198

GWD 60 166 226

LWK 46 152 198

MSL 45 125 170

YRI 43 173 216

PUR 40 168 208

CLM 36 152 188

PEL 27 143 170

MXL 39 89 128

CHS 79 131 210

CDX 103 83 186

KHV 97 101 198

CHB 83 123 206

JPT 91 117 208

CEU 59 139 198

FIN 50 148 198

GBR 46 136 182

IBS 40 174 214

TSI 63 151 214

BEB 80 92 172

GIH 77 129 206

ITU 93 111 204

PJL 78 114 192

STU 93 111 204

Total: 15783430 5008

Locus Population pair P-Value S.E.

----------- --------------------- ------- -------

rs7240419 ASW & ACB 0.88822 0.00077

rs7240419 ESN & ACB 0.90208 0.00088

rs7240419 ESN & ASW 0.78073 0.00147

rs7240419 GWD & ACB 0.25066 0.00347

rs7240419 GWD & ASW 0.24346 0.00310

rs7240419 GWD & ESN 0.30845 0.00352

rs7240419 LWK & ACB 0.71808 0.00211

rs7240419 LWK & ASW 0.58607 0.00258

rs7240419 LWK & ESN 0.90489 0.00085

rs7240419 LWK & GWD 0.42881 0.00393

rs7240419 MSL & ACB 0.26282 0.00326

rs7240419 MSL & ASW 0.27149 0.00281

rs7240419 MSL & ESN 0.39817 0.00359

rs7240419 MSL & GWD 1.00000 0.00000

rs7240419 MSL & LWK 0.55005 0.00289

rs7240419 YRI & ACB 0.80305 0.00162

rs7240419 YRI & ASW 1.00000 0.00000

rs7240419 YRI & ESN 0.62474 0.00225

rs7240419 YRI & GWD 0.11870 0.00277

rs7240419 YRI & LWK 0.47477 0.00301

rs7240419 YRI & MSL 0.14361 0.00222

rs7240419 PUR & ACB 0.62365 0.00267

rs7240419 PUR & ASW 0.88650 0.00082

rs7240419 PUR & ESN 0.46077 0.00335

rs7240419 PUR & GWD 0.08443 0.00208

rs7240419 PUR & LWK 0.32913 0.00321

rs7240419 PUR & MSL 0.10545 0.00219

rs7240419 PUR & YRI 0.90210 0.00087

rs7240419 CLM & ACB 0.61038 0.00244

rs7240419 CLM & ASW 0.88345 0.00080

rs7240419 CLM & ESN 0.53454 0.00306

rs7240419 CLM & GWD 0.08038 0.00210

rs7240419 CLM & LWK 0.38352 0.00320

rs7240419 CLM & MSL 0.10484 0.00219

rs7240419 CLM & YRI 0.89847 0.00087

rs7240419 CLM & PUR 1.00000 0.00000

rs7240419 PEL & ACB 0.22963 0.00304

rs7240419 PEL & ASW 0.34823 0.00252

rs7240419 PEL & ESN 0.14489 0.00252

rs7240419 PEL & GWD 0.01492 0.00081

rs7240419 PEL & LWK 0.08992 0.00209

rs7240419 PEL & MSL 0.02220 0.00096

rs7240419 PEL & YRI 0.35556 0.00302

rs7240419 PEL & PUR 0.41500 0.00296

rs7240419 PEL & CLM 0.48826 0.00284

rs7240419 MXL & ACB 0.08616 0.00215

rs7240419 MXL & ASW 0.08659 0.00182

rs7240419 MXL & ESN 0.11699 0.00207

rs7240419 MXL & GWD 0.46023 0.00277

rs7240419 MXL & LWK 0.15700 0.00261

rs7240419 MXL & MSL 0.51310 0.00266

rs7240419 MXL & YRI 0.03536 0.00126

rs7240419 MXL & PUR 0.02483 0.00096

rs7240419 MXL & CLM 0.02226 0.00090

rs7240419 MXL & PEL 0.00285 0.00026

rs7240419 CHS & ACB 0.00049 0.00011

rs7240419 CHS & ASW 0.00151 0.00022

rs7240419 CHS & ESN 0.00069 0.00016

rs7240419 CHS & GWD 0.01333 0.00074

rs7240419 CHS & LWK 0.00243 0.00041

rs7240419 CHS & MSL 0.02155 0.00105

rs7240419 CHS & YRI 0.00012 0.00007

rs7240419 CHS & PUR 0.00003 0.00002

rs7240419 CHS & CLM 0.00005 0.00002

rs7240419 CHS & PEL 0.00000 0.00000

rs7240419 CHS & MXL 0.19982 0.00311

rs7240419 CDX & ACB 0.00000 0.00000

rs7240419 CDX & ASW 0.00000 0.00000

rs7240419 CDX & ESN 0.00000 0.00000

rs7240419 CDX & GWD 0.00000 0.00000

rs7240419 CDX & LWK 0.00000 0.00000

rs7240419 CDX & MSL 0.00000 0.00000

rs7240419 CDX & YRI 0.00000 0.00000

rs7240419 CDX & PUR 0.00000 0.00000

rs7240419 CDX & CLM 0.00000 0.00000

rs7240419 CDX & PEL 0.00000 0.00000

rs7240419 CDX & MXL 0.00001 0.00001

rs7240419 CDX & CHS 0.00034 0.00010

rs7240419 KHV & ACB 0.00000 0.00000

rs7240419 KHV & ASW 0.00000 0.00000

rs7240419 KHV & ESN 0.00000 0.00000

rs7240419 KHV & GWD 0.00000 0.00000

rs7240419 KHV & LWK 0.00000 0.00000

rs7240419 KHV & MSL 0.00001 0.00001

rs7240419 KHV & YRI 0.00000 0.00000

rs7240419 KHV & PUR 0.00000 0.00000

rs7240419 KHV & CLM 0.00000 0.00000

rs7240419 KHV & PEL 0.00000 0.00000

rs7240419 KHV & MXL 0.00092 0.00018

rs7240419 KHV & CHS 0.02378 0.00113

rs7240419 KHV & CDX 0.21808 0.00358

rs7240419 CHB & ACB 0.00004 0.00002

rs7240419 CHB & ASW 0.00028 0.00007

rs7240419 CHB & ESN 0.00007 0.00004

rs7240419 CHB & GWD 0.00287 0.00034

rs7240419 CHB & LWK 0.00025 0.00008

rs7240419 CHB & MSL 0.00660 0.00061

rs7240419 CHB & YRI 0.00000 0.00000

rs7240419 CHB & PUR 0.00000 0.00000

rs7240419 CHB & CLM 0.00003 0.00003

rs7240419 CHB & PEL 0.00000 0.00000

rs7240419 CHB & MXL 0.07879 0.00194

rs7240419 CHB & CHS 0.61705 0.00359

rs7240419 CHB & CDX 0.00323 0.00041

rs7240419 CHB & KHV 0.08929 0.00248

rs7240419 JPT & ACB 0.00001 0.00001

rs7240419 JPT & ASW 0.00003 0.00003

rs7240419 JPT & ESN 0.00003 0.00003

rs7240419 JPT & GWD 0.00027 0.00010

rs7240419 JPT & LWK 0.00002 0.00001

rs7240419 JPT & MSL 0.00063 0.00014

rs7240419 JPT & YRI 0.00000 0.00000

rs7240419 JPT & PUR 0.00000 0.00000

rs7240419 JPT & CLM 0.00000 0.00000

rs7240419 JPT & PEL 0.00000 0.00000

rs7240419 JPT & MXL 0.01452 0.00078

rs7240419 JPT & CHS 0.23284 0.00415

rs7240419 JPT & CDX 0.02280 0.00119

rs7240419 JPT & KHV 0.31973 0.00416

rs7240419 JPT & CHB 0.48498 0.00349

rs7240419 CEU & ACB 0.06582 0.00199

rs7240419 CEU & ASW 0.06700 0.00188

rs7240419 CEU & ESN 0.10973 0.00245

rs7240419 CEU & GWD 0.51364 0.00342

rs7240419 CEU & LWK 0.17125 0.00289

rs7240419 CEU & MSL 0.48901 0.00327

rs7240419 CEU & YRI 0.02259 0.00108

rs7240419 CEU & PUR 0.01480 0.00079

rs7240419 CEU & CLM 0.01770 0.00091

rs7240419 CEU & PEL 0.00182 0.00022

rs7240419 CEU & MXL 0.90150 0.00092

rs7240419 CEU & CHS 0.11661 0.00262

rs7240419 CEU & CDX 0.00000 0.00000

rs7240419 CEU & KHV 0.00005 0.00002

rs7240419 CEU & CHB 0.02519 0.00121

rs7240419 CEU & JPT 0.00371 0.00041

rs7240419 FIN & ACB 0.40137 0.00324

rs7240419 FIN & ASW 0.34328 0.00329

rs7240419 FIN & ESN 0.55188 0.00313

rs7240419 FIN & GWD 0.82504 0.00145

rs7240419 FIN & LWK 0.72149 0.00226

rs7240419 FIN & MSL 0.80842 0.00165

rs7240419 FIN & YRI 0.19353 0.00320

rs7240419 FIN & PUR 0.15245 0.00268

rs7240419 FIN & CLM 0.17511 0.00287

rs7240419 FIN & PEL 0.03188 0.00120

rs7240419 FIN & MXL 0.31033 0.00314

rs7240419 FIN & CHS 0.00749 0.00061

rs7240419 FIN & CDX 0.00000 0.00000

rs7240419 FIN & KHV 0.00000 0.00000

rs7240419 FIN & CHB 0.00131 0.00027

rs7240419 FIN & JPT 0.00020 0.00012

rs7240419 FIN & CEU 0.37182 0.00380

rs7240419 GBR & ACB 0.38755 0.00347

rs7240419 GBR & ASW 0.33642 0.00278

rs7240419 GBR & ESN 0.54842 0.00275

rs7240419 GBR & GWD 0.82209 0.00163

rs7240419 GBR & LWK 0.71839 0.00213

rs7240419 GBR & MSL 0.80665 0.00162

rs7240419 GBR & YRI 0.22691 0.00339

rs7240419 GBR & PUR 0.17658 0.00266

rs7240419 GBR & CLM 0.17548 0.00291

rs7240419 GBR & PEL 0.03531 0.00116

rs7240419 GBR & MXL 0.36442 0.00301

rs7240419 GBR & CHS 0.00990 0.00075

rs7240419 GBR & CDX 0.00000 0.00000

rs7240419 GBR & KHV 0.00000 0.00000

rs7240419 GBR & CHB 0.00141 0.00023

rs7240419 GBR & JPT 0.00011 0.00005

rs7240419 GBR & CEU 0.36072 0.00399

rs7240419 GBR & FIN 1.00000 0.00000

rs7240419 IBS & ACB 0.53155 0.00276

rs7240419 IBS & ASW 0.77412 0.00168

rs7240419 IBS & ESN 0.39839 0.00333

rs7240419 IBS & GWD 0.05301 0.00169

rs7240419 IBS & LWK 0.27180 0.00313

rs7240419 IBS & MSL 0.08339 0.00183

rs7240419 IBS & YRI 0.80836 0.00155

rs7240419 IBS & PUR 0.90191 0.00085

rs7240419 IBS & CLM 1.00000 0.00000

rs7240419 IBS & PEL 0.50087 0.00289

rs7240419 IBS & MXL 0.01738 0.00085

rs7240419 IBS & CHS 0.00001 0.00001

rs7240419 IBS & CDX 0.00000 0.00000

rs7240419 IBS & KHV 0.00000 0.00000

rs7240419 IBS & CHB 0.00000 0.00000

rs7240419 IBS & JPT 0.00000 0.00000

rs7240419 IBS & CEU 0.01033 0.00069

rs7240419 IBS & FIN 0.11929 0.00263

rs7240419 IBS & GBR 0.14187 0.00283

rs7240419 TSI & ACB 0.06734 0.00197

rs7240419 TSI & ASW 0.07326 0.00179

rs7240419 TSI & ESN 0.11639 0.00255

rs7240419 TSI & GWD 0.52755 0.00357

rs7240419 TSI & LWK 0.18089 0.00340

rs7240419 TSI & MSL 0.56986 0.00319

rs7240419 TSI & YRI 0.02435 0.00119

rs7240419 TSI & PUR 0.01700 0.00084

rs7240419 TSI & CLM 0.02032 0.00109

rs7240419 TSI & PEL 0.00297 0.00036

rs7240419 TSI & MXL 0.90308 0.00081

rs7240419 TSI & CHS 0.08181 0.00229

rs7240419 TSI & CDX 0.00000 0.00000

rs7240419 TSI & KHV 0.00003 0.00002

rs7240419 TSI & CHB 0.02407 0.00125

rs7240419 TSI & JPT 0.00167 0.00027

rs7240419 TSI & CEU 1.00000 0.00000

rs7240419 TSI & FIN 0.37930 0.00355

rs7240419 TSI & GBR 0.36481 0.00362

rs7240419 TSI & IBS 0.01327 0.00081

rs7240419 BEB & ACB 0.00000 0.00000

rs7240419 BEB & ASW 0.00002 0.00002

rs7240419 BEB & ESN 0.00004 0.00004

rs7240419 BEB & GWD 0.00014 0.00006

rs7240419 BEB & LWK 0.00000 0.00000

rs7240419 BEB & MSL 0.00003 0.00002

rs7240419 BEB & YRI 0.00000 0.00000

rs7240419 BEB & PUR 0.00000 0.00000

rs7240419 BEB & CLM 0.00000 0.00000

rs7240419 BEB & PEL 0.00000 0.00000

rs7240419 BEB & MXL 0.00593 0.00048

rs7240419 BEB & CHS 0.09248 0.00228

rs7240419 BEB & CDX 0.11115 0.00265

rs7240419 BEB & KHV 0.67179 0.00229

rs7240419 BEB & CHB 0.24689 0.00347

rs7240419 BEB & JPT 0.60000 0.00342

rs7240419 BEB & CEU 0.00116 0.00022

rs7240419 BEB & FIN 0.00001 0.00000

rs7240419 BEB & GBR 0.00008 0.00004

rs7240419 BEB & IBS 0.00000 0.00000

rs7240419 BEB & TSI 0.00054 0.00011

rs7240419 GIH & ACB 0.00073 0.00020

rs7240419 GIH & ASW 0.00135 0.00020

rs7240419 GIH & ESN 0.00105 0.00025

rs7240419 GIH & GWD 0.01843 0.00107

rs7240419 GIH & LWK 0.00252 0.00025

rs7240419 GIH & MSL 0.02767 0.00107

rs7240419 GIH & YRI 0.00009 0.00004

rs7240419 GIH & PUR 0.00002 0.00001

rs7240419 GIH & CLM 0.00004 0.00002

rs7240419 GIH & PEL 0.00001 0.00001

rs7240419 GIH & MXL 0.23331 0.00334

rs7240419 GIH & CHS 1.00000 0.00000

rs7240419 GIH & CDX 0.00020 0.00006

rs7240419 GIH & KHV 0.02346 0.00126

rs7240419 GIH & CHB 0.61346 0.00357

rs7240419 GIH & JPT 0.19379 0.00328

rs7240419 GIH & CEU 0.11507 0.00250

rs7240419 GIH & FIN 0.01023 0.00076

rs7240419 GIH & GBR 0.01422 0.00095

rs7240419 GIH & IBS 0.00009 0.00004

rs7240419 GIH & TSI 0.09778 0.00265

rs7240419 GIH & BEB 0.07904 0.00250

rs7240419 ITU & ACB 0.00000 0.00000

rs7240419 ITU & ASW 0.00000 0.00000

rs7240419 ITU & ESN 0.00000 0.00000

rs7240419 ITU & GWD 0.00001 0.00001

rs7240419 ITU & LWK 0.00000 0.00000

rs7240419 ITU & MSL 0.00025 0.00009

rs7240419 ITU & YRI 0.00000 0.00000

rs7240419 ITU & PUR 0.00000 0.00000

rs7240419 ITU & CLM 0.00000 0.00000

rs7240419 ITU & PEL 0.00000 0.00000

rs7240419 ITU & MXL 0.00747 0.00054

rs7240419 ITU & CHS 0.11254 0.00317

rs7240419 ITU & CDX 0.05209 0.00195

rs7240419 ITU & KHV 0.55111 0.00342

rs7240419 ITU & CHB 0.32507 0.00412

rs7240419 ITU & JPT 0.76832 0.00223

rs7240419 ITU & CEU 0.00117 0.00019

rs7240419 ITU & FIN 0.00001 0.00001

rs7240419 ITU & GBR 0.00004 0.00002

rs7240419 ITU & IBS 0.00000 0.00000

rs7240419 ITU & TSI 0.00091 0.00018

rs7240419 ITU & BEB 0.91711 0.00077

rs7240419 ITU & GIH 0.10457 0.00261

rs7240419 PJL & ACB 0.00013 0.00005

rs7240419 PJL & ASW 0.00021 0.00005

rs7240419 PJL & ESN 0.00022 0.00010

rs7240419 PJL & GWD 0.00221 0.00028

rs7240419 PJL & LWK 0.00032 0.00013

rs7240419 PJL & MSL 0.00610 0.00050

rs7240419 PJL & YRI 0.00000 0.00000

rs7240419 PJL & PUR 0.00000 0.00000

rs7240419 PJL & CLM 0.00005 0.00004

rs7240419 PJL & PEL 0.00000 0.00000

rs7240419 PJL & MXL 0.07477 0.00171

rs7240419 PJL & CHS 0.53602 0.00374

rs7240419 PJL & CDX 0.00555 0.00055

rs7240419 PJL & KHV 0.10347 0.00259

rs7240419 PJL & CHB 1.00000 0.00000

rs7240419 PJL & JPT 0.54520 0.00370

rs7240419 PJL & CEU 0.02695 0.00135

rs7240419 PJL & FIN 0.00184 0.00030

rs7240419 PJL & GBR 0.00236 0.00029

rs7240419 PJL & IBS 0.00000 0.00000

rs7240419 PJL & TSI 0.02150 0.00112

rs7240419 PJL & BEB 0.29027 0.00358

rs7240419 PJL & GIH 0.53606 0.00344

rs7240419 PJL & ITU 0.36005 0.00406

rs7240419 STU & ACB 0.00000 0.00000

rs7240419 STU & ASW 0.00000 0.00000

rs7240419 STU & ESN 0.00000 0.00000

rs7240419 STU & GWD 0.00009 0.00005

rs7240419 STU & LWK 0.00000 0.00000

rs7240419 STU & MSL 0.00013 0.00005

rs7240419 STU & YRI 0.00000 0.00000

rs7240419 STU & PUR 0.00000 0.00000

rs7240419 STU & CLM 0.00000 0.00000

rs7240419 STU & PEL 0.00000 0.00000

rs7240419 STU & MXL 0.00785 0.00063

rs7240419 STU & CHS 0.10889 0.00275

rs7240419 STU & CDX 0.05605 0.00178

rs7240419 STU & KHV 0.55280 0.00310

rs7240419 STU & CHB 0.31730 0.00392

rs7240419 STU & JPT 0.76879 0.00227

rs7240419 STU & CEU 0.00104 0.00018

rs7240419 STU & FIN 0.00002 0.00001

rs7240419 STU & GBR 0.00000 0.00000

rs7240419 STU & IBS 0.00000 0.00000

rs7240419 STU & TSI 0.00079 0.00017

rs7240419 STU & BEB 0.91792 0.00090

rs7240419 STU & GIH 0.10931 0.00302

rs7240419 STU & ITU 1.00000 0.00000

rs7240419 STU & PJL 0.36814 0.00385

Locus: rs6076187

=================================

Pop Alleles:

-----------------------------------------------------------

1 3 Total

ACB 47 145 192

ASW 34 88 122

ESN 75 123 198

GWD 50 176 226

LWK 73 125 198

MSL 39 131 170

YRI 58 158 216

PUR 18 190 208

CLM 11 177 188

PEL 9 161 170

MXL 9 119 128

CHS 18 192 210

CDX 17 169 186

KHV 18 180 198

CHB 32 174 206

JPT 22 186 208

CEU 4 194 198

FIN 14 184 198

GBR 7 175 182

IBS 8 206 214

TSI 12 202 214

BEB 10 162 172

GIH 2 204 206

ITU 8 196 204

PJL 5 187 192

STU 14 190 204

Total: 614 4394 5008

Locus Population pair P-Value S.E.

----------- --------------------- ------- -------

rs6076187 ASW & ACB 0.50960 0.00292

rs6076187 ESN & ACB 0.00501 0.00045

rs6076187 ESN & ASW 0.07259 0.00179

rs6076187 GWD & ACB 0.63923 0.00276

rs6076187 GWD & ASW 0.23978 0.00304

rs6076187 GWD & ESN 0.00040 0.00010

rs6076187 LWK & ACB 0.00842 0.00070

rs6076187 LWK & ASW 0.10882 0.00222

rs6076187 LWK & ESN 0.91892 0.00102

rs6076187 LWK & GWD 0.00094 0.00017

rs6076187 MSL & ACB 0.80422 0.00149

rs6076187 MSL & ASW 0.41192 0.00312

rs6076187 MSL & ESN 0.00253 0.00029

rs6076187 MSL & GWD 0.90370 0.00084

rs6076187 MSL & LWK 0.00468 0.00040

rs6076187 YRI & ACB 0.65351 0.00274

rs6076187 YRI & ASW 0.89818 0.00102

rs6076187 YRI & ESN 0.01997 0.00113

rs6076187 YRI & GWD 0.27009 0.00357

rs6076187 YRI & LWK 0.03559 0.00141

rs6076187 YRI & MSL 0.40665 0.00301

rs6076187 PUR & ACB 0.00002 0.00002

rs6076187 PUR & ASW 0.00001 0.00001

rs6076187 PUR & ESN 0.00000 0.00000

rs6076187 PUR & GWD 0.00011 0.00004

rs6076187 PUR & LWK 0.00000 0.00000

rs6076187 PUR & MSL 0.00011 0.00004

rs6076187 PUR & YRI 0.00000 0.00000

rs6076187 CLM & ACB 0.00000 0.00000

rs6076187 CLM & ASW 0.00000 0.00000

rs6076187 CLM & ESN 0.00000 0.00000

rs6076187 CLM & GWD 0.00001 0.00001

rs6076187 CLM & LWK 0.00000 0.00000

rs6076187 CLM & MSL 0.00000 0.00000

rs6076187 CLM & YRI 0.00000 0.00000

rs6076187 CLM & PUR 0.33281 0.00237

rs6076187 PEL & ACB 0.00000 0.00000

rs6076187 PEL & ASW 0.00001 0.00001

rs6076187 PEL & ESN 0.00000 0.00000

rs6076187 PEL & GWD 0.00003 0.00002

rs6076187 PEL & LWK 0.00000 0.00000

rs6076187 PEL & MSL 0.00001 0.00001

rs6076187 PEL & YRI 0.00000 0.00000

rs6076187 PEL & PUR 0.23550 0.00202

rs6076187 PEL & CLM 1.00000 0.00000

rs6076187 MXL & ACB 0.00004 0.00002

rs6076187 MXL & ASW 0.00001 0.00001

rs6076187 MXL & ESN 0.00000 0.00000

rs6076187 MXL & GWD 0.00031 0.00009

rs6076187 MXL & LWK 0.00000 0.00000

rs6076187 MXL & MSL 0.00014 0.00004

rs6076187 MXL & YRI 0.00000 0.00000

rs6076187 MXL & PUR 0.68187 0.00150

rs6076187 MXL & CLM 0.81411 0.00084

rs6076187 MXL & PEL 0.62595 0.00150

rs6076187 CHS & ACB 0.00001 0.00001

rs6076187 CHS & ASW 0.00000 0.00000

rs6076187 CHS & ESN 0.00000 0.00000

rs6076187 CHS & GWD 0.00016 0.00006

rs6076187 CHS & LWK 0.00000 0.00000

rs6076187 CHS & MSL 0.00004 0.00002

rs6076187 CHS & YRI 0.00000 0.00000

rs6076187 CHS & PUR 1.00000 0.00000

rs6076187 CHS & CLM 0.33358 0.00239

rs6076187 CHS & PEL 0.23814 0.00172

rs6076187 CHS & MXL 0.68333 0.00150

rs6076187 CDX & ACB 0.00004 0.00002

rs6076187 CDX & ASW 0.00004 0.00002

rs6076187 CDX & ESN 0.00000 0.00000

rs6076187 CDX & GWD 0.00027 0.00006

rs6076187 CDX & LWK 0.00000 0.00000

rs6076187 CDX & MSL 0.00034 0.00007

rs6076187 CDX & YRI 0.00001 0.00001

rs6076187 CDX & PUR 1.00000 0.00000

rs6076187 CDX & CLM 0.24584 0.00201

rs6076187 CDX & PEL 0.22160 0.00156

rs6076187 CDX & MXL 0.54231 0.00175

rs6076187 CDX & CHS 0.86025 0.00077

rs6076187 KHV & ACB 0.00011 0.00005

rs6076187 KHV & ASW 0.00001 0.00001

rs6076187 KHV & ESN 0.00000 0.00000

rs6076187 KHV & GWD 0.00029 0.00008

rs6076187 KHV & LWK 0.00000 0.00000

rs6076187 KHV & MSL 0.00032 0.00008

rs6076187 KHV & YRI 0.00000 0.00000

rs6076187 KHV & PUR 1.00000 0.00000

rs6076187 KHV & CLM 0.25260 0.00218

rs6076187 KHV & PEL 0.22990 0.00202

rs6076187 KHV & MXL 0.54462 0.00189

rs6076187 KHV & CHS 0.86281 0.00088

rs6076187 KHV & CDX 1.00000 0.00000

rs6076187 CHB & ACB 0.03323 0.00117

rs6076187 CHB & ASW 0.01081 0.00067

rs6076187 CHB & ESN 0.00000 0.00000

rs6076187 CHB & GWD 0.08665 0.00206

rs6076187 CHB & LWK 0.00001 0.00001

rs6076187 CHB & MSL 0.08569 0.00216

rs6076187 CHB & YRI 0.00696 0.00050

rs6076187 CHB & PUR 0.03469 0.00097

rs6076187 CHB & CLM 0.00232 0.00022

rs6076187 CHB & PEL 0.00136 0.00016

rs6076187 CHB & MXL 0.02462 0.00072

rs6076187 CHB & CHS 0.03391 0.00112

rs6076187 CHB & CDX 0.06583 0.00140

rs6076187 CHB & KHV 0.05263 0.00129

rs6076187 JPT & ACB 0.00035 0.00012

rs6076187 JPT & ASW 0.00010 0.00004

rs6076187 JPT & ESN 0.00000 0.00000

rs6076187 JPT & GWD 0.00096 0.00014

rs6076187 JPT & LWK 0.00000 0.00000

rs6076187 JPT & MSL 0.00218 0.00024

rs6076187 JPT & YRI 0.00000 0.00000

rs6076187 JPT & PUR 0.61739 0.00191

rs6076187 JPT & CLM 0.10230 0.00145

rs6076187 JPT & PEL 0.08698 0.00136

rs6076187 JPT & MXL 0.33231 0.00215

rs6076187 JPT & CHS 0.51171 0.00226

rs6076187 JPT & CDX 0.73679 0.00161

rs6076187 JPT & KHV 0.62299 0.00190

rs6076187 JPT & CHB 0.14728 0.00255

rs6076187 CEU & ACB 0.00000 0.00000

rs6076187 CEU & ASW 0.00000 0.00000

rs6076187 CEU & ESN 0.00000 0.00000

rs6076187 CEU & GWD 0.00000 0.00000

rs6076187 CEU & LWK 0.00000 0.00000

rs6076187 CEU & MSL 0.00000 0.00000

rs6076187 CEU & YRI 0.00000 0.00000

rs6076187 CEU & PUR 0.00343 0.00023

rs6076187 CEU & CLM 0.06631 0.00092

rs6076187 CEU & PEL 0.15449 0.00142

rs6076187 CEU & MXL 0.03895 0.00072

rs6076187 CEU & CHS 0.00359 0.00022

rs6076187 CEU & CDX 0.00281 0.00018

rs6076187 CEU & KHV 0.00317 0.00019

rs6076187 CEU & CHB 0.00000 0.00000

rs6076187 CEU & JPT 0.00030 0.00006

rs6076187 FIN & ACB 0.00000 0.00000

rs6076187 FIN & ASW 0.00001 0.00001

rs6076187 FIN & ESN 0.00000 0.00000

rs6076187 FIN & GWD 0.00000 0.00000

rs6076187 FIN & LWK 0.00000 0.00000

rs6076187 FIN & MSL 0.00001 0.00001

rs6076187 FIN & YRI 0.00000 0.00000

rs6076187 FIN & PUR 0.58646 0.00188

rs6076187 FIN & CLM 0.68360 0.00138

rs6076187 FIN & PEL 0.52419 0.00189

rs6076187 FIN & MXL 1.00000 0.00000

rs6076187 FIN & CHS 0.58736 0.00174

rs6076187 FIN & CDX 0.57576 0.00174

rs6076187 FIN & KHV 0.57838 0.00196

rs6076187 FIN & CHB 0.00707 0.00041

rs6076187 FIN & JPT 0.22582 0.00220

rs6076187 FIN & CEU 0.02732 0.00064

rs6076187 GBR & ACB 0.00000 0.00000

rs6076187 GBR & ASW 0.00000 0.00000

rs6076187 GBR & ESN 0.00000 0.00000

rs6076187 GBR & GWD 0.00000 0.00000

rs6076187 GBR & LWK 0.00000 0.00000

rs6076187 GBR & MSL 0.00000 0.00000

rs6076187 GBR & YRI 0.00000 0.00000

rs6076187 GBR & PUR 0.06103 0.00118

rs6076187 GBR & CLM 0.46954 0.00177

rs6076187 GBR & PEL 0.61094 0.00135

rs6076187 GBR & MXL 0.29378 0.00178

rs6076187 GBR & CHS 0.06401 0.00108

rs6076187 GBR & CDX 0.05647 0.00092

rs6076187 GBR & KHV 0.04120 0.00077

rs6076187 GBR & CHB 0.00015 0.00004

rs6076187 GBR & JPT 0.01173 0.00046

rs6076187 GBR & CEU 0.36473 0.00148

rs6076187 GBR & FIN 0.18623 0.00160

rs6076187 IBS & ACB 0.00000 0.00000

rs6076187 IBS & ASW 0.00000 0.00000

rs6076187 IBS & ESN 0.00000 0.00000

rs6076187 IBS & GWD 0.00000 0.00000

rs6076187 IBS & LWK 0.00000 0.00000

rs6076187 IBS & MSL 0.00000 0.00000

rs6076187 IBS & YRI 0.00000 0.00000

rs6076187 IBS & PUR 0.04163 0.00095

rs6076187 IBS & CLM 0.35897 0.00204

rs6076187 IBS & PEL 0.61879 0.00131

rs6076187 IBS & MXL 0.20039 0.00145

rs6076187 IBS & CHS 0.04424 0.00096

rs6076187 IBS & CDX 0.03637 0.00090

rs6076187 IBS & KHV 0.04081 0.00093

rs6076187 IBS & CHB 0.00002 0.00001

rs6076187 IBS & JPT 0.00728 0.00030

rs6076187 IBS & CEU 0.38450 0.00163

rs6076187 IBS & FIN 0.18815 0.00182

rs6076187 IBS & GBR 1.00000 0.00000

rs6076187 TSI & ACB 0.00000 0.00000

rs6076187 TSI & ASW 0.00000 0.00000

rs6076187 TSI & ESN 0.00000 0.00000

rs6076187 TSI & GWD 0.00000 0.00000

rs6076187 TSI & LWK 0.00000 0.00000

rs6076187 TSI & MSL 0.00000 0.00000

rs6076187 TSI & YRI 0.00000 0.00000

rs6076187 TSI & PUR 0.25767 0.00236

rs6076187 TSI & CLM 1.00000 0.00000

rs6076187 TSI & PEL 1.00000 0.00000

rs6076187 TSI & MXL 0.64495 0.00129

rs6076187 TSI & CHS 0.25700 0.00205

rs6076187 TSI & CDX 0.24275 0.00194

rs6076187 TSI & KHV 0.18584 0.00201

rs6076187 TSI & CHB 0.00131 0.00015

rs6076187 TSI & JPT 0.07322 0.00136

rs6076187 TSI & CEU 0.07327 0.00106

rs6076187 TSI & FIN 0.55098 0.00173

rs6076187 TSI & GBR 0.48432 0.00145

rs6076187 TSI & IBS 0.49208 0.00177

rs6076187 BEB & ACB 0.00000 0.00000

rs6076187 BEB & ASW 0.00000 0.00000

rs6076187 BEB & ESN 0.00000 0.00000

rs6076187 BEB & GWD 0.00002 0.00002

rs6076187 BEB & LWK 0.00000 0.00000

rs6076187 BEB & MSL 0.00000 0.00000

rs6076187 BEB & YRI 0.00000 0.00000

rs6076187 BEB & PUR 0.32957 0.00212

rs6076187 BEB & CLM 1.00000 0.00000

rs6076187 BEB & PEL 1.00000 0.00000

rs6076187 BEB & MXL 0.81178 0.00076

rs6076187 BEB & CHS 0.32957 0.00223

rs6076187 BEB & CDX 0.31773 0.00207

rs6076187 BEB & KHV 0.24248 0.00195

rs6076187 BEB & CHB 0.00276 0.00024

rs6076187 BEB & JPT 0.10034 0.00159

rs6076187 BEB & CEU 0.09752 0.00120

rs6076187 BEB & FIN 0.67677 0.00127

rs6076187 BEB & GBR 0.46107 0.00145

rs6076187 BEB & IBS 0.46476 0.00171

rs6076187 BEB & TSI 1.00000 0.00000

rs6076187 GIH & ACB 0.00000 0.00000

rs6076187 GIH & ASW 0.00000 0.00000

rs6076187 GIH & ESN 0.00000 0.00000

rs6076187 GIH & GWD 0.00000 0.00000

rs6076187 GIH & LWK 0.00000 0.00000

rs6076187 GIH & MSL 0.00000 0.00000

rs6076187 GIH & YRI 0.00000 0.00000

rs6076187 GIH & PUR 0.00021 0.00004

rs6076187 GIH & CLM 0.00981 0.00031

rs6076187 GIH & PEL 0.02643 0.00057

rs6076187 GIH & MXL 0.00880 0.00026

rs6076187 GIH & CHS 0.00031 0.00005

rs6076187 GIH & CDX 0.00026 0.00005

rs6076187 GIH & KHV 0.00015 0.00004

rs6076187 GIH & CHB 0.00000 0.00000

rs6076187 GIH & JPT 0.00002 0.00001

rs6076187 GIH & CEU 0.44046 0.00125

rs6076187 GIH & FIN 0.00157 0.00012

rs6076187 GIH & GBR 0.08906 0.00093

rs6076187 GIH & IBS 0.10655 0.00109

rs6076187 GIH & TSI 0.01070 0.00036

rs6076187 GIH & BEB 0.01473 0.00042

rs6076187 ITU & ACB 0.00000 0.00000

rs6076187 ITU & ASW 0.00000 0.00000

rs6076187 ITU & ESN 0.00000 0.00000

rs6076187 ITU & GWD 0.00000 0.00000

rs6076187 ITU & LWK 0.00000 0.00000

rs6076187 ITU & MSL 0.00000 0.00000

rs6076187 ITU & YRI 0.00000 0.00000

rs6076187 ITU & PUR 0.06590 0.00110

rs6076187 ITU & CLM 0.48222 0.00173

rs6076187 ITU & PEL 0.62198 0.00134

rs6076187 ITU & MXL 0.30667 0.00169

rs6076187 ITU & CHS 0.06858 0.00111

rs6076187 ITU & CDX 0.04163 0.00091

rs6076187 ITU & KHV 0.04219 0.00111

rs6076187 ITU & CHB 0.00004 0.00002

rs6076187 ITU & JPT 0.01265 0.00045

rs6076187 ITU & CEU 0.38226 0.00135

rs6076187 ITU & FIN 0.19193 0.00162

rs6076187 ITU & GBR 1.00000 0.00000

rs6076187 ITU & IBS 1.00000 0.00000

rs6076187 ITU & TSI 0.49517 0.00199

rs6076187 ITU & BEB 0.47266 0.00169

rs6076187 ITU & GIH 0.06126 0.00077

rs6076187 PJL & ACB 0.00000 0.00000

rs6076187 PJL & ASW 0.00000 0.00000

rs6076187 PJL & ESN 0.00000 0.00000

rs6076187 PJL & GWD 0.00000 0.00000

rs6076187 PJL & LWK 0.00000 0.00000

rs6076187 PJL & MSL 0.00000 0.00000

rs6076187 PJL & YRI 0.00000 0.00000

rs6076187 PJL & PUR 0.01054 0.00044

rs6076187 PJL & CLM 0.13080 0.00123

rs6076187 PJL & PEL 0.27661 0.00169

rs6076187 PJL & MXL 0.09048 0.00122

rs6076187 PJL & CHS 0.00975 0.00043

rs6076187 PJL & CDX 0.00766 0.00032

rs6076187 PJL & KHV 0.00820 0.00038

rs6076187 PJL & CHB 0.00001 0.00001

rs6076187 PJL & JPT 0.00143 0.00013

rs6076187 PJL & CEU 0.74900 0.00076

rs6076187 PJL & FIN 0.05876 0.00090

rs6076187 PJL & GBR 0.56846 0.00126

rs6076187 PJL & IBS 0.58098 0.00121

rs6076187 PJL & TSI 0.14558 0.00134

rs6076187 PJL & BEB 0.18649 0.00142

rs6076187 PJL & GIH 0.26843 0.00121

rs6076187 PJL & ITU 0.57439 0.00138

rs6076187 STU & ACB 0.00000 0.00000

rs6076187 STU & ASW 0.00000 0.00000

rs6076187 STU & ESN 0.00000 0.00000

rs6076187 STU & GWD 0.00000 0.00000

rs6076187 STU & LWK 0.00000 0.00000

rs6076187 STU & MSL 0.00005 0.00003

rs6076187 STU & YRI 0.00000 0.00000

rs6076187 STU & PUR 0.58602 0.00177

rs6076187 STU & CLM 0.83674 0.00075

rs6076187 STU & PEL 0.66545 0.00153

rs6076187 STU & MXL 1.00000 0.00000

rs6076187 STU & CHS 0.58281 0.00198

rs6076187 STU & CDX 0.45657 0.00242

rs6076187 STU & KHV 0.46729 0.00245

rs6076187 STU & CHB 0.00734 0.00045

rs6076187 STU & JPT 0.22815 0.00240

rs6076187 STU & CEU 0.02753 0.00056

rs6076187 STU & FIN 1.00000 0.00000

rs6076187 STU & GBR 0.25877 0.00193

rs6076187 STU & IBS 0.18694 0.00173

rs6076187 STU & TSI 0.68695 0.00167

rs6076187 STU & BEB 0.83255 0.00078

rs6076187 STU & GIH 0.00184 0.00015

rs6076187 STU & ITU 0.27544 0.00208

rs6076187 STU & PJL 0.05986 0.00094

Locus: rs4676496

=================================

Pop Alleles:

-----------------------------------------------------------

1 3 Total

ACB 127 65 192

ASW 70 52 122

ESN 117 81 198

GWD 122 104 226

LWK 138 60 198

MSL 98 72 170

YRI 134 82 216

PUR 129 79 208

CLM 111 77 188

PEL 94 76 170

MXL 66 62 128

CHS 133 77 210

CDX 125 61 186

KHV 137 61 198

CHB 122 84 206

JPT 142 66 208

CEU 109 89 198

FIN 104 94 198

GBR 91 91 182

IBS 101 113 214

TSI 118 96 214

BEB 112 60 172

GIH 139 67 206

ITU 131 73 204

PJL 123 69 192

STU 134 70 204

Total: 30271981 5008

Locus Population pair P-Value S.E.

----------- --------------------- ------- -------

rs4676496 ASW & ACB 0.12000 0.00272

rs4676496 ESN & ACB 0.17879 0.00332

rs4676496 ESN & ASW 0.81513 0.00180

rs4676496 GWD & ACB 0.01184 0.00093

rs4676496 GWD & ASW 0.56797 0.00296

rs4676496 GWD & ESN 0.32950 0.00426

rs4676496 LWK & ACB 0.51351 0.00332

rs4676496 LWK & ASW 0.02755 0.00115

rs4676496 LWK & ESN 0.03289 0.00135

rs4676496 LWK & GWD 0.00136 0.00023

rs4676496 MSL & ACB 0.10289 0.00219

rs4676496 MSL & ASW 1.00000 0.00000

rs4676496 MSL & ESN 0.82994 0.00177

rs4676496 MSL & GWD 0.47263 0.00404

rs4676496 MSL & LWK 0.01795 0.00109

rs4676496 YRI & ACB 0.41111 0.00435

rs4676496 YRI & ASW 0.42359 0.00391

rs4676496 YRI & ESN 0.55287 0.00355

rs4676496 YRI & GWD 0.10412 0.00311

rs4676496 YRI & LWK 0.11930 0.00282

rs4676496 YRI & MSL 0.39946 0.00373

rs4676496 PUR & ACB 0.41084 0.00396

rs4676496 PUR & ASW 0.41959 0.00373

rs4676496 PUR & ESN 0.61426 0.00348

rs4676496 PUR & GWD 0.09831 0.00264

rs4676496 PUR & LWK 0.11625 0.00221

rs4676496 PUR & MSL 0.40611 0.00420

rs4676496 PUR & YRI 1.00000 0.00000

rs4676496 CLM & ACB 0.16909 0.00266

rs4676496 CLM & ASW 0.81444 0.00164

rs4676496 CLM & ESN 1.00000 0.00000

rs4676496 CLM & GWD 0.31826 0.00355

rs4676496 CLM & LWK 0.03414 0.00140

rs4676496 CLM & MSL 0.82863 0.00176

rs4676496 CLM & YRI 0.53952 0.00305

rs4676496 CLM & PUR 0.60369 0.00322

rs4676496 PEL & ACB 0.04211 0.00157

rs4676496 PEL & ASW 0.81197 0.00156

rs4676496 PEL & ESN 0.52902 0.00385

rs4676496 PEL & GWD 0.84196 0.00159

rs4676496 PEL & LWK 0.00449 0.00039

rs4676496 PEL & MSL 0.74512 0.00242

rs4676496 PEL & YRI 0.20996 0.00370

rs4676496 PEL & PUR 0.20585 0.00348

rs4676496 PEL & CLM 0.52439 0.00370

rs4676496 MXL & ACB 0.01122 0.00071

rs4676496 MXL & ASW 0.37593 0.00356

rs4676496 MXL & ESN 0.20939 0.00304

rs4676496 MXL & GWD 0.73634 0.00261

rs4676496 MXL & LWK 0.00136 0.00020

rs4676496 MXL & MSL 0.34745 0.00365

rs4676496 MXL & YRI 0.06796 0.00201

rs4676496 MXL & PUR 0.07025 0.00194

rs4676496 MXL & CLM 0.20814 0.00340

rs4676496 MXL & PEL 0.55740 0.00261

rs4676496 CHS & ACB 0.60141 0.00314

rs4676496 CHS & ASW 0.29302 0.00344

rs4676496 CHS & ESN 0.41680 0.00399

rs4676496 CHS & GWD 0.05146 0.00184

rs4676496 CHS & LWK 0.20382 0.00356

rs4676496 CHS & MSL 0.29109 0.00362

rs4676496 CHS & YRI 0.84229 0.00188

rs4676496 CHS & PUR 0.84021 0.00176

rs4676496 CHS & CLM 0.40665 0.00403

rs4676496 CHS & PEL 0.11444 0.00274

rs4676496 CHS & MXL 0.03859 0.00151

rs4676496 CDX & ACB 0.82638 0.00172

rs4676496 CDX & ASW 0.09424 0.00214

rs4676496 CDX & ESN 0.10703 0.00233

rs4676496 CDX & GWD 0.00663 0.00065

rs4676496 CDX & LWK 0.66331 0.00247

rs4676496 CDX & MSL 0.07633 0.00205

rs4676496 CDX & YRI 0.30489 0.00347

rs4676496 CDX & PUR 0.29900 0.00400

rs4676496 CDX & CLM 0.11100 0.00291

rs4676496 CDX & PEL 0.02209 0.00122

rs4676496 CDX & MXL 0.00666 0.00057

rs4676496 CDX & CHS 0.46481 0.00378

rs4676496 KHV & ACB 0.58438 0.00344

rs4676496 KHV & ASW 0.03976 0.00145

rs4676496 KHV & ESN 0.04555 0.00190

rs4676496 KHV & GWD 0.00116 0.00019

rs4676496 KHV & LWK 1.00000 0.00000

rs4676496 KHV & MSL 0.02173 0.00102

rs4676496 KHV & YRI 0.14343 0.00302

rs4676496 KHV & PUR 0.14053 0.00282

rs4676496 KHV & CLM 0.04313 0.00155

rs4676496 KHV & PEL 0.00673 0.00055

rs4676496 KHV & MXL 0.00146 0.00025

rs4676496 KHV & CHS 0.24930 0.00363

rs4676496 KHV & CDX 0.74502 0.00247

rs4676496 CHB & ACB 0.17860 0.00316

rs4676496 CHB & ASW 0.81797 0.00153

rs4676496 CHB & ESN 1.00000 0.00000

rs4676496 CHB & GWD 0.28340 0.00396

rs4676496 CHB & LWK 0.02894 0.00136

rs4676496 CHB & MSL 0.83434 0.00193

rs4676496 CHB & YRI 0.61414 0.00334

rs4676496 CHB & PUR 0.61663 0.00340

rs4676496 CHB & CLM 1.00000 0.00000

rs4676496 CHB & PEL 0.46802 0.00396

rs4676496 CHB & MXL 0.17416 0.00310

rs4676496 CHB & CHS 0.42024 0.00431

rs4676496 CHB & CDX 0.10861 0.00261

rs4676496 CHB & KHV 0.03684 0.00172

rs4676496 JPT & ACB 0.67653 0.00280

rs4676496 JPT & ASW 0.05691 0.00176

rs4676496 JPT & ESN 0.06352 0.00216

rs4676496 JPT & GWD 0.00320 0.00036

rs4676496 JPT & LWK 0.82972 0.00157

rs4676496 JPT & MSL 0.04074 0.00166

rs4676496 JPT & YRI 0.18143 0.00360

rs4676496 JPT & PUR 0.21647 0.00351

rs4676496 JPT & CLM 0.05957 0.00196

rs4676496 JPT & PEL 0.01036 0.00074

rs4676496 JPT & MXL 0.00312 0.00043

rs4676496 JPT & CHS 0.30132 0.00357

rs4676496 JPT & CDX 0.83089 0.00161

rs4676496 JPT & KHV 0.91446 0.00095

rs4676496 JPT & CHB 0.06515 0.00211

rs4676496 CEU & ACB 0.02831 0.00132

rs4676496 CEU & ASW 0.72697 0.00259

rs4676496 CEU & ESN 0.47708 0.00371

rs4676496 CEU & GWD 0.84535 0.00161

rs4676496 CEU & LWK 0.00401 0.00048

rs4676496 CEU & MSL 0.67675 0.00336

rs4676496 CEU & YRI 0.16731 0.00334

rs4676496 CEU & PUR 0.15852 0.00328

rs4676496 CEU & CLM 0.47449 0.00379

rs4676496 CEU & PEL 1.00000 0.00000

rs4676496 CEU & MXL 0.57418 0.00340

rs4676496 CEU & CHS 0.10724 0.00308

rs4676496 CEU & CDX 0.01491 0.00096

rs4676496 CEU & KHV 0.00548 0.00049

rs4676496 CEU & CHB 0.42029 0.00354

rs4676496 CEU & JPT 0.00820 0.00074

rs4676496 FIN & ACB 0.00636 0.00057

rs4676496 FIN & ASW 0.41471 0.00358

rs4676496 FIN & ESN 0.22530 0.00355

rs4676496 FIN & GWD 0.76683 0.00217

rs4676496 FIN & LWK 0.00071 0.00017

rs4676496 FIN & MSL 0.34753 0.00409

rs4676496 FIN & YRI 0.05821 0.00205

rs4676496 FIN & PUR 0.05692 0.00233

rs4676496 FIN & CLM 0.21368 0.00352

rs4676496 FIN & PEL 0.60228 0.00363

rs4676496 FIN & MXL 0.90947 0.00096

rs4676496 FIN & CHS 0.02581 0.00134

rs4676496 FIN & CDX 0.00374 0.00039

rs4676496 FIN & KHV 0.00068 0.00012

rs4676496 FIN & CHB 0.19664 0.00402

rs4676496 FIN & JPT 0.00176 0.00026

rs4676496 FIN & CEU 0.68782 0.00307

rs4676496 GBR & ACB 0.00122 0.00019

rs4676496 GBR & ASW 0.23733 0.00345

rs4676496 GBR & ESN 0.07708 0.00232

rs4676496 GBR & GWD 0.43051 0.00350

rs4676496 GBR & LWK 0.00012 0.00005

rs4676496 GBR & MSL 0.16440 0.00266

rs4676496 GBR & YRI 0.01814 0.00103

rs4676496 GBR & PUR 0.02224 0.00138

rs4676496 GBR & CLM 0.09149 0.00268

rs4676496 GBR & PEL 0.33518 0.00384

rs4676496 GBR & MXL 0.81462 0.00156

rs4676496 GBR & CHS 0.01060 0.00074

rs4676496 GBR & CDX 0.00110 0.00017

rs4676496 GBR & KHV 0.00025 0.00007

rs4676496 GBR & CHB 0.08181 0.00227

rs4676496 GBR & JPT 0.00014 0.00007

rs4676496 GBR & CEU 0.35479 0.00405

rs4676496 GBR & FIN 0.68282 0.00296

rs4676496 IBS & ACB 0.00007 0.00003

rs4676496 IBS & ASW 0.09176 0.00207

rs4676496 IBS & ESN 0.01812 0.00121

rs4676496 IBS & GWD 0.18243 0.00354

rs4676496 IBS & LWK 0.00000 0.00000

rs4676496 IBS & MSL 0.05131 0.00186

rs4676496 IBS & YRI 0.00183 0.00024

rs4676496 IBS & PUR 0.00218 0.00034

rs4676496 IBS & CLM 0.02237 0.00111

rs4676496 IBS & PEL 0.12155 0.00287

rs4676496 IBS & MXL 0.50388 0.00332

rs4676496 IBS & CHS 0.00063 0.00013

rs4676496 IBS & CDX 0.00014 0.00009

rs4676496 IBS & KHV 0.00001 0.00001

rs4676496 IBS & CHB 0.01371 0.00079

rs4676496 IBS & JPT 0.00000 0.00000

rs4676496 IBS & CEU 0.11135 0.00309

rs4676496 IBS & FIN 0.32040 0.00344

rs4676496 IBS & GBR 0.61735 0.00313

rs4676496 TSI & ACB 0.02689 0.00124

rs4676496 TSI & ASW 0.73311 0.00237

rs4676496 TSI & ESN 0.42435 0.00466

rs4676496 TSI & GWD 0.84764 0.00184

rs4676496 TSI & LWK 0.00313 0.00035

rs4676496 TSI & MSL 0.67943 0.00274

rs4676496 TSI & YRI 0.16800 0.00326

rs4676496 TSI & PUR 0.16603 0.00352

rs4676496 TSI & CLM 0.47735 0.00391

rs4676496 TSI & PEL 1.00000 0.00000

rs4676496 TSI & MXL 0.57740 0.00295

rs4676496 TSI & CHS 0.09074 0.00293

rs4676496 TSI & CDX 0.01399 0.00095

rs4676496 TSI & KHV 0.00486 0.00052

rs4676496 TSI & CHB 0.43160 0.00422

rs4676496 TSI & JPT 0.00622 0.00061

rs4676496 TSI & CEU 1.00000 0.00000

rs4676496 TSI & FIN 0.62559 0.00312

rs4676496 TSI & GBR 0.31444 0.00354

rs4676496 TSI & IBS 0.12323 0.00300

rs4676496 BEB & ACB 0.91380 0.00083

rs4676496 BEB & ASW 0.18170 0.00274

rs4676496 BEB & ESN 0.23109 0.00340

rs4676496 BEB & GWD 0.02993 0.00158

rs4676496 BEB & LWK 0.36681 0.00374

rs4676496 BEB & MSL 0.18896 0.00344

rs4676496 BEB & YRI 0.59777 0.00335

rs4676496 BEB & PUR 0.59258 0.00295

rs4676496 BEB & CLM 0.27584 0.00374

rs4676496 BEB & PEL 0.07920 0.00220

rs4676496 BEB & MXL 0.02372 0.00110

rs4676496 BEB & CHS 0.75152 0.00243

rs4676496 BEB & CDX 0.73736 0.00233

rs4676496 BEB & KHV 0.43048 0.00345

rs4676496 BEB & CHB 0.24723 0.00355

rs4676496 BEB & JPT 0.58642 0.00336

rs4676496 BEB & CEU 0.05609 0.00208

rs4676496 BEB & FIN 0.01397 0.00088

rs4676496 BEB & GBR 0.00485 0.00050

rs4676496 BEB & IBS 0.00035 0.00011

rs4676496 BEB & TSI 0.04780 0.00171

rs4676496 GIH & ACB 0.83484 0.00170

rs4676496 GIH & ASW 0.07612 0.00192

rs4676496 GIH & ESN 0.09361 0.00258

rs4676496 GIH & GWD 0.00564 0.00053

rs4676496 GIH & LWK 0.66975 0.00344

rs4676496 GIH & MSL 0.05293 0.00173

rs4676496 GIH & YRI 0.26197 0.00367

rs4676496 GIH & PUR 0.26125 0.00346

rs4676496 GIH & CLM 0.09455 0.00263

rs4676496 GIH & PEL 0.01852 0.00106

rs4676496 GIH & MXL 0.00547 0.00049

rs4676496 GIH & CHS 0.41482 0.00427

rs4676496 GIH & CDX 1.00000 0.00000

rs4676496 GIH & KHV 0.75007 0.00224

rs4676496 GIH & CHB 0.10215 0.00255

rs4676496 GIH & JPT 0.91626 0.00096

rs4676496 GIH & CEU 0.01081 0.00078

rs4676496 GIH & FIN 0.00228 0.00033

rs4676496 GIH & GBR 0.00058 0.00017

rs4676496 GIH & IBS 0.00000 0.00000

rs4676496 GIH & TSI 0.01225 0.00087

rs4676496 GIH & BEB 0.65977 0.00272

rs4676496 ITU & ACB 0.75272 0.00236

rs4676496 ITU & ASW 0.23176 0.00300

rs4676496 ITU & ESN 0.30101 0.00412

rs4676496 ITU & GWD 0.04201 0.00198

rs4676496 ITU & LWK 0.24729 0.00326

rs4676496 ITU & MSL 0.20309 0.00351

rs4676496 ITU & YRI 0.68336 0.00328

rs4676496 ITU & PUR 0.67689 0.00283

rs4676496 ITU & CLM 0.29535 0.00406

rs4676496 ITU & PEL 0.08794 0.00252

rs4676496 ITU & MXL 0.02909 0.00128

rs4676496 ITU & CHS 0.91962 0.00095

rs4676496 ITU & CDX 0.59432 0.00304

rs4676496 ITU & KHV 0.28985 0.00337

rs4676496 ITU & CHB 0.31216 0.00390

rs4676496 ITU & JPT 0.40285 0.00368

rs4676496 ITU & CEU 0.06938 0.00214

rs4676496 ITU & FIN 0.02009 0.00105

rs4676496 ITU & GBR 0.00544 0.00050

rs4676496 ITU & IBS 0.00074 0.00016

rs4676496 ITU & TSI 0.07383 0.00216

rs4676496 ITU & BEB 0.91486 0.00075

rs4676496 ITU & GIH 0.53246 0.00365

rs4676496 PJL & ACB 0.74804 0.00259

rs4676496 PJL & ASW 0.28218 0.00329

rs4676496 PJL & ESN 0.34788 0.00447

rs4676496 PJL & GWD 0.04472 0.00185

rs4676496 PJL & LWK 0.28031 0.00347

rs4676496 PJL & MSL 0.23876 0.00361

rs4676496 PJL & YRI 0.67954 0.00282

rs4676496 PJL & PUR 0.68536 0.00252

rs4676496 PJL & CLM 0.34063 0.00329

rs4676496 PJL & PEL 0.10573 0.00261

rs4676496 PJL & MXL 0.02993 0.00124

rs4676496 PJL & CHS 0.91786 0.00088

rs4676496 PJL & CDX 0.58649 0.00363

rs4676496 PJL & KHV 0.33137 0.00362

rs4676496 PJL & CHB 0.35868 0.00412

rs4676496 PJL & JPT 0.40313 0.00419

rs4676496 PJL & CEU 0.08301 0.00271

rs4676496 PJL & FIN 0.02523 0.00121

rs4676496 PJL & GBR 0.00598 0.00047

rs4676496 PJL & IBS 0.00063 0.00015

rs4676496 PJL & TSI 0.06996 0.00226

rs4676496 PJL & BEB 0.91347 0.00078

rs4676496 PJL & GIH 0.53000 0.00401

rs4676496 PJL & ITU 1.00000 0.00000

rs4676496 STU & ACB 1.00000 0.00000

rs4676496 STU & ASW 0.15287 0.00285

rs4676496 STU & ESN 0.18836 0.00357

rs4676496 STU & GWD 0.01343 0.00106

rs4676496 STU & LWK 0.39991 0.00338

rs4676496 STU & MSL 0.13320 0.00304

rs4676496 STU & YRI 0.48014 0.00354

rs4676496 STU & PUR 0.47098 0.00401

rs4676496 STU & CLM 0.20867 0.00313

rs4676496 STU & PEL 0.04426 0.00171

rs4676496 STU & MXL 0.01076 0.00082

rs4676496 STU & CHS 0.67817 0.00289

rs4676496 STU & CDX 0.83141 0.00170

rs4676496 STU & KHV 0.45810 0.00314

rs4676496 STU & CHB 0.18406 0.00351

rs4676496 STU & JPT 0.59338 0.00320

rs4676496 STU & CEU 0.03287 0.00157

rs4676496 STU & FIN 0.00761 0.00064

rs4676496 STU & GBR 0.00183 0.00032

rs4676496 STU & IBS 0.00016 0.00006

rs4676496 STU & TSI 0.02985 0.00144

rs4676496 STU & BEB 0.91387 0.00091

rs4676496 STU & GIH 0.75500 0.00203

rs4676496 STU & ITU 0.83560 0.00170

rs4676496 STU & PJL 0.75332 0.00241

Locus: rs517339

=================================

Pop Alleles:

-----------------------------------------------------------

2 4 Total

ACB 101 91 192

ASW 73 49 122

ESN 89 109 198

GWD 91 135 226

LWK 108 90 198

MSL 84 86 170

YRI 94 122 216

PUR 65 143 208

CLM 48 140 188

PEL 45 125 170

MXL 43 85 128

CHS 21 189 210

CDX 17 169 186

KHV 25 173 198

CHB 27 179 206

JPT 25 183 208

CEU 80 118 198

FIN 76 122 198

GBR 75 107 182

IBS 93 121 214

TSI 65 149 214

BEB 44 128 172

GIH 65 141 206

ITU 54 150 204

PJL 57 135 192

STU 54 150 204

Total: 16193389 5008

Locus Population pair P-Value S.E.

----------- --------------------- ------- -------

rs517339 ASW & ACB 0.24534 0.00364

rs517339 ESN & ACB 0.15113 0.00307

rs517339 ESN & ASW 0.01142 0.00080

rs517339 GWD & ACB 0.01303 0.00111

rs517339 GWD & ASW 0.00079 0.00020

rs517339 GWD & ESN 0.37313 0.00429

rs517339 LWK & ACB 0.76085 0.00248

rs517339 LWK & ASW 0.42128 0.00324

rs517339 LWK & ESN 0.06992 0.00196

rs517339 LWK & GWD 0.00295 0.00036

rs517339 MSL & ACB 0.59877 0.00294

rs517339 MSL & ASW 0.09337 0.00219

rs517339 MSL & ESN 0.40678 0.00373

rs517339 MSL & GWD 0.08279 0.00278

rs517339 MSL & LWK 0.33822 0.00371

rs517339 YRI & ACB 0.07556 0.00221

rs517339 YRI & ASW 0.00488 0.00049

rs517339 YRI & ESN 0.84291 0.00179

rs517339 YRI & GWD 0.50326 0.00365

rs517339 YRI & LWK 0.02674 0.00119

rs517339 YRI & MSL 0.26769 0.00421

rs517339 PUR & ACB 0.00001 0.00001

rs517339 PUR & ASW 0.00000 0.00000

rs517339 PUR & ESN 0.00563 0.00046

rs517339 PUR & GWD 0.05791 0.00229

rs517339 PUR & LWK 0.00000 0.00000

rs517339 PUR & MSL 0.00062 0.00018

rs517339 PUR & YRI 0.00815 0.00063

rs517339 CLM & ACB 0.00000 0.00000

rs517339 CLM & ASW 0.00000 0.00000

rs517339 CLM & ESN 0.00027 0.00012

rs517339 CLM & GWD 0.00166 0.00027

rs517339 CLM & LWK 0.00000 0.00000

rs517339 CLM & MSL 0.00000 0.00000

rs517339 CLM & YRI 0.00008 0.00003

rs517339 CLM & PUR 0.21583 0.00344

rs517339 PEL & ACB 0.00000 0.00000

rs517339 PEL & ASW 0.00000 0.00000

rs517339 PEL & ESN 0.00023 0.00007

rs517339 PEL & GWD 0.00477 0.00050

rs517339 PEL & LWK 0.00000 0.00000

rs517339 PEL & MSL 0.00000 0.00000

rs517339 PEL & YRI 0.00070 0.00015

rs517339 PEL & PUR 0.35763 0.00385

rs517339 PEL & CLM 0.90461 0.00096

rs517339 MXL & ACB 0.00098 0.00018

rs517339 MXL & ASW 0.00007 0.00003

rs517339 MXL & ESN 0.04928 0.00144

rs517339 MXL & GWD 0.24817 0.00298

rs517339 MXL & LWK 0.00027 0.00009

rs517339 MXL & MSL 0.00694 0.00053

rs517339 MXL & YRI 0.06905 0.00188

rs517339 MXL & PUR 0.72234 0.00224

rs517339 MXL & CLM 0.12764 0.00233

rs517339 MXL & PEL 0.19697 0.00269

rs517339 CHS & ACB 0.00000 0.00000

rs517339 CHS & ASW 0.00000 0.00000

rs517339 CHS & ESN 0.00000 0.00000

rs517339 CHS & GWD 0.00000 0.00000

rs517339 CHS & LWK 0.00000 0.00000

rs517339 CHS & MSL 0.00000 0.00000

rs517339 CHS & YRI 0.00000 0.00000

rs517339 CHS & PUR 0.00000 0.00000

rs517339 CHS & CLM 0.00003 0.00001

rs517339 CHS & PEL 0.00001 0.00001

rs517339 CHS & MXL 0.00000 0.00000

rs517339 CDX & ACB 0.00000 0.00000

rs517339 CDX & ASW 0.00000 0.00000

rs517339 CDX & ESN 0.00000 0.00000

rs517339 CDX & GWD 0.00000 0.00000

rs517339 CDX & LWK 0.00000 0.00000

rs517339 CDX & MSL 0.00000 0.00000

rs517339 CDX & YRI 0.00000 0.00000

rs517339 CDX & PUR 0.00000 0.00000

rs517339 CDX & CLM 0.00003 0.00002

rs517339 CDX & PEL 0.00002 0.00001

rs517339 CDX & MXL 0.00000 0.00000

rs517339 CDX & CHS 0.86570 0.00080

rs517339 KHV & ACB 0.00000 0.00000

rs517339 KHV & ASW 0.00000 0.00000

rs517339 KHV & ESN 0.00000 0.00000

rs517339 KHV & GWD 0.00000 0.00000

rs517339 KHV & LWK 0.00000 0.00000

rs517339 KHV & MSL 0.00000 0.00000

rs517339 KHV & YRI 0.00000 0.00000

rs517339 KHV & PUR 0.00000 0.00000

rs517339 KHV & CLM 0.00156 0.00017

rs517339 KHV & PEL 0.00085 0.00014

rs517339 KHV & MXL 0.00001 0.00001

rs517339 KHV & CHS 0.43610 0.00248

rs517339 KHV & CDX 0.33118 0.00240

rs517339 CHB & ACB 0.00000 0.00000

rs517339 CHB & ASW 0.00000 0.00000

rs517339 CHB & ESN 0.00000 0.00000

rs517339 CHB & GWD 0.00000 0.00000

rs517339 CHB & LWK 0.00000 0.00000

rs517339 CHB & MSL 0.00000 0.00000

rs517339 CHB & YRI 0.00000 0.00000

rs517339 CHB & PUR 0.00000 0.00000

rs517339 CHB & CLM 0.00178 0.00020

rs517339 CHB & PEL 0.00148 0.00025

rs517339 CHB & MXL 0.00003 0.00002

rs517339 CHB & CHS 0.36324 0.00248

rs517339 CHB & CDX 0.26535 0.00293

rs517339 CHB & KHV 1.00000 0.00000

rs517339 JPT & ACB 0.00000 0.00000

rs517339 JPT & ASW 0.00000 0.00000

rs517339 JPT & ESN 0.00000 0.00000

rs517339 JPT & GWD 0.00000 0.00000

rs517339 JPT & LWK 0.00000 0.00000

rs517339 JPT & MSL 0.00000 0.00000

rs517339 JPT & YRI 0.00000 0.00000

rs517339 JPT & PUR 0.00000 0.00000

rs517339 JPT & CLM 0.00058 0.00014

rs517339 JPT & PEL 0.00050 0.00012

rs517339 JPT & MXL 0.00000 0.00000

rs517339 JPT & CHS 0.53073 0.00262

rs517339 JPT & CDX 0.41256 0.00233

rs517339 JPT & KHV 0.88176 0.00077

rs517339 JPT & CHB 0.76761 0.00169

rs517339 CEU & ACB 0.01741 0.00106

rs517339 CEU & ASW 0.00076 0.00017

rs517339 CEU & ESN 0.41610 0.00342

rs517339 CEU & GWD 1.00000 0.00000

rs517339 CEU & LWK 0.00625 0.00056

rs517339 CEU & MSL 0.09175 0.00263

rs517339 CEU & YRI 0.55375 0.00382

rs517339 CEU & PUR 0.05897 0.00173

rs517339 CEU & CLM 0.00219 0.00027

rs517339 CEU & PEL 0.00535 0.00049

rs517339 CEU & MXL 0.24271 0.00313

rs517339 CEU & CHS 0.00000 0.00000

rs517339 CEU & CDX 0.00000 0.00000

rs517339 CEU & KHV 0.00000 0.00000

rs517339 CEU & CHB 0.00000 0.00000

rs517339 CEU & JPT 0.00000 0.00000

rs517339 FIN & ACB 0.00515 0.00047

rs517339 FIN & ASW 0.00013 0.00006

rs517339 FIN & ESN 0.22500 0.00338

rs517339 FIN & GWD 0.76688 0.00235

rs517339 FIN & LWK 0.00159 0.00026

rs517339 FIN & MSL 0.03333 0.00137

rs517339 FIN & YRI 0.31752 0.00407

rs517339 FIN & PUR 0.14649 0.00322

rs517339 FIN & CLM 0.00838 0.00063

rs517339 FIN & PEL 0.01816 0.00102

rs517339 FIN & MXL 0.40720 0.00338

rs517339 FIN & CHS 0.00000 0.00000

rs517339 FIN & CDX 0.00000 0.00000

rs517339 FIN & KHV 0.00000 0.00000

rs517339 FIN & CHB 0.00000 0.00000

rs517339 FIN & JPT 0.00000 0.00000

rs517339 FIN & CEU 0.76283 0.00236

rs517339 GBR & ACB 0.03182 0.00162

rs517339 GBR & ASW 0.00190 0.00028

rs517339 GBR & ESN 0.46713 0.00390

rs517339 GBR & GWD 0.91872 0.00094

rs517339 GBR & LWK 0.01003 0.00071

rs517339 GBR & MSL 0.13023 0.00292

rs517339 GBR & YRI 0.68705 0.00305

rs517339 GBR & PUR 0.04389 0.00164

rs517339 GBR & CLM 0.00145 0.00018

rs517339 GBR & PEL 0.00524 0.00051

rs517339 GBR & MXL 0.19130 0.00292

rs517339 GBR & CHS 0.00000 0.00000

rs517339 GBR & CDX 0.00000 0.00000

rs517339 GBR & KHV 0.00000 0.00000

rs517339 GBR & CHB 0.00000 0.00000

rs517339 GBR & JPT 0.00000 0.00000

rs517339 GBR & CEU 0.91666 0.00094

rs517339 GBR & FIN 0.60674 0.00339

rs517339 IBS & ACB 0.07020 0.00232

rs517339 IBS & ASW 0.00444 0.00048

rs517339 IBS & ESN 0.76191 0.00242

rs517339 IBS & GWD 0.49952 0.00378

rs517339 IBS & LWK 0.03275 0.00160

rs517339 IBS & MSL 0.25777 0.00348

rs517339 IBS & YRI 1.00000 0.00000

rs517339 IBS & PUR 0.01293 0.00089

rs517339 IBS & CLM 0.00025 0.00010

rs517339 IBS & PEL 0.00059 0.00013

rs517339 IBS & MXL 0.08638 0.00235

rs517339 IBS & CHS 0.00000 0.00000

rs517339 IBS & CDX 0.00000 0.00000

rs517339 IBS & KHV 0.00000 0.00000

rs517339 IBS & CHB 0.00000 0.00000

rs517339 IBS & JPT 0.00000 0.00000

rs517339 IBS & CEU 0.55062 0.00315

rs517339 IBS & FIN 0.31289 0.00414

rs517339 IBS & GBR 0.68396 0.00291

rs517339 TSI & ACB 0.00002 0.00002

rs517339 TSI & ASW 0.00000 0.00000

rs517339 TSI & ESN 0.00356 0.00044

rs517339 TSI & GWD 0.03808 0.00163

rs517339 TSI & LWK 0.00000 0.00000

rs517339 TSI & MSL 0.00026 0.00011

rs517339 TSI & YRI 0.00590 0.00057

rs517339 TSI & PUR 0.91680 0.00097

rs517339 TSI & CLM 0.31843 0.00356

rs517339 TSI & PEL 0.43276 0.00354

rs517339 TSI & MXL 0.55475 0.00311

rs517339 TSI & CHS 0.00000 0.00000

rs517339 TSI & CDX 0.00000 0.00000

rs517339 TSI & KHV 0.00001 0.00001

rs517339 TSI & CHB 0.00002 0.00001

rs517339 TSI & JPT 0.00000 0.00000

rs517339 TSI & CEU 0.03874 0.00153

rs517339 TSI & FIN 0.10105 0.00294

rs517339 TSI & GBR 0.02949 0.00141

rs517339 TSI & IBS 0.00649 0.00064

rs517339 BEB & ACB 0.00000 0.00000

rs517339 BEB & ASW 0.00000 0.00000

rs517339 BEB & ESN 0.00010 0.00005

rs517339 BEB & GWD 0.00233 0.00026

rs517339 BEB & LWK 0.00000 0.00000

rs517339 BEB & MSL 0.00001 0.00001

rs517339 BEB & YRI 0.00037 0.00010

rs517339 BEB & PUR 0.25397 0.00325

rs517339 BEB & CLM 1.00000 0.00000

rs517339 BEB & PEL 0.90250 0.00085

rs517339 BEB & MXL 0.15203 0.00268

rs517339 BEB & CHS 0.00008 0.00004

rs517339 BEB & CDX 0.00004 0.00002

rs517339 BEB & KHV 0.00175 0.00025

rs517339 BEB & CHB 0.00229 0.00026

rs517339 BEB & JPT 0.00074 0.00017

rs517339 BEB & CEU 0.00287 0.00030

rs517339 BEB & FIN 0.01094 0.00077

rs517339 BEB & GBR 0.00243 0.00030

rs517339 BEB & IBS 0.00026 0.00007

rs517339 BEB & TSI 0.31181 0.00352

rs517339 GIH & ACB 0.00003 0.00002

rs517339 GIH & ASW 0.00001 0.00000

rs517339 GIH & ESN 0.00514 0.00052

rs517339 GIH & GWD 0.06921 0.00217

rs517339 GIH & LWK 0.00000 0.00000

rs517339 GIH & MSL 0.00033 0.00009

rs517339 GIH & YRI 0.01029 0.00087

rs517339 GIH & PUR 1.00000 0.00000

rs517339 GIH & CLM 0.21822 0.00317

rs517339 GIH & PEL 0.30560 0.00326

rs517339 GIH & MXL 0.72101 0.00207

rs517339 GIH & CHS 0.00000 0.00000

rs517339 GIH & CDX 0.00000 0.00000

rs517339 GIH & KHV 0.00000 0.00000

rs517339 GIH & CHB 0.00000 0.00000

rs517339 GIH & JPT 0.00000 0.00000

rs517339 GIH & CEU 0.07990 0.00224

rs517339 GIH & FIN 0.17200 0.00333

rs517339 GIH & GBR 0.05905 0.00191

rs517339 GIH & IBS 0.01346 0.00079

rs517339 GIH & TSI 0.83345 0.00170

rs517339 GIH & BEB 0.21441 0.00316

rs517339 ITU & ACB 0.00000 0.00000

rs517339 ITU & ASW 0.00000 0.00000

rs517339 ITU & ESN 0.00011 0.00004

rs517339 ITU & GWD 0.00258 0.00040

rs517339 ITU & LWK 0.00000 0.00000

rs517339 ITU & MSL 0.00002 0.00001

rs517339 ITU & YRI 0.00029 0.00011

rs517339 ITU & PUR 0.32666 0.00361

rs517339 ITU & CLM 0.90920 0.00086

rs517339 ITU & PEL 1.00000 0.00000

rs517339 ITU & MXL 0.17451 0.00302

rs517339 ITU & CHS 0.00002 0.00002

rs517339 ITU & CDX 0.00000 0.00000

rs517339 ITU & KHV 0.00062 0.00015

rs517339 ITU & CHB 0.00066 0.00016

rs517339 ITU & JPT 0.00012 0.00004

rs517339 ITU & CEU 0.00459 0.00049

rs517339 ITU & FIN 0.01479 0.00087

rs517339 ITU & GBR 0.00246 0.00033

rs517339 ITU & IBS 0.00019 0.00007

rs517339 ITU & TSI 0.38853 0.00367

rs517339 ITU & BEB 0.90503 0.00093

rs517339 ITU & GIH 0.27165 0.00382

rs517339 PJL & ACB 0.00002 0.00002

rs517339 PJL & ASW 0.00000 0.00000

rs517339 PJL & ESN 0.00249 0.00038

rs517339 PJL & GWD 0.02401 0.00116

rs517339 PJL & LWK 0.00000 0.00000

rs517339 PJL & MSL 0.00016 0.00008

rs517339 PJL & YRI 0.00359 0.00042

rs517339 PJL & PUR 0.74279 0.00253

rs517339 PJL & CLM 0.42367 0.00339

rs517339 PJL & PEL 0.56611 0.00334

rs517339 PJL & MXL 0.53595 0.00316

rs517339 PJL & CHS 0.00000 0.00000

rs517339 PJL & CDX 0.00000 0.00000

rs517339 PJL & KHV 0.00001 0.00001

rs517339 PJL & CHB 0.00009 0.00004

rs517339 PJL & JPT 0.00002 0.00001

rs517339 PJL & CEU 0.03405 0.00154

rs517339 PJL & FIN 0.08513 0.00217

rs517339 PJL & GBR 0.02400 0.00113

rs517339 PJL & IBS 0.00550 0.00051

rs517339 PJL & TSI 0.91403 0.00100

rs517339 PJL & BEB 0.41124 0.00374

rs517339 PJL & GIH 0.74497 0.00238

rs517339 PJL & ITU 0.50183 0.00322

rs517339 STU & ACB 0.00000 0.00000

rs517339 STU & ASW 0.00000 0.00000

rs517339 STU & ESN 0.00007 0.00004

rs517339 STU & GWD 0.00331 0.00038

rs517339 STU & LWK 0.00000 0.00000

rs517339 STU & MSL 0.00000 0.00000

rs517339 STU & YRI 0.00043 0.00016

rs517339 STU & PUR 0.33481 0.00366

rs517339 STU & CLM 0.90873 0.00089

rs517339 STU & PEL 1.00000 0.00000

rs517339 STU & MXL 0.17132 0.00263

rs517339 STU & CHS 0.00003 0.00003

rs517339 STU & CDX 0.00000 0.00000

rs517339 STU & KHV 0.00083 0.00016

rs517339 STU & CHB 0.00071 0.00014

rs517339 STU & JPT 0.00027 0.00007

rs517339 STU & CEU 0.00380 0.00043

rs517339 STU & FIN 0.01316 0.00099

rs517339 STU & GBR 0.00226 0.00027

rs517339 STU & IBS 0.00019 0.00006

rs517339 STU & TSI 0.38353 0.00400

rs517339 STU & BEB 0.90597 0.00089

rs517339 STU & GIH 0.27770 0.00369

rs517339 STU & ITU 1.00000 0.00000

rs517339 STU & PJL 0.50090 0.00357

Locus: rs12554036

=================================

Pop Alleles:

-----------------------------------------------------------

3 4 Total

ACB 171 21 192

ASW 113 9 122

ESN 184 14 198

GWD 195 31 226

LWK 186 12 198

MSL 156 14 170

YRI 207 9 216

PUR 166 42 208

CLM 150 38 188

PEL 161 9 170

MXL 121 7 128

CHS 195 15 210

CDX 179 7 186

KHV 188 10 198

CHB 191 15 206

JPT 200 8 208

CEU 147 51 198

FIN 170 28 198

GBR 135 47 182

IBS 165 49 214

TSI 158 56 214

BEB 140 32 172

GIH 177 29 206

ITU 176 28 204

PJL 148 44 192

STU 161 43 204

Total: 4340668 5008

Locus Population pair P-Value S.E.

----------- --------------------- ------- -------

rs12554036 ASW & ACB 0.33111 0.00206

rs12554036 ESN & ACB 0.21628 0.00213

rs12554036 ESN & ASW 1.00000 0.00000

rs12554036 GWD & ACB 0.45905 0.00305

rs12554036 GWD & ASW 0.08149 0.00141

rs12554036 GWD & ESN 0.02826 0.00095

rs12554036 LWK & ACB 0.09952 0.00166

rs12554036 LWK & ASW 0.81883 0.00076

rs12554036 LWK & ESN 0.83859 0.00087

rs12554036 LWK & GWD 0.00965 0.00050

rs12554036 MSL & ACB 0.47605 0.00214

rs12554036 MSL & ASW 0.82993 0.00082

rs12554036 MSL & ESN 0.69918 0.00151

rs12554036 MSL & GWD 0.11115 0.00190

rs12554036 MSL & LWK 0.54237 0.00192

rs12554036 YRI & ACB 0.01299 0.00052

rs12554036 YRI & ASW 0.31634 0.00187

rs12554036 YRI & ESN 0.28319 0.00192

rs12554036 YRI & GWD 0.00043 0.00008

rs12554036 YRI & LWK 0.50492 0.00185

rs12554036 YRI & MSL 0.12716 0.00147

rs12554036 PUR & ACB 0.01558 0.00080

rs12554036 PUR & ASW 0.00146 0.00017

rs12554036 PUR & ESN 0.00025 0.00006

rs12554036 PUR & GWD 0.09714 0.00207

rs12554036 PUR & LWK 0.00004 0.00003

rs12554036 PUR & MSL 0.00112 0.00015

rs12554036 PUR & YRI 0.00000 0.00000

rs12554036 CLM & ACB 0.01668 0.00075

rs12554036 CLM & ASW 0.00176 0.00019

rs12554036 CLM & ESN 0.00021 0.00006

rs12554036 CLM & GWD 0.08417 0.00187

rs12554036 CLM & LWK 0.00007 0.00004

rs12554036 CLM & MSL 0.00172 0.00020

rs12554036 CLM & YRI 0.00000 0.00000

rs12554036 CLM & PUR 1.00000 0.00000

rs12554036 PEL & ACB 0.05784 0.00132

rs12554036 PEL & ASW 0.62417 0.00116

rs12554036 PEL & ESN 0.52308 0.00201

rs12554036 PEL & GWD 0.00723 0.00043

rs12554036 PEL & LWK 0.82362 0.00088

rs12554036 PEL & MSL 0.38902 0.00228

rs12554036 PEL & YRI 0.63380 0.00144

rs12554036 PEL & PUR 0.00003 0.00002

rs12554036 PEL & CLM 0.00004 0.00002

rs12554036 MXL & ACB 0.10710 0.00151

rs12554036 MXL & ASW 0.60964 0.00155

rs12554036 MXL & ESN 0.64728 0.00145

rs12554036 MXL & GWD 0.01911 0.00081

rs12554036 MXL & LWK 1.00000 0.00000

rs12554036 MXL & MSL 0.37290 0.00197

rs12554036 MXL & YRI 0.60415 0.00154

rs12554036 MXL & PUR 0.00013 0.00004

rs12554036 MXL & CLM 0.00010 0.00004

rs12554036 MXL & PEL 1.00000 0.00000

rs12554036 CHS & ACB 0.22318 0.00228

rs12554036 CHS & ASW 1.00000 0.00000

rs12554036 CHS & ESN 1.00000 0.00000

rs12554036 CHS & GWD 0.02852 0.00096

rs12554036 CHS & LWK 0.69396 0.00165

rs12554036 CHS & MSL 0.70347 0.00146

rs12554036 CHS & YRI 0.21007 0.00173

rs12554036 CHS & PUR 0.00015 0.00009

rs12554036 CHS & CLM 0.00026 0.00007

rs12554036 CHS & PEL 0.52868 0.00176

rs12554036 CHS & MXL 0.65282 0.00163

rs12554036 CDX & ACB 0.01036 0.00047

rs12554036 CDX & ASW 0.19519 0.00162

rs12554036 CDX & ESN 0.17779 0.00159

rs12554036 CDX & GWD 0.00036 0.00006

rs12554036 CDX & LWK 0.35172 0.00191

rs12554036 CDX & MSL 0.11566 0.00145

rs12554036 CDX & YRI 1.00000 0.00000

rs12554036 CDX & PUR 0.00000 0.00000

rs12554036 CDX & CLM 0.00000 0.00000

rs12554036 CDX & PEL 0.60978 0.00163

rs12554036 CDX & MXL 0.57949 0.00143

rs12554036 CDX & CHS 0.18821 0.00159

rs12554036 KHV & ACB 0.03936 0.00097

rs12554036 KHV & ASW 0.46719 0.00170

rs12554036 KHV & ESN 0.52778 0.00177

rs12554036 KHV & GWD 0.00278 0.00024

rs12554036 KHV & LWK 0.82721 0.00087

rs12554036 KHV & MSL 0.28828 0.00169

rs12554036 KHV & YRI 0.81580 0.00088

rs12554036 KHV & PUR 0.00000 0.00000

rs12554036 KHV & CLM 0.00000 0.00000

rs12554036 KHV & PEL 1.00000 0.00000

rs12554036 KHV & MXL 1.00000 0.00000

rs12554036 KHV & CHS 0.41796 0.00201

rs12554036 KHV & CDX 0.62417 0.00120

rs12554036 CHB & ACB 0.22152 0.00233

rs12554036 CHB & ASW 1.00000 0.00000

rs12554036 CHB & ESN 1.00000 0.00000

rs12554036 CHB & GWD 0.04139 0.00119

rs12554036 CHB & LWK 0.69286 0.00144

rs12554036 CHB & MSL 0.84657 0.00084

rs12554036 CHB & YRI 0.20823 0.00192

rs12554036 CHB & PUR 0.00011 0.00003

rs12554036 CHB & CLM 0.00020 0.00005

rs12554036 CHB & PEL 0.52800 0.00196

rs12554036 CHB & MXL 0.65136 0.00140

rs12554036 CHB & CHS 1.00000 0.00000

rs12554036 CHB & CDX 0.18553 0.00181

rs12554036 CHB & KHV 0.40981 0.00212

rs12554036 JPT & ACB 0.00714 0.00037

rs12554036 JPT & ASW 0.20138 0.00156

rs12554036 JPT & ESN 0.18865 0.00178

rs12554036 JPT & GWD 0.00030 0.00006

rs12554036 JPT & LWK 0.35745 0.00171

rs12554036 JPT & MSL 0.07808 0.00133

rs12554036 JPT & YRI 1.00000 0.00000

rs12554036 JPT & PUR 0.00000 0.00000

rs12554036 JPT & CLM 0.00000 0.00000

rs12554036 JPT & PEL 0.61961 0.00130

rs12554036 JPT & MXL 0.58772 0.00147

rs12554036 JPT & CHS 0.19792 0.00169

rs12554036 JPT & CDX 1.00000 0.00000

rs12554036 JPT & KHV 0.63299 0.00143

rs12554036 JPT & CHB 0.13971 0.00153

rs12554036 CEU & ACB 0.00024 0.00008

rs12554036 CEU & ASW 0.00002 0.00001

rs12554036 CEU & ESN 0.00000 0.00000

rs12554036 CEU & GWD 0.00206 0.00026

rs12554036 CEU & LWK 0.00000 0.00000

rs12554036 CEU & MSL 0.00002 0.00001

rs12554036 CEU & YRI 0.00000 0.00000

rs12554036 CEU & PUR 0.19228 0.00345

rs12554036 CEU & CLM 0.23079 0.00336

rs12554036 CEU & PEL 0.00000 0.00000

rs12554036 CEU & MXL 0.00000 0.00000

rs12554036 CEU & CHS 0.00000 0.00000

rs12554036 CEU & CDX 0.00000 0.00000

rs12554036 CEU & KHV 0.00000 0.00000

rs12554036 CEU & CHB 0.00000 0.00000

rs12554036 CEU & JPT 0.00000 0.00000

rs12554036 FIN & ACB 0.36368 0.00267

rs12554036 FIN & ASW 0.07398 0.00156

rs12554036 FIN & ESN 0.03228 0.00095

rs12554036 FIN & GWD 1.00000 0.00000

rs12554036 FIN & LWK 0.01189 0.00059

rs12554036 FIN & MSL 0.10118 0.00189

rs12554036 FIN & YRI 0.00031 0.00006

rs12554036 FIN & PUR 0.11118 0.00230

rs12554036 FIN & CLM 0.14106 0.00226

rs12554036 FIN & PEL 0.00513 0.00035

rs12554036 FIN & MXL 0.01640 0.00060

rs12554036 FIN & CHS 0.02208 0.00081

rs12554036 FIN & CDX 0.00026 0.00004

rs12554036 FIN & KHV 0.00327 0.00024

rs12554036 FIN & CHB 0.03439 0.00101

rs12554036 FIN & JPT 0.00034 0.00007

rs12554036 FIN & CEU 0.00554 0.00042

rs12554036 GBR & ACB 0.00034 0.00009

rs12554036 GBR & ASW 0.00007 0.00003

rs12554036 GBR & ESN 0.00000 0.00000

rs12554036 GBR & GWD 0.00267 0.00037

rs12554036 GBR & LWK 0.00000 0.00000

rs12554036 GBR & MSL 0.00001 0.00001

rs12554036 GBR & YRI 0.00000 0.00000

rs12554036 GBR & PUR 0.23035 0.00309

rs12554036 GBR & CLM 0.22041 0.00283

rs12554036 GBR & PEL 0.00000 0.00000

rs12554036 GBR & MXL 0.00000 0.00000

rs12554036 GBR & CHS 0.00000 0.00000

rs12554036 GBR & CDX 0.00000 0.00000

rs12554036 GBR & KHV 0.00000 0.00000

rs12554036 GBR & CHB 0.00000 0.00000

rs12554036 GBR & JPT 0.00000 0.00000

rs12554036 GBR & CEU 1.00000 0.00000

rs12554036 GBR & FIN 0.00460 0.00038

rs12554036 IBS & ACB 0.00151 0.00020

rs12554036 IBS & ASW 0.00020 0.00006

rs12554036 IBS & ESN 0.00002 0.00001

rs12554036 IBS & GWD 0.01300 0.00075

rs12554036 IBS & LWK 0.00000 0.00000

rs12554036 IBS & MSL 0.00006 0.00002

rs12554036 IBS & YRI 0.00000 0.00000

rs12554036 IBS & PUR 0.55894 0.00278

rs12554036 IBS & CLM 0.54444 0.00303

rs12554036 IBS & PEL 0.00000 0.00000

rs12554036 IBS & MXL 0.00001 0.00001

rs12554036 IBS & CHS 0.00000 0.00000

rs12554036 IBS & CDX 0.00000 0.00000

rs12554036 IBS & KHV 0.00000 0.00000

rs12554036 IBS & CHB 0.00001 0.00001

rs12554036 IBS & JPT 0.00000 0.00000

rs12554036 IBS & CEU 0.56607 0.00281

rs12554036 IBS & FIN 0.02495 0.00096

rs12554036 IBS & GBR 0.55277 0.00302

rs12554036 TSI & ACB 0.00003 0.00001

rs12554036 TSI & ASW 0.00000 0.00000

rs12554036 TSI & ESN 0.00000 0.00000

rs12554036 TSI & GWD 0.00116 0.00021

rs12554036 TSI & LWK 0.00000 0.00000

rs12554036 TSI & MSL 0.00000 0.00000

rs12554036 TSI & YRI 0.00000 0.00000

rs12554036 TSI & PUR 0.16386 0.00274

rs12554036 TSI & CLM 0.19480 0.00301

rs12554036 TSI & PEL 0.00000 0.00000

rs12554036 TSI & MXL 0.00000 0.00000

rs12554036 TSI & CHS 0.00000 0.00000

rs12554036 TSI & CDX 0.00000 0.00000

rs12554036 TSI & KHV 0.00000 0.00000

rs12554036 TSI & CHB 0.00000 0.00000

rs12554036 TSI & JPT 0.00000 0.00000

rs12554036 TSI & CEU 1.00000 0.00000

rs12554036 TSI & FIN 0.00286 0.00031

rs12554036 TSI & GBR 1.00000 0.00000

rs12554036 TSI & IBS 0.49803 0.00358

rs12554036 BEB & ACB 0.05332 0.00143

rs12554036 BEB & ASW 0.00633 0.00039

rs12554036 BEB & ESN 0.00083 0.00014

rs12554036 BEB & GWD 0.21527 0.00262

rs12554036 BEB & LWK 0.00017 0.00004

rs12554036 BEB & MSL 0.00615 0.00041

rs12554036 BEB & YRI 0.00001 0.00001

rs12554036 BEB & PUR 0.79189 0.00137

rs12554036 BEB & CLM 0.78987 0.00162

rs12554036 BEB & PEL 0.00019 0.00004

rs12554036 BEB & MXL 0.00081 0.00012

rs12554036 BEB & CHS 0.00060 0.00009

rs12554036 BEB & CDX 0.00000 0.00000

rs12554036 BEB & KHV 0.00005 0.00002

rs12554036 BEB & CHB 0.00158 0.00017

rs12554036 BEB & JPT 0.00000 0.00000

rs12554036 BEB & CEU 0.10170 0.00244

rs12554036 BEB & FIN 0.25915 0.00285

rs12554036 BEB & GBR 0.12316 0.00224

rs12554036 BEB & IBS 0.31163 0.00361

rs12554036 BEB & TSI 0.08724 0.00202

rs12554036 GIH & ACB 0.36309 0.00295

rs12554036 GIH & ASW 0.07628 0.00138

rs12554036 GIH & ESN 0.02270 0.00083

rs12554036 GIH & GWD 1.00000 0.00000

rs12554036 GIH & LWK 0.00796 0.00044

rs12554036 GIH & MSL 0.10522 0.00173

rs12554036 GIH & YRI 0.00058 0.00010

rs12554036 GIH & PUR 0.11799 0.00269

rs12554036 GIH & CLM 0.10597 0.00237

rs12554036 GIH & PEL 0.00541 0.00034

rs12554036 GIH & MXL 0.01199 0.00053

rs12554036 GIH & CHS 0.02517 0.00089

rs12554036 GIH & CDX 0.00035 0.00007

rs12554036 GIH & KHV 0.00266 0.00025

rs12554036 GIH & CHB 0.03608 0.00105

rs12554036 GIH & JPT 0.00021 0.00005

rs12554036 GIH & CEU 0.00402 0.00038

rs12554036 GIH & FIN 1.00000 0.00000

rs12554036 GIH & GBR 0.00438 0.00040

rs12554036 GIH & IBS 0.02251 0.00097

rs12554036 GIH & TSI 0.00207 0.00026

rs12554036 GIH & BEB 0.26537 0.00290

rs12554036 ITU & ACB 0.44265 0.00278

rs12554036 ITU & ASW 0.10544 0.00174

rs12554036 ITU & ESN 0.03363 0.00095

rs12554036 ITU & GWD 1.00000 0.00000

rs12554036 ITU & LWK 0.01246 0.00064

rs12554036 ITU & MSL 0.10504 0.00189

rs12554036 ITU & YRI 0.00084 0.00013

rs12554036 ITU & PUR 0.08844 0.00207

rs12554036 ITU & CLM 0.10601 0.00214

rs12554036 ITU & PEL 0.00744 0.00032

rs12554036 ITU & MXL 0.01778 0.00056

rs12554036 ITU & CHS 0.03545 0.00108

rs12554036 ITU & CDX 0.00057 0.00009

rs12554036 ITU & KHV 0.00307 0.00022

rs12554036 ITU & CHB 0.03978 0.00126

rs12554036 ITU & JPT 0.00039 0.00008

rs12554036 ITU & CEU 0.00274 0.00030

rs12554036 ITU & FIN 1.00000 0.00000

rs12554036 ITU & GBR 0.00281 0.00035

rs12554036 ITU & IBS 0.01689 0.00084

rs12554036 ITU & TSI 0.00145 0.00021

rs12554036 ITU & BEB 0.20466 0.00281

rs12554036 ITU & GIH 1.00000 0.00000

rs12554036 PJL & ACB 0.00284 0.00028

rs12554036 PJL & ASW 0.00027 0.00006

rs12554036 PJL & ESN 0.00000 0.00000

rs12554036 PJL & GWD 0.02167 0.00087

rs12554036 PJL & LWK 0.00001 0.00001

rs12554036 PJL & MSL 0.00013 0.00004

rs12554036 PJL & YRI 0.00000 0.00000

rs12554036 PJL & PUR 0.54596 0.00317

rs12554036 PJL & CLM 0.53403 0.00320

rs12554036 PJL & PEL 0.00000 0.00000

rs12554036 PJL & MXL 0.00003 0.00002

rs12554036 PJL & CHS 0.00002 0.00002

rs12554036 PJL & CDX 0.00000 0.00000

rs12554036 PJL & KHV 0.00000 0.00000

rs12554036 PJL & CHB 0.00002 0.00001

rs12554036 PJL & JPT 0.00000 0.00000

rs12554036 PJL & CEU 0.55466 0.00320

rs12554036 PJL & FIN 0.02696 0.00116

rs12554036 PJL & GBR 0.54142 0.00322

rs12554036 PJL & IBS 1.00000 0.00000

rs12554036 PJL & TSI 0.49073 0.00352

rs12554036 PJL & BEB 0.36779 0.00322

rs12554036 PJL & GIH 0.02816 0.00119

rs12554036 PJL & ITU 0.01765 0.00073

rs12554036 STU & ACB 0.00581 0.00041

rs12554036 STU & ASW 0.00077 0.00009

rs12554036 STU & ESN 0.00002 0.00001

rs12554036 STU & GWD 0.05438 0.00154

rs12554036 STU & LWK 0.00000 0.00000

rs12554036 STU & MSL 0.00042 0.00008

rs12554036 STU & YRI 0.00000 0.00000

rs12554036 STU & PUR 0.90372 0.00093

rs12554036 STU & CLM 0.90037 0.00082

rs12554036 STU & PEL 0.00000 0.00000

rs12554036 STU & MXL 0.00006 0.00003

rs12554036 STU & CHS 0.00009 0.00004

rs12554036 STU & CDX 0.00000 0.00000

rs12554036 STU & KHV 0.00000 0.00000

rs12554036 STU & CHB 0.00013 0.00008

rs12554036 STU & JPT 0.00000 0.00000

rs12554036 STU & CEU 0.28750 0.00314

rs12554036 STU & FIN 0.08772 0.00185

rs12554036 STU & GBR 0.28230 0.00358

rs12554036 STU & IBS 0.72490 0.00233

rs12554036 STU & TSI 0.25681 0.00337

rs12554036 STU & BEB 0.60439 0.00270

rs12554036 STU & GIH 0.06796 0.00162

rs12554036 STU & ITU 0.06849 0.00186

rs12554036 STU & PJL 0.71664 0.00244

Locus: rs4810992

=================================

Pop Alleles:

-----------------------------------------------------------

1 3 Total

ACB 6 186 192

ASW 0 122 122

ESN 0 198 198

GWD 1 225 226

LWK 0 198 198

MSL 3 167 170

YRI 5 211 216

PUR 36 172 208

CLM 24 164 188

PEL 24 146 170

MXL 31 97 128

CHS 19 191 210

CDX 18 168 186

KHV 13 185 198

CHB 12 194 206

JPT 14 194 208

CEU 59 139 198

FIN 38 160 198

GBR 53 129 182

IBS 80 134 214

TSI 92 122 214

BEB 20 152 172

GIH 33 173 206

ITU 24 180 204

PJL 25 167 192

STU 37 167 204

Total: 667 4341 5008

Locus Population pair P-Value S.E.

----------- --------------------- ------- -------

rs4810992 ASW & ACB 0.05224 0.00062

rs4810992 ESN & ACB 0.01341 0.00029

rs4810992 ESN & ASW No table

rs4810992 GWD & ACB 0.05128 0.00068

rs4810992 GWD & ASW 1.00000 0.00000

rs4810992 GWD & ESN 1.00000 0.00000

rs4810992 LWK & ACB 0.01330 0.00033

rs4810992 LWK & ASW No table

rs4810992 LWK & ESN No table

rs4810992 LWK & GWD 1.00000 0.00000

rs4810992 MSL & ACB 0.51048 0.00134

rs4810992 MSL & ASW 0.26698 0.00087

rs4810992 MSL & ESN 0.09705 0.00073

rs4810992 MSL & GWD 0.31776 0.00104

rs4810992 MSL & LWK 0.09823 0.00075

rs4810992 YRI & ACB 0.76197 0.00085

rs4810992 YRI & ASW 0.11170 0.00076

rs4810992 YRI & ESN 0.06154 0.00055

rs4810992 YRI & GWD 0.11551 0.00085

rs4810992 YRI & LWK 0.06153 0.00059

rs4810992 YRI & MSL 0.73921 0.00083

rs4810992 PUR & ACB 0.00000 0.00000

rs4810992 PUR & ASW 0.00000 0.00000

rs4810992 PUR & ESN 0.00000 0.00000

rs4810992 PUR & GWD 0.00000 0.00000

rs4810992 PUR & LWK 0.00000 0.00000

rs4810992 PUR & MSL 0.00000 0.00000

rs4810992 PUR & YRI 0.00000 0.00000

rs4810992 CLM & ACB 0.00056 0.00008

rs4810992 CLM & ASW 0.00001 0.00000

rs4810992 CLM & ESN 0.00000 0.00000

rs4810992 CLM & GWD 0.00000 0.00000

rs4810992 CLM & LWK 0.00000 0.00000

rs4810992 CLM & MSL 0.00004 0.00002

rs4810992 CLM & YRI 0.00008 0.00004

rs4810992 CLM & PUR 0.26563 0.00263

rs4810992 PEL & ACB 0.00015 0.00004

rs4810992 PEL & ASW 0.00000 0.00000

rs4810992 PEL & ESN 0.00000 0.00000

rs4810992 PEL & GWD 0.00000 0.00000

rs4810992 PEL & LWK 0.00000 0.00000

rs4810992 PEL & MSL 0.00005 0.00003

rs4810992 PEL & YRI 0.00000 0.00000

rs4810992 PEL & PUR 0.48107 0.00280

rs4810992 PEL & CLM 0.75519 0.00182

rs4810992 MXL & ACB 0.00000 0.00000

rs4810992 MXL & ASW 0.00000 0.00000

rs4810992 MXL & ESN 0.00000 0.00000

rs4810992 MXL & GWD 0.00000 0.00000

rs4810992 MXL & LWK 0.00000 0.00000

rs4810992 MXL & MSL 0.00000 0.00000

rs4810992 MXL & YRI 0.00000 0.00000

rs4810992 MXL & PUR 0.15753 0.00215

rs4810992 MXL & CLM 0.01155 0.00049

rs4810992 MXL & PEL 0.03540 0.00116

rs4810992 CHS & ACB 0.02181 0.00060

rs4810992 CHS & ASW 0.00014 0.00003

rs4810992 CHS & ESN 0.00000 0.00000

rs4810992 CHS & GWD 0.00001 0.00001

rs4810992 CHS & LWK 0.00000 0.00000

rs4810992 CHS & MSL 0.00186 0.00016

rs4810992 CHS & YRI 0.00293 0.00019

rs4810992 CHS & PUR 0.01421 0.00067

rs4810992 CHS & CLM 0.25625 0.00250

rs4810992 CHS & PEL 0.14398 0.00179

rs4810992 CHS & MXL 0.00022 0.00006

rs4810992 CDX & ACB 0.01065 0.00042

rs4810992 CDX & ASW 0.00011 0.00003

rs4810992 CDX & ESN 0.00000 0.00000

rs4810992 CDX & GWD 0.00001 0.00001

rs4810992 CDX & LWK 0.00000 0.00000

rs4810992 CDX & MSL 0.00122 0.00010

rs4810992 CDX & YRI 0.00208 0.00017

rs4810992 CDX & PUR 0.02947 0.00087

rs4810992 CDX & CLM 0.41554 0.00267

rs4810992 CDX & PEL 0.25029 0.00229

rs4810992 CDX & MXL 0.00083 0.00013

rs4810992 CDX & CHS 0.86421 0.00094

rs4810992 KHV & ACB 0.15738 0.00157

rs4810992 KHV & ASW 0.00153 0.00010

rs4810992 KHV & ESN 0.00021 0.00004

rs4810992 KHV & GWD 0.00044 0.00006

rs4810992 KHV & LWK 0.00022 0.00004

rs4810992 KHV & MSL 0.03664 0.00064

rs4810992 KHV & YRI 0.05102 0.00091

rs4810992 KHV & PUR 0.00099 0.00013

rs4810992 KHV & CLM 0.05776 0.00133

rs4810992 KHV & PEL 0.02186 0.00072

rs4810992 KHV & MXL 0.00002 0.00002

rs4810992 KHV & CHS 0.36301 0.00230

rs4810992 KHV & CDX 0.34567 0.00230

rs4810992 CHB & ACB 0.23183 0.00183

rs4810992 CHB & ASW 0.00352 0.00015

rs4810992 CHB & ESN 0.00039 0.00006

rs4810992 CHB & GWD 0.00114 0.00009

rs4810992 CHB & LWK 0.00042 0.00005

rs4810992 CHB & MSL 0.06186 0.00089

rs4810992 CHB & YRI 0.08329 0.00118

rs4810992 CHB & PUR 0.00019 0.00005

rs4810992 CHB & CLM 0.02268 0.00081

rs4810992 CHB & PEL 0.00768 0.00038

rs4810992 CHB & MXL 0.00000 0.00000

rs4810992 CHB & CHS 0.26443 0.00196

rs4810992 CHB & CDX 0.18287 0.00185

rs4810992 CHB & KHV 0.83814 0.00081

rs4810992 JPT & ACB 0.11221 0.00124

rs4810992 JPT & ASW 0.00145 0.00011

rs4810992 JPT & ESN 0.00007 0.00002

rs4810992 JPT & GWD 0.00023 0.00003

rs4810992 JPT & LWK 0.00012 0.00003

rs4810992 JPT & MSL 0.02431 0.00059

rs4810992 JPT & YRI 0.03327 0.00070

rs4810992 JPT & PUR 0.00118 0.00016

rs4810992 JPT & CLM 0.05943 0.00135

rs4810992 JPT & PEL 0.02405 0.00073

rs4810992 JPT & MXL 0.00005 0.00004

rs4810992 JPT & CHS 0.46765 0.00219

rs4810992 JPT & CDX 0.35178 0.00211

rs4810992 JPT & KHV 1.00000 0.00000

rs4810992 JPT & CHB 0.83994 0.00082

rs4810992 CEU & ACB 0.00000 0.00000

rs4810992 CEU & ASW 0.00000 0.00000

rs4810992 CEU & ESN 0.00000 0.00000

rs4810992 CEU & GWD 0.00000 0.00000

rs4810992 CEU & LWK 0.00000 0.00000

rs4810992 CEU & MSL 0.00000 0.00000

rs4810992 CEU & YRI 0.00000 0.00000

rs4810992 CEU & PUR 0.00280 0.00030

rs4810992 CEU & CLM 0.00009 0.00007

rs4810992 CEU & PEL 0.00054 0.00012

rs4810992 CEU & MXL 0.30863 0.00277

rs4810992 CEU & CHS 0.00000 0.00000

rs4810992 CEU & CDX 0.00000 0.00000

rs4810992 CEU & KHV 0.00000 0.00000

rs4810992 CEU & CHB 0.00000 0.00000

rs4810992 CEU & JPT 0.00000 0.00000

rs4810992 FIN & ACB 0.00000 0.00000

rs4810992 FIN & ASW 0.00000 0.00000

rs4810992 FIN & ESN 0.00000 0.00000

rs4810992 FIN & GWD 0.00000 0.00000

rs4810992 FIN & LWK 0.00000 0.00000

rs4810992 FIN & MSL 0.00000 0.00000

rs4810992 FIN & YRI 0.00000 0.00000

rs4810992 FIN & PUR 0.69912 0.00216

rs4810992 FIN & CLM 0.09728 0.00198

rs4810992 FIN & PEL 0.21915 0.00251

rs4810992 FIN & MXL 0.32821 0.00268

rs4810992 FIN & CHS 0.00468 0.00038

rs4810992 FIN & CDX 0.00863 0.00044

rs4810992 FIN & KHV 0.00022 0.00005

rs4810992 FIN & CHB 0.00004 0.00002

rs4810992 FIN & JPT 0.00020 0.00006

rs4810992 FIN & CEU 0.01915 0.00089

rs4810992 GBR & ACB 0.00000 0.00000

rs4810992 GBR & ASW 0.00000 0.00000

rs4810992 GBR & ESN 0.00000 0.00000

rs4810992 GBR & GWD 0.00000 0.00000

rs4810992 GBR & LWK 0.00000 0.00000

rs4810992 GBR & MSL 0.00000 0.00000

rs4810992 GBR & YRI 0.00000 0.00000

rs4810992 GBR & PUR 0.00714 0.00054

rs4810992 GBR & CLM 0.00018 0.00006

rs4810992 GBR & PEL 0.00064 0.00014

rs4810992 GBR & MXL 0.36966 0.00335

rs4810992 GBR & CHS 0.00000 0.00000

rs4810992 GBR & CDX 0.00000 0.00000

rs4810992 GBR & KHV 0.00000 0.00000

rs4810992 GBR & CHB 0.00000 0.00000

rs4810992 GBR & JPT 0.00000 0.00000

rs4810992 GBR & CEU 0.91008 0.00077

rs4810992 GBR & FIN 0.03008 0.00123

rs4810992 IBS & ACB 0.00000 0.00000

rs4810992 IBS & ASW 0.00000 0.00000

rs4810992 IBS & ESN 0.00000 0.00000

rs4810992 IBS & GWD 0.00000 0.00000

rs4810992 IBS & LWK 0.00000 0.00000

rs4810992 IBS & MSL 0.00000 0.00000

rs4810992 IBS & YRI 0.00000 0.00000

rs4810992 IBS & PUR 0.00000 0.00000

rs4810992 IBS & CLM 0.00000 0.00000

rs4810992 IBS & PEL 0.00000 0.00000

rs4810992 IBS & MXL 0.01248 0.00062

rs4810992 IBS & CHS 0.00000 0.00000

rs4810992 IBS & CDX 0.00000 0.00000

rs4810992 IBS & KHV 0.00000 0.00000

rs4810992 IBS & CHB 0.00000 0.00000

rs4810992 IBS & JPT 0.00000 0.00000

rs4810992 IBS & CEU 0.11164 0.00251

rs4810992 IBS & FIN 0.00002 0.00001

rs4810992 IBS & GBR 0.08626 0.00233

rs4810992 TSI & ACB 0.00000 0.00000

rs4810992 TSI & ASW 0.00000 0.00000

rs4810992 TSI & ESN 0.00000 0.00000

rs4810992 TSI & GWD 0.00000 0.00000

rs4810992 TSI & LWK 0.00000 0.00000

rs4810992 TSI & MSL 0.00000 0.00000

rs4810992 TSI & YRI 0.00000 0.00000

rs4810992 TSI & PUR 0.00000 0.00000

rs4810992 TSI & CLM 0.00000 0.00000

rs4810992 TSI & PEL 0.00000 0.00000

rs4810992 TSI & MXL 0.00037 0.00009

rs4810992 TSI & CHS 0.00000 0.00000

rs4810992 TSI & CDX 0.00000 0.00000

rs4810992 TSI & KHV 0.00000 0.00000

rs4810992 TSI & CHB 0.00000 0.00000

rs4810992 TSI & JPT 0.00000 0.00000

rs4810992 TSI & CEU 0.00585 0.00057

rs4810992 TSI & FIN 0.00000 0.00000

rs4810992 TSI & GBR 0.00407 0.00048

rs4810992 TSI & IBS 0.28168 0.00372

rs4810992 BEB & ACB 0.00196 0.00016

rs4810992 BEB & ASW 0.00003 0.00001

rs4810992 BEB & ESN 0.00000 0.00000

rs4810992 BEB & GWD 0.00000 0.00000

rs4810992 BEB & LWK 0.00000 0.00000

rs4810992 BEB & MSL 0.00036 0.00006

rs4810992 BEB & YRI 0.00028 0.00005

rs4810992 BEB & PUR 0.15096 0.00233

rs4810992 BEB & CLM 0.74983 0.00143

rs4810992 BEB & PEL 0.52500 0.00250

rs4810992 BEB & MXL 0.00502 0.00039

rs4810992 BEB & CHS 0.49673 0.00246

rs4810992 BEB & CDX 0.60774 0.00190

rs4810992 BEB & KHV 0.10003 0.00149

rs4810992 BEB & CHB 0.06325 0.00137

rs4810992 BEB & JPT 0.10727 0.00170

rs4810992 BEB & CEU 0.00001 0.00000

rs4810992 BEB & FIN 0.05968 0.00132

rs4810992 BEB & GBR 0.00005 0.00003

rs4810992 BEB & IBS 0.00000 0.00000

rs4810992 BEB & TSI 0.00000 0.00000

rs4810992 GIH & ACB 0.00001 0.00001

rs4810992 GIH & ASW 0.00000 0.00000

rs4810992 GIH & ESN 0.00000 0.00000

rs4810992 GIH & GWD 0.00000 0.00000

rs4810992 GIH & LWK 0.00000 0.00000

rs4810992 GIH & MSL 0.00000 0.00000

rs4810992 GIH & YRI 0.00000 0.00000

rs4810992 GIH & PUR 0.79351 0.00165

rs4810992 GIH & CLM 0.39369 0.00304

rs4810992 GIH & PEL 0.66091 0.00206

rs4810992 GIH & MXL 0.08344 0.00184

rs4810992 GIH & CHS 0.03639 0.00102

rs4810992 GIH & CDX 0.07012 0.00150

rs4810992 GIH & KHV 0.00298 0.00024

rs4810992 GIH & CHB 0.00132 0.00017

rs4810992 GIH & JPT 0.00308 0.00027

rs4810992 GIH & CEU 0.00125 0.00018

rs4810992 GIH & FIN 0.43043 0.00313

rs4810992 GIH & GBR 0.00184 0.00023

rs4810992 GIH & IBS 0.00000 0.00000

rs4810992 GIH & TSI 0.00000 0.00000

rs4810992 GIH & BEB 0.23366 0.00239

rs4810992 ITU & ACB 0.00142 0.00015

rs4810992 ITU & ASW 0.00000 0.00000

rs4810992 ITU & ESN 0.00000 0.00000

rs4810992 ITU & GWD 0.00000 0.00000

rs4810992 ITU & LWK 0.00000 0.00000

rs4810992 ITU & MSL 0.00012 0.00003

rs4810992 ITU & YRI 0.00022 0.00005

rs4810992 ITU & PUR 0.12593 0.00248

rs4810992 ITU & CLM 0.87822 0.00088

rs4810992 ITU & PEL 0.53799 0.00249

rs4810992 ITU & MXL 0.00348 0.00027

rs4810992 ITU & CHS 0.41767 0.00234

rs4810992 ITU & CDX 0.51850 0.00211

rs4810992 ITU & KHV 0.08478 0.00141

rs4810992 ITU & CHB 0.03674 0.00095

rs4810992 ITU & JPT 0.08682 0.00157

rs4810992 ITU & CEU 0.00000 0.00000

rs4810992 ITU & FIN 0.05373 0.00144

rs4810992 ITU & GBR 0.00005 0.00003

rs4810992 ITU & IBS 0.00000 0.00000

rs4810992 ITU & TSI 0.00000 0.00000

rs4810992 ITU & BEB 1.00000 0.00000

rs4810992 ITU & GIH 0.25561 0.00261

rs4810992 PJL & ACB 0.00048 0.00007

rs4810992 PJL & ASW 0.00000 0.00000

rs4810992 PJL & ESN 0.00000 0.00000

rs4810992 PJL & GWD 0.00000 0.00000

rs4810992 PJL & LWK 0.00000 0.00000

rs4810992 PJL & MSL 0.00002 0.00001

rs4810992 PJL & YRI 0.00003 0.00001

rs4810992 PJL & PUR 0.26942 0.00302

rs4810992 PJL & CLM 1.00000 0.00000

rs4810992 PJL & PEL 0.87717 0.00083

rs4810992 PJL & MXL 0.01537 0.00065

rs4810992 PJL & CHS 0.26088 0.00247

rs4810992 PJL & CDX 0.33307 0.00285

rs4810992 PJL & KHV 0.04203 0.00099

rs4810992 PJL & CHB 0.01530 0.00055

rs4810992 PJL & JPT 0.04181 0.00108

rs4810992 PJL & CEU 0.00015 0.00007

rs4810992 PJL & FIN 0.10563 0.00207

rs4810992 PJL & GBR 0.00002 0.00001

rs4810992 PJL & IBS 0.00000 0.00000

rs4810992 PJL & TSI 0.00000 0.00000

rs4810992 PJL & BEB 0.75063 0.00154

rs4810992 PJL & GIH 0.47753 0.00270

rs4810992 PJL & ITU 0.76168 0.00129

rs4810992 STU & ACB 0.00000 0.00000

rs4810992 STU & ASW 0.00000 0.00000

rs4810992 STU & ESN 0.00000 0.00000

rs4810992 STU & GWD 0.00000 0.00000

rs4810992 STU & LWK 0.00000 0.00000

rs4810992 STU & MSL 0.00000 0.00000

rs4810992 STU & YRI 0.00000 0.00000

rs4810992 STU & PUR 0.89854 0.00093

rs4810992 STU & CLM 0.16535 0.00260

rs4810992 STU & PEL 0.33079 0.00290

rs4810992 STU & MXL 0.20985 0.00221

rs4810992 STU & CHS 0.00959 0.00054

rs4810992 STU & CDX 0.02006 0.00079

rs4810992 STU & KHV 0.00043 0.00009

rs4810992 STU & CHB 0.00015 0.00004

rs4810992 STU & JPT 0.00049 0.00011

rs4810992 STU & CEU 0.00759 0.00061

rs4810992 STU & FIN 0.79835 0.00155

rs4810992 STU & GBR 0.01186 0.00073

rs4810992 STU & IBS 0.00002 0.00002

rs4810992 STU & TSI 0.00000 0.00000

rs4810992 STU & BEB 0.08679 0.00197

rs4810992 STU & GIH 0.60461 0.00250

rs4810992 STU & ITU 0.09609 0.00185

rs4810992 STU & PJL 0.16425 0.00234

Locus: rs229243

=================================

Pop Alleles:

-----------------------------------------------------------

1 2 Total

ACB 123 69 192

ASW 75 47 122

ESN 115 83 198

GWD 148 78 226

LWK 137 61 198

MSL 123 47 170

YRI 133 83 216

PUR 88 120 208

CLM 108 80 188

PEL 92 78 170

MXL 54 74 128

CHS 129 81 210

CDX 118 68 186

KHV 135 63 198

CHB 137 69 206

JPT 137 71 208

CEU 62 136 198

FIN 66 132 198

GBR 51 131 182

IBS 81 133 214

TSI 91 123 214

BEB 62 110 172

GIH 95 111 206

ITU 95 109 204

PJL 81 111 192

STU 82 122 204

Total: 26182390 5008

Locus Population pair P-Value S.E.

----------- --------------------- ------- -------

rs229243 ASW & ACB 0.72106 0.00237

rs229243 ESN & ACB 0.25280 0.00356

rs229243 ESN & ASW 0.56200 0.00312

rs229243 GWD & ACB 0.83890 0.00173

rs229243 GWD & ASW 0.48546 0.00326

rs229243 GWD & ESN 0.13123 0.00309

rs229243 LWK & ACB 0.33745 0.00385

rs229243 LWK & ASW 0.18113 0.00317

rs229243 LWK & ESN 0.02629 0.00123

rs229243 LWK & GWD 0.46412 0.00357

rs229243 MSL & ACB 0.11374 0.00238

rs229243 MSL & ASW 0.05757 0.00158

rs229243 MSL & ESN 0.00420 0.00041

rs229243 MSL & GWD 0.15272 0.00303

rs229243 MSL & LWK 0.57068 0.00303

rs229243 YRI & ACB 0.60706 0.00340

rs229243 YRI & ASW 1.00000 0.00000

rs229243 YRI & ESN 0.48708 0.00347

rs229243 YRI & GWD 0.42829 0.00385

rs229243 YRI & LWK 0.11905 0.00288

rs229243 YRI & MSL 0.03141 0.00145

rs229243 PUR & ACB 0.00001 0.00001

rs229243 PUR & ASW 0.00084 0.00018

rs229243 PUR & ESN 0.00192 0.00033

rs229243 PUR & GWD 0.00000 0.00000

rs229243 PUR & LWK 0.00000 0.00000

rs229243 PUR & MSL 0.00000 0.00000

rs229243 PUR & YRI 0.00009 0.00005

rs229243 CLM & ACB 0.20767 0.00313

rs229243 CLM & ASW 0.56014 0.00323

rs229243 CLM & ESN 0.91690 0.00083

rs229243 CLM & GWD 0.10193 0.00266

rs229243 CLM & LWK 0.01825 0.00097

rs229243 CLM & MSL 0.00459 0.00046

rs229243 CLM & YRI 0.40476 0.00383

rs229243 CLM & PUR 0.00362 0.00044

rs229243 PEL & ACB 0.06611 0.00173

rs229243 PEL & ASW 0.22992 0.00333

rs229243 PEL & ESN 0.45950 0.00425

rs229243 PEL & GWD 0.02380 0.00130

rs229243 PEL & LWK 0.00339 0.00042

rs229243 PEL & MSL 0.00052 0.00010

rs229243 PEL & YRI 0.14425 0.00297

rs229243 PEL & PUR 0.02294 0.00131

rs229243 PEL & CLM 0.58575 0.00322

rs229243 MXL & ACB 0.00014 0.00005

rs229243 MXL & ASW 0.00273 0.00039

rs229243 MXL & ESN 0.00634 0.00050

rs229243 MXL & GWD 0.00002 0.00001

rs229243 MXL & LWK 0.00000 0.00000

rs229243 MXL & MSL 0.00000 0.00000

rs229243 MXL & YRI 0.00055 0.00014

rs229243 MXL & PUR 1.00000 0.00000

rs229243 MXL & CLM 0.00925 0.00068

rs229243 MXL & PEL 0.04677 0.00167

rs229243 CHS & ACB 0.60780 0.00314

rs229243 CHS & ASW 1.00000 0.00000

rs229243 CHS & ESN 0.54446 0.00379

rs229243 CHS & GWD 0.43257 0.00418

rs229243 CHS & LWK 0.12071 0.00293

rs229243 CHS & MSL 0.03028 0.00122

rs229243 CHS & YRI 1.00000 0.00000

rs229243 CHS & PUR 0.00015 0.00008

rs229243 CHS & CLM 0.47438 0.00353

rs229243 CHS & PEL 0.17469 0.00298

rs229243 CHS & MXL 0.00051 0.00014

rs229243 CDX & ACB 0.91517 0.00082

rs229243 CDX & ASW 0.81161 0.00165

rs229243 CDX & ESN 0.29347 0.00389

rs229243 CDX & GWD 0.67913 0.00274

rs229243 CDX & LWK 0.23455 0.00339

rs229243 CDX & MSL 0.09268 0.00246

rs229243 CDX & YRI 0.75642 0.00243

rs229243 CDX & PUR 0.00000 0.00000

rs229243 CDX & CLM 0.24787 0.00357

rs229243 CDX & PEL 0.08581 0.00216

rs229243 CDX & MXL 0.00021 0.00009

rs229243 CDX & CHS 0.75634 0.00231

rs229243 KHV & ACB 0.40053 0.00382

rs229243 KHV & ASW 0.22478 0.00317

rs229243 KHV & ESN 0.04297 0.00166

rs229243 KHV & GWD 0.60400 0.00338

rs229243 KHV & LWK 0.91259 0.00090

rs229243 KHV & MSL 0.42269 0.00347

rs229243 KHV & YRI 0.18360 0.00329

rs229243 KHV & PUR 0.00000 0.00000

rs229243 KHV & CLM 0.03578 0.00156

rs229243 KHV & PEL 0.00742 0.00062

rs229243 KHV & MXL 0.00000 0.00000

rs229243 KHV & CHS 0.17597 0.00330

rs229243 KHV & CDX 0.33911 0.00415

rs229243 CHB & ACB 0.67383 0.00254

rs229243 CHB & ASW 0.39770 0.00315

rs229243 CHB & ESN 0.08665 0.00262

rs229243 CHB & GWD 0.83783 0.00183

rs229243 CHB & LWK 0.59068 0.00310

rs229243 CHB & MSL 0.26186 0.00368

rs229243 CHB & YRI 0.31037 0.00377

rs229243 CHB & PUR 0.00000 0.00000

rs229243 CHB & CLM 0.07877 0.00235

rs229243 CHB & PEL 0.01495 0.00102

rs229243 CHB & MXL 0.00000 0.00000

rs229243 CHB & CHS 0.30303 0.00368

rs229243 CHB & CDX 0.59571 0.00349

rs229243 CHB & KHV 0.75034 0.00253

rs229243 JPT & ACB 0.75202 0.00229

rs229243 JPT & ASW 0.47952 0.00320

rs229243 JPT & ESN 0.12520 0.00271

rs229243 JPT & GWD 1.00000 0.00000

rs229243 JPT & LWK 0.52481 0.00327

rs229243 JPT & MSL 0.18474 0.00299

rs229243 JPT & YRI 0.36027 0.00444

rs229243 JPT & PUR 0.00000 0.00000

rs229243 JPT & CLM 0.09183 0.00250

rs229243 JPT & PEL 0.02491 0.00126

rs229243 JPT & MXL 0.00000 0.00000

rs229243 JPT & CHS 0.36938 0.00410

rs229243 JPT & CDX 0.67271 0.00288

rs229243 JPT & KHV 0.66728 0.00264

rs229243 JPT & CHB 0.91689 0.00089

rs229243 CEU & ACB 0.00000 0.00000

rs229243 CEU & ASW 0.00000 0.00000

rs229243 CEU & ESN 0.00000 0.00000

rs229243 CEU & GWD 0.00000 0.00000

rs229243 CEU & LWK 0.00000 0.00000

rs229243 CEU & MSL 0.00000 0.00000

rs229243 CEU & YRI 0.00000 0.00000

rs229243 CEU & PUR 0.02402 0.00127

rs229243 CEU & CLM 0.00000 0.00000

rs229243 CEU & PEL 0.00000 0.00000

rs229243 CEU & MXL 0.05685 0.00181

rs229243 CEU & CHS 0.00000 0.00000

rs229243 CEU & CDX 0.00000 0.00000

rs229243 CEU & KHV 0.00000 0.00000

rs229243 CEU & CHB 0.00000 0.00000

rs229243 CEU & JPT 0.00000 0.00000

rs229243 FIN & ACB 0.00000 0.00000

rs229243 FIN & ASW 0.00000 0.00000

rs229243 FIN & ESN 0.00000 0.00000

rs229243 FIN & GWD 0.00000 0.00000

rs229243 FIN & LWK 0.00000 0.00000

rs229243 FIN & MSL 0.00000 0.00000

rs229243 FIN & YRI 0.00000 0.00000

rs229243 FIN & PUR 0.06831 0.00245

rs229243 FIN & CLM 0.00000 0.00000

rs229243 FIN & PEL 0.00003 0.00002

rs229243 FIN & MXL 0.12564 0.00220

rs229243 FIN & CHS 0.00000 0.00000

rs229243 FIN & CDX 0.00000 0.00000

rs229243 FIN & KHV 0.00000 0.00000

rs229243 FIN & CHB 0.00000 0.00000

rs229243 FIN & JPT 0.00000 0.00000

rs229243 FIN & CEU 0.74721 0.00248

rs229243 GBR & ACB 0.00000 0.00000

rs229243 GBR & ASW 0.00000 0.00000

rs229243 GBR & ESN 0.00000 0.00000

rs229243 GBR & GWD 0.00000 0.00000

rs229243 GBR & LWK 0.00000 0.00000

rs229243 GBR & MSL 0.00000 0.00000

rs229243 GBR & YRI 0.00000 0.00000

rs229243 GBR & PUR 0.00444 0.00043

rs229243 GBR & CLM 0.00000 0.00000

rs229243 GBR & PEL 0.00000 0.00000

rs229243 GBR & MXL 0.00982 0.00063

rs229243 GBR & CHS 0.00000 0.00000

rs229243 GBR & CDX 0.00000 0.00000

rs229243 GBR & KHV 0.00000 0.00000

rs229243 GBR & CHB 0.00000 0.00000

rs229243 GBR & JPT 0.00000 0.00000

rs229243 GBR & CEU 0.50543 0.00361

rs229243 GBR & FIN 0.26520 0.00338

rs229243 IBS & ACB 0.00000 0.00000

rs229243 IBS & ASW 0.00000 0.00000

rs229243 IBS & ESN 0.00004 0.00003

rs229243 IBS & GWD 0.00000 0.00000

rs229243 IBS & LWK 0.00000 0.00000

rs229243 IBS & MSL 0.00000 0.00000

rs229243 IBS & YRI 0.00000 0.00000

rs229243 IBS & PUR 0.37303 0.00407

rs229243 IBS & CLM 0.00009 0.00006

rs229243 IBS & PEL 0.00214 0.00030

rs229243 IBS & MXL 0.48522 0.00340

rs229243 IBS & CHS 0.00000 0.00000

rs229243 IBS & CDX 0.00000 0.00000

rs229243 IBS & KHV 0.00000 0.00000

rs229243 IBS & CHB 0.00000 0.00000

rs229243 IBS & JPT 0.00000 0.00000

rs229243 IBS & CEU 0.17907 0.00301

rs229243 IBS & FIN 0.36168 0.00413

rs229243 IBS & GBR 0.04621 0.00164

rs229243 TSI & ACB 0.00000 0.00000

rs229243 TSI & ASW 0.00110 0.00017

rs229243 TSI & ESN 0.00155 0.00025

rs229243 TSI & GWD 0.00000 0.00000

rs229243 TSI & LWK 0.00000 0.00000

rs229243 TSI & MSL 0.00000 0.00000

rs229243 TSI & YRI 0.00012 0.00007

rs229243 TSI & PUR 1.00000 0.00000

rs229243 TSI & CLM 0.00462 0.00054

rs229243 TSI & PEL 0.03110 0.00119

rs229243 TSI & MXL 1.00000 0.00000

rs229243 TSI & CHS 0.00016 0.00008

rs229243 TSI & CDX 0.00010 0.00005

rs229243 TSI & KHV 0.00000 0.00000

rs229243 TSI & CHB 0.00000 0.00000

rs229243 TSI & JPT 0.00000 0.00000

rs229243 TSI & CEU 0.01937 0.00124

rs229243 TSI & FIN 0.06897 0.00223

rs229243 TSI & GBR 0.00280 0.00033

rs229243 TSI & IBS 0.37530 0.00449

rs229243 BEB & ACB 0.00000 0.00000

rs229243 BEB & ASW 0.00001 0.00001

rs229243 BEB & ESN 0.00001 0.00001

rs229243 BEB & GWD 0.00000 0.00000

rs229243 BEB & LWK 0.00000 0.00000

rs229243 BEB & MSL 0.00000 0.00000

rs229243 BEB & YRI 0.00000 0.00000

rs229243 BEB & PUR 0.24738 0.00365

rs229243 BEB & CLM 0.00005 0.00003

rs229243 BEB & PEL 0.00072 0.00015

rs229243 BEB & MXL 0.28194 0.00331

rs229243 BEB & CHS 0.00000 0.00000

rs229243 BEB & CDX 0.00000 0.00000

rs229243 BEB & KHV 0.00000 0.00000

rs229243 BEB & CHB 0.00000 0.00000

rs229243 BEB & JPT 0.00000 0.00000

rs229243 BEB & CEU 0.37736 0.00384

rs229243 BEB & FIN 0.65805 0.00264

rs229243 BEB & GBR 0.10999 0.00265

rs229243 BEB & IBS 0.75153 0.00233

rs229243 BEB & TSI 0.21489 0.00386

rs229243 GIH & ACB 0.00035 0.00012

rs229243 GIH & ASW 0.00742 0.00058

rs229243 GIH & ESN 0.01878 0.00091

rs229243 GIH & GWD 0.00003 0.00002

rs229243 GIH & LWK 0.00000 0.00000

rs229243 GIH & MSL 0.00000 0.00000

rs229243 GIH & YRI 0.00207 0.00030

rs229243 GIH & PUR 0.49401 0.00366

rs229243 GIH & CLM 0.02591 0.00127

rs229243 GIH & PEL 0.14366 0.00343

rs229243 GIH & MXL 0.49723 0.00336

rs229243 GIH & CHS 0.00240 0.00040

rs229243 GIH & CDX 0.00080 0.00016

rs229243 GIH & KHV 0.00001 0.00001

rs229243 GIH & CHB 0.00003 0.00002

rs229243 GIH & JPT 0.00010 0.00005

rs229243 GIH & CEU 0.00308 0.00040

rs229243 GIH & FIN 0.01205 0.00086

rs229243 GIH & GBR 0.00026 0.00007

rs229243 GIH & IBS 0.09349 0.00223

rs229243 GIH & TSI 0.49373 0.00384

rs229243 GIH & BEB 0.05734 0.00209

rs229243 ITU & ACB 0.00039 0.00009

rs229243 ITU & ASW 0.01140 0.00067

rs229243 ITU & ESN 0.02023 0.00118

rs229243 ITU & GWD 0.00001 0.00001

rs229243 ITU & LWK 0.00000 0.00000

rs229243 ITU & MSL 0.00000 0.00000

rs229243 ITU & YRI 0.00264 0.00037

rs229243 ITU & PUR 0.43229 0.00435

rs229243 ITU & CLM 0.03302 0.00154

rs229243 ITU & PEL 0.17675 0.00316

rs229243 ITU & MXL 0.49624 0.00324

rs229243 ITU & CHS 0.00339 0.00042

rs229243 ITU & CDX 0.00112 0.00022

rs229243 ITU & KHV 0.00003 0.00002

rs229243 ITU & CHB 0.00008 0.00004

rs229243 ITU & JPT 0.00014 0.00008

rs229243 ITU & CEU 0.00199 0.00028

rs229243 ITU & FIN 0.00786 0.00065

rs229243 ITU & GBR 0.00009 0.00003

rs229243 ITU & IBS 0.07681 0.00298

rs229243 ITU & TSI 0.43174 0.00397

rs229243 ITU & BEB 0.04382 0.00155

rs229243 ITU & GIH 1.00000 0.00000

rs229243 PJL & ACB 0.00000 0.00000

rs229243 PJL & ASW 0.00153 0.00026

rs229243 PJL & ESN 0.00182 0.00028

rs229243 PJL & GWD 0.00000 0.00000

rs229243 PJL & LWK 0.00000 0.00000

rs229243 PJL & MSL 0.00000 0.00000

rs229243 PJL & YRI 0.00013 0.00006

rs229243 PJL & PUR 1.00000 0.00000

rs229243 PJL & CLM 0.00499 0.00056

rs229243 PJL & PEL 0.02735 0.00121

rs229243 PJL & MXL 1.00000 0.00000

rs229243 PJL & CHS 0.00012 0.00006

rs229243 PJL & CDX 0.00018 0.00010

rs229243 PJL & KHV 0.00000 0.00000

rs229243 PJL & CHB 0.00000 0.00000

rs229243 PJL & JPT 0.00000 0.00000

rs229243 PJL & CEU 0.02966 0.00131

rs229243 PJL & FIN 0.07620 0.00236

rs229243 PJL & GBR 0.00487 0.00042

rs229243 PJL & IBS 0.41679 0.00432

rs229243 PJL & TSI 1.00000 0.00000

rs229243 PJL & BEB 0.24162 0.00362

rs229243 PJL & GIH 0.47721 0.00416

rs229243 PJL & ITU 0.41559 0.00329

rs229243 STU & ACB 0.00000 0.00000

rs229243 STU & ASW 0.00015 0.00008

rs229243 STU & ESN 0.00055 0.00013

rs229243 STU & GWD 0.00000 0.00000

rs229243 STU & LWK 0.00000 0.00000

rs229243 STU & MSL 0.00000 0.00000

rs229243 STU & YRI 0.00001 0.00001

rs229243 STU & PUR 0.68672 0.00342

rs229243 STU & CLM 0.00092 0.00017

rs229243 STU & PEL 0.00868 0.00074

rs229243 STU & MXL 0.73309 0.00241

rs229243 STU & CHS 0.00000 0.00000

rs229243 STU & CDX 0.00000 0.00000

rs229243 STU & KHV 0.00000 0.00000

rs229243 STU & CHB 0.00000 0.00000

rs229243 STU & JPT 0.00000 0.00000

rs229243 STU & CEU 0.07801 0.00205

rs229243 STU & FIN 0.17515 0.00314

rs229243 STU & GBR 0.01349 0.00089

rs229243 STU & IBS 0.68495 0.00278

rs229243 STU & TSI 0.68444 0.00294

rs229243 STU & BEB 0.46347 0.00374

rs229243 STU & GIH 0.23298 0.00362

rs229243 STU & ITU 0.22718 0.00373

rs229243 STU & PJL 0.75650 0.00215

Locus: rs6809184

=================================

Pop Alleles:

-----------------------------------------------------------

2 4 Total

ACB 177 15 192

ASW 110 12 122

ESN 185 13 198

GWD 193 33 226

LWK 178 20 198

MSL 140 30 170

YRI 194 22 216

PUR 200 8 208

CLM 183 5 188

PEL 166 4 170

MXL 125 3 128

CHS 206 4 210

CDX 185 1 186

KHV 198 0 198

CHB 205 1 206

JPT 207 1 208

CEU 187 11 198

FIN 183 15 198

GBR 171 11 182

IBS 201 13 214

TSI 207 7 214

BEB 154 18 172

GIH 186 20 206

ITU 184 20 204

PJL 169 23 192

STU 188 16 204

Total: 4682326 5008

Locus Population pair P-Value S.E.

----------- --------------------- ------- -------

rs6809184 ASW & ACB 0.67904 0.00146

rs6809184 ESN & ACB 0.70041 0.00158

rs6809184 ESN & ASW 0.39350 0.00205

rs6809184 GWD & ACB 0.03241 0.00092

rs6809184 GWD & ASW 0.24375 0.00212

rs6809184 GWD & ESN 0.00783 0.00045

rs6809184 LWK & ACB 0.47806 0.00222

rs6809184 LWK & ASW 1.00000 0.00000

rs6809184 LWK & ESN 0.27566 0.00195

rs6809184 LWK & GWD 0.18463 0.00266

rs6809184 MSL & ACB 0.00628 0.00041

rs6809184 MSL & ASW 0.06784 0.00147

rs6809184 MSL & ESN 0.00126 0.00016

rs6809184 MSL & GWD 0.48939 0.00271

rs6809184 MSL & LWK 0.04620 0.00107

rs6809184 YRI & ACB 0.49476 0.00242

rs6809184 YRI & ASW 1.00000 0.00000

rs6809184 YRI & ESN 0.21920 0.00224

rs6809184 YRI & GWD 0.19403 0.00276

rs6809184 YRI & LWK 1.00000 0.00000

rs6809184 YRI & MSL 0.03642 0.00118

rs6809184 PUR & ACB 0.13091 0.00164

rs6809184 PUR & ASW 0.05286 0.00099

rs6809184 PUR & ESN 0.26520 0.00205

rs6809184 PUR & GWD 0.00006 0.00002

rs6809184 PUR & LWK 0.01843 0.00070

rs6809184 PUR & MSL 0.00003 0.00002

rs6809184 PUR & YRI 0.01269 0.00049

rs6809184 CLM & ACB 0.03733 0.00087

rs6809184 CLM & ASW 0.00910 0.00032

rs6809184 CLM & ESN 0.08979 0.00111

rs6809184 CLM & GWD 0.00001 0.00000

rs6809184 CLM & LWK 0.00345 0.00026

rs6809184 CLM & MSL 0.00000 0.00000

rs6809184 CLM & YRI 0.00265 0.00019

rs6809184 CLM & PUR 0.58205 0.00147

rs6809184 PEL & ACB 0.03120 0.00070

rs6809184 PEL & ASW 0.00814 0.00034

rs6809184 PEL & ESN 0.08026 0.00100

rs6809184 PEL & GWD 0.00002 0.00001

rs6809184 PEL & LWK 0.00261 0.00019

rs6809184 PEL & MSL 0.00000 0.00000

rs6809184 PEL & YRI 0.00208 0.00018

rs6809184 PEL & PUR 0.55841 0.00124

rs6809184 PEL & CLM 1.00000 0.00000

rs6809184 MXL & ACB 0.04753 0.00077

rs6809184 MXL & ASW 0.01549 0.00043

rs6809184 MXL & ESN 0.11490 0.00115

rs6809184 MXL & GWD 0.00018 0.00006

rs6809184 MXL & LWK 0.00764 0.00037

rs6809184 MXL & MSL 0.00002 0.00001

rs6809184 MXL & YRI 0.00499 0.00025

rs6809184 MXL & PUR 0.54268 0.00135

rs6809184 MXL & CLM 1.00000 0.00000

rs6809184 MXL & PEL 1.00000 0.00000

rs6809184 CHS & ACB 0.00758 0.00032

rs6809184 CHS & ASW 0.00214 0.00015

rs6809184 CHS & ESN 0.02443 0.00051

rs6809184 CHS & GWD 0.00000 0.00000

rs6809184 CHS & LWK 0.00054 0.00008

rs6809184 CHS & MSL 0.00000 0.00000

rs6809184 CHS & YRI 0.00038 0.00008

rs6809184 CHS & PUR 0.25825 0.00153

rs6809184 CHS & CLM 0.74107 0.00069

rs6809184 CHS & PEL 1.00000 0.00000

rs6809184 CHS & MXL 1.00000 0.00000

rs6809184 CDX & ACB 0.00042 0.00007

rs6809184 CDX & ASW 0.00006 0.00002

rs6809184 CDX & ESN 0.00156 0.00011

rs6809184 CDX & GWD 0.00000 0.00000

rs6809184 CDX & LWK 0.00001 0.00001

rs6809184 CDX & MSL 0.00000 0.00000

rs6809184 CDX & YRI 0.00001 0.00000

rs6809184 CDX & PUR 0.03992 0.00060

rs6809184 CDX & CLM 0.21501 0.00107

rs6809184 CDX & PEL 0.19762 0.00103

rs6809184 CDX & MXL 0.30741 0.00100

rs6809184 CDX & CHS 0.37675 0.00112

rs6809184 KHV & ACB 0.00001 0.00001

rs6809184 KHV & ASW 0.00000 0.00000

rs6809184 KHV & ESN 0.00017 0.00003

rs6809184 KHV & GWD 0.00000 0.00000

rs6809184 KHV & LWK 0.00000 0.00000

rs6809184 KHV & MSL 0.00000 0.00000

rs6809184 KHV & YRI 0.00000 0.00000

rs6809184 KHV & PUR 0.00751 0.00024

rs6809184 KHV & CLM 0.02671 0.00042

rs6809184 KHV & PEL 0.04442 0.00065

rs6809184 KHV & MXL 0.05924 0.00069

rs6809184 KHV & CHS 0.12496 0.00075

rs6809184 KHV & CDX 0.48479 0.00066

rs6809184 CHB & ACB 0.00015 0.00003

rs6809184 CHB & ASW 0.00004 0.00001

rs6809184 CHB & ESN 0.00064 0.00007

rs6809184 CHB & GWD 0.00000 0.00000

rs6809184 CHB & LWK 0.00000 0.00000

rs6809184 CHB & MSL 0.00000 0.00000

rs6809184 CHB & YRI 0.00000 0.00000

rs6809184 CHB & PUR 0.03583 0.00047

rs6809184 CHB & CLM 0.10782 0.00082

rs6809184 CHB & PEL 0.17917 0.00094

rs6809184 CHB & MXL 0.30215 0.00104

rs6809184 CHB & CHS 0.37221 0.00105

rs6809184 CHB & CDX 1.00000 0.00000

rs6809184 CHB & KHV 1.00000 0.00000

rs6809184 JPT & ACB 0.00015 0.00003

rs6809184 JPT & ASW 0.00005 0.00002

rs6809184 JPT & ESN 0.00070 0.00009

rs6809184 JPT & GWD 0.00000 0.00000

rs6809184 JPT & LWK 0.00001 0.00000

rs6809184 JPT & MSL 0.00000 0.00000

rs6809184 JPT & YRI 0.00002 0.00002

rs6809184 JPT & PUR 0.03760 0.00059

rs6809184 JPT & CLM 0.10593 0.00091

rs6809184 JPT & PEL 0.18028 0.00106

rs6809184 JPT & MXL 0.30261 0.00084

rs6809184 JPT & CHS 0.37237 0.00106

rs6809184 JPT & CDX 1.00000 0.00000

rs6809184 JPT & KHV 1.00000 0.00000

rs6809184 JPT & CHB 1.00000 0.00000

rs6809184 CEU & ACB 0.41617 0.00179

rs6809184 CEU & ASW 0.18321 0.00171

rs6809184 CEU & ESN 0.83337 0.00091

rs6809184 CEU & GWD 0.00213 0.00021

rs6809184 CEU & LWK 0.13256 0.00181

rs6809184 CEU & MSL 0.00033 0.00006

rs6809184 CEU & YRI 0.10204 0.00154

rs6809184 CEU & PUR 0.48280 0.00168

rs6809184 CEU & CLM 0.20204 0.00156

rs6809184 CEU & PEL 0.18443 0.00141

rs6809184 CEU & MXL 0.17993 0.00139

rs6809184 CEU & CHS 0.06563 0.00099

rs6809184 CEU & CDX 0.00571 0.00021

rs6809184 CEU & KHV 0.00080 0.00007

rs6809184 CEU & CHB 0.00267 0.00016

rs6809184 CEU & JPT 0.00273 0.00016

rs6809184 FIN & ACB 1.00000 0.00000

rs6809184 FIN & ASW 0.53660 0.00189

rs6809184 FIN & ESN 0.84602 0.00081

rs6809184 FIN & GWD 0.03117 0.00111

rs6809184 FIN & LWK 0.47753 0.00209

rs6809184 FIN & MSL 0.00422 0.00029

rs6809184 FIN & YRI 0.39747 0.00224

rs6809184 FIN & PUR 0.13043 0.00153

rs6809184 FIN & CLM 0.03708 0.00080

rs6809184 FIN & PEL 0.03208 0.00054

rs6809184 FIN & MXL 0.04757 0.00089

rs6809184 FIN & CHS 0.00867 0.00035

rs6809184 FIN & CDX 0.00043 0.00005

rs6809184 FIN & KHV 0.00004 0.00001

rs6809184 FIN & CHB 0.00019 0.00003

rs6809184 FIN & JPT 0.00023 0.00004

rs6809184 FIN & CEU 0.54356 0.00201

rs6809184 GBR & ACB 0.54597 0.00158

rs6809184 GBR & ASW 0.27045 0.00208

rs6809184 GBR & ESN 1.00000 0.00000

rs6809184 GBR & GWD 0.00591 0.00036

rs6809184 GBR & LWK 0.19307 0.00213

rs6809184 GBR & MSL 0.00081 0.00011

rs6809184 GBR & YRI 0.14533 0.00161

rs6809184 GBR & PUR 0.35357 0.00178

rs6809184 GBR & CLM 0.12920 0.00143

rs6809184 GBR & PEL 0.11495 0.00114

rs6809184 GBR & MXL 0.16789 0.00134

rs6809184 GBR & CHS 0.06021 0.00081

rs6809184 GBR & CDX 0.00267 0.00015

rs6809184 GBR & KHV 0.00021 0.00003

rs6809184 GBR & CHB 0.00191 0.00013

rs6809184 GBR & JPT 0.00178 0.00012

rs6809184 GBR & CEU 1.00000 0.00000

rs6809184 GBR & FIN 0.68588 0.00152

rs6809184 IBS & ACB 0.55706 0.00185

rs6809184 IBS & ASW 0.27841 0.00186

rs6809184 IBS & ESN 0.84545 0.00084

rs6809184 IBS & GWD 0.00532 0.00035

rs6809184 IBS & LWK 0.14694 0.00178

rs6809184 IBS & MSL 0.00054 0.00012

rs6809184 IBS & YRI 0.15617 0.00209

rs6809184 IBS & PUR 0.37245 0.00191

rs6809184 IBS & CLM 0.14711 0.00152

rs6809184 IBS & PEL 0.08615 0.00103

rs6809184 IBS & MXL 0.12474 0.00127

rs6809184 IBS & CHS 0.04530 0.00086

rs6809184 IBS & CDX 0.00234 0.00015

rs6809184 IBS & KHV 0.00025 0.00004

rs6809184 IBS & CHB 0.00170 0.00011

rs6809184 IBS & JPT 0.00171 0.00011

rs6809184 IBS & CEU 0.83728 0.00079

rs6809184 IBS & FIN 0.56183 0.00201

rs6809184 IBS & GBR 1.00000 0.00000

rs6809184 TSI & ACB 0.04930 0.00082

rs6809184 TSI & ASW 0.02440 0.00068

rs6809184 TSI & ESN 0.16737 0.00175

rs6809184 TSI & GWD 0.00002 0.00001

rs6809184 TSI & LWK 0.00804 0.00035

rs6809184 TSI & MSL 0.00000 0.00000

rs6809184 TSI & YRI 0.00580 0.00033

rs6809184 TSI & PUR 0.79875 0.00085

rs6809184 TSI & CLM 0.77664 0.00084

rs6809184 TSI & PEL 0.76143 0.00079

rs6809184 TSI & MXL 0.74931 0.00081

rs6809184 TSI & CHS 0.54399 0.00115

rs6809184 TSI & CDX 0.07305 0.00073

rs6809184 TSI & KHV 0.01554 0.00034

rs6809184 TSI & CHB 0.06837 0.00075

rs6809184 TSI & JPT 0.06888 0.00074

rs6809184 TSI & CEU 0.33591 0.00185

rs6809184 TSI & FIN 0.07643 0.00122

rs6809184 TSI & GBR 0.22663 0.00153

rs6809184 TSI & IBS 0.25099 0.00192

rs6809184 BEB & ACB 0.46409 0.00235

rs6809184 BEB & ASW 1.00000 0.00000

rs6809184 BEB & ESN 0.19526 0.00199

rs6809184 BEB & GWD 0.23076 0.00226

rs6809184 BEB & LWK 1.00000 0.00000

rs6809184 BEB & MSL 0.06278 0.00134

rs6809184 BEB & YRI 1.00000 0.00000

rs6809184 BEB & PUR 0.01336 0.00044

rs6809184 BEB & CLM 0.00392 0.00021

rs6809184 BEB & PEL 0.00346 0.00018

rs6809184 BEB & MXL 0.00585 0.00027

rs6809184 BEB & CHS 0.00050 0.00007

rs6809184 BEB & CDX 0.00002 0.00001

rs6809184 BEB & KHV 0.00000 0.00000

rs6809184 BEB & CHB 0.00001 0.00000

rs6809184 BEB & JPT 0.00002 0.00001

rs6809184 BEB & CEU 0.11848 0.00159

rs6809184 BEB & FIN 0.36465 0.00207

rs6809184 BEB & GBR 0.17543 0.00211

rs6809184 BEB & IBS 0.13124 0.00156

rs6809184 BEB & TSI 0.00634 0.00034

rs6809184 GIH & ACB 0.59406 0.00190

rs6809184 GIH & ASW 1.00000 0.00000

rs6809184 GIH & ESN 0.27858 0.00234

rs6809184 GIH & GWD 0.14192 0.00228

rs6809184 GIH & LWK 1.00000 0.00000

rs6809184 GIH & MSL 0.03155 0.00112

rs6809184 GIH & YRI 0.87276 0.00089

rs6809184 GIH & PUR 0.01896 0.00060

rs6809184 GIH & CLM 0.00649 0.00031

rs6809184 GIH & PEL 0.00500 0.00029

rs6809184 GIH & MXL 0.00785 0.00031

rs6809184 GIH & CHS 0.00058 0.00007

rs6809184 GIH & CDX 0.00003 0.00002

rs6809184 GIH & KHV 0.00000 0.00000

rs6809184 GIH & CHB 0.00001 0.00001

rs6809184 GIH & JPT 0.00001 0.00001

rs6809184 GIH & CEU 0.13497 0.00162

rs6809184 GIH & FIN 0.48448 0.00222

rs6809184 GIH & GBR 0.19203 0.00212

rs6809184 GIH & IBS 0.20697 0.00213

rs6809184 GIH & TSI 0.00930 0.00042

rs6809184 GIH & BEB 0.86390 0.00084

rs6809184 ITU & ACB 0.59796 0.00198

rs6809184 ITU & ASW 1.00000 0.00000

rs6809184 ITU & ESN 0.27651 0.00205

rs6809184 ITU & GWD 0.14563 0.00225

rs6809184 ITU & LWK 1.00000 0.00000

rs6809184 ITU & MSL 0.03325 0.00096

rs6809184 ITU & YRI 1.00000 0.00000

rs6809184 ITU & PUR 0.01909 0.00059

rs6809184 ITU & CLM 0.00343 0.00020

rs6809184 ITU & PEL 0.00304 0.00027

rs6809184 ITU & MXL 0.00825 0.00033

rs6809184 ITU & CHS 0.00058 0.00008

rs6809184 ITU & CDX 0.00003 0.00002

rs6809184 ITU & KHV 0.00000 0.00000

rs6809184 ITU & CHB 0.00001 0.00000

rs6809184 ITU & JPT 0.00000 0.00000

rs6809184 ITU & CEU 0.13573 0.00195

rs6809184 ITU & FIN 0.47829 0.00232

rs6809184 ITU & GBR 0.19199 0.00189

rs6809184 ITU & IBS 0.20388 0.00193

rs6809184 ITU & TSI 0.00858 0.00040

rs6809184 ITU & BEB 0.86442 0.00090

rs6809184 ITU & GIH 1.00000 0.00000

rs6809184 PJL & ACB 0.23053 0.00210

rs6809184 PJL & ASW 0.58837 0.00181

rs6809184 PJL & ESN 0.08093 0.00143

rs6809184 PJL & GWD 0.47182 0.00314

rs6809184 PJL & LWK 0.62925 0.00194

rs6809184 PJL & MSL 0.13767 0.00197

rs6809184 PJL & YRI 0.63530 0.00220

rs6809184 PJL & PUR 0.00251 0.00022

rs6809184 PJL & CLM 0.00059 0.00008

rs6809184 PJL & PEL 0.00040 0.00007

rs6809184 PJL & MXL 0.00168 0.00016

rs6809184 PJL & CHS 0.00010 0.00003

rs6809184 PJL & CDX 0.00000 0.00000

rs6809184 PJL & KHV 0.00000 0.00000

rs6809184 PJL & CHB 0.00000 0.00000

rs6809184 PJL & JPT 0.00000 0.00000

rs6809184 PJL & CEU 0.03039 0.00085

rs6809184 PJL & FIN 0.17047 0.00210

rs6809184 PJL & GBR 0.05207 0.00131

rs6809184 PJL & IBS 0.05495 0.00126

rs6809184 PJL & TSI 0.00107 0.00011

rs6809184 PJL & BEB 0.73950 0.00149

rs6809184 PJL & GIH 0.51465 0.00242

rs6809184 PJL & ITU 0.52186 0.00240

rs6809184 STU & ACB 1.00000 0.00000

rs6809184 STU & ASW 0.54685 0.00190

rs6809184 STU & ESN 0.70197 0.00155

rs6809184 STU & GWD 0.03416 0.00116

rs6809184 STU & LWK 0.48981 0.00246

rs6809184 STU & MSL 0.00676 0.00042

rs6809184 STU & YRI 0.49990 0.00250

rs6809184 STU & PUR 0.09417 0.00139

rs6809184 STU & CLM 0.02508 0.00061

rs6809184 STU & PEL 0.02113 0.00053

rs6809184 STU & MXL 0.04769 0.00081

rs6809184 STU & CHS 0.00570 0.00030

rs6809184 STU & CDX 0.00029 0.00005

rs6809184 STU & KHV 0.00003 0.00001

rs6809184 STU & CHB 0.00006 0.00002

rs6809184 STU & JPT 0.00012 0.00004

rs6809184 STU & CEU 0.42593 0.00209

rs6809184 STU & FIN 1.00000 0.00000

rs6809184 STU & GBR 0.55101 0.00205

rs6809184 STU & IBS 0.56600 0.00223

rs6809184 STU & TSI 0.05267 0.00101

rs6809184 STU & BEB 0.46866 0.00227

rs6809184 STU & GIH 0.60022 0.00178

rs6809184 STU & ITU 0.60545 0.00207

rs6809184 STU & PJL 0.17592 0.00193

Locus: rs6962939

=================================

Pop Alleles:

-----------------------------------------------------------

1 4 Total

ACB 28 164 192

ASW 10 112 122

ESN 28 170 198

GWD 28 198 226

LWK 33 165 198

MSL 31 139 170

YRI 30 186 216

PUR 9 199 208

CLM 6 182 188

PEL 5 165 170

MXL 3 125 128

CHS 0 210 210

CDX 0 186 186

KHV 0 198 198

CHB 2 204 206

JPT 1 207 208

CEU 8 190 198

FIN 2 196 198

GBR 5 177 182

IBS 12 202 214

TSI 7 207 214

BEB 10 162 172

GIH 5 201 206

ITU 5 199 204

PJL 7 185 192

STU 9 195 204

Total: 284 4724 5008

Locus Population pair P-Value S.E.

----------- --------------------- ------- -------

rs6962939 ASW & ACB 0.10942 0.00177

rs6962939 ESN & ACB 1.00000 0.00000

rs6962939 ESN & ASW 0.11245 0.00166

rs6962939 GWD & ACB 0.56501 0.00255

rs6962939 GWD & ASW 0.27960 0.00267

rs6962939 GWD & ESN 0.66695 0.00232

rs6962939 LWK & ACB 0.58088 0.00270

rs6962939 LWK & ASW 0.03003 0.00091

rs6962939 LWK & ESN 0.58024 0.00302

rs6962939 LWK & GWD 0.21873 0.00314

rs6962939 MSL & ACB 0.39468 0.00294

rs6962939 MSL & ASW 0.01697 0.00071

rs6962939 MSL & ESN 0.31616 0.00298

rs6962939 MSL & GWD 0.11593 0.00205

rs6962939 MSL & LWK 0.78466 0.00142

rs6962939 YRI & ACB 0.88660 0.00084

rs6962939 YRI & ASW 0.12597 0.00224

rs6962939 YRI & ESN 1.00000 0.00000

rs6962939 YRI & GWD 0.67468 0.00232

rs6962939 YRI & LWK 0.49403 0.00256

rs6962939 YRI & MSL 0.26390 0.00290

rs6962939 PUR & ACB 0.00035 0.00006

rs6962939 PUR & ASW 0.21892 0.00166

rs6962939 PUR & ESN 0.00082 0.00012

rs6962939 PUR & GWD 0.00283 0.00023

rs6962939 PUR & LWK 0.00004 0.00002

rs6962939 PUR & MSL 0.00001 0.00000

rs6962939 PUR & YRI 0.00069 0.00010

rs6962939 CLM & ACB 0.00009 0.00003

rs6962939 CLM & ASW 0.06705 0.00108

rs6962939 CLM & ESN 0.00013 0.00004

rs6962939 CLM & GWD 0.00051 0.00008

rs6962939 CLM & LWK 0.00000 0.00000

rs6962939 CLM & MSL 0.00001 0.00001

rs6962939 CLM & YRI 0.00020 0.00005

rs6962939 CLM & PUR 0.60794 0.00136

rs6962939 PEL & ACB 0.00006 0.00003

rs6962939 PEL & ASW 0.05883 0.00087

rs6962939 PEL & ESN 0.00020 0.00004

rs6962939 PEL & GWD 0.00079 0.00011

rs6962939 PEL & LWK 0.00002 0.00002

rs6962939 PEL & MSL 0.00000 0.00000

rs6962939 PEL & YRI 0.00010 0.00003

rs6962939 PEL & PUR 0.58754 0.00121

rs6962939 PEL & CLM 1.00000 0.00000

rs6962939 MXL & ACB 0.00015 0.00004

rs6962939 MXL & ASW 0.04609 0.00081

rs6962939 MXL & ESN 0.00020 0.00005

rs6962939 MXL & GWD 0.00088 0.00012

rs6962939 MXL & LWK 0.00002 0.00001

rs6962939 MXL & MSL 0.00001 0.00001

rs6962939 MXL & YRI 0.00029 0.00006

rs6962939 MXL & PUR 0.38691 0.00148

rs6962939 MXL & CLM 0.74354 0.00076

rs6962939 MXL & PEL 1.00000 0.00000

rs6962939 CHS & ACB 0.00000 0.00000

rs6962939 CHS & ASW 0.00003 0.00001

rs6962939 CHS & ESN 0.00000 0.00000

rs6962939 CHS & GWD 0.00000 0.00000

rs6962939 CHS & LWK 0.00000 0.00000

rs6962939 CHS & MSL 0.00000 0.00000

rs6962939 CHS & YRI 0.00000 0.00000

rs6962939 CHS & PUR 0.00177 0.00013

rs6962939 CHS & CLM 0.01096 0.00030

rs6962939 CHS & PEL 0.01757 0.00035

rs6962939 CHS & MXL 0.05413 0.00063

rs6962939 CDX & ACB 0.00000 0.00000

rs6962939 CDX & ASW 0.00008 0.00002

rs6962939 CDX & ESN 0.00000 0.00000

rs6962939 CDX & GWD 0.00000 0.00000

rs6962939 CDX & LWK 0.00000 0.00000

rs6962939 CDX & MSL 0.00000 0.00000

rs6962939 CDX & YRI 0.00000 0.00000

rs6962939 CDX & PUR 0.00384 0.00016

rs6962939 CDX & CLM 0.03031 0.00039

rs6962939 CDX & PEL 0.02400 0.00040

rs6962939 CDX & MXL 0.06621 0.00062

rs6962939 CDX & CHS No table

rs6962939 KHV & ACB 0.00001 0.00001

rs6962939 KHV & ASW 0.00004 0.00002

rs6962939 KHV & ESN 0.00000 0.00000

rs6962939 KHV & GWD 0.00000 0.00000

rs6962939 KHV & LWK 0.00000 0.00000

rs6962939 KHV & MSL 0.00000 0.00000

rs6962939 KHV & YRI 0.00000 0.00000

rs6962939 KHV & PUR 0.00355 0.00015

rs6962939 KHV & CLM 0.01238 0.00031

rs6962939 KHV & PEL 0.02048 0.00039

rs6962939 KHV & MXL 0.05932 0.00065

rs6962939 KHV & CHS No table

rs6962939 KHV & CDX No table

rs6962939 CHB & ACB 0.00000 0.00000

rs6962939 CHB & ASW 0.00421 0.00019

rs6962939 CHB & ESN 0.00000 0.00000

rs6962939 CHB & GWD 0.00000 0.00000

rs6962939 CHB & LWK 0.00000 0.00000

rs6962939 CHB & MSL 0.00000 0.00000

rs6962939 CHB & YRI 0.00000 0.00000

rs6962939 CHB & PUR 0.06227 0.00080

rs6962939 CHB & CLM 0.15836 0.00108

rs6962939 CHB & PEL 0.25133 0.00128

rs6962939 CHB & MXL 0.37722 0.00127

rs6962939 CHB & CHS 0.24563 0.00093

rs6962939 CHB & CDX 0.49970 0.00069

rs6962939 CHB & KHV 0.49784 0.00067

rs6962939 JPT & ACB 0.00000 0.00000

rs6962939 JPT & ASW 0.00026 0.00004

rs6962939 JPT & ESN 0.00000 0.00000

rs6962939 JPT & GWD 0.00000 0.00000

rs6962939 JPT & LWK 0.00000 0.00000

rs6962939 JPT & MSL 0.00000 0.00000

rs6962939 JPT & YRI 0.00000 0.00000

rs6962939 JPT & PUR 0.01987 0.00038

rs6962939 JPT & CLM 0.05694 0.00064

rs6962939 JPT & PEL 0.09343 0.00087

rs6962939 JPT & MXL 0.30161 0.00103

rs6962939 JPT & CHS 0.49772 0.00073

rs6962939 JPT & CDX 1.00000 0.00000

rs6962939 JPT & KHV 1.00000 0.00000

rs6962939 JPT & CHB 0.62182 0.00071

rs6962939 CEU & ACB 0.00039 0.00007

rs6962939 CEU & ASW 0.13642 0.00166

rs6962939 CEU & ESN 0.00056 0.00010

rs6962939 CEU & GWD 0.00239 0.00020

rs6962939 CEU & LWK 0.00003 0.00001

rs6962939 CEU & MSL 0.00003 0.00002

rs6962939 CEU & YRI 0.00057 0.00010

rs6962939 CEU & PUR 1.00000 0.00000

rs6962939 CEU & CLM 0.78691 0.00077

rs6962939 CEU & PEL 0.58942 0.00153

rs6962939 CEU & MXL 0.53753 0.00132

rs6962939 CEU & CHS 0.00276 0.00014

rs6962939 CEU & CDX 0.00722 0.00024

rs6962939 CEU & KHV 0.00724 0.00022

rs6962939 CEU & CHB 0.05631 0.00078

rs6962939 CEU & JPT 0.01834 0.00041

rs6962939 FIN & ACB 0.00000 0.00000

rs6962939 FIN & ASW 0.00427 0.00017

rs6962939 FIN & ESN 0.00000 0.00000

rs6962939 FIN & GWD 0.00000 0.00000

rs6962939 FIN & LWK 0.00000 0.00000

rs6962939 FIN & MSL 0.00000 0.00000

rs6962939 FIN & YRI 0.00000 0.00000

rs6962939 FIN & PUR 0.06236 0.00074

rs6962939 FIN & CLM 0.16618 0.00118

rs6962939 FIN & PEL 0.25534 0.00112

rs6962939 FIN & MXL 0.38560 0.00122

rs6962939 FIN & CHS 0.23723 0.00084

rs6962939 FIN & CDX 0.49909 0.00066

rs6962939 FIN & KHV 0.49882 0.00067

rs6962939 FIN & CHB 1.00000 0.00000

rs6962939 FIN & JPT 0.61461 0.00075

rs6962939 FIN & CEU 0.10381 0.00104

rs6962939 GBR & ACB 0.00006 0.00004

rs6962939 GBR & ASW 0.05597 0.00096

rs6962939 GBR & ESN 0.00013 0.00004

rs6962939 GBR & GWD 0.00032 0.00005

rs6962939 GBR & LWK 0.00000 0.00000

rs6962939 GBR & MSL 0.00001 0.00000

rs6962939 GBR & YRI 0.00005 0.00003

rs6962939 GBR & PUR 0.42958 0.00147

rs6962939 GBR & CLM 1.00000 0.00000

rs6962939 GBR & PEL 1.00000 0.00000

rs6962939 GBR & MXL 1.00000 0.00000

rs6962939 GBR & CHS 0.02117 0.00040

rs6962939 GBR & CDX 0.02876 0.00046

rs6962939 GBR & KHV 0.02468 0.00040

rs6962939 GBR & CHB 0.26129 0.00134

rs6962939 GBR & JPT 0.10182 0.00096

rs6962939 GBR & CEU 0.57886 0.00135

rs6962939 GBR & FIN 0.26617 0.00132

rs6962939 IBS & ACB 0.00245 0.00022

rs6962939 IBS & ASW 0.49276 0.00168

rs6962939 IBS & ESN 0.00450 0.00029

rs6962939 IBS & GWD 0.01895 0.00069

rs6962939 IBS & LWK 0.00038 0.00007

rs6962939 IBS & MSL 0.00016 0.00005

rs6962939 IBS & YRI 0.00615 0.00040

rs6962939 IBS & PUR 0.65671 0.00147

rs6962939 IBS & CLM 0.33301 0.00194

rs6962939 IBS & PEL 0.22561 0.00185

rs6962939 IBS & MXL 0.18173 0.00130

rs6962939 IBS & CHS 0.00045 0.00006

rs6962939 IBS & CDX 0.00056 0.00007

rs6962939 IBS & KHV 0.00046 0.00006

rs6962939 IBS & CHB 0.01251 0.00039

rs6962939 IBS & JPT 0.00316 0.00016

rs6962939 IBS & CEU 0.49682 0.00174

rs6962939 IBS & FIN 0.01191 0.00037

rs6962939 IBS & GBR 0.21463 0.00137

rs6962939 TSI & ACB 0.00008 0.00003

rs6962939 TSI & ASW 0.06808 0.00093

rs6962939 TSI & ESN 0.00002 0.00001

rs6962939 TSI & GWD 0.00035 0.00006

rs6962939 TSI & LWK 0.00000 0.00000

rs6962939 TSI & MSL 0.00000 0.00000

rs6962939 TSI & YRI 0.00012 0.00004

rs6962939 TSI & PUR 0.61841 0.00137

rs6962939 TSI & CLM 1.00000 0.00000

rs6962939 TSI & PEL 1.00000 0.00000

rs6962939 TSI & MXL 0.74957 0.00079

rs6962939 TSI & CHS 0.01467 0.00031

rs6962939 TSI & CDX 0.01600 0.00034

rs6962939 TSI & KHV 0.01578 0.00033

rs6962939 TSI & CHB 0.17702 0.00106

rs6962939 TSI & JPT 0.06767 0.00083

rs6962939 TSI & CEU 0.79528 0.00078

rs6962939 TSI & FIN 0.17722 0.00122

rs6962939 TSI & GBR 0.77998 0.00074

rs6962939 TSI & IBS 0.34915 0.00195

rs6962939 BEB & ACB 0.00648 0.00040

rs6962939 BEB & ASW 0.48601 0.00166

rs6962939 BEB & ESN 0.00973 0.00049

rs6962939 BEB & GWD 0.02686 0.00094

rs6962939 BEB & LWK 0.00094 0.00014

rs6962939 BEB & MSL 0.00042 0.00008

rs6962939 BEB & YRI 0.01184 0.00048

rs6962939 BEB & PUR 0.63541 0.00148

rs6962939 BEB & CLM 0.30648 0.00163

rs6962939 BEB & PEL 0.28860 0.00163

rs6962939 BEB & MXL 0.16809 0.00144

rs6962939 BEB & CHS 0.00035 0.00006

rs6962939 BEB & CDX 0.00067 0.00007

rs6962939 BEB & KHV 0.00044 0.00006

rs6962939 BEB & CHB 0.01496 0.00039

rs6962939 BEB & JPT 0.00283 0.00016

rs6962939 BEB & CEU 0.47743 0.00177

rs6962939 BEB & FIN 0.01487 0.00037

rs6962939 BEB & GBR 0.19093 0.00145

rs6962939 BEB & IBS 1.00000 0.00000

rs6962939 BEB & TSI 0.32006 0.00170

rs6962939 GIH & ACB 0.00001 0.00001

rs6962939 GIH & ASW 0.02603 0.00065

rs6962939 GIH & ESN 0.00001 0.00001

rs6962939 GIH & GWD 0.00016 0.00006

rs6962939 GIH & LWK 0.00000 0.00000

rs6962939 GIH & MSL 0.00000 0.00000

rs6962939 GIH & YRI 0.00001 0.00001

rs6962939 GIH & PUR 0.41574 0.00161

rs6962939 GIH & CLM 0.76446 0.00076

rs6962939 GIH & PEL 1.00000 0.00000

rs6962939 GIH & MXL 1.00000 0.00000

rs6962939 GIH & CHS 0.02930 0.00044

rs6962939 GIH & CDX 0.06209 0.00061

rs6962939 GIH & KHV 0.06102 0.00065

rs6962939 GIH & CHB 0.45120 0.00114

rs6962939 GIH & JPT 0.12010 0.00087

rs6962939 GIH & CEU 0.40759 0.00158

rs6962939 GIH & FIN 0.44949 0.00134

rs6962939 GIH & GBR 1.00000 0.00000

rs6962939 GIH & IBS 0.13689 0.00134

rs6962939 GIH & TSI 0.77182 0.00087

rs6962939 GIH & BEB 0.11507 0.00122

rs6962939 ITU & ACB 0.00000 0.00000

rs6962939 ITU & ASW 0.02681 0.00054

rs6962939 ITU & ESN 0.00004 0.00003

rs6962939 ITU & GWD 0.00010 0.00003

rs6962939 ITU & LWK 0.00000 0.00000

rs6962939 ITU & MSL 0.00000 0.00000

rs6962939 ITU & YRI 0.00002 0.00001

rs6962939 ITU & PUR 0.41874 0.00153

rs6962939 ITU & CLM 0.76313 0.00078

rs6962939 ITU & PEL 1.00000 0.00000

rs6962939 ITU & MXL 1.00000 0.00000

rs6962939 ITU & CHS 0.02820 0.00044

rs6962939 ITU & CDX 0.06215 0.00059

rs6962939 ITU & KHV 0.06062 0.00055

rs6962939 ITU & CHB 0.28559 0.00132

rs6962939 ITU & JPT 0.12071 0.00101

rs6962939 ITU & CEU 0.41081 0.00147

rs6962939 ITU & FIN 0.45094 0.00109

rs6962939 ITU & GBR 1.00000 0.00000

rs6962939 ITU & IBS 0.13592 0.00134

rs6962939 ITU & TSI 0.77201 0.00075

rs6962939 ITU & BEB 0.11460 0.00126

rs6962939 ITU & GIH 1.00000 0.00000

rs6962939 PJL & ACB 0.00028 0.00005

rs6962939 PJL & ASW 0.12155 0.00124

rs6962939 PJL & ESN 0.00031 0.00006

rs6962939 PJL & GWD 0.00113 0.00014

rs6962939 PJL & LWK 0.00000 0.00000

rs6962939 PJL & MSL 0.00000 0.00000

rs6962939 PJL & YRI 0.00024 0.00006

rs6962939 PJL & PUR 0.80282 0.00074

rs6962939 PJL & CLM 1.00000 0.00000

rs6962939 PJL & PEL 0.77497 0.00078

rs6962939 PJL & MXL 0.54208 0.00133

rs6962939 PJL & CHS 0.00510 0.00020

rs6962939 PJL & CDX 0.01534 0.00038

rs6962939 PJL & KHV 0.00681 0.00021

rs6962939 PJL & CHB 0.09546 0.00093

rs6962939 PJL & JPT 0.03075 0.00057

rs6962939 PJL & CEU 1.00000 0.00000

rs6962939 PJL & FIN 0.10006 0.00115

rs6962939 PJL & GBR 0.77257 0.00084

rs6962939 PJL & IBS 0.47900 0.00165

rs6962939 PJL & TSI 1.00000 0.00000

rs6962939 PJL & BEB 0.45802 0.00174

rs6962939 PJL & GIH 0.56347 0.00132

rs6962939 PJL & ITU 0.56589 0.00145

rs6962939 STU & ACB 0.00060 0.00010

rs6962939 STU & ASW 0.21908 0.00172

rs6962939 STU & ESN 0.00092 0.00014

rs6962939 STU & GWD 0.00308 0.00022

rs6962939 STU & LWK 0.00004 0.00002

rs6962939 STU & MSL 0.00003 0.00002

rs6962939 STU & YRI 0.00106 0.00012

rs6962939 STU & PUR 1.00000 0.00000

rs6962939 STU & CLM 0.60679 0.00146

rs6962939 STU & PEL 0.58739 0.00126

rs6962939 STU & MXL 0.38315 0.00138

rs6962939 STU & CHS 0.00160 0.00011

rs6962939 STU & CDX 0.00370 0.00015

rs6962939 STU & KHV 0.00347 0.00015

rs6962939 STU & CHB 0.03511 0.00065

rs6962939 STU & JPT 0.01005 0.00031

rs6962939 STU & CEU 1.00000 0.00000

rs6962939 STU & FIN 0.06199 0.00076

rs6962939 STU & GBR 0.42404 0.00176

rs6962939 STU & IBS 0.65628 0.00148

rs6962939 STU & TSI 0.61446 0.00146

rs6962939 STU & BEB 0.64080 0.00156

rs6962939 STU & GIH 0.28891 0.00161

rs6962939 STU & ITU 0.41756 0.00157

rs6962939 STU & PJL 0.80039 0.00077

Locus: rs9792144

=================================

Pop Alleles:

-----------------------------------------------------------

2 3 Total

ACB 150 42 192

ASW 100 22 122

ESN 152 46 198

GWD 196 30 226

LWK 149 49 198

MSL 142 28 170

YRI 175 41 216

PUR 194 14 208

CLM 145 43 188

PEL 132 38 170

MXL 115 13 128

CHS 179 31 210

CDX 144 42 186

KHV 150 48 198

CHB 176 30 206

JPT 180 28 208

CEU 169 29 198

FIN 155 43 198

GBR 166 16 182

IBS 186 28 214

TSI 191 23 214

BEB 154 18 172

GIH 185 21 206

ITU 161 43 204

PJL 167 25 192

STU 175 29 204

Total: 4188820 5008

Locus Population pair P-Value S.E.

----------- --------------------- ------- -------

rs9792144 ASW & ACB 0.46910 0.00267

rs9792144 ESN & ACB 0.80915 0.00166

rs9792144 ESN & ASW 0.32720 0.00304

rs9792144 GWD & ACB 0.02739 0.00106

rs9792144 GWD & ASW 0.27238 0.00263

rs9792144 GWD & ESN 0.01142 0.00073

rs9792144 LWK & ACB 0.54856 0.00316

rs9792144 LWK & ASW 0.16701 0.00267

rs9792144 LWK & ESN 0.81346 0.00169

rs9792144 LWK & GWD 0.00261 0.00028

rs9792144 MSL & ACB 0.23153 0.00285

rs9792144 MSL & ASW 0.75282 0.00173

rs9792144 MSL & ESN 0.11624 0.00198

rs9792144 MSL & GWD 0.39234 0.00325

rs9792144 MSL & LWK 0.05510 0.00163

rs9792144 YRI & ACB 0.53751 0.00307

rs9792144 YRI & ASW 0.88543 0.00080

rs9792144 YRI & ESN 0.33029 0.00329

rs9792144 YRI & GWD 0.12038 0.00215

rs9792144 YRI & LWK 0.19166 0.00301

rs9792144 YRI & MSL 0.59550 0.00263

rs9792144 PUR & ACB 0.00000 0.00000

rs9792144 PUR & ASW 0.00273 0.00022

rs9792144 PUR & ESN 0.00000 0.00000

rs9792144 PUR & GWD 0.02702 0.00084

rs9792144 PUR & LWK 0.00000 0.00000

rs9792144 PUR & MSL 0.00440 0.00031

rs9792144 PUR & YRI 0.00010 0.00003

rs9792144 CLM & ACB 0.90300 0.00078

rs9792144 CLM & ASW 0.32497 0.00244

rs9792144 CLM & ESN 1.00000 0.00000

rs9792144 CLM & GWD 0.01417 0.00070

rs9792144 CLM & LWK 0.71964 0.00217

rs9792144 CLM & MSL 0.14515 0.00244

rs9792144 CLM & YRI 0.38829 0.00335

rs9792144 CLM & PUR 0.00001 0.00001

rs9792144 PEL & ACB 1.00000 0.00000

rs9792144 PEL & ASW 0.38241 0.00287

rs9792144 PEL & ESN 0.90219 0.00090

rs9792144 PEL & GWD 0.02071 0.00088

rs9792144 PEL & LWK 0.62648 0.00269

rs9792144 PEL & MSL 0.21538 0.00258

rs9792144 PEL & YRI 0.44414 0.00310

rs9792144 PEL & PUR 0.00003 0.00003

rs9792144 PEL & CLM 1.00000 0.00000

rs9792144 MXL & ACB 0.00687 0.00042

rs9792144 MXL & ASW 0.10178 0.00160

rs9792144 MXL & ESN 0.00307 0.00034

rs9792144 MXL & GWD 0.40415 0.00222

rs9792144 MXL & LWK 0.00083 0.00012

rs9792144 MXL & MSL 0.12862 0.00189

rs9792144 MXL & YRI 0.03389 0.00100

rs9792144 MXL & PUR 0.30230 0.00220

rs9792144 MXL & CLM 0.00383 0.00032

rs9792144 MXL & PEL 0.00527 0.00033

rs9792144 CHS & ACB 0.07248 0.00173

rs9792144 CHS & ASW 0.43914 0.00281

rs9792144 CHS & ESN 0.03312 0.00129

rs9792144 CHS & GWD 0.67824 0.00228

rs9792144 CHS & LWK 0.01175 0.00078

rs9792144 CHS & MSL 0.67722 0.00199

rs9792144 CHS & YRI 0.30333 0.00330

rs9792144 CHS & PUR 0.01219 0.00058

rs9792144 CHS & CLM 0.03980 0.00127

rs9792144 CHS & PEL 0.05949 0.00140

rs9792144 CHS & MXL 0.24531 0.00245

rs9792144 CDX & ACB 0.90075 0.00094

rs9792144 CDX & ASW 0.38768 0.00336

rs9792144 CDX & ESN 0.90556 0.00085

rs9792144 CDX & GWD 0.01813 0.00079

rs9792144 CDX & LWK 0.63138 0.00271

rs9792144 CDX & MSL 0.17941 0.00237

rs9792144 CDX & YRI 0.39026 0.00348

rs9792144 CDX & PUR 0.00001 0.00001

rs9792144 CDX & CLM 1.00000 0.00000

rs9792144 CDX & PEL 1.00000 0.00000

rs9792144 CDX & MXL 0.00439 0.00035

rs9792144 CDX & CHS 0.05145 0.00154

rs9792144 KHV & ACB 0.63097 0.00286

rs9792144 KHV & ASW 0.20903 0.00297

rs9792144 KHV & ESN 0.90611 0.00088

rs9792144 KHV & GWD 0.00340 0.00032

rs9792144 KHV & LWK 1.00000 0.00000

rs9792144 KHV & MSL 0.07252 0.00186

rs9792144 KHV & YRI 0.22557 0.00297

rs9792144 KHV & PUR 0.00000 0.00000

rs9792144 KHV & CLM 0.81445 0.00165

rs9792144 KHV & PEL 0.71213 0.00250

rs9792144 KHV & MXL 0.00105 0.00019

rs9792144 KHV & CHS 0.01682 0.00084

rs9792144 KHV & CDX 0.72214 0.00232

rs9792144 CHB & ACB 0.06675 0.00173

rs9792144 CHB & ASW 0.43263 0.00301

rs9792144 CHB & ESN 0.02951 0.00102

rs9792144 CHB & GWD 0.78363 0.00145

rs9792144 CHB & LWK 0.01142 0.00060

rs9792144 CHB & MSL 0.66748 0.00229

rs9792144 CHB & YRI 0.24090 0.00313

rs9792144 CHB & PUR 0.01034 0.00047

rs9792144 CHB & CLM 0.03808 0.00123

rs9792144 CHB & PEL 0.06084 0.00169

rs9792144 CHB & MXL 0.24692 0.00270

rs9792144 CHB & CHS 1.00000 0.00000

rs9792144 CHB & CDX 0.05034 0.00142

rs9792144 CHB & KHV 0.01629 0.00082

rs9792144 JPT & ACB 0.03585 0.00120

rs9792144 JPT & ASW 0.33989 0.00275

rs9792144 JPT & ESN 0.01341 0.00068

rs9792144 JPT & GWD 1.00000 0.00000

rs9792144 JPT & LWK 0.00531 0.00047

rs9792144 JPT & MSL 0.46751 0.00253

rs9792144 JPT & YRI 0.14799 0.00258

rs9792144 JPT & PUR 0.03254 0.00089

rs9792144 JPT & CLM 0.01804 0.00088

rs9792144 JPT & PEL 0.02779 0.00103

rs9792144 JPT & MXL 0.39740 0.00258

rs9792144 JPT & CHS 0.77977 0.00175

rs9792144 JPT & CDX 0.02562 0.00100

rs9792144 JPT & KHV 0.00690 0.00053

rs9792144 JPT & CHB 0.77874 0.00145

rs9792144 CEU & ACB 0.06692 0.00166

rs9792144 CEU & ASW 0.43166 0.00245

rs9792144 CEU & ESN 0.03947 0.00135

rs9792144 CEU & GWD 0.77676 0.00167

rs9792144 CEU & LWK 0.01597 0.00078

rs9792144 CEU & MSL 0.66691 0.00214

rs9792144 CEU & YRI 0.29450 0.00298

rs9792144 CEU & PUR 0.00987 0.00051

rs9792144 CEU & CLM 0.05046 0.00147

rs9792144 CEU & PEL 0.05861 0.00143

rs9792144 CEU & MXL 0.24324 0.00264

rs9792144 CEU & CHS 1.00000 0.00000

rs9792144 CEU & CDX 0.05096 0.00151

rs9792144 CEU & KHV 0.02377 0.00101

rs9792144 CEU & CHB 1.00000 0.00000

rs9792144 CEU & JPT 0.77534 0.00153

rs9792144 FIN & ACB 1.00000 0.00000

rs9792144 FIN & ASW 0.47727 0.00297

rs9792144 FIN & ESN 0.81010 0.00167

rs9792144 FIN & GWD 0.02898 0.00101

rs9792144 FIN & LWK 0.55626 0.00341

rs9792144 FIN & MSL 0.23308 0.00262

rs9792144 FIN & YRI 0.53798 0.00301

rs9792144 FIN & PUR 0.00001 0.00000

rs9792144 FIN & CLM 0.80639 0.00168

rs9792144 FIN & PEL 0.89905 0.00085

rs9792144 FIN & MXL 0.00660 0.00044

rs9792144 FIN & CHS 0.07358 0.00195

rs9792144 FIN & CDX 0.90182 0.00083

rs9792144 FIN & KHV 0.63499 0.00269

rs9792144 FIN & CHB 0.07069 0.00168

rs9792144 FIN & JPT 0.03586 0.00134

rs9792144 FIN & CEU 0.09117 0.00222

rs9792144 GBR & ACB 0.00046 0.00011

rs9792144 GBR & ASW 0.02149 0.00071

rs9792144 GBR & ESN 0.00017 0.00005

rs9792144 GBR & GWD 0.16245 0.00217

rs9792144 GBR & LWK 0.00003 0.00001

rs9792144 GBR & MSL 0.03690 0.00093

rs9792144 GBR & YRI 0.00382 0.00030

rs9792144 GBR & PUR 0.56911 0.00187

rs9792144 GBR & CLM 0.00020 0.00007

rs9792144 GBR & PEL 0.00052 0.00010

rs9792144 GBR & MXL 0.69729 0.00147

rs9792144 GBR & CHS 0.08441 0.00138

rs9792144 GBR & CDX 0.00021 0.00006

rs9792144 GBR & KHV 0.00004 0.00002

rs9792144 GBR & CHB 0.08612 0.00162

rs9792144 GBR & JPT 0.15376 0.00216

rs9792144 GBR & CEU 0.08217 0.00153

rs9792144 GBR & FIN 0.00038 0.00008

rs9792144 IBS & ACB 0.02616 0.00087

rs9792144 IBS & ASW 0.26409 0.00265

rs9792144 IBS & ESN 0.01070 0.00060

rs9792144 IBS & GWD 1.00000 0.00000

rs9792144 IBS & LWK 0.00388 0.00041

rs9792144 IBS & MSL 0.38354 0.00288

rs9792144 IBS & YRI 0.10988 0.00193

rs9792144 IBS & PUR 0.03497 0.00110

rs9792144 IBS & CLM 0.01153 0.00058

rs9792144 IBS & PEL 0.02042 0.00108

rs9792144 IBS & MXL 0.49628 0.00252

rs9792144 IBS & CHS 0.67550 0.00202

rs9792144 IBS & CDX 0.01608 0.00082

rs9792144 IBS & KHV 0.00550 0.00045

rs9792144 IBS & CHB 0.67027 0.00203

rs9792144 IBS & JPT 1.00000 0.00000

rs9792144 IBS & CEU 0.67116 0.00190

rs9792144 IBS & FIN 0.02660 0.00119

rs9792144 IBS & GBR 0.20042 0.00278

rs9792144 TSI & ACB 0.00288 0.00026

rs9792144 TSI & ASW 0.06862 0.00132

rs9792144 TSI & ESN 0.00090 0.00015

rs9792144 TSI & GWD 0.46297 0.00302

rs9792144 TSI & LWK 0.00027 0.00007

rs9792144 TSI & MSL 0.12901 0.00203

rs9792144 TSI & YRI 0.02184 0.00094

rs9792144 TSI & PUR 0.17058 0.00210

rs9792144 TSI & CLM 0.00106 0.00020

rs9792144 TSI & PEL 0.00281 0.00032

rs9792144 TSI & MXL 1.00000 0.00000

rs9792144 TSI & CHS 0.24445 0.00316

rs9792144 TSI & CDX 0.00192 0.00023

rs9792144 TSI & KHV 0.00042 0.00010

rs9792144 TSI & CHB 0.24395 0.00280

rs9792144 TSI & JPT 0.45397 0.00248

rs9792144 TSI & CEU 0.23750 0.00282

rs9792144 TSI & FIN 0.00288 0.00028

rs9792144 TSI & GBR 0.61332 0.00182

rs9792144 TSI & IBS 0.54977 0.00227

rs9792144 BEB & ACB 0.00411 0.00034

rs9792144 BEB & ASW 0.08239 0.00135

rs9792144 BEB & ESN 0.00146 0.00019

rs9792144 BEB & GWD 0.44271 0.00298

rs9792144 BEB & LWK 0.00028 0.00012

rs9792144 BEB & MSL 0.11210 0.00199

rs9792144 BEB & YRI 0.02334 0.00104

rs9792144 BEB & PUR 0.26784 0.00205

rs9792144 BEB & CLM 0.00179 0.00016

rs9792144 BEB & PEL 0.00292 0.00024

rs9792144 BEB & MXL 1.00000 0.00000

rs9792144 BEB & CHS 0.22281 0.00271

rs9792144 BEB & CDX 0.00245 0.00024

rs9792144 BEB & KHV 0.00072 0.00013

rs9792144 BEB & CHB 0.28263 0.00282

rs9792144 BEB & JPT 0.42707 0.00255

rs9792144 BEB & CEU 0.27445 0.00267

rs9792144 BEB & FIN 0.00454 0.00037

rs9792144 BEB & GBR 0.71832 0.00148

rs9792144 BEB & IBS 0.43700 0.00318

rs9792144 BEB & TSI 1.00000 0.00000

rs9792144 GIH & ACB 0.00165 0.00024

rs9792144 GIH & ASW 0.05972 0.00147

rs9792144 GIH & ESN 0.00048 0.00009

rs9792144 GIH & GWD 0.37009 0.00274

rs9792144 GIH & LWK 0.00015 0.00006

rs9792144 GIH & MSL 0.08988 0.00154

rs9792144 GIH & YRI 0.01241 0.00064

rs9792144 GIH & PUR 0.22449 0.00201

rs9792144 GIH & CLM 0.00092 0.00018

rs9792144 GIH & PEL 0.00173 0.00022

rs9792144 GIH & MXL 1.00000 0.00000

rs9792144 GIH & CHS 0.18008 0.00263

rs9792144 GIH & CDX 0.00063 0.00010

rs9792144 GIH & KHV 0.00017 0.00006

rs9792144 GIH & CHB 0.23107 0.00259

rs9792144 GIH & JPT 0.36390 0.00260

rs9792144 GIH & CEU 0.22789 0.00266

rs9792144 GIH & FIN 0.00199 0.00026

rs9792144 GIH & GBR 0.72818 0.00160

rs9792144 GIH & IBS 0.36653 0.00261

rs9792144 GIH & TSI 0.87419 0.00087

rs9792144 GIH & BEB 1.00000 0.00000

rs9792144 ITU & ACB 0.90332 0.00079

rs9792144 ITU & ASW 0.56801 0.00261

rs9792144 ITU & ESN 0.63098 0.00264

rs9792144 ITU & GWD 0.03874 0.00109

rs9792144 ITU & LWK 0.41361 0.00319

rs9792144 ITU & MSL 0.29008 0.00268

rs9792144 ITU & YRI 0.62660 0.00274

rs9792144 ITU & PUR 0.00001 0.00001

rs9792144 ITU & CLM 0.71423 0.00267

rs9792144 ITU & PEL 0.80435 0.00176

rs9792144 ITU & MXL 0.01024 0.00046

rs9792144 ITU & CHS 0.10235 0.00214

rs9792144 ITU & CDX 0.80724 0.00152

rs9792144 ITU & KHV 0.47620 0.00326

rs9792144 ITU & CHB 0.09596 0.00183

rs9792144 ITU & JPT 0.04919 0.00139

rs9792144 ITU & CEU 0.11683 0.00232

rs9792144 ITU & FIN 0.90449 0.00089

rs9792144 ITU & GBR 0.00061 0.00010

rs9792144 ITU & IBS 0.03658 0.00117

rs9792144 ITU & TSI 0.00458 0.00044

rs9792144 ITU & BEB 0.00766 0.00053

rs9792144 ITU & GIH 0.00217 0.00022

rs9792144 PJL & ACB 0.02990 0.00113

rs9792144 PJL & ASW 0.25784 0.00250

rs9792144 PJL & ESN 0.01240 0.00064

rs9792144 PJL & GWD 1.00000 0.00000

rs9792144 PJL & LWK 0.00409 0.00035

rs9792144 PJL & MSL 0.37332 0.00304

rs9792144 PJL & YRI 0.10971 0.00228

rs9792144 PJL & PUR 0.04193 0.00096

rs9792144 PJL & CLM 0.01618 0.00082

rs9792144 PJL & PEL 0.02402 0.00090

rs9792144 PJL & MXL 0.47938 0.00233

rs9792144 PJL & CHS 0.66471 0.00214

rs9792144 PJL & CDX 0.01617 0.00073

rs9792144 PJL & KHV 0.00634 0.00049

rs9792144 PJL & CHB 0.66758 0.00210

rs9792144 PJL & JPT 1.00000 0.00000

rs9792144 PJL & CEU 0.66297 0.00215

rs9792144 PJL & FIN 0.03198 0.00123

rs9792144 PJL & GBR 0.24637 0.00249

rs9792144 PJL & IBS 1.00000 0.00000

rs9792144 PJL & TSI 0.54031 0.00272

rs9792144 PJL & BEB 0.51580 0.00263

rs9792144 PJL & GIH 0.43432 0.00237

rs9792144 PJL & ITU 0.04382 0.00136

rs9792144 STU & ACB 0.04804 0.00149

rs9792144 STU & ASW 0.43100 0.00229

rs9792144 STU & ESN 0.02029 0.00095

rs9792144 STU & GWD 0.78042 0.00159

rs9792144 STU & LWK 0.00811 0.00051

rs9792144 STU & MSL 0.56751 0.00245

rs9792144 STU & YRI 0.19296 0.00264

rs9792144 STU & PUR 0.01556 0.00068

rs9792144 STU & CLM 0.03852 0.00139

rs9792144 STU & PEL 0.04429 0.00134

rs9792144 STU & MXL 0.31074 0.00203

rs9792144 STU & CHS 0.88929 0.00095

rs9792144 STU & CDX 0.03742 0.00112

rs9792144 STU & KHV 0.01065 0.00056

rs9792144 STU & CHB 1.00000 0.00000

rs9792144 STU & JPT 0.88709 0.00085

rs9792144 STU & CEU 1.00000 0.00000

rs9792144 STU & FIN 0.05100 0.00133

rs9792144 STU & GBR 0.11063 0.00186

rs9792144 STU & IBS 0.77650 0.00157

rs9792144 STU & TSI 0.30439 0.00290

rs9792144 STU & BEB 0.27849 0.00285

rs9792144 STU & GIH 0.22941 0.00271

rs9792144 STU & ITU 0.09191 0.00194

rs9792144 STU & PJL 0.76858 0.00154

Locus: rs6108746

=================================

Pop Alleles:

-----------------------------------------------------------

2 4 Total

ACB 22 170 192

ASW 11 111 122

ESN 25 173 198

GWD 23 203 226

LWK 27 171 198

MSL 27 143 170

YRI 31 185 216

PUR 29 179 208

CLM 22 166 188

PEL 25 145 170

MXL 22 106 128

CHS 8 202 210

CDX 8 178 186

KHV 4 194 198

CHB 9 197 206

JPT 9 199 208

CEU 40 158 198

FIN 33 165 198

GBR 37 145 182

IBS 36 178 214

TSI 51 163 214

BEB 21 151 172

GIH 31 175 206

ITU 29 175 204

PJL 41 151 192

STU 35 169 204

Total: 656 4352 5008

Locus Population pair P-Value S.E.

----------- --------------------- ------- -------

rs6108746 ASW & ACB 0.57420 0.00189

rs6108746 ESN & ACB 0.75723 0.00159

rs6108746 ESN & ASW 0.36952 0.00226

rs6108746 GWD & ACB 0.75132 0.00152

rs6108746 GWD & ASW 0.84962 0.00092

rs6108746 GWD & ESN 0.44425 0.00267

rs6108746 LWK & ACB 0.53659 0.00252

rs6108746 LWK & ASW 0.22244 0.00228

rs6108746 LWK & ESN 0.88133 0.00094

rs6108746 LWK & GWD 0.28838 0.00258

rs6108746 MSL & ACB 0.28210 0.00254

rs6108746 MSL & ASW 0.11215 0.00175

rs6108746 MSL & ESN 0.45815 0.00263

rs6108746 MSL & GWD 0.12755 0.00202

rs6108746 MSL & LWK 0.55920 0.00256

rs6108746 YRI & ACB 0.46154 0.00299

rs6108746 YRI & ASW 0.17363 0.00189

rs6108746 YRI & ESN 0.66644 0.00205

rs6108746 YRI & GWD 0.19293 0.00246

rs6108746 YRI & LWK 0.88814 0.00072

rs6108746 YRI & MSL 0.77309 0.00181

rs6108746 PUR & ACB 0.55188 0.00280

rs6108746 PUR & ASW 0.22438 0.00235

rs6108746 PUR & ESN 0.76951 0.00147

rs6108746 PUR & GWD 0.23415 0.00246

rs6108746 PUR & LWK 1.00000 0.00000

rs6108746 PUR & MSL 0.66360 0.00196

rs6108746 PUR & YRI 1.00000 0.00000

rs6108746 CLM & ACB 1.00000 0.00000

rs6108746 CLM & ASW 0.46696 0.00211

rs6108746 CLM & ESN 0.87806 0.00083

rs6108746 CLM & GWD 0.63740 0.00218

rs6108746 CLM & LWK 0.64985 0.00236

rs6108746 CLM & MSL 0.28644 0.00244

rs6108746 CLM & YRI 0.46184 0.00275

rs6108746 CLM & PUR 0.55055 0.00255

rs6108746 PEL & ACB 0.43760 0.00263

rs6108746 PEL & ASW 0.15482 0.00175

rs6108746 PEL & ESN 0.65071 0.00225

rs6108746 PEL & GWD 0.21553 0.00246

rs6108746 PEL & LWK 0.87938 0.00083

rs6108746 PEL & MSL 0.88009 0.00088

rs6108746 PEL & YRI 1.00000 0.00000

rs6108746 PEL & PUR 0.88340 0.00092

rs6108746 PEL & CLM 0.43655 0.00256

rs6108746 MXL & ACB 0.18625 0.00248

rs6108746 MXL & ASW 0.06333 0.00114

rs6108746 MXL & ESN 0.26341 0.00258

rs6108746 MXL & GWD 0.06720 0.00143

rs6108746 MXL & LWK 0.42760 0.00270

rs6108746 MXL & MSL 0.87419 0.00093

rs6108746 MXL & YRI 0.53735 0.00255

rs6108746 MXL & PUR 0.43915 0.00249

rs6108746 MXL & CLM 0.18647 0.00210

rs6108746 MXL & PEL 0.62846 0.00221

rs6108746 CHS & ACB 0.00414 0.00028

rs6108746 CHS & ASW 0.08218 0.00100

rs6108746 CHS & ESN 0.00166 0.00015

rs6108746 CHS & GWD 0.01438 0.00054

rs6108746 CHS & LWK 0.00058 0.00008

rs6108746 CHS & MSL 0.00006 0.00004

rs6108746 CHS & YRI 0.00012 0.00004

rs6108746 CHS & PUR 0.00022 0.00005

rs6108746 CHS & CLM 0.00315 0.00023

rs6108746 CHS & PEL 0.00019 0.00004

rs6108746 CHS & MXL 0.00004 0.00002

rs6108746 CDX & ACB 0.01202 0.00051

rs6108746 CDX & ASW 0.14489 0.00143

rs6108746 CDX & ESN 0.00333 0.00026

rs6108746 CDX & GWD 0.02439 0.00065

rs6108746 CDX & LWK 0.00131 0.00014

rs6108746 CDX & MSL 0.00024 0.00004

rs6108746 CDX & YRI 0.00067 0.00011

rs6108746 CDX & PUR 0.00104 0.00016

rs6108746 CDX & CLM 0.01266 0.00057

rs6108746 CDX & PEL 0.00087 0.00012

rs6108746 CDX & MXL 0.00025 0.00006

rs6108746 CDX & CHS 1.00000 0.00000

rs6108746 KHV & ACB 0.00015 0.00004

rs6108746 KHV & ASW 0.01144 0.00035

rs6108746 KHV & ESN 0.00013 0.00004

rs6108746 KHV & GWD 0.00048 0.00008

rs6108746 KHV & LWK 0.00000 0.00000

rs6108746 KHV & MSL 0.00000 0.00000

rs6108746 KHV & YRI 0.00000 0.00000

rs6108746 KHV & PUR 0.00000 0.00000

rs6108746 KHV & CLM 0.00020 0.00006

rs6108746 KHV & PEL 0.00000 0.00000

rs6108746 KHV & MXL 0.00000 0.00000

rs6108746 KHV & CHS 0.38351 0.00155

rs6108746 KHV & CDX 0.24639 0.00155

rs6108746 CHB & ACB 0.00934 0.00043

rs6108746 CHB & ASW 0.15046 0.00135

rs6108746 CHB & ESN 0.00378 0.00025

rs6108746 CHB & GWD 0.02534 0.00073

rs6108746 CHB & LWK 0.00119 0.00013

rs6108746 CHB & MSL 0.00013 0.00004

rs6108746 CHB & YRI 0.00058 0.00010

rs6108746 CHB & PUR 0.00117 0.00016

rs6108746 CHB & CLM 0.00791 0.00036

rs6108746 CHB & PEL 0.00072 0.00010

rs6108746 CHB & MXL 0.00011 0.00003

rs6108746 CHB & CHS 0.80714 0.00088

rs6108746 CHB & CDX 1.00000 0.00000

rs6108746 CHB & KHV 0.25925 0.00161

rs6108746 JPT & ACB 0.00929 0.00045

rs6108746 JPT & ASW 0.15223 0.00144

rs6108746 JPT & ESN 0.00356 0.00026

rs6108746 JPT & GWD 0.02586 0.00073

rs6108746 JPT & LWK 0.00122 0.00014

rs6108746 JPT & MSL 0.00021 0.00006

rs6108746 JPT & YRI 0.00048 0.00010

rs6108746 JPT & PUR 0.00098 0.00014

rs6108746 JPT & CLM 0.00800 0.00036

rs6108746 JPT & PEL 0.00071 0.00012

rs6108746 JPT & MXL 0.00009 0.00002

rs6108746 JPT & CHS 0.80943 0.00084

rs6108746 JPT & CDX 1.00000 0.00000

rs6108746 JPT & KHV 0.26079 0.00135

rs6108746 JPT & CHB 1.00000 0.00000

rs6108746 CEU & ACB 0.01843 0.00082

rs6108746 CEU & ASW 0.00738 0.00036

rs6108746 CEU & ESN 0.05809 0.00168

rs6108746 CEU & GWD 0.00399 0.00039

rs6108746 CEU & LWK 0.10866 0.00188

rs6108746 CEU & MSL 0.33876 0.00288

rs6108746 CEU & YRI 0.11852 0.00226

rs6108746 CEU & PUR 0.11355 0.00238

rs6108746 CEU & CLM 0.02691 0.00112

rs6108746 CEU & PEL 0.17364 0.00265

rs6108746 CEU & MXL 0.56466 0.00264

rs6108746 CEU & CHS 0.00000 0.00000

rs6108746 CEU & CDX 0.00000 0.00000

rs6108746 CEU & KHV 0.00000 0.00000

rs6108746 CEU & CHB 0.00000 0.00000

rs6108746 CEU & JPT 0.00000 0.00000

rs6108746 FIN & ACB 0.15126 0.00225

rs6108746 FIN & ASW 0.06577 0.00140

rs6108746 FIN & ESN 0.31772 0.00320

rs6108746 FIN & GWD 0.06233 0.00156

rs6108746 FIN & LWK 0.48406 0.00317

rs6108746 FIN & MSL 0.88647 0.00079

rs6108746 FIN & YRI 0.58407 0.00255

rs6108746 FIN & PUR 0.49995 0.00296

rs6108746 FIN & CLM 0.18756 0.00262

rs6108746 FIN & PEL 0.66564 0.00219

rs6108746 FIN & MXL 1.00000 0.00000

rs6108746 FIN & CHS 0.00002 0.00001

rs6108746 FIN & CDX 0.00013 0.00004

rs6108746 FIN & KHV 0.00000 0.00000

rs6108746 FIN & CHB 0.00011 0.00004

rs6108746 FIN & JPT 0.00001 0.00001

rs6108746 FIN & CEU 0.43730 0.00291

rs6108746 GBR & ACB 0.02499 0.00098

rs6108746 GBR & ASW 0.00948 0.00047

rs6108746 GBR & ESN 0.05345 0.00130

rs6108746 GBR & GWD 0.00411 0.00030

rs6108746 GBR & LWK 0.10091 0.00186

rs6108746 GBR & MSL 0.32777 0.00319

rs6108746 GBR & YRI 0.14114 0.00237

rs6108746 GBR & PUR 0.11067 0.00234

rs6108746 GBR & CLM 0.03283 0.00116

rs6108746 GBR & PEL 0.20982 0.00259

rs6108746 GBR & MXL 0.55403 0.00295

rs6108746 GBR & CHS 0.00000 0.00000

rs6108746 GBR & CDX 0.00000 0.00000

rs6108746 GBR & KHV 0.00000 0.00000

rs6108746 GBR & CHB 0.00001 0.00001

rs6108746 GBR & JPT 0.00000 0.00000

rs6108746 GBR & CEU 1.00000 0.00000

rs6108746 GBR & FIN 0.42054 0.00283

rs6108746 IBS & ACB 0.15434 0.00248

rs6108746 IBS & ASW 0.05041 0.00108

rs6108746 IBS & ESN 0.26562 0.00277

rs6108746 IBS & GWD 0.04778 0.00152

rs6108746 IBS & LWK 0.41440 0.00322

rs6108746 IBS & MSL 0.89063 0.00100

rs6108746 IBS & YRI 0.50708 0.00298

rs6108746 IBS & PUR 0.41888 0.00319

rs6108746 IBS & CLM 0.15983 0.00240

rs6108746 IBS & PEL 0.57762 0.00254

rs6108746 IBS & MXL 1.00000 0.00000

rs6108746 IBS & CHS 0.00000 0.00000

rs6108746 IBS & CDX 0.00005 0.00003

rs6108746 IBS & KHV 0.00000 0.00000

rs6108746 IBS & CHB 0.00002 0.00001

rs6108746 IBS & JPT 0.00001 0.00001

rs6108746 IBS & CEU 0.44826 0.00308

rs6108746 IBS & FIN 1.00000 0.00000

rs6108746 IBS & GBR 0.43390 0.00291

rs6108746 TSI & ACB 0.00131 0.00022

rs6108746 TSI & ASW 0.00074 0.00012

rs6108746 TSI & ESN 0.00340 0.00032

rs6108746 TSI & GWD 0.00015 0.00006

rs6108746 TSI & LWK 0.00810 0.00059

rs6108746 TSI & MSL 0.05400 0.00161

rs6108746 TSI & YRI 0.01423 0.00079

rs6108746 TSI & PUR 0.01353 0.00074

rs6108746 TSI & CLM 0.00160 0.00023

rs6108746 TSI & PEL 0.02814 0.00106

rs6108746 TSI & MXL 0.16807 0.00264

rs6108746 TSI & CHS 0.00000 0.00000

rs6108746 TSI & CDX 0.00000 0.00000

rs6108746 TSI & KHV 0.00000 0.00000

rs6108746 TSI & CHB 0.00000 0.00000

rs6108746 TSI & JPT 0.00000 0.00000

rs6108746 TSI & CEU 0.40642 0.00345

rs6108746 TSI & FIN 0.08582 0.00228

rs6108746 TSI & GBR 0.46378 0.00368

rs6108746 TSI & IBS 0.09290 0.00228

rs6108746 BEB & ACB 0.87149 0.00079

rs6108746 BEB & ASW 0.45293 0.00188

rs6108746 BEB & ESN 1.00000 0.00000

rs6108746 BEB & GWD 0.62654 0.00199

rs6108746 BEB & LWK 0.75758 0.00153

rs6108746 BEB & MSL 0.35125 0.00276

rs6108746 BEB & YRI 0.55484 0.00246

rs6108746 BEB & PUR 0.65021 0.00201

rs6108746 BEB & CLM 1.00000 0.00000

rs6108746 BEB & PEL 0.52818 0.00235

rs6108746 BEB & MXL 0.24655 0.00227

rs6108746 BEB & CHS 0.00274 0.00024

rs6108746 BEB & CDX 0.00570 0.00028

rs6108746 BEB & KHV 0.00015 0.00004

rs6108746 BEB & CHB 0.00674 0.00038

rs6108746 BEB & JPT 0.00657 0.00033

rs6108746 BEB & CEU 0.04865 0.00117

rs6108746 BEB & FIN 0.24052 0.00259

rs6108746 BEB & GBR 0.04286 0.00127

rs6108746 BEB & IBS 0.24766 0.00253

rs6108746 BEB & TSI 0.00428 0.00037

rs6108746 GIH & ACB 0.30754 0.00298

rs6108746 GIH & ASW 0.12493 0.00173

rs6108746 GIH & ESN 0.56177 0.00231

rs6108746 GIH & GWD 0.14443 0.00226

rs6108746 GIH & LWK 0.77762 0.00170

rs6108746 GIH & MSL 0.88603 0.00092

rs6108746 GIH & YRI 0.89142 0.00091

rs6108746 GIH & PUR 0.78160 0.00158

rs6108746 GIH & CLM 0.37452 0.00252

rs6108746 GIH & PEL 1.00000 0.00000

rs6108746 GIH & MXL 0.64441 0.00198

rs6108746 GIH & CHS 0.00013 0.00004

rs6108746 GIH & CDX 0.00035 0.00008

rs6108746 GIH & KHV 0.00000 0.00000

rs6108746 GIH & CHB 0.00030 0.00007

rs6108746 GIH & JPT 0.00016 0.00004

rs6108746 GIH & CEU 0.18986 0.00264

rs6108746 GIH & FIN 0.68354 0.00241

rs6108746 GIH & GBR 0.18453 0.00277

rs6108746 GIH & IBS 0.68831 0.00250

rs6108746 GIH & TSI 0.02706 0.00118

rs6108746 GIH & BEB 0.46048 0.00290

rs6108746 ITU & ACB 0.45677 0.00267

rs6108746 ITU & ASW 0.16730 0.00200

rs6108746 ITU & ESN 0.66570 0.00222

rs6108746 ITU & GWD 0.23492 0.00246

rs6108746 ITU & LWK 0.88582 0.00090

rs6108746 ITU & MSL 0.66431 0.00214

rs6108746 ITU & YRI 1.00000 0.00000

rs6108746 ITU & PUR 1.00000 0.00000

rs6108746 ITU & CLM 0.55214 0.00267

rs6108746 ITU & PEL 1.00000 0.00000

rs6108746 ITU & MXL 0.53141 0.00244

rs6108746 ITU & CHS 0.00028 0.00007

rs6108746 ITU & CDX 0.00085 0.00011

rs6108746 ITU & KHV 0.00001 0.00001

rs6108746 ITU & CHB 0.00043 0.00007

rs6108746 ITU & JPT 0.00050 0.00009

rs6108746 ITU & CEU 0.11156 0.00216

rs6108746 ITU & FIN 0.58506 0.00254

rs6108746 ITU & GBR 0.13529 0.00214

rs6108746 ITU & IBS 0.49668 0.00305

rs6108746 ITU & TSI 0.01362 0.00072

rs6108746 ITU & BEB 0.64800 0.00229

rs6108746 ITU & GIH 0.88802 0.00097

rs6108746 PJL & ACB 0.01293 0.00062

rs6108746 PJL & ASW 0.00498 0.00031

rs6108746 PJL & ESN 0.03019 0.00111

rs6108746 PJL & GWD 0.00152 0.00021

rs6108746 PJL & LWK 0.04752 0.00146

rs6108746 PJL & MSL 0.22327 0.00262

rs6108746 PJL & YRI 0.06732 0.00163

rs6108746 PJL & PUR 0.06522 0.00165

rs6108746 PJL & CLM 0.01364 0.00062

rs6108746 PJL & PEL 0.10400 0.00193

rs6108746 PJL & MXL 0.39607 0.00307

rs6108746 PJL & CHS 0.00000 0.00000

rs6108746 PJL & CDX 0.00000 0.00000

rs6108746 PJL & KHV 0.00000 0.00000

rs6108746 PJL & CHB 0.00000 0.00000

rs6108746 PJL & JPT 0.00000 0.00000

rs6108746 PJL & CEU 0.80175 0.00159

rs6108746 PJL & FIN 0.24999 0.00289

rs6108746 PJL & GBR 0.89872 0.00087

rs6108746 PJL & IBS 0.25149 0.00299

rs6108746 PJL & TSI 0.56225 0.00308

rs6108746 PJL & BEB 0.02583 0.00112

rs6108746 PJL & GIH 0.11765 0.00244

rs6108746 PJL & ITU 0.06446 0.00181

rs6108746 STU & ACB 0.11896 0.00186

rs6108746 STU & ASW 0.04760 0.00108

rs6108746 STU & ESN 0.21431 0.00304

rs6108746 STU & GWD 0.04751 0.00131

rs6108746 STU & LWK 0.33904 0.00254

rs6108746 STU & MSL 0.78066 0.00144

rs6108746 STU & YRI 0.50250 0.00285

rs6108746 STU & PUR 0.41419 0.00298

rs6108746 STU & CLM 0.14725 0.00213

rs6108746 STU & PEL 0.56834 0.00261

rs6108746 STU & MXL 1.00000 0.00000

rs6108746 STU & CHS 0.00001 0.00001

rs6108746 STU & CDX 0.00008 0.00004

rs6108746 STU & KHV 0.00000 0.00000

rs6108746 STU & CHB 0.00001 0.00001

rs6108746 STU & JPT 0.00000 0.00000

rs6108746 STU & CEU 0.44623 0.00301

rs6108746 STU & FIN 1.00000 0.00000

rs6108746 STU & GBR 0.43943 0.00267

rs6108746 STU & IBS 1.00000 0.00000

rs6108746 STU & TSI 0.11757 0.00259

rs6108746 STU & BEB 0.19182 0.00252

rs6108746 STU & GIH 0.59361 0.00234

rs6108746 STU & ITU 0.49814 0.00283

rs6108746 STU & PJL 0.30499 0.00302

Locus: rs6111609

=================================

Pop Alleles:

-----------------------------------------------------------

1 2 Total

ACB 4 188 192

ASW 2 120 122

ESN 0 198 198

GWD 1 225 226

LWK 4 194 198

MSL 0 170 170

YRI 0 216 216

PUR 27 181 208

CLM 20 168 188

PEL 10 160 170

MXL 10 118 128

CHS 6 204 210

CDX 4 182 186

KHV 4 194 198

CHB 4 202 206

JPT 7 201 208

CEU 37 161 198

FIN 32 166 198

GBR 42 140 182

IBS 52 162 214

TSI 45 169 214

BEB 12 160 172

GIH 20 186 206

ITU 5 199 204

PJL 12 180 192

STU 6 198 204

Total: 366 4642 5008

Locus Population pair P-Value S.E.

----------- --------------------- ------- -------

rs6111609 ASW & ACB 1.00000 0.00000

rs6111609 ESN & ACB 0.05782 0.00057

rs6111609 ESN & ASW 0.14534 0.00083

rs6111609 GWD & ACB 0.18381 0.00103

rs6111609 GWD & ASW 0.55391 0.00072

rs6111609 GWD & ESN 1.00000 0.00000

rs6111609 LWK & ACB 1.00000 0.00000

rs6111609 LWK & ASW 1.00000 0.00000

rs6111609 LWK & ESN 0.12407 0.00089

rs6111609 LWK & GWD 0.18996 0.00107

rs6111609 MSL & ACB 0.12486 0.00083

rs6111609 MSL & ASW 0.17390 0.00084

rs6111609 MSL & ESN No table

rs6111609 MSL & GWD 1.00000 0.00000

rs6111609 MSL & LWK 0.12766 0.00076

rs6111609 YRI & ACB 0.04851 0.00059

rs6111609 YRI & ASW 0.12981 0.00079

rs6111609 YRI & ESN No table

rs6111609 YRI & GWD 1.00000 0.00000

rs6111609 YRI & LWK 0.05121 0.00053

rs6111609 YRI & MSL No table

rs6111609 PUR & ACB 0.00004 0.00002

rs6111609 PUR & ASW 0.00027 0.00006

rs6111609 PUR & ESN 0.00000 0.00000

rs6111609 PUR & GWD 0.00000 0.00000

rs6111609 PUR & LWK 0.00000 0.00000

rs6111609 PUR & MSL 0.00000 0.00000

rs6111609 PUR & YRI 0.00000 0.00000

rs6111609 CLM & ACB 0.00056 0.00010

rs6111609 CLM & ASW 0.00167 0.00013

rs6111609 CLM & ESN 0.00000 0.00000

rs6111609 CLM & GWD 0.00001 0.00001

rs6111609 CLM & LWK 0.00043 0.00007

rs6111609 CLM & MSL 0.00000 0.00000

rs6111609 CLM & YRI 0.00000 0.00000

rs6111609 CLM & PUR 0.53359 0.00239

rs6111609 PEL & ACB 0.09887 0.00104

rs6111609 PEL & ASW 0.08259 0.00099

rs6111609 PEL & ESN 0.00037 0.00006

rs6111609 PEL & GWD 0.00282 0.00015

rs6111609 PEL & LWK 0.09827 0.00093

rs6111609 PEL & MSL 0.00171 0.00011

rs6111609 PEL & YRI 0.00021 0.00004

rs6111609 PEL & PUR 0.02432 0.00078

rs6111609 PEL & CLM 0.12818 0.00166

rs6111609 MXL & ACB 0.02377 0.00045

rs6111609 MXL & ASW 0.03422 0.00056

rs6111609 MXL & ESN 0.00009 0.00002

rs6111609 MXL & GWD 0.00017 0.00003

rs6111609 MXL & LWK 0.02172 0.00054

rs6111609 MXL & MSL 0.00019 0.00003

rs6111609 MXL & YRI 0.00005 0.00002

rs6111609 MXL & PUR 0.15370 0.00225

rs6111609 MXL & CLM 0.43823 0.00210

rs6111609 MXL & PEL 0.63936 0.00152

rs6111609 CHS & ACB 0.75269 0.00075

rs6111609 CHS & ASW 0.71559 0.00080

rs6111609 CHS & ESN 0.03042 0.00044

rs6111609 CHS & GWD 0.05897 0.00068

rs6111609 CHS & LWK 0.75115 0.00076

rs6111609 CHS & MSL 0.03508 0.00045

rs6111609 CHS & YRI 0.01382 0.00029

rs6111609 CHS & PUR 0.00006 0.00002

rs6111609 CHS & CLM 0.00214 0.00018

rs6111609 CHS & PEL 0.19749 0.00155

rs6111609 CHS & MXL 0.06194 0.00094

rs6111609 CDX & ACB 1.00000 0.00000

rs6111609 CDX & ASW 1.00000 0.00000

rs6111609 CDX & ESN 0.05336 0.00054

rs6111609 CDX & GWD 0.17971 0.00102

rs6111609 CDX & LWK 1.00000 0.00000

rs6111609 CDX & MSL 0.12451 0.00083

rs6111609 CDX & YRI 0.04394 0.00057

rs6111609 CDX & PUR 0.00003 0.00002

rs6111609 CDX & CLM 0.00105 0.00013

rs6111609 CDX & PEL 0.10144 0.00109

rs6111609 CDX & MXL 0.02380 0.00051

rs6111609 CDX & CHS 0.75547 0.00081

rs6111609 KHV & ACB 1.00000 0.00000

rs6111609 KHV & ASW 1.00000 0.00000

rs6111609 KHV & ESN 0.12205 0.00066

rs6111609 KHV & GWD 0.18947 0.00106

rs6111609 KHV & LWK 1.00000 0.00000

rs6111609 KHV & MSL 0.12750 0.00069

rs6111609 KHV & YRI 0.05190 0.00052

rs6111609 KHV & PUR 0.00003 0.00002

rs6111609 KHV & CLM 0.00047 0.00007

rs6111609 KHV & PEL 0.09839 0.00098

rs6111609 KHV & MXL 0.02246 0.00050

rs6111609 KHV & CHS 0.75223 0.00082

rs6111609 KHV & CDX 1.00000 0.00000

rs6111609 CHB & ACB 1.00000 0.00000

rs6111609 CHB & ASW 1.00000 0.00000

rs6111609 CHB & ESN 0.12412 0.00080

rs6111609 CHB & GWD 0.19607 0.00114

rs6111609 CHB & LWK 1.00000 0.00000

rs6111609 CHB & MSL 0.13099 0.00077

rs6111609 CHB & YRI 0.05645 0.00055

rs6111609 CHB & PUR 0.00000 0.00000

rs6111609 CHB & CLM 0.00033 0.00005

rs6111609 CHB & PEL 0.05686 0.00088

rs6111609 CHB & MXL 0.02155 0.00051

rs6111609 CHB & CHS 0.75139 0.00072

rs6111609 CHB & CDX 1.00000 0.00000

rs6111609 CHB & KHV 1.00000 0.00000

rs6111609 JPT & ACB 0.54456 0.00108

rs6111609 JPT & ASW 0.49499 0.00115

rs6111609 JPT & ESN 0.01513 0.00032

rs6111609 JPT & GWD 0.03089 0.00054

rs6111609 JPT & LWK 0.54351 0.00131

rs6111609 JPT & MSL 0.01828 0.00033

rs6111609 JPT & YRI 0.00636 0.00020

rs6111609 JPT & PUR 0.00041 0.00007

rs6111609 JPT & CLM 0.00530 0.00032

rs6111609 JPT & PEL 0.32081 0.00163

rs6111609 JPT & MXL 0.11984 0.00120

rs6111609 JPT & CHS 0.78591 0.00080

rs6111609 JPT & CDX 0.54934 0.00133

rs6111609 JPT & KHV 0.54524 0.00129

rs6111609 JPT & CHB 0.54337 0.00125

rs6111609 CEU & ACB 0.00000 0.00000

rs6111609 CEU & ASW 0.00000 0.00000

rs6111609 CEU & ESN 0.00000 0.00000

rs6111609 CEU & GWD 0.00000 0.00000

rs6111609 CEU & LWK 0.00000 0.00000

rs6111609 CEU & MSL 0.00000 0.00000

rs6111609 CEU & YRI 0.00000 0.00000

rs6111609 CEU & PUR 0.13143 0.00226

rs6111609 CEU & CLM 0.02932 0.00097

rs6111609 CEU & PEL 0.00026 0.00007

rs6111609 CEU & MXL 0.00610 0.00047

rs6111609 CEU & CHS 0.00000 0.00000

rs6111609 CEU & CDX 0.00000 0.00000

rs6111609 CEU & KHV 0.00000 0.00000

rs6111609 CEU & CHB 0.00000 0.00000

rs6111609 CEU & JPT 0.00000 0.00000

rs6111609 FIN & ACB 0.00000 0.00000

rs6111609 FIN & ASW 0.00005 0.00004

rs6111609 FIN & ESN 0.00000 0.00000

rs6111609 FIN & GWD 0.00000 0.00000

rs6111609 FIN & LWK 0.00000 0.00000

rs6111609 FIN & MSL 0.00000 0.00000

rs6111609 FIN & YRI 0.00000 0.00000

rs6111609 FIN & PUR 0.39899 0.00280

rs6111609 FIN & CLM 0.13640 0.00209

rs6111609 FIN & PEL 0.00190 0.00021

rs6111609 FIN & MXL 0.02833 0.00089

rs6111609 FIN & CHS 0.00001 0.00001

rs6111609 FIN & CDX 0.00000 0.00000

rs6111609 FIN & KHV 0.00000 0.00000

rs6111609 FIN & CHB 0.00000 0.00000

rs6111609 FIN & JPT 0.00003 0.00003

rs6111609 FIN & CEU 0.59849 0.00274

rs6111609 GBR & ACB 0.00000 0.00000

rs6111609 GBR & ASW 0.00000 0.00000

rs6111609 GBR & ESN 0.00000 0.00000

rs6111609 GBR & GWD 0.00000 0.00000

rs6111609 GBR & LWK 0.00000 0.00000

rs6111609 GBR & MSL 0.00000 0.00000

rs6111609 GBR & YRI 0.00000 0.00000

rs6111609 GBR & PUR 0.01257 0.00077

rs6111609 GBR & CLM 0.00202 0.00020

rs6111609 GBR & PEL 0.00000 0.00000

rs6111609 GBR & MXL 0.00022 0.00008

rs6111609 GBR & CHS 0.00000 0.00000

rs6111609 GBR & CDX 0.00000 0.00000

rs6111609 GBR & KHV 0.00000 0.00000

rs6111609 GBR & CHB 0.00000 0.00000

rs6111609 GBR & JPT 0.00000 0.00000

rs6111609 GBR & CEU 0.31173 0.00363

rs6111609 GBR & FIN 0.09438 0.00199

rs6111609 IBS & ACB 0.00000 0.00000

rs6111609 IBS & ASW 0.00000 0.00000

rs6111609 IBS & ESN 0.00000 0.00000

rs6111609 IBS & GWD 0.00000 0.00000

rs6111609 IBS & LWK 0.00000 0.00000

rs6111609 IBS & MSL 0.00000 0.00000

rs6111609 IBS & YRI 0.00000 0.00000

rs6111609 IBS & PUR 0.00419 0.00039

rs6111609 IBS & CLM 0.00052 0.00011

rs6111609 IBS & PEL 0.00000 0.00000

rs6111609 IBS & MXL 0.00016 0.00005

rs6111609 IBS & CHS 0.00000 0.00000

rs6111609 IBS & CDX 0.00000 0.00000

rs6111609 IBS & KHV 0.00000 0.00000

rs6111609 IBS & CHB 0.00000 0.00000

rs6111609 IBS & JPT 0.00000 0.00000

rs6111609 IBS & CEU 0.18560 0.00266

rs6111609 IBS & FIN 0.04947 0.00175

rs6111609 IBS & GBR 0.80881 0.00167

rs6111609 TSI & ACB 0.00000 0.00000

rs6111609 TSI & ASW 0.00000 0.00000

rs6111609 TSI & ESN 0.00000 0.00000

rs6111609 TSI & GWD 0.00000 0.00000

rs6111609 TSI & LWK 0.00000 0.00000

rs6111609 TSI & MSL 0.00000 0.00000

rs6111609 TSI & YRI 0.00000 0.00000

rs6111609 TSI & PUR 0.03802 0.00131

rs6111609 TSI & CLM 0.00601 0.00045

rs6111609 TSI & PEL 0.00002 0.00001

rs6111609 TSI & MXL 0.00121 0.00016

rs6111609 TSI & CHS 0.00000 0.00000

rs6111609 TSI & CDX 0.00000 0.00000

rs6111609 TSI & KHV 0.00000 0.00000

rs6111609 TSI & CHB 0.00000 0.00000

rs6111609 TSI & JPT 0.00000 0.00000

rs6111609 TSI & CEU 0.62280 0.00264

rs6111609 TSI & FIN 0.20873 0.00259

rs6111609 TSI & GBR 0.63179 0.00274

rs6111609 TSI & IBS 0.49102 0.00317

rs6111609 BEB & ACB 0.03672 0.00076

rs6111609 BEB & ASW 0.05086 0.00079

rs6111609 BEB & ESN 0.00003 0.00001

rs6111609 BEB & GWD 0.00075 0.00006

rs6111609 BEB & LWK 0.03669 0.00069

rs6111609 BEB & MSL 0.00038 0.00006

rs6111609 BEB & YRI 0.00004 0.00001

rs6111609 BEB & PUR 0.06276 0.00134

rs6111609 BEB & CLM 0.27138 0.00205

rs6111609 BEB & PEL 0.82506 0.00076

rs6111609 BEB & MXL 0.82430 0.00081

rs6111609 BEB & CHS 0.08599 0.00103

rs6111609 BEB & CDX 0.03864 0.00071

rs6111609 BEB & KHV 0.03660 0.00067

rs6111609 BEB & CHB 0.01972 0.00050

rs6111609 BEB & JPT 0.15516 0.00147

rs6111609 BEB & CEU 0.00095 0.00013

rs6111609 BEB & FIN 0.00755 0.00047

rs6111609 BEB & GBR 0.00002 0.00001

rs6111609 BEB & IBS 0.00000 0.00000

rs6111609 BEB & TSI 0.00015 0.00005

rs6111609 GIH & ACB 0.00141 0.00014

rs6111609 GIH & ASW 0.00313 0.00020

rs6111609 GIH & ESN 0.00000 0.00000

rs6111609 GIH & GWD 0.00000 0.00000

rs6111609 GIH & LWK 0.00104 0.00010

rs6111609 GIH & MSL 0.00000 0.00000

rs6111609 GIH & YRI 0.00000 0.00000

rs6111609 GIH & PUR 0.35824 0.00259

rs6111609 GIH & CLM 0.86935 0.00089

rs6111609 GIH & PEL 0.18642 0.00191

rs6111609 GIH & MXL 0.56653 0.00211

rs6111609 GIH & CHS 0.00404 0.00027

rs6111609 GIH & CDX 0.00225 0.00017

rs6111609 GIH & KHV 0.00130 0.00012

rs6111609 GIH & CHB 0.00108 0.00011

rs6111609 GIH & JPT 0.00988 0.00046

rs6111609 GIH & CEU 0.01150 0.00069

rs6111609 GIH & FIN 0.05737 0.00168

rs6111609 GIH & GBR 0.00049 0.00007

rs6111609 GIH & IBS 0.00001 0.00001

rs6111609 GIH & TSI 0.00228 0.00028

rs6111609 GIH & BEB 0.36162 0.00230

rs6111609 ITU & ACB 1.00000 0.00000

rs6111609 ITU & ASW 0.71757 0.00083

rs6111609 ITU & ESN 0.06097 0.00060

rs6111609 ITU & GWD 0.10611 0.00084

rs6111609 ITU & LWK 1.00000 0.00000

rs6111609 ITU & MSL 0.06586 0.00062

rs6111609 ITU & YRI 0.02642 0.00041

rs6111609 ITU & PUR 0.00004 0.00002

rs6111609 ITU & CLM 0.00147 0.00015

rs6111609 ITU & PEL 0.11357 0.00126

rs6111609 ITU & MXL 0.02807 0.00065

rs6111609 ITU & CHS 1.00000 0.00000

rs6111609 ITU & CDX 1.00000 0.00000

rs6111609 ITU & KHV 1.00000 0.00000

rs6111609 ITU & CHB 0.74930 0.00079

rs6111609 ITU & JPT 0.77047 0.00075

rs6111609 ITU & CEU 0.00000 0.00000

rs6111609 ITU & FIN 0.00001 0.00001

rs6111609 ITU & GBR 0.00000 0.00000

rs6111609 ITU & IBS 0.00000 0.00000

rs6111609 ITU & TSI 0.00000 0.00000

rs6111609 ITU & BEB 0.04497 0.00081

rs6111609 ITU & GIH 0.00322 0.00020

rs6111609 PJL & ACB 0.07045 0.00087

rs6111609 PJL & ASW 0.05649 0.00099

rs6111609 PJL & ESN 0.00017 0.00003

rs6111609 PJL & GWD 0.00077 0.00009

rs6111609 PJL & LWK 0.04199 0.00070

rs6111609 PJL & MSL 0.00051 0.00006

rs6111609 PJL & YRI 0.00008 0.00002

rs6111609 PJL & PUR 0.02820 0.00099

rs6111609 PJL & CLM 0.14146 0.00161

rs6111609 PJL & PEL 1.00000 0.00000

rs6111609 PJL & MXL 0.65400 0.00152

rs6111609 PJL & CHS 0.14671 0.00121

rs6111609 PJL & CDX 0.07171 0.00103

rs6111609 PJL & KHV 0.04240 0.00079

rs6111609 PJL & CHB 0.03955 0.00079

rs6111609 PJL & JPT 0.23987 0.00186

rs6111609 PJL & CEU 0.00018 0.00005

rs6111609 PJL & FIN 0.00236 0.00023

rs6111609 PJL & GBR 0.00000 0.00000

rs6111609 PJL & IBS 0.00000 0.00000

rs6111609 PJL & TSI 0.00005 0.00004

rs6111609 PJL & BEB 0.83483 0.00081

rs6111609 PJL & GIH 0.26886 0.00225

rs6111609 PJL & ITU 0.08181 0.00108

rs6111609 STU & ACB 0.75172 0.00088

rs6111609 STU & ASW 0.50077 0.00127

rs6111609 STU & ESN 0.03085 0.00043

rs6111609 STU & GWD 0.05586 0.00064

rs6111609 STU & LWK 0.75040 0.00076

rs6111609 STU & MSL 0.03358 0.00047

rs6111609 STU & YRI 0.01262 0.00029

rs6111609 STU & PUR 0.00021 0.00005

rs6111609 STU & CLM 0.00388 0.00024

rs6111609 STU & PEL 0.20330 0.00172

rs6111609 STU & MXL 0.06313 0.00093

rs6111609 STU & CHS 1.00000 0.00000

rs6111609 STU & CDX 0.75207 0.00077

rs6111609 STU & KHV 0.75203 0.00070

rs6111609 STU & CHB 0.54240 0.00123

rs6111609 STU & JPT 1.00000 0.00000

rs6111609 STU & CEU 0.00000 0.00000

rs6111609 STU & FIN 0.00000 0.00000

rs6111609 STU & GBR 0.00000 0.00000

rs6111609 STU & IBS 0.00000 0.00000

rs6111609 STU & TSI 0.00000 0.00000

rs6111609 STU & BEB 0.08796 0.00126

rs6111609 STU & GIH 0.00767 0.00036

rs6111609 STU & ITU 1.00000 0.00000

rs6111609 STU & PJL 0.14999 0.00146

Locus: rs2922921

=================================

Pop Alleles:

-----------------------------------------------------------

1 3 Total

ACB 1 191 192

ASW 0 122 122

ESN 0 198 198

GWD 0 226 226

LWK 0 198 198

MSL 0 170 170

YRI 0 216 216

PUR 3 205 208

CLM 1 187 188

PEL 1 169 170

MXL 0 128 128

CHS 0 210 210

CDX 0 186 186

KHV 0 198 198

CHB 0 206 206

JPT 0 208 208

CEU 4 194 198

FIN 1 197 198

GBR 0 182 182

IBS 4 210 214

TSI 9 205 214

BEB 2 170 172

GIH 13 193 206

ITU 5 199 204

PJL 7 185 192

STU 7 197 204

Total: 58 4950 5008

Locus Population pair P-Value S.E.

----------- --------------------- ------- -------

rs2922921 ASW & ACB 1.00000 0.00000

rs2922921 ESN & ACB 0.49162 0.00061

rs2922921 ESN & ASW No table

rs2922921 GWD & ACB 0.45963 0.00072

rs2922921 GWD & ASW No table

rs2922921 GWD & ESN No table

rs2922921 LWK & ACB 0.49250 0.00065

rs2922921 LWK & ASW No table

rs2922921 LWK & ESN No table

rs2922921 LWK & GWD No table

rs2922921 MSL & ACB 1.00000 0.00000

rs2922921 MSL & ASW No table

rs2922921 MSL & ESN No table

rs2922921 MSL & GWD No table

rs2922921 MSL & LWK No table

rs2922921 YRI & ACB 0.47123 0.00066

rs2922921 YRI & ASW No table

rs2922921 YRI & ESN No table

rs2922921 YRI & GWD No table

rs2922921 YRI & LWK No table

rs2922921 YRI & MSL No table

rs2922921 PUR & ACB 0.62555 0.00075

rs2922921 PUR & ASW 0.29925 0.00081

rs2922921 PUR & ESN 0.24785 0.00087

rs2922921 PUR & GWD 0.11023 0.00067

rs2922921 PUR & LWK 0.24840 0.00089

rs2922921 PUR & MSL 0.25511 0.00073

rs2922921 PUR & YRI 0.11672 0.00093

rs2922921 CLM & ACB 1.00000 0.00000

rs2922921 CLM & ASW 1.00000 0.00000

rs2922921 CLM & ESN 0.48607 0.00069

rs2922921 CLM & GWD 0.45382 0.00094

rs2922921 CLM & LWK 0.48708 0.00074

rs2922921 CLM & MSL 1.00000 0.00000

rs2922921 CLM & YRI 0.46586 0.00085

rs2922921 CLM & PUR 0.62526 0.00067

rs2922921 PEL & ACB 1.00000 0.00000

rs2922921 PEL & ASW 1.00000 0.00000

rs2922921 PEL & ESN 0.46150 0.00080

rs2922921 PEL & GWD 0.43031 0.00076

rs2922921 PEL & LWK 0.46244 0.00067

rs2922921 PEL & MSL 1.00000 0.00000

rs2922921 PEL & YRI 0.43895 0.00085

rs2922921 PEL & PUR 0.63055 0.00071

rs2922921 PEL & CLM 1.00000 0.00000

rs2922921 MXL & ACB 1.00000 0.00000

rs2922921 MXL & ASW No table

rs2922921 MXL & ESN No table

rs2922921 MXL & GWD No table

rs2922921 MXL & LWK No table

rs2922921 MXL & MSL No table

rs2922921 MXL & YRI No table

rs2922921 MXL & PUR 0.29057 0.00094

rs2922921 MXL & CLM 1.00000 0.00000

rs2922921 MXL & PEL 1.00000 0.00000

rs2922921 CHS & ACB 0.47682 0.00070

rs2922921 CHS & ASW No table

rs2922921 CHS & ESN No table

rs2922921 CHS & GWD No table

rs2922921 CHS & LWK No table

rs2922921 CHS & MSL No table

rs2922921 CHS & YRI No table

rs2922921 CHS & PUR 0.12259 0.00077

rs2922921 CHS & CLM 0.47200 0.00075

rs2922921 CHS & PEL 0.44863 0.00087

rs2922921 CHS & MXL No table

rs2922921 CDX & ACB 1.00000 0.00000

rs2922921 CDX & ASW No table

rs2922921 CDX & ESN No table

rs2922921 CDX & GWD No table

rs2922921 CDX & LWK No table

rs2922921 CDX & MSL No table

rs2922921 CDX & YRI No table

rs2922921 CDX & PUR 0.25095 0.00086

rs2922921 CDX & CLM 1.00000 0.00000

rs2922921 CDX & PEL 0.47862 0.00072

rs2922921 CDX & MXL No table

rs2922921 CDX & CHS No table

rs2922921 KHV & ACB 0.49233 0.00076

rs2922921 KHV & ASW No table

rs2922921 KHV & ESN No table

rs2922921 KHV & GWD No table

rs2922921 KHV & LWK No table

rs2922921 KHV & MSL No table

rs2922921 KHV & YRI No table

rs2922921 KHV & PUR 0.24892 0.00087

rs2922921 KHV & CLM 0.48667 0.00067

rs2922921 KHV & PEL 0.46195 0.00069

rs2922921 KHV & MXL No table

rs2922921 KHV & CHS No table

rs2922921 KHV & CDX No table

rs2922921 CHB & ACB 0.48290 0.00079

rs2922921 CHB & ASW No table

rs2922921 CHB & ESN No table

rs2922921 CHB & GWD No table

rs2922921 CHB & LWK No table

rs2922921 CHB & MSL No table

rs2922921 CHB & YRI No table

rs2922921 CHB & PUR 0.24719 0.00094

rs2922921 CHB & CLM 0.47615 0.00081

rs2922921 CHB & PEL 0.45059 0.00082

rs2922921 CHB & MXL No table

rs2922921 CHB & CHS No table

rs2922921 CHB & CDX No table

rs2922921 CHB & KHV No table

rs2922921 JPT & ACB 0.47906 0.00072

rs2922921 JPT & ASW No table

rs2922921 JPT & ESN No table

rs2922921 JPT & GWD No table

rs2922921 JPT & LWK No table

rs2922921 JPT & MSL No table

rs2922921 JPT & YRI No table

rs2922921 JPT & PUR 0.24765 0.00083

rs2922921 JPT & CLM 0.47520 0.00087

rs2922921 JPT & PEL 0.44886 0.00075

rs2922921 JPT & MXL No table

rs2922921 JPT & CHS No table

rs2922921 JPT & CDX No table

rs2922921 JPT & KHV No table

rs2922921 JPT & CHB No table

rs2922921 CEU & ACB 0.37275 0.00108

rs2922921 CEU & ASW 0.16582 0.00079

rs2922921 CEU & ESN 0.12248 0.00068

rs2922921 CEU & GWD 0.04739 0.00056

rs2922921 CEU & LWK 0.12404 0.00072

rs2922921 CEU & MSL 0.12718 0.00071

rs2922921 CEU & YRI 0.05129 0.00054

rs2922921 CEU & PUR 0.71996 0.00077

rs2922921 CEU & CLM 0.37181 0.00107

rs2922921 CEU & PEL 0.37985 0.00112

rs2922921 CEU & MXL 0.15598 0.00091

rs2922921 CEU & CHS 0.05514 0.00054

rs2922921 CEU & CDX 0.12449 0.00066

rs2922921 CEU & KHV 0.12182 0.00073

rs2922921 CEU & CHB 0.05597 0.00059

rs2922921 CEU & JPT 0.05512 0.00068

rs2922921 FIN & ACB 1.00000 0.00000

rs2922921 FIN & ASW 1.00000 0.00000

rs2922921 FIN & ESN 1.00000 0.00000

rs2922921 FIN & GWD 0.46771 0.00078

rs2922921 FIN & LWK 1.00000 0.00000

rs2922921 FIN & MSL 1.00000 0.00000

rs2922921 FIN & YRI 0.47731 0.00065

rs2922921 FIN & PUR 0.62377 0.00079

rs2922921 FIN & CLM 1.00000 0.00000

rs2922921 FIN & PEL 1.00000 0.00000

rs2922921 FIN & MXL 1.00000 0.00000

rs2922921 FIN & CHS 0.48572 0.00069

rs2922921 FIN & CDX 1.00000 0.00000

rs2922921 FIN & KHV 1.00000 0.00000

rs2922921 FIN & CHB 0.49033 0.00071

rs2922921 FIN & JPT 0.48739 0.00076

rs2922921 FIN & CEU 0.37229 0.00112

rs2922921 GBR & ACB 1.00000 0.00000

rs2922921 GBR & ASW No table

rs2922921 GBR & ESN No table

rs2922921 GBR & GWD No table

rs2922921 GBR & LWK No table

rs2922921 GBR & MSL No table

rs2922921 GBR & YRI No table

rs2922921 GBR & PUR 0.25127 0.00088

rs2922921 GBR & CLM 1.00000 0.00000

rs2922921 GBR & PEL 0.48457 0.00080

rs2922921 GBR & MXL No table

rs2922921 GBR & CHS No table

rs2922921 GBR & CDX No table

rs2922921 GBR & KHV No table

rs2922921 GBR & CHB No table

rs2922921 GBR & JPT No table

rs2922921 GBR & CEU 0.12419 0.00076

rs2922921 GBR & FIN 1.00000 0.00000

rs2922921 IBS & ACB 0.37600 0.00096

rs2922921 IBS & ASW 0.18124 0.00093

rs2922921 IBS & ESN 0.12381 0.00073

rs2922921 IBS & GWD 0.05484 0.00049

rs2922921 IBS & LWK 0.12435 0.00072

rs2922921 IBS & MSL 0.13225 0.00074

rs2922921 IBS & YRI 0.06074 0.00059

rs2922921 IBS & PUR 1.00000 0.00000

rs2922921 IBS & CLM 0.37757 0.00106

rs2922921 IBS & PEL 0.38880 0.00105

rs2922921 IBS & MXL 0.17170 0.00089

rs2922921 IBS & CHS 0.12382 0.00081

rs2922921 IBS & CDX 0.12614 0.00081

rs2922921 IBS & KHV 0.12417 0.00080

rs2922921 IBS & CHB 0.12451 0.00077

rs2922921 IBS & JPT 0.12255 0.00077

rs2922921 IBS & CEU 1.00000 0.00000

rs2922921 IBS & FIN 0.37409 0.00112

rs2922921 IBS & GBR 0.12735 0.00079

rs2922921 TSI & ACB 0.02131 0.00040

rs2922921 TSI & ASW 0.01848 0.00034

rs2922921 TSI & ESN 0.00406 0.00019

rs2922921 TSI & GWD 0.00147 0.00009

rs2922921 TSI & LWK 0.00374 0.00015

rs2922921 TSI & MSL 0.00520 0.00019

rs2922921 TSI & YRI 0.00162 0.00010

rs2922921 TSI & PUR 0.14045 0.00126

rs2922921 TSI & CLM 0.02275 0.00047

rs2922921 TSI & PEL 0.02773 0.00057

rs2922921 TSI & MXL 0.01580 0.00035

rs2922921 TSI & CHS 0.00369 0.00016

rs2922921 TSI & CDX 0.00412 0.00018

rs2922921 TSI & KHV 0.00397 0.00016

rs2922921 TSI & CHB 0.00369 0.00016

rs2922921 TSI & JPT 0.00374 0.00017

rs2922921 TSI & CEU 0.26662 0.00161

rs2922921 TSI & FIN 0.02035 0.00047

rs2922921 TSI & GBR 0.00457 0.00018

rs2922921 TSI & IBS 0.25669 0.00144

rs2922921 BEB & ACB 0.60457 0.00075

rs2922921 BEB & ASW 0.51259 0.00079

rs2922921 BEB & ESN 0.21393 0.00086

rs2922921 BEB & GWD 0.18828 0.00084

rs2922921 BEB & LWK 0.21534 0.00096

rs2922921 BEB & MSL 0.49842 0.00083

rs2922921 BEB & YRI 0.19470 0.00090

rs2922921 BEB & PUR 1.00000 0.00000

rs2922921 BEB & CLM 0.60695 0.00079

rs2922921 BEB & PEL 1.00000 0.00000

rs2922921 BEB & MXL 0.50797 0.00065

rs2922921 BEB & CHS 0.20048 0.00090

rs2922921 BEB & CDX 0.22996 0.00099

rs2922921 BEB & KHV 0.21648 0.00090

rs2922921 BEB & CHB 0.20627 0.00079

rs2922921 BEB & JPT 0.20501 0.00091

rs2922921 BEB & CEU 0.69043 0.00078

rs2922921 BEB & FIN 0.59869 0.00065

rs2922921 BEB & GBR 0.23567 0.00087

rs2922921 BEB & IBS 0.69624 0.00070

rs2922921 BEB & TSI 0.12100 0.00108

rs2922921 GIH & ACB 0.00156 0.00012

rs2922921 GIH & ASW 0.00257 0.00013

rs2922921 GIH & ESN 0.00022 0.00004

rs2922921 GIH & GWD 0.00007 0.00002

rs2922921 GIH & LWK 0.00019 0.00004

rs2922921 GIH & MSL 0.00044 0.00007

rs2922921 GIH & YRI 0.00007 0.00003

rs2922921 GIH & PUR 0.01013 0.00037

rs2922921 GIH & CLM 0.00171 0.00011

rs2922921 GIH & PEL 0.00235 0.00013

rs2922921 GIH & MXL 0.00177 0.00012

rs2922921 GIH & CHS 0.00014 0.00003

rs2922921 GIH & CDX 0.00020 0.00004

rs2922921 GIH & KHV 0.00016 0.00003

rs2922921 GIH & CHB 0.00016 0.00003

rs2922921 GIH & JPT 0.00010 0.00003

rs2922921 GIH & CEU 0.04392 0.00085

rs2922921 GIH & FIN 0.00159 0.00012

rs2922921 GIH & GBR 0.00024 0.00003

rs2922921 GIH & IBS 0.02586 0.00067

rs2922921 GIH & TSI 0.38568 0.00196

rs2922921 GIH & BEB 0.01501 0.00044

rs2922921 ITU & ACB 0.21743 0.00108

rs2922921 ITU & ASW 0.10131 0.00085

rs2922921 ITU & ESN 0.06178 0.00052

rs2922921 ITU & GWD 0.02340 0.00047

rs2922921 ITU & LWK 0.06124 0.00058

rs2922921 ITU & MSL 0.06628 0.00071

rs2922921 ITU & YRI 0.02683 0.00043

rs2922921 ITU & PUR 0.49823 0.00122

rs2922921 ITU & CLM 0.21728 0.00115

rs2922921 ITU & PEL 0.22603 0.00103

rs2922921 ITU & MXL 0.09407 0.00072

rs2922921 ITU & CHS 0.02794 0.00043

rs2922921 ITU & CDX 0.06211 0.00059

rs2922921 ITU & KHV 0.06075 0.00062

rs2922921 ITU & CHB 0.02876 0.00047

rs2922921 ITU & JPT 0.02898 0.00046

rs2922921 ITU & CEU 1.00000 0.00000

rs2922921 ITU & FIN 0.21628 0.00115

rs2922921 ITU & GBR 0.06212 0.00069

rs2922921 ITU & IBS 0.74641 0.00082

rs2922921 ITU & TSI 0.41869 0.00153

rs2922921 ITU & BEB 0.45990 0.00136

rs2922921 ITU & GIH 0.08742 0.00129

rs2922921 PJL & ACB 0.06713 0.00075

rs2922921 PJL & ASW 0.03200 0.00047

rs2922921 PJL & ESN 0.00655 0.00020

rs2922921 PJL & GWD 0.00412 0.00016

rs2922921 PJL & LWK 0.00631 0.00019

rs2922921 PJL & MSL 0.01582 0.00031

rs2922921 PJL & YRI 0.00471 0.00016

rs2922921 PJL & PUR 0.20615 0.00114

rs2922921 PJL & CLM 0.06760 0.00071

rs2922921 PJL & PEL 0.07181 0.00080

rs2922921 PJL & MXL 0.02864 0.00049

rs2922921 PJL & CHS 0.00491 0.00018

rs2922921 PJL & CDX 0.01511 0.00031

rs2922921 PJL & KHV 0.00628 0.00020

rs2922921 PJL & CHB 0.00590 0.00020

rs2922921 PJL & JPT 0.00565 0.00022

rs2922921 PJL & CEU 0.37388 0.00136

rs2922921 PJL & FIN 0.03438 0.00060

rs2922921 PJL & GBR 0.01524 0.00033

rs2922921 PJL & IBS 0.36354 0.00145

rs2922921 PJL & TSI 0.80466 0.00090

rs2922921 PJL & BEB 0.18137 0.00108

rs2922921 PJL & GIH 0.25670 0.00187

rs2922921 PJL & ITU 0.56494 0.00126

rs2922921 STU & ACB 0.06856 0.00074

rs2922921 STU & ASW 0.04908 0.00058

rs2922921 STU & ESN 0.01481 0.00031

rs2922921 STU & GWD 0.00483 0.00017

rs2922921 STU & LWK 0.01462 0.00032

rs2922921 STU & MSL 0.01763 0.00036

rs2922921 STU & YRI 0.00616 0.00021

rs2922921 STU & PUR 0.21707 0.00125

rs2922921 STU & CLM 0.06920 0.00072

rs2922921 STU & PEL 0.07659 0.00078

rs2922921 STU & MXL 0.03370 0.00048

rs2922921 STU & CHS 0.00641 0.00020

rs2922921 STU & CDX 0.01508 0.00032

rs2922921 STU & KHV 0.01450 0.00035

rs2922921 STU & CHB 0.00705 0.00021

rs2922921 STU & JPT 0.00749 0.00021

rs2922921 STU & CEU 0.54337 0.00125

rs2922921 STU & FIN 0.06716 0.00078

rs2922921 STU & GBR 0.01574 0.00031

rs2922921 STU & IBS 0.37150 0.00146

rs2922921 STU & TSI 0.80116 0.00077

rs2922921 STU & BEB 0.18818 0.00128

rs2922921 STU & GIH 0.25056 0.00189

rs2922921 STU & ITU 0.77045 0.00092

rs2922921 STU & PJL 1.00000 0.00000

Locus: rs3922636

=================================

Pop Alleles:

----------------------------------------

1 2 3 Total

ACB 7 3 182 192

ASW 0 2 120 122

ESN 0 1 197 198

GWD 0 2 224 226

LWK 0 2 196 198

MSL 0 3 167 170

YRI 1 0 215 216

PUR 52 1 155 208

CLM 39 0 149 188

PEL 27 0 143 170

MXL 25 0 103 128

CHS 52 0 158 210

CDX 53 0 133 186

KHV 37 0 161 198

CHB 42 0 164 206

JPT 46 0 162 208

CEU 37 0 161 198

FIN 47 0 151 198

GBR 34 0 148 182

IBS 41 0 173 214

TSI 50 0 164 214

BEB 39 0 133 172

GIH 78 0 128 206

ITU 57 0 147 204

PJL 43 0 149 192

STU 56 0 148 204

Total: 863 14 4131 5008

Locus Population pair P-Value S.E.

----------- --------------------- ------- -------

rs3922636  ASW & ACB 0.06749 0.00110

rs3922636  ESN & ACB 0.00327 0.00020

rs3922636  ESN & ASW 0.55996 0.00075

rs3922636  GWD & ACB 0.00394 0.00022

rs3922636  GWD & ASW 0.61672 0.00085

rs3922636  GWD & ESN 1.00000 0.00000

rs3922636  LWK & ACB 0.01378 0.00040

rs3922636  LWK & ASW 1.00000 0.00000

rs3922636  LWK & ESN 1.00000 0.00000

rs3922636  LWK & GWD 1.00000 0.00000

rs3922636  MSL & ACB 0.03091 0.00059

rs3922636  MSL & ASW 1.00000 0.00000

rs3922636  MSL & ESN 0.34073 0.00104

rs3922636  MSL & GWD 0.65506 0.00075

rs3922636  MSL & LWK 0.66503 0.00085

rs3922636  YRI & ACB 0.01014 0.00041

rs3922636  YRI & ASW 0.13015 0.00112

rs3922636  YRI & ESN 0.72858 0.00128

rs3922636  YRI & GWD 0.36731 0.00114

rs3922636  YRI & LWK 0.22877 0.00147

rs3922636  YRI & MSL 0.08278 0.00103

rs3922636  PUR & ACB 0.00000 0.00000

rs3922636  PUR & ASW 0.00000 0.00000

rs3922636  PUR & ESN 0.00000 0.00000

rs3922636  PUR & GWD 0.00000 0.00000

rs3922636  PUR & LWK 0.00000 0.00000

rs3922636  PUR & MSL 0.00000 0.00000

rs3922636  PUR & YRI 0.00000 0.00000

rs3922636  CLM & ACB 0.00000 0.00000

rs3922636  CLM & ASW 0.00000 0.00000

rs3922636  CLM & ESN 0.00000 0.00000

rs3922636  CLM & GWD 0.00000 0.00000

rs3922636  CLM & LWK 0.00000 0.00000

rs3922636  CLM & MSL 0.00000 0.00000

rs3922636  CLM & YRI 0.00000 0.00000

rs3922636  CLM & PUR 0.38042 0.00520

rs3922636  PEL & ACB 0.00005 0.00004

rs3922636  PEL & ASW 0.00000 0.00000

rs3922636  PEL & ESN 0.00000 0.00000

rs3922636  PEL & GWD 0.00000 0.00000

rs3922636  PEL & LWK 0.00000 0.00000

rs3922636  PEL & MSL 0.00000 0.00000

rs3922636  PEL & YRI 0.00000 0.00000

rs3922636  PEL & PUR 0.03586 0.00197

rs3922636  PEL & CLM 0.27555 0.00346

rs3922636  MXL & ACB 0.00000 0.00000

rs3922636  MXL & ASW 0.00000 0.00000

rs3922636  MXL & ESN 0.00000 0.00000

rs3922636  MXL & GWD 0.00000 0.00000

rs3922636  MXL & LWK 0.00000 0.00000

rs3922636  MXL & MSL 0.00000 0.00000

rs3922636  MXL & YRI 0.00000 0.00000

rs3922636  MXL & PUR 0.36246 0.00440

rs3922636  MXL & CLM 0.88657 0.00086

rs3922636  MXL & PEL 0.44688 0.00271

rs3922636  CHS & ACB 0.00000 0.00000

rs3922636  CHS & ASW 0.00000 0.00000

rs3922636  CHS & ESN 0.00000 0.00000

rs3922636  CHS & GWD 0.00000 0.00000

rs3922636  CHS & LWK 0.00000 0.00000

rs3922636  CHS & MSL 0.00000 0.00000

rs3922636  CHS & YRI 0.00000 0.00000

rs3922636  CHS & PUR 0.95698 0.00080

rs3922636  CHS & CLM 0.40503 0.00339

rs3922636  CHS & PEL 0.04382 0.00134

rs3922636  CHS & MXL 0.28707 0.00283

rs3922636  CDX & ACB 0.00000 0.00000

rs3922636  CDX & ASW 0.00000 0.00000

rs3922636  CDX & ESN 0.00000 0.00000

rs3922636  CDX & GWD 0.00000 0.00000

rs3922636  CDX & LWK 0.00000 0.00000

rs3922636  CDX & MSL 0.00000 0.00000

rs3922636  CDX & YRI 0.00000 0.00000

rs3922636  CDX & PUR 0.51829 0.00551

rs3922636  CDX & CLM 0.09579 0.00247

rs3922636  CDX & PEL 0.00576 0.00045

rs3922636  CDX & MXL 0.08257 0.00195

rs3922636  CDX & CHS 0.42498 0.00331

rs3922636  KHV & ACB 0.00000 0.00000

rs3922636  KHV & ASW 0.00000 0.00000

rs3922636  KHV & ESN 0.00000 0.00000

rs3922636  KHV & GWD 0.00000 0.00000

rs3922636  KHV & LWK 0.00000 0.00000

rs3922636  KHV & MSL 0.00000 0.00000

rs3922636  KHV & YRI 0.00000 0.00000

rs3922636  KHV & PUR 0.11820 0.00359

rs3922636  KHV & CLM 0.69808 0.00207

rs3922636  KHV & PEL 0.49759 0.00293

rs3922636  KHV & MXL 0.88542 0.00085

rs3922636  KHV & CHS 0.14967 0.00301

rs3922636  KHV & CDX 0.02939 0.00123

rs3922636  CHB & ACB 0.00000 0.00000

rs3922636  CHB & ASW 0.00000 0.00000

rs3922636  CHB & ESN 0.00000 0.00000

rs3922636  CHB & GWD 0.00000 0.00000

rs3922636  CHB & LWK 0.00000 0.00000

rs3922636  CHB & MSL 0.00000 0.00000

rs3922636  CHB & YRI 0.00000 0.00000

rs3922636  CHB & PUR 0.26659 0.00482

rs3922636  CHB & CLM 1.00000 0.00000

rs3922636  CHB & PEL 0.28340 0.00349

rs3922636  CHB & MXL 0.89007 0.00084

rs3922636  CHB & CHS 0.28885 0.00341

rs3922636  CHB & CDX 0.07776 0.00187

rs3922636  CHB & KHV 0.70764 0.00223

rs3922636  JPT & ACB 0.00000 0.00000

rs3922636  JPT & ASW 0.00000 0.00000

rs3922636  JPT & ESN 0.00000 0.00000

rs3922636  JPT & GWD 0.00000 0.00000

rs3922636  JPT & LWK 0.00000 0.00000

rs3922636  JPT & MSL 0.00000 0.00000

rs3922636  JPT & YRI 0.00000 0.00000

rs3922636  JPT & PUR 0.48167 0.00421

rs3922636  JPT & CLM 0.80742 0.00175

rs3922636  JPT & PEL 0.14961 0.00227

rs3922636  JPT & MXL 0.58282 0.00258

rs3922636  JPT & CHS 0.56620 0.00350

rs3922636  JPT & CDX 0.16025 0.00304

rs3922636  JPT & KHV 0.45954 0.00340

rs3922636  JPT & CHB 0.71706 0.00196

rs3922636  CEU & ACB 0.00000 0.00000

rs3922636  CEU & ASW 0.00000 0.00000

rs3922636  CEU & ESN 0.00000 0.00000

rs3922636  CEU & GWD 0.00000 0.00000

rs3922636  CEU & LWK 0.00000 0.00000

rs3922636  CEU & MSL 0.00000 0.00000

rs3922636  CEU & YRI 0.00000 0.00000

rs3922636  CEU & PUR 0.11776 0.00351

rs3922636  CEU & CLM 0.70088 0.00215

rs3922636  CEU & PEL 0.49687 0.00298

rs3922636  CEU & MXL 0.88427 0.00084

rs3922636  CEU & CHS 0.15219 0.00282

rs3922636  CEU & CDX 0.02751 0.00102

rs3922636  CEU & KHV 1.00000 0.00000

rs3922636  CEU & CHB 0.70551 0.00214

rs3922636  CEU & JPT 0.46333 0.00320

rs3922636  FIN & ACB 0.00000 0.00000

rs3922636  FIN & ASW 0.00000 0.00000

rs3922636  FIN & ESN 0.00000 0.00000
[truncated: 438,228 more chars]
